# Deboronative functionalization of alkylboron species *via* a radical-transfer strategy†

**DOI:** 10.1039/d4sc02889a

**Published:** 2024-08-08

**Authors:** Fuyang Yue, Mingxing Li, Kangkang Yang, Hongjian Song, Yuxiu Liu, Qingmin Wang

**Affiliations:** a State Key Laboratory of Elemento-Organic Chemistry, Research Institute of Elemento-Organic Chemistry, Frontiers Science Center for New Organic Matter, College of Chemistry, Nankai University Tianjin 300071 People's Republic of China wangqm@nankai.edu.cn

## Abstract

With advances in organoboron chemistry, boron-centered functional groups have become increasingly attractive. In particular, alkylboron species are highly versatile reagents for organic synthesis, but the direct generation of alkyl radicals from commonly used, bench-stable boron species has not been thoroughly investigated. Herein, we describe a method for activating C–B bonds by nitrogen- or oxygen-radical transfer that is applicable to alkylboronic acids and esters and can be used for both Michael addition reactions and Minisci reactions to generate alkyl or arylated products.

## Abstract 2

We describe a method for activating C–B bonds by nitrogen- or oxygen-radical transfer that is applicable to alkylboronic acids and esters.

## Graphical abstract

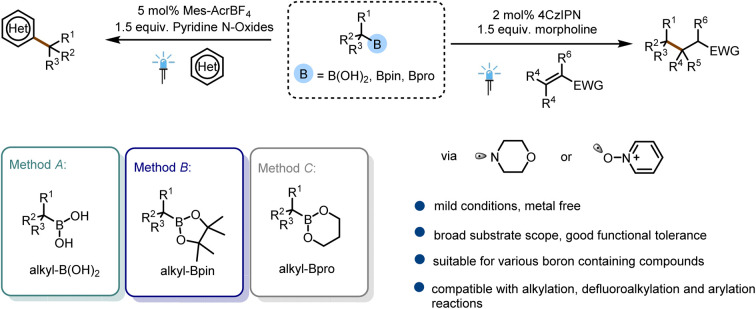

## Introduction

Organoboron compounds are versatile coupling reagents in modern organic chemistry because of their wide availability, bench stability, easy preparation, low toxicity, and structural diversity.^1^ Conventional reactions of organoboron compounds typically involve metal-catalyzed coupling *via* a closed-shell mechanism.^2^ However, alkylboronic acids are typically less reactive than arylboronic acids owing to the nonpolar nature of alkyl C–B bonds,^3^ and thus the effects of unfavorable polarity have hindered the widespread use of alkylboronic acids in certain coupling reactions.^3^ Recently, open-shell activation modes based on single electron transfer have emerged as a powerful strategy for overcoming the limitations imposed by the unfavorable polarity effects.^4^ The use of organoboron compounds as alkyl radical precursors is well established.^5^ However, direct activation of neutral organoboron compounds such as alkylboronic acids and alkylboronic pinacol esters has proved to be more challenging than the well-established methods employing alkylboranes^6^ and trifluoroborate^7^ as carbon-radical precursors. In recent years, chemists have made many attempts to use neutral boronic acids and esters in visible-light-based photochemistry applications and have developed a number elegant methods to get around the fact that their high oxidation potential prevents them from participating in photocatalytic reactions.^8^

**Fig. 1:**
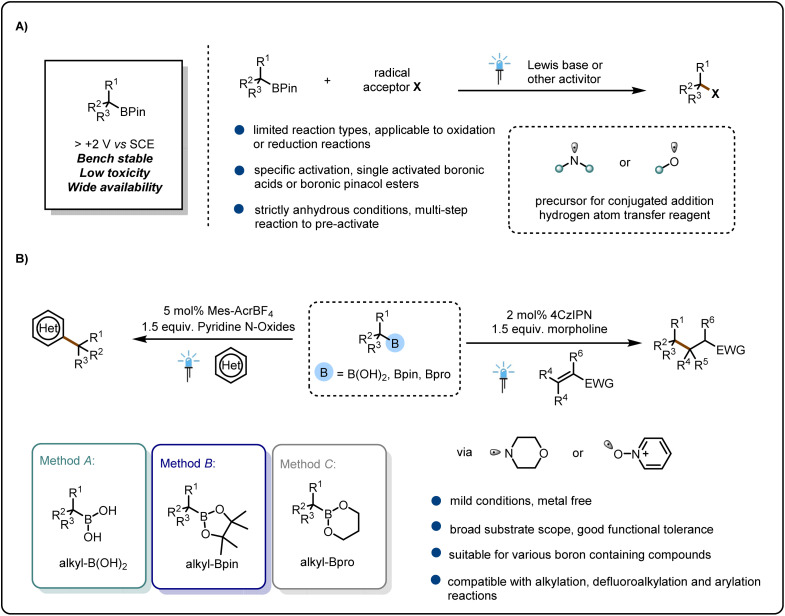
From inspiration to reaction design. (A) State-of-the-art in radical chemistry of boronic pinacol esters. (B) This work: amine/oxygen-radical-transfer strategy for generating C(sp^3^) radicals.

Most of these methods involve the introduction of nucleophilic reagents into the reaction system; through acid–base pairing, the lone-pair electrons of the nucleophilic reagents interact with the empty p orbitals of the boron atom to form electron-rich boron salt complexes, which have low oxidation potentials and can thus be directly oxidized by the photocatalysts to form carbon free radicals that can participate in subsequent reactions^5,8^ (Fig. 1A). Some investigators have activated alkylboronic esters by using strong bases, such as organic lithium reagents; the high nucleophilicity of these reagents allows them to form complexes with the esters.^9^ However, this activation mode requires strictly anhydrous conditions. Moreover, most of these methods can be used to activate only a specific class of boron compounds; for example, many methods can activate only alkylboronic acids and not alkylboronic esters. This lack of versatility is detrimental to the development of boron compounds and prevents chemists from achieving transformations of a wide variety of alkylboron compounds by means of a single class of methods. Therefore, it is important to find a universal method.^5–10^

Amine radicals have often been used as substrates for free-radical addition reactions or as inducers of hydrogen atom transfer reactions.^11^ These uses have been explored in elegant work reported by Lei *et al.*,^11^ Chen *et al.*,^12^ and others.^13^ In 2022, Maier and Speckmeier^14^ successfully activated alkyl borate esters by using amine radicals. These investigators developed a method for highly selective C(sp^2^)–C(sp^3^) cross-coupling reactions of aryl halides and alkyl borate esters, and they also demonstrated the excellent properties of the method. Very recently, the Studer group^15^ reported that reaction of amino radicals with alkyl borate esters generates alkyl radicals and that efficient alkyloximation reactions of olefins can be achieved under mild conditions by using alkyl borate esters and nitrosamines.

Inspired by the above-mentioned work, we envisioned the possibility of using simple heteroatom radicals to induce C–B bond cleavage to generate alkyl radicals and to develop a general strategy for activating a wide range of boronic acids. In this study, we found that the formation of an amine or oxygen radical by irradiation with visible light induced C–B bond cleavage to generate alkyl radicals, and we successfully applied this strategy to the Giese addition reaction (a reduction reaction) and the Minisci reaction (an oxidation reaction). The strategy is applicable to boronic acids, boronic pinacol esters, and boronic propylene glycol esters (Fig. 1B).

For our initial experiments, we chose *N*-phenylacrylamide (1a) and cyclohexylboronic acid (2a) as model substrates (Table 1). To our delight, when a solution of the substrates in dry acetonitrile ([1a] = 0.1 M) containing 4CzIPN (2 mol%) as a photocatalyst and morpholine (AR-1, 1.5 equiv.) as an activation reagent was irradiated with a 456 nm LED at room temperature (∼25 °C) under argon for 12 h, desired addition product 3 was obtained in 92% yield (entry 1). Various other photocatalysts were tested, including Ir[dF(CF_3_)ppy]_2_(dtbbpy)PF_6_ (entry 2; see ESI† for additional details), but 4CzIPN was found to be optimal. Different activation reagents were also assessed: piperidine (AR-2) and *n*-butylamine (AR-3) gave lower yields of 3 (31% and 30%, respectively; entries 3 and 4), and all the other tested activation reagents gave yields of <20% (see ESI†).

**Table 1:** Optimization of conditions for addition reaction between *N*-phenylacrylamide (1a) with cyclohexylboronic acid 2a^a^

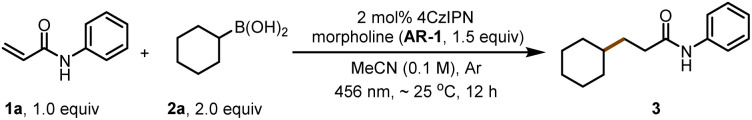		
Entry	Deviation from standard conditions	Yield^b^ (%)
1	None	92 (90^c^)
2	Ir[dF(CF_3_)ppy]_2_(dtbbpy)PF_6_ as PC	65
3	Piperidine (AR-2) as activation reagent	31
4	*n*-Butylamine (AR-3) as activation reagent	30
5	Dichloromethane as solvent	85
6	Toluene as solvent	87
7^d^	2b instead of 2a	97(93^c^)
8^e^	2c instead of 2a	85(82^c^)
9	No activation reagent	NR
10	No PC/light	NR
11	Under air	44
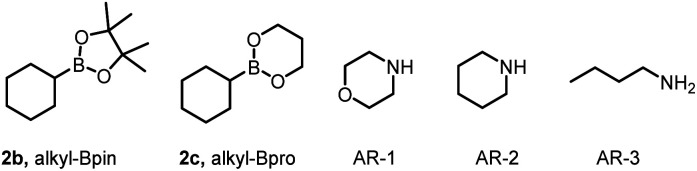		

a Standard conditions: 1a (0.2 mmol), 2a (0.4 mmol), photocatalyst (PC, 0.004 mmol), activation reagent (0.3 mmol), MeCN (2 mL), Ar, 456 nm LED, rt, 12 h.

b Yields were determined by ^1^H NMR spectroscopy with dibromomethane as an internal standard. NR = no reaction.

c Isolated yield.

d 2b (0.4 mmol), toluene (2 mL).

e 2c (0.4 mmol), dichloromethane (2 mL).

Solvent screening revealed that dichloromethane and toluene gave slightly lower yields of 3 than acetonitrile (compare entry 1 with entries 5 and 6); the other tested solvents gave yields of <45% (see ESI†). Then we replaced cyclohexylboronic acid (2a) with cyclohexylboronic pinacol ester (2b, alkyl-Bpin) or 2-cyclohexyl-1,3,2-dioxaborinane (2c, alkyl-Bpro) as the radical precursor. Reaction of 2b in toluene gave the highest yield of 3a (97%, entry 7). However, when the radical precursor was 2c, the solvent had to be changed to dichloromethane (entry 8). Control experiments proved that the activation reagent, the photocatalyst, light, and exclusion of oxygen were essential for the transformation (entries 9–11).

**Fig. 2:**
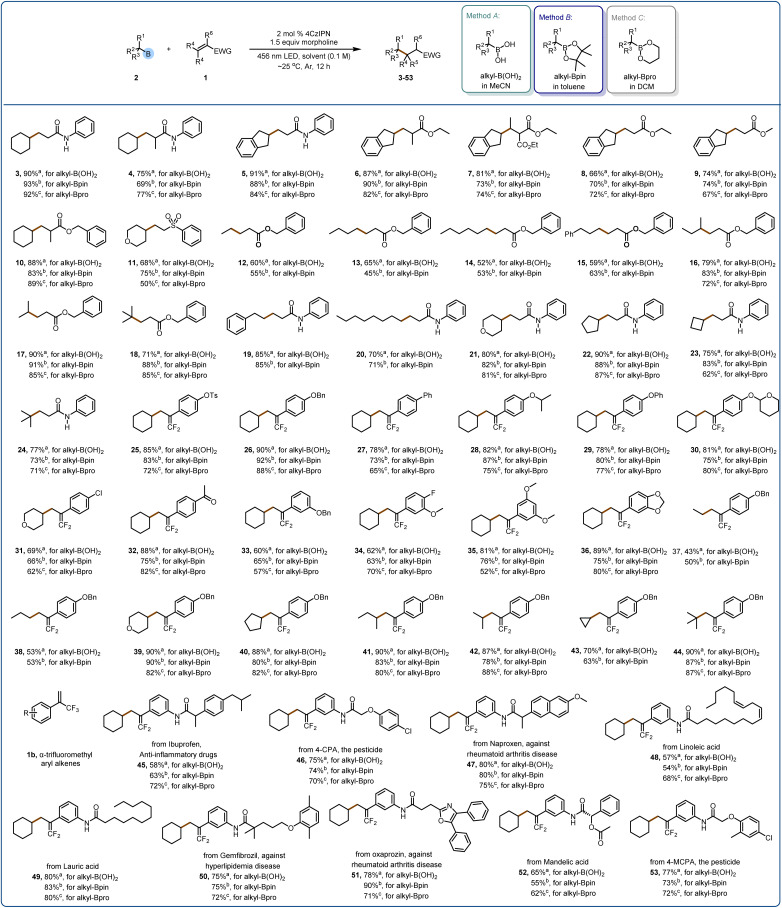
Substrate scope of the deboronative functionalization reaction. Reaction conditions: 1 (0.2 mmol), 2 (0.4 mmol), 4CzIPN (2 mol%), morpholine (0.3 mmol, 1.5 equiv.), 456 nm LED, solvent (2.0 mL, [1] = 0.1 M), ∼25 °C, Ar, 12 h. Isolated yields are reported. EWG = electron-withdrawing group. ^*a*^Method A: Alkyl-B(OH)_2_ (0.4 mmol, 2.0 equiv.), MeCN (2.0 mL). ^*b*^Method B: Alkyl-BPin (0.4 mmol, 2.0 equiv.), toluene (2.0 mL). ^*c*^Method C: Alkyl-BPro (0.4 mmol, 2.0 equiv.), dichloromethane (2.0 mL).

Next, we evaluated the substrate scope of the reaction (Fig. 2). We endeavored to assess substrates with functional groups that would be useful to the end user (*e.g.*, in a drug discovery setting) but that might be problematic under reaction conditions involving strong oxidizing or reducing intermediates, which are common in photoredox reactions. Specifically, we tested the method on various Michael acceptors 1 and several alkylboron species 2. The mild reaction conditions were found to be compatible with acceptors bearing ester, amide, or sulfone moieties, which offer handles for subsequent synthetic manipulations. Specifically, acrylamide-based acceptors were well tolerated, giving 3–5 in excellent yields, as were α-alkyl acrylates and unsubstituted acrylates (6–10). Moreover, phenyl vinyl sulfone furnished conjugate adduct 11 in moderate yield (68%). Next, we studied the scope of the reaction with respect to the alkylboron species by using benzyl acrylate as the Michael acceptor. We found that a wide range of primary alkylboron species gave the desired products in moderate to good yields. For example, linear alkylboron species afforded 12–14 in 52–65% yields. We were pleased to find that a phenylpropyl compound was amenable to the reaction conditions, giving a 59% yield of 15. Unactivated secondary and tertiary alkylboron species generate more-stable radicals than primary alkylboron species and therefore gave higher yields of the corresponding products (16–18). Alkylboron species reacted with phenylacrylamide as the acceptor, giving the target products (19–24) in moderate to excellent yields.

Because the incorporation of fluorinated groups into organic molecules often confers desirable pharmacological properties such as increased metabolic stability and improved lipophilicity and bioavailability, chemists are increasingly interested in developing methods to synthesize fluorinated compounds.^16^ Therefore, to further demonstrate the versatility of our method, we used it to accomplish intramolecular radical polarity cross-elimination reactions, that is, defluorinative alkylation reactions.

For this purpose, we chose α-trifluoromethyl aryl alkenes 1b as substrates and achieved defluorinative alkylation under our optimal reaction conditions. Specifically, we investigated the substrate scope by carrying out reactions of the α-trifluoromethyl aryl alkenes with alkylboron species (Fig. 2). Specifically, α-trifluoromethyl aryl alkenes with an electron-donating group at the para position of the phenyl ring gave the corresponding products (25–30) in moderate to good yields. However, the yields were relatively low for aryl alkenes bearing an electron-withdrawing group on the phenyl ring (31 and 32). In addition, a *meta*-chloro-substituted compound and several disubstituted compounds gave the corresponding products (33 and 34–36, respectively). Two primary alkylboron compounds were suitable substrates, giving 37 and 38. Secondary and tertiary alkylboron species gave the corresponding products (39–43 and 44, respectively) in yields that were higher than the yields obtained with primary alkylboronic acids. We also used our mild defluorinative alkylation method to functionalize some drug molecules, pesticides, and structurally complex natural products, obtaining the corresponding products (45–53) in good to excellent yields. Taken together, the results shown in Fig. 2 demonstrate the robustness of our method.

**Fig. 3:**
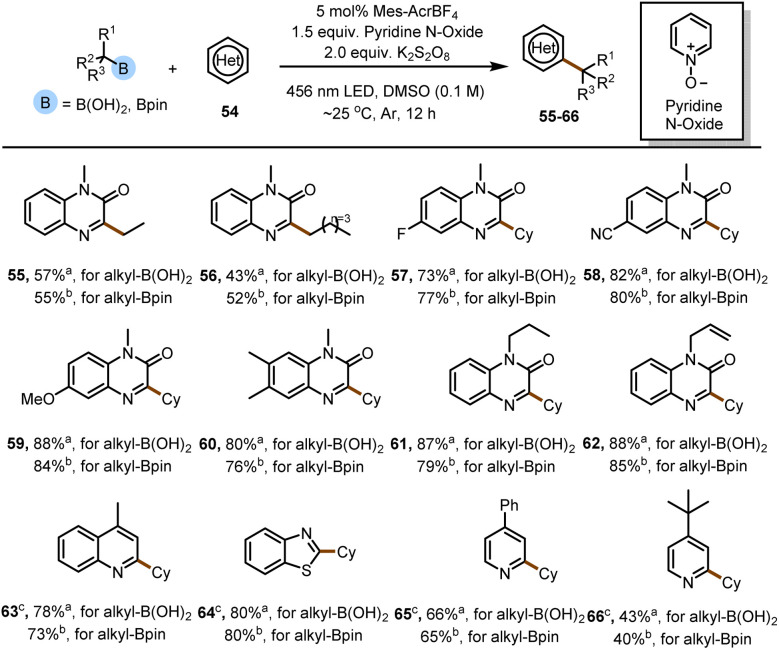
Substrate scope of the Minisci reaction. Reaction conditions: 54 (0.2 mmol), 2 (0.4 mmol), pyridine *N*-oxide (0.3 mmol, 1.5 equiv.), K_2_S_2_O_8_ (0.4 mmol), 456 nm LED, DMSO (2.0 mL, [54] = 0.1 M), ∼25 °C, Ar, 12 h. Isolated yields are reported. ^*a*^Alkyl-B(OH)_2_ (0.4 mmol, 2.0 equiv.). ^*b*^Alkyl-BPin (0.4 mmol, 2.0 equiv.). ^*c*^TFA (0.4 mmol, 2.0 equiv.).

Although this study focused on electron-deficient olefins as radical acceptors, we expanded the scope by using heteroarenes, which required the use of terminal oxidizing agents (Fig. 3). When we chose morpholine as the activating reagent, the yield of the reaction was poor, but when we replaced morpholine with pyridine *N*-oxide, the yield was better. In the presence of K_2_S_2_O_8_ as the terminal oxidant, various quinoxalin-2(1*H*)-ones were alkylated with alkylboron species to generate products 55–62 in good yields. Some other heterocycles (63–66) can also be compatible with this reaction, but additional trifluoroacetic acid is needed to activate the heterocycle.

**Fig. 4:**
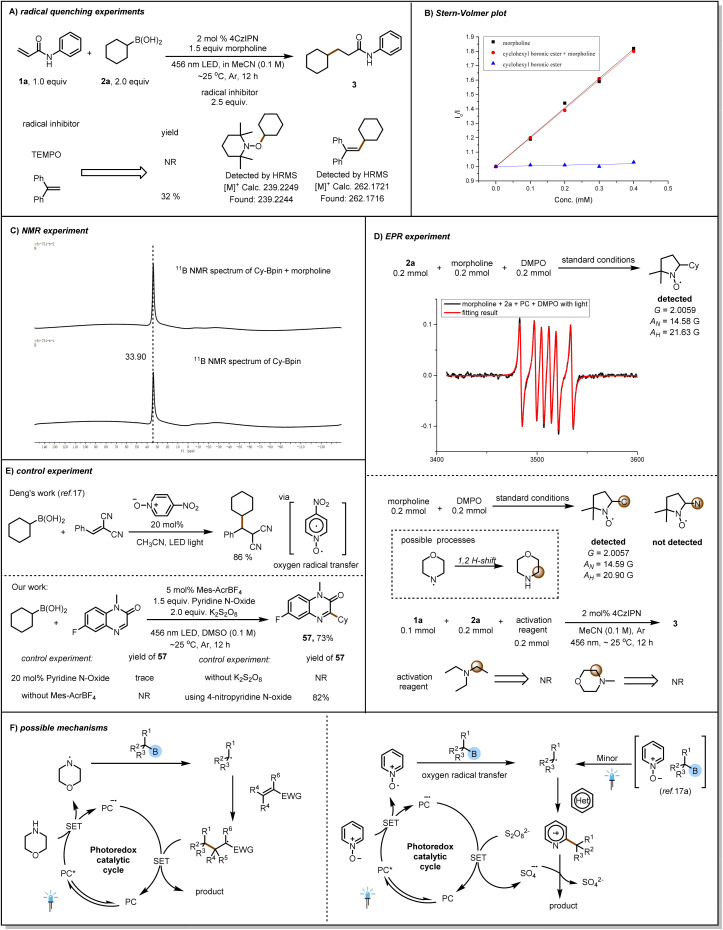
Mechanistic experiments. (A) Radical quenching experiments. (B) Stern–Volmer plot. (C) NMR experiment. (D) EPR experiment. (E) Control experiment. (F) Possible mechanisms.

We performed several experiments to gain insight into the reaction mechanism. We found that the reaction of 1a and 2a was inhibited by radical scavengers, and we detected radical-trapping products by means of high-resolution mass spectrometry (Fig. 4A). This indicates that the reaction produces alkyl radicals, which is a free radical process. Next, we carried out a light/dark experiment (see ESI†), which showed that the reaction of 1a and 2a stopped when there was no light. This result suggests that any chain propagation process was transient and that light was essential for product formation. Then Stern–Volmer plots showed that the excited-state photocatalyst was not quenched by cyclohexylboronic pinacol ester 2b and that the quenching rates observed with morpholine alone and with the combination of morpholine and 2b were the same (Fig. 4B).

This indicates that it seems that the same reagent (morpholine) quenches the excited state photocatalyst. In addition, no significant shift in the NMR signal of [^11^B] was observed when the alkylboronic pinacol ester was mixed with morpholine, a finding that suggests that the ester did not form a direct complex with morpholine (Fig. 4C). In order to better explain the reaction as a free radical process, we conducted EPR experiments (Fig. 4D) and successfully detected cyclohexyl radicals. However, when only morpholine and photocatalyst were present in the reaction system, we did not directly detect amine radicals, but instead detected alkyl carbon radicals. We speculate that this is because amine radical cannot exist stably in the reaction, resulting in 1,2 H-shift and being captured by DMPO. However, in order to further investigate the role of α-aminoalkyl radical, we replaced morpholine with substances such as triethylamine. We found that this reaction could not proceed, which means that it was not the α-aminoalkyl radical that induced the cleavage of the C–B bond. In 2023, Deng's research group^17*a*^ achieved the radical addition reaction of alkylboronic acid using catalytic amounts of pyridine *N*-oxide without the use of photocatalysts (Fig. 4E). This boron removal strategy was achieved through oxygen radical transfer. We found through controlled experiments that reducing the amount of pyridine *N*-oxide, not using photocatalysts, and not using oxidants cannot achieve the conversion of product 57.

These results indicate that the excited-state photocatalyst was quenched by morpholine and that the alkyl radical was produced by amine-radical-induced scission of the C–B bond. On the basis of the above-described results, we propose the reaction mechanism (Fig. 4F), briefly, the excited-state photocatalyst is quenched by morpholine to produce an aminyl radical, which subsequently reacts with the alkylboron species to release the corresponding alkyl radical, which then undergoes a Michael addition reaction with the acceptor compound.

## Conclusions

In conclusion, we have developed a photochemical method for generating alkyl radicals from readily available alkylboron compounds *via* homolytic substitution reactions of the boron group with amine or oxygen radicals. The method is applicable to a wide range of boron compounds, from which the corresponding alkyl radicals can be efficiently generated. The method can be used not only for reductive Michael addition reactions but also for Minisci reactions in the presence of an oxidizing agent.

## Data availability

The ESI† includes all experimental details, including optimization of the synthetic method, synthesis and characterization of all starting materials and products reported in this study, and mechanistic studies. NMR spectra of all products reported are included as well.

## Author contributions

F. Y. conceived the chemistry and designed the experiments under the guidance of Professor Q. W.; F. Y., M. L. and K. Y. conducted the experiments or analyzed the data. F. Y. wrote the manuscript. All authors have given approval to the final version of the manuscript.

## Conflicts of interest

There are no conflicts to declare.

## Supplementary Material

SC-OLF-D4SC02889A-s001
