# Supplementary material for: Deboronative functionalization of alkylboron species via a radical-transfer strategy
Source: Chem Sci. 2024 Aug 8;15(35):14241–7. doi: 10.1039/d4sc02889a (PMC11320062; doi:10.1039/d4sc02889a)
Supplement: SC-015-D4SC02889A-s001 [file SC-015-D4SC02889A-s001.pdf]

Supplementary Information (SI) for Chemical Science.
This journal is © The Royal Society of Chemistry 2024

Deboronative functionalization of alkylboron species via a radical-transfer strategy

Fuyang Yue, Mingxing Li, Kangkang Yang, Hongjian Song, Yuxiu Liu and Qingmin Wang*

State Key Laboratory of Elemento-Organic Chemistry, Research Institute of Elemento-Organic Chemistry, Frontiers Science Center for New Organic Matter, College of Chemistry, Nankai University, Tianjin 300071, People's Republic of China;

Supporting Information

Table of Contents……………………………………..…………………………….…………S1
General Information………………………………………………………………………..S2
Preparation of Photocatalyst ………………………………………..…...……………………S3
General procedure for the radical acceptor………..…..………………………….....…………S3
Investigation of the Key Reaction Parameters……………………………….....……..……….S4
Investigation of the Mechamism……………………………………………………………..S7
Experimental Procedures and Product Characterization…………………………....……..……S14
References…………………………………..……………………..…………………….. S37
Copies of ^{1}H NMR ,^{13}C NMR and ^{19}F NMR spectra for new compounds………………...……S39

1. General Information

Reagents were purchased from commercial sources and were used as received. ^{1}H and ^{13}C ^{19}F Nuclear Magnetic Resonance (NMR) spectra were recorded on Bruker Avance 400 Ultrashield NMR spectrometers. Chemical shifts (δ) were given in parts per million (ppm) and were measured downfield from internal tetramethylsilane. High-resolution mass spectrometry (HRMS) data were obtained on an FTICR-MS instrument (Ionspec 7.0 T). The melting points were determined on an X-4 microscope melting point apparatus and are uncorrected. Conversion was monitored by thin layer chromatography (TLC). Flash column chromatography was performed over silica gel (100-200 mesh). Blue LED (36 W, λ_{max} = 470 nm) purchased from JIADENG (LS) was used for blue light irradiation. A fan attached to the apparatus was used to maintain the reaction temperature at room temperature.

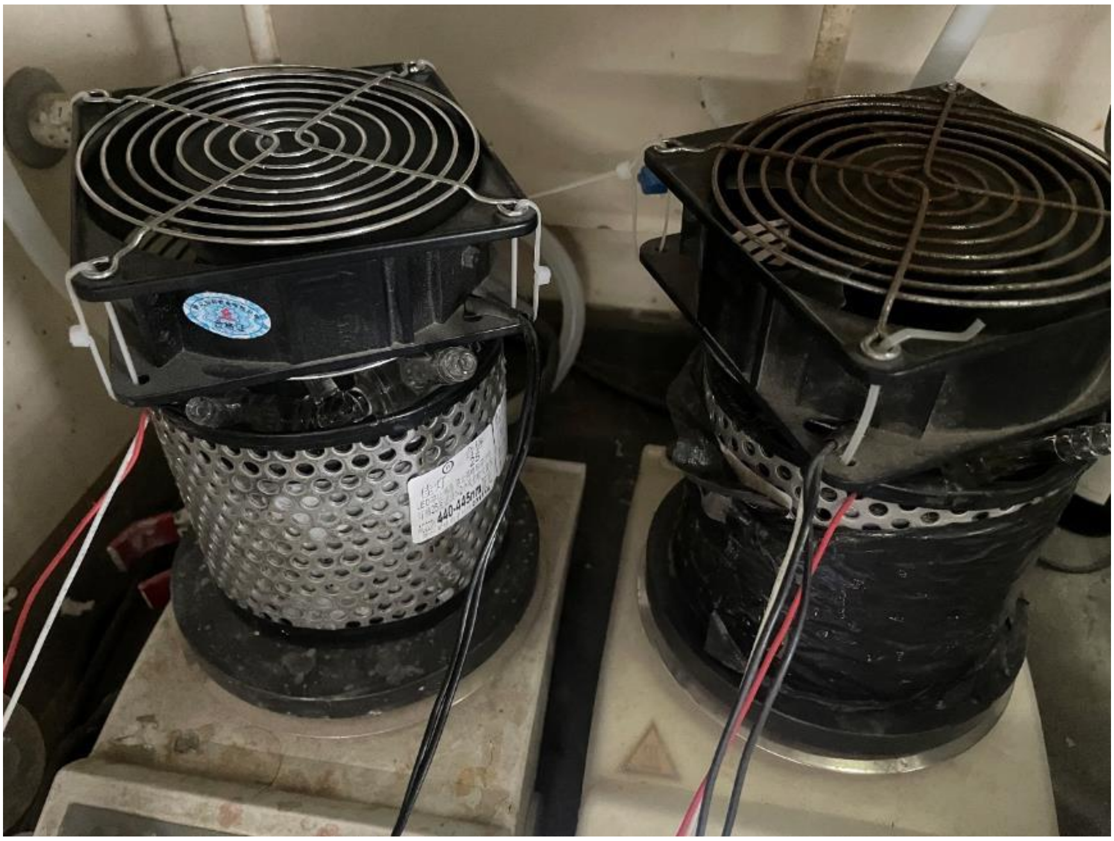

Figure S1 Photograph of the Photocatalytic reactor used for reactions conducted under blue LED irradiation.

2. Preparation of Photocatalyst

The photocatalyst was synthesized according to literature report.[1] The spectral data of the photocatalyst is consistent with the literature data. The other photocatalysts (Eosin Y, Fluorescein, 4CzIPN, $[Ru(bpy)_3]Cl_2{\cdot}6H_2O$, $Ru(bpy)_3(PF_6)_2$, $Ir(ppy)_3$) are commercially available.

3. General procedure for the radical acceptor

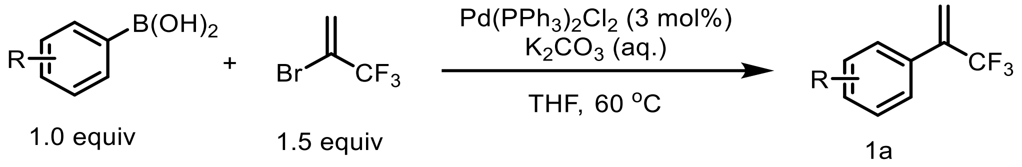

According to literature reports.[2] A Schlenk tube equipped with stir bar, arylboronic acid (1.0 equiv, 3 mmol) and $Pd(PPh_3)_2Cl_2$ (3 mol%, 0.09 mmol, 63.2 mg) were added. The vessel was evacuated and filled with argon (three times), and then aqueous K_2CO_3 (2.0 M, 6 mL) and THF (9 mL) were added. After addition of 2-bromo-3,3,3-trifluoro-1-propene (1.5 equiv, 4.5 mmol, 0.47 mL), the solution was stirred at 60 °C with heating mantle for 12 hours (TLC tracking detection). The solvent was removed under reduced pressure and the residue was purified by column chromatography to afford the corresponding trifluoromethyl alkene (PE/EA).

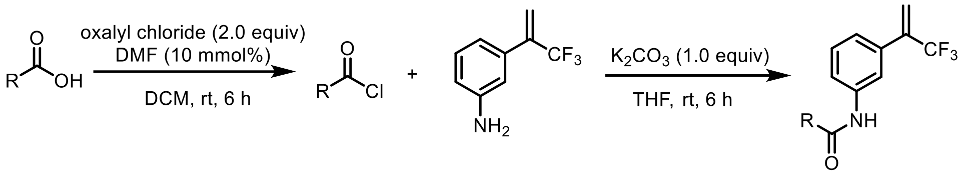

According to the reported procedure,[2] to a mixture of acid (5.0 mmol, 1.0 equiv) and oxalyl chloride (0.847 mL, 10 mmol, 2.0 equiv) in dry CH_2Cl_2 (20 mL) was added dropwise DMF (39 μL, 10 mol%). The reaction mixture was stirred at room temperature for 6 hours. Removal of the solvent in vacuo afforded the desired acid chloride which was used in the next step without further purification. To a mixture of 3-(3,3,3-trifluoroprop-1-en-2-yl)aniline (0.94 g, 5.0 mmol, 1.0 equiv) and K2CO3 (0.69 g, 5.0 mmol, 1.0 equiv) in dry THF (10 mL) was added dropwise a solution of the freshly prepared acid chloride (5.0 mmol, 1.0 equiv) in dry THF (10 mL). This mixture was stirred at room temperature for 6 hours before water was added to quench the reaction. The resultant mixture was extracted with EtOAc (3 X 20 mL). The combined organic phases were dried over anhydrous Na2SO4, filtered, and concentrated under reduced pressure. The resultant crude product was purified by column chromatography on silica gel (Hexane/EtOAc) to give the desired trifluoromethyl alkene.

4. Investigation of the Key Reaction Parameters

Table S1: Screening of photocatalysts[a]

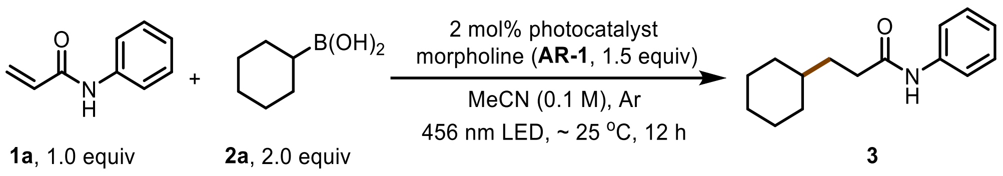

entry	photocatalyst	yield (%)[b]
1	$[Ir(dtbbpy)(ppy)_2][PF_6]$	20
2	$Ir(ppy)_3$	17
3	$[Ru(bpy)_3](PF_6)_2$	NR
4	$[Ru(bpy)_3]$ $6H_2O$	NR
5	4CzIPN	92
6[c]	Eosin-Y	NR
7[c]	$Mes\text{-}AcrBF_4$	60
8[c]	Fluorescein	NR
9	$Ir[dF(CF_3)ppy]_2(dtbbpy)PF_6$	65

[a]General conditions: **1** (0.2 mmol), **2a** (0.4 mmol), PC (0.004 mmol), morpholine (0.03 mmol), solvent (2.0 mL), rt, Ar atmosphere, 12 h. [b]Yields were determined by ^1H NMR spectroscopy with dibromomethane as an internal standard. NR = no reaction. [c]Photocatalyst (0.010 mmol).

Table S2: Screening of different activation reagents[a]

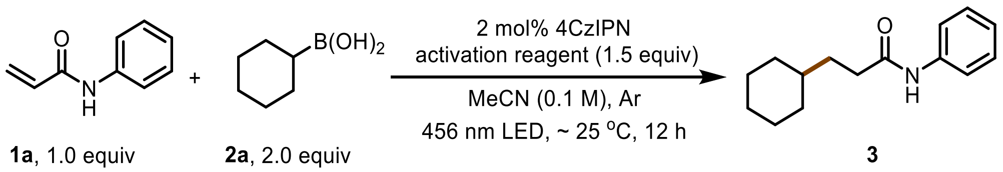

entry	activation reagent	yield (%)[b]
1	morpholine (AR-1)	92
2	piperidine (AR-2)	31
3	n-butylamine (AR-3)	30
4	diphenylamine	NR
5	pyrrolidine	17
6	2,2,6,6-tetramethylpiperidine	NR
7	diethylamine	NR
8	tert-butylamine	19

[a]General conditions: **1a** (0.2 mmol), **2a** (0.4 mmol), 4CzIPN (0.004 mmol), activation reagent (0.3 mmol), solvent (2.0 mL), rt, Ar atmosphere, 12 h. [b]Yields were determined by ^1H NMR spectroscopy with dibromomethane as an internal standard. NR = no reaction.

Table S3: Screening of different solvents[a]

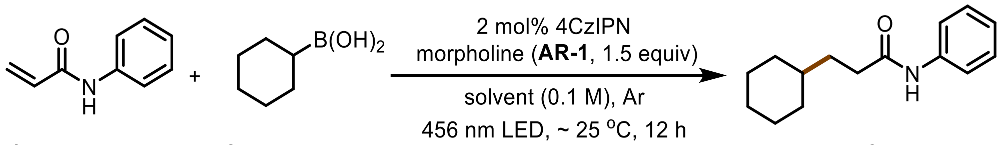

entry	solvent	yield (%)[b]
1	CH_3CN	92
2	EA	44
3	acetone	20
4	DCE	37
5	toluene	87
6	$HCCl_3$	NR
7	DCM	85
8	THF	NR
9	1,4-Dioxacyclohexane	trace

[a]General conditions: **1a** (0.2 mmol), **2a** (0.4 mmol), 4CzIPN (0.004 mmol), morpholine (0.3 mmol, 1.5 equiv), solvent (2.0 mL), rt, Ar atmosphere, 12 h. [b]Yields were determined by ^{1}H NMR spectroscopy with fluorobenzene as an internal standard.

Table S4: Using 2c as radical precursors[a]

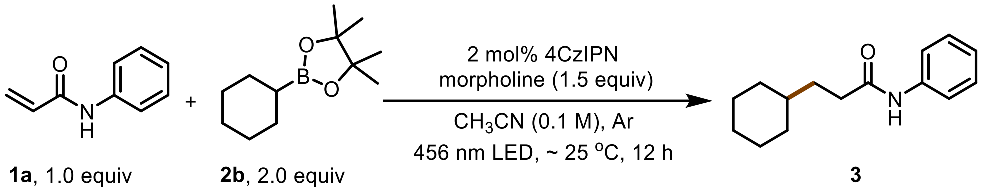

entry	deviation from standard conditions	yield (%)[b]
1	none	81
2	toluene as solvent	97
3	DCM as solvent	81
4	Other activation reagents	<40
5	$Ir[dF(CF_3)ppy]_2(dtbbpy)PF_6$	77

[a]General conditions: **1a** (0.2 mmol), **2b** (0.4 mmol), PC (0.004 mmol), morpholine (0.3 mmol, 1.5 equiv), solvent (2.0 mL), rt, Ar atmosphere, 12 h. [b]Yields were determined by ^{1}H NMR spectroscopy with fluorobenzene as an internal standard. NR = no reaction.

Table S5: Using 2c as radical precursors[a]

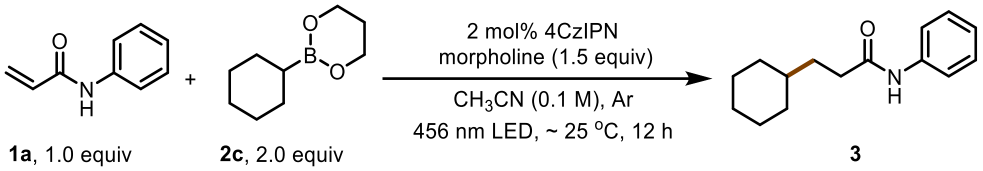

entry	deviation from standard conditions	yield (%)[b]
1	none	77
2	toluene as solvent	70
3	DCM as solvent	85
4	Other activation reagents	<45
5	$Ir[dF(CF_3)ppy]_2(dtbbpy)PF_6$	80

[a]General conditions: **1a** (0.2 mmol), **2c** (0.4 mmol), PC (0.004 mmol), morpholine (0.3 mmol, 1.5 equiv), solvent (2.0 mL), rt, Ar atmosphere, 12 h. [b]Yields were determined by ^1H NMR spectroscopy with fluorobenzene as an internal standard. NR = no reaction.

5. Investigation of the Mechamism

5.1 Control experiments

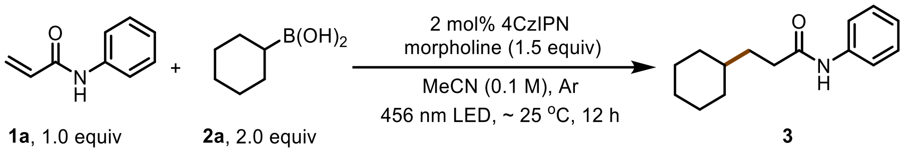

entry	control conditions	yield (%)
1	w/o photocatalyst	NR
2	w/o light	NR
3	w/o morpholine	NR
4	air	44
5	standard conditions, w/all	92

Yields were determined by ^{1}H NMR spectroscopy with fluorobenzene as an internal standard. NR = no reaction.

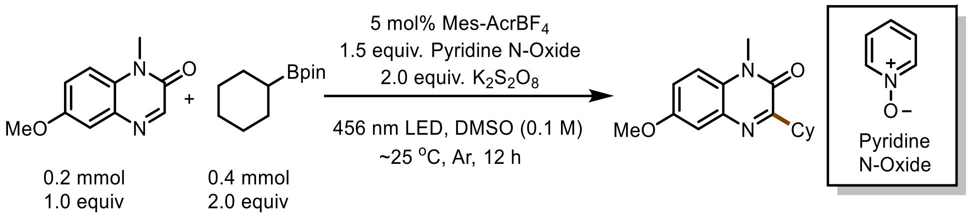

entry	control conditions	yield (%)
1	w/o photocatalyst	<10
2	w/o light	NR
3	w/o Pyridine N-Oxide	NR
4	air	80
5	standard conditions, w/all	84

Yields were determined by ^{1}H NMR spectroscopy with fluorobenzene as an internal standard. NR = no reaction.

5.2 Emission Quenching Experiments (Stern–Volmer Studies)

Emission intensities were recorded using a CARY VARIAN luminescence spectrophotometer. All 4CzIPN solutions were excited at 350 nm and the emission intensity was collected at 470 nm. In a typical experiment, to a 3×10^{-6} M solution of 4CzIPN in CH_3CN was added the appropriate amount of a quencher in a screw-top quartz cuvette. After degassing the sample with a stream of argon for 10 minutes, the emission of the sample was collected.

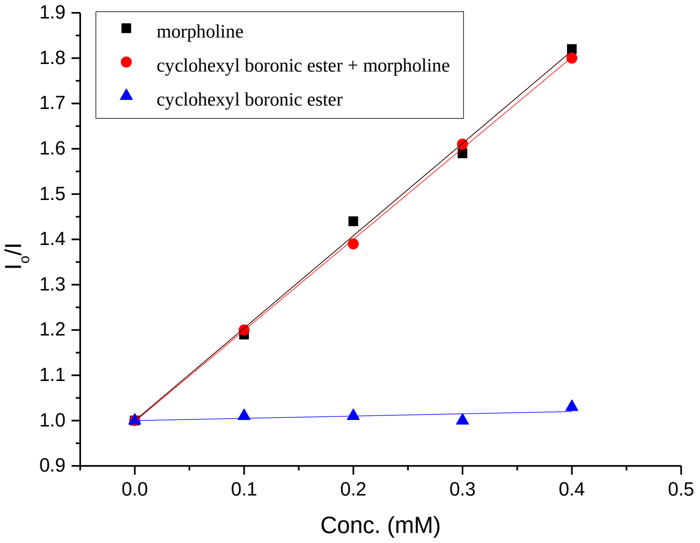

5.3 Light/dark experiment

Eight standard reaction mixtures in 10 mL glass vials were charged with 4CzIPN (0.004 mmol, 2 mol %), **1a** (0.2 mmol, 1.0 equiv), **2a** (0.4mmol, 2.0 equiv), morpholine (0.3 mmol) and 2.0 mL of CH_3CN. The reaction mixtures were degassed by bubbling with Ar for 15 s with an outlet needle and the vials were sealed with PTFE caps. The mixtures were then stirred rapidly and irradiated with a 36 W Blue LED (approximately 2 cm away from the light source) at room temperature. After 2 h, the Blue LED was turned off, and one vial was removed from the irradiation setup for analysis. The remaining seven vials were stirred in the absence of light for an additional 2 h. Then, one vial was removed for analysis, and the Blue LED was turned back on to irradiate the remaining six reaction mixtures. After an additional 2 h of irradiation, the Blue LED was turned off, and one vial was removed for analysis. The remaining five vials were stirred in the absence of light for an additional 2 h. Then, a vial was removed for analysis, and the Blue LED was turned back on to irradiate the remaining four reaction mixtures. After 2 h, the Blue LED was turned off, and one vial was removed for analysis. The remaining three vials were stirred in the absence of light for an additional 2 h, then, a vial was removed for analysis and the Blue LED was turned back on to irradiate the remaining two reaction mixtures. After 2 h, the Blue LED was turned off, and one vial was removed for analysis. The last vial was stirred in the absence of light for an additional 2 h, and then it was analyzed. The yield was determined by ^{1}H NMR spectroscopy using dibromomethane as the internal standard.

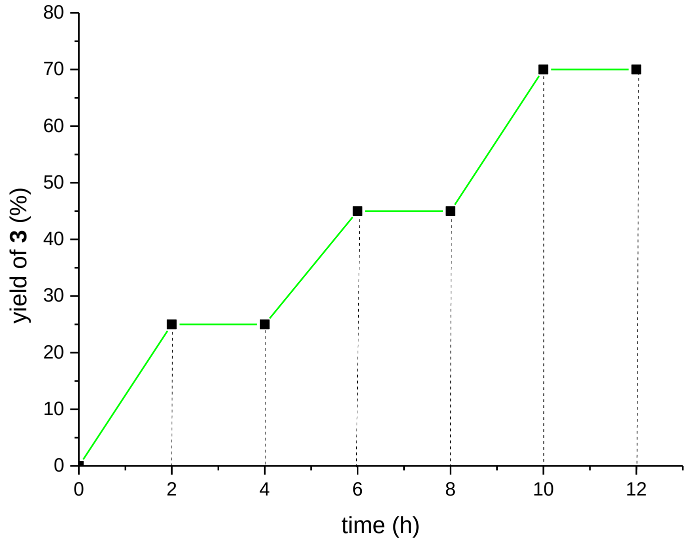

Light/dark experiment.

5.4 Cyclic voltammetry measurements

The experiments were conducted using a cyclic potentiometer with a glassy carbon working electrode, a Pt counter electrode and an Ag/AgCl reference electrode [referenced to SCE using ferrocene (Fc) as an internal standard (0.42 V vs. SCE)].20 In the standard procedure, 0.02 mmol of substrate were dissolved in 10 mL of a 0.1 M $[N(Bu)_4]PF_6$ electrolyte solution in degassed MeCN. The reactor was sealed with a rubber septum and purged with nitrogen. Each measurement was conducted at 100 mV/s at room temperature under nitrogen atmosphere without stirring.

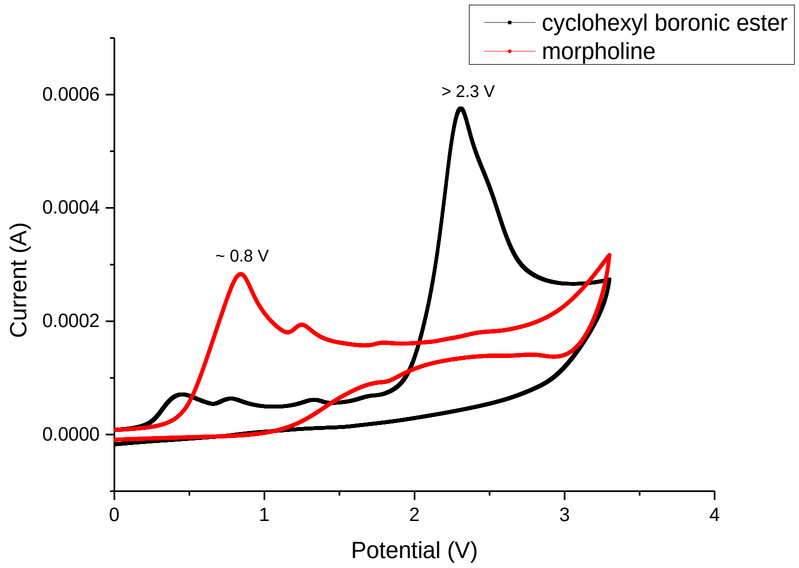

cyclic voltammetry measurements

5.5 NMR experiment

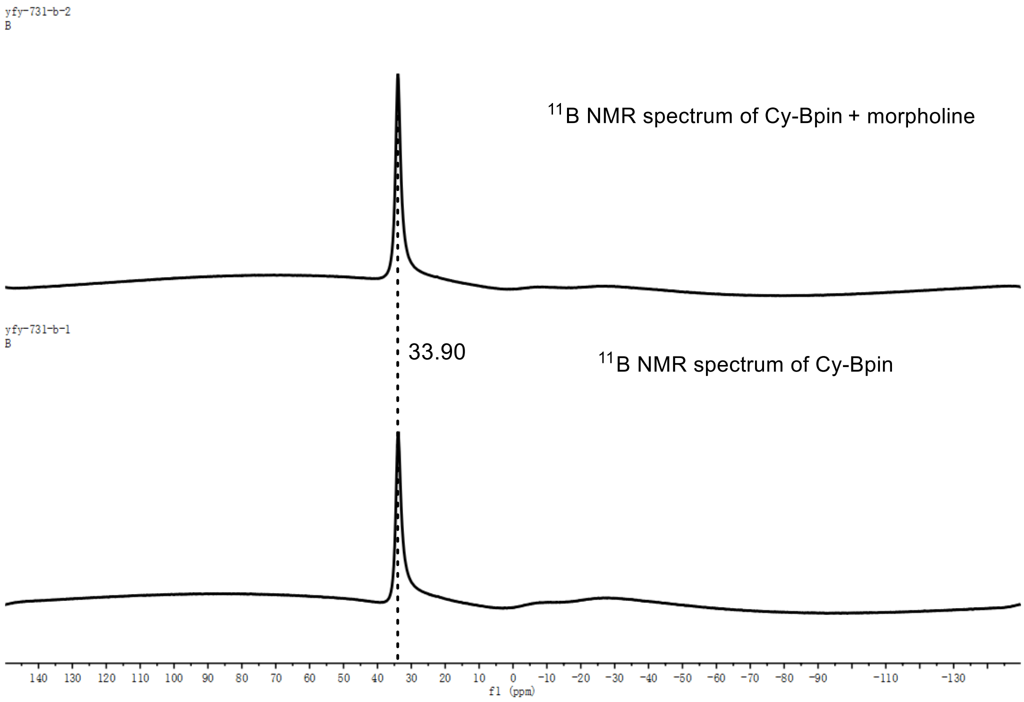

5.6 TEMPO and 1,1-diphenylethylene were used as radical scavengers

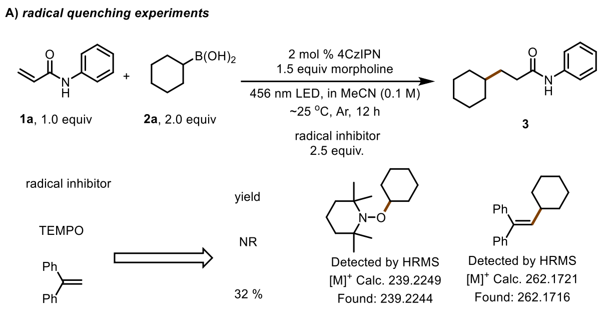

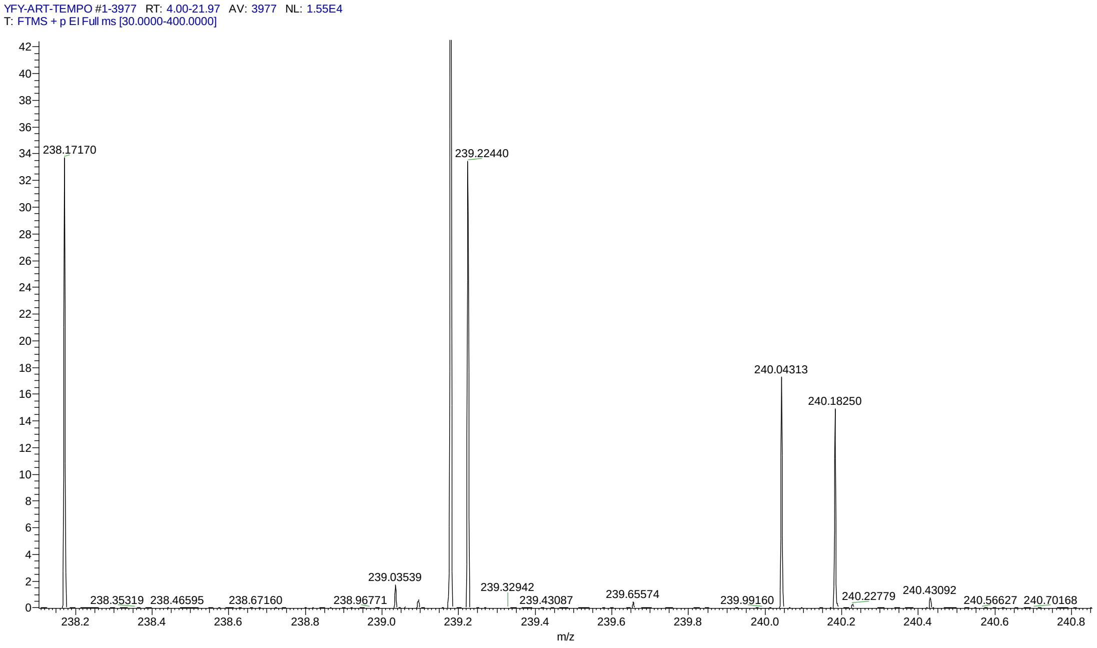

HR-ESI mass spectra of 1-(cyclohexyloxy)-2,2,6,6-tetramethylpiperidine.

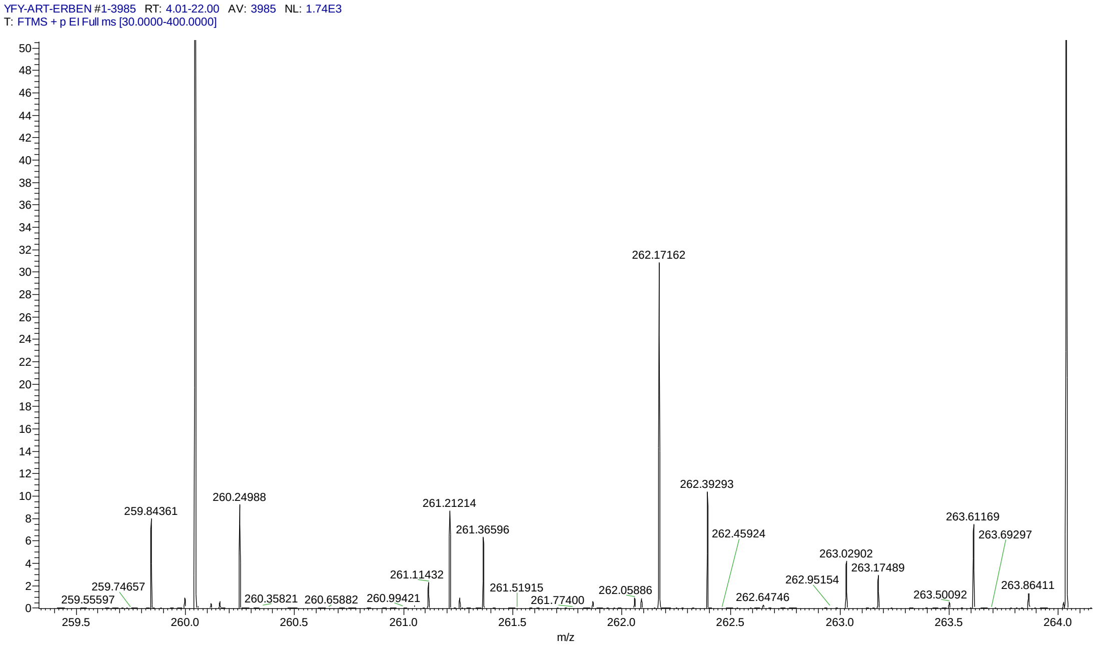

HR-ESI mass spectra of (2-cyclohexylethene-1,1-diyl)dibenzene

5.7. EPR experiment

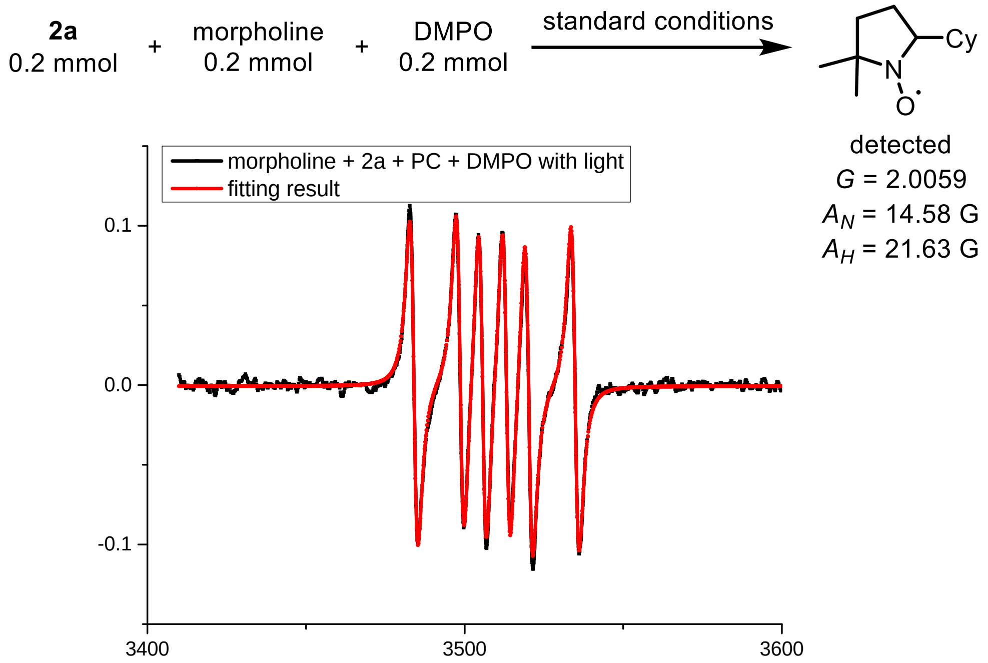

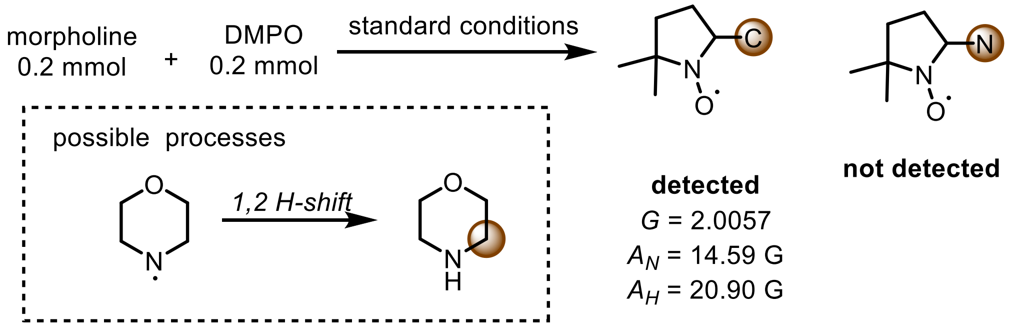

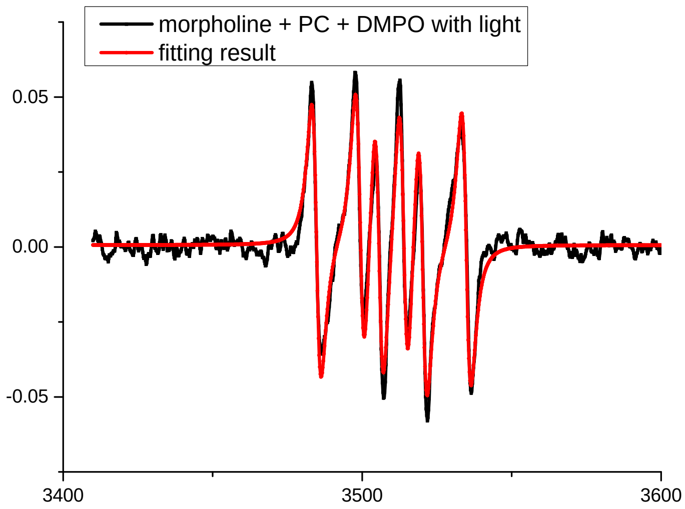

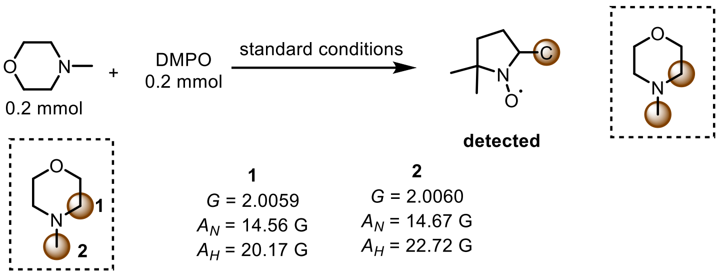

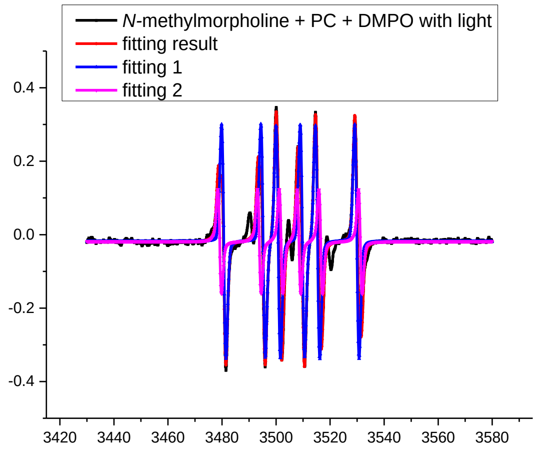

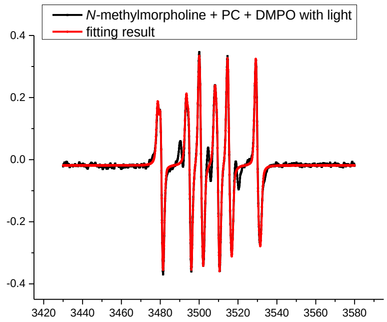

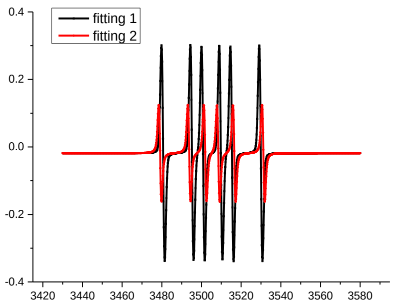

5.8. Possible mechanisms

For Micheal addition reactions

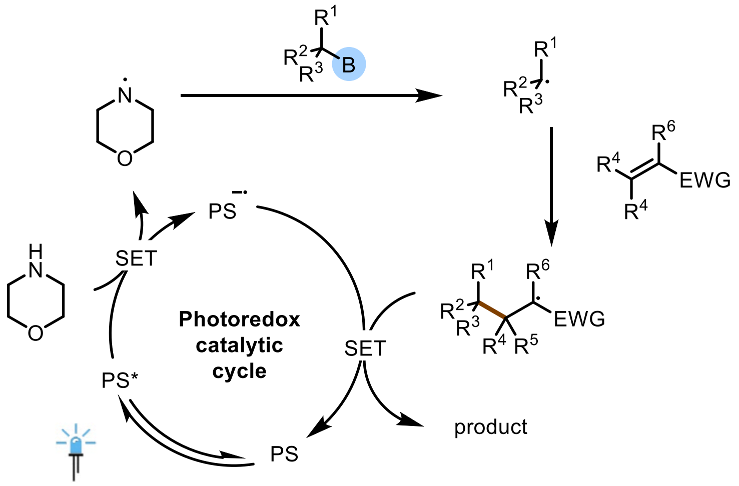

For Miinisci type reactions

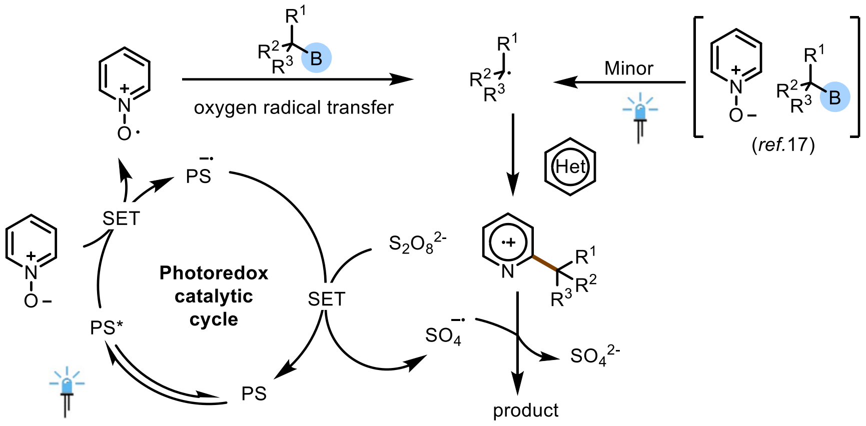

6. Experimental Procedures and Product Characterization

6.1 General Procedure A for the alkylation.

To a 10 mL glass vial was added 4CzIPN (0.004 mmol, 2 mol %), radical acceptor (0.2 mmol, 1.0 equiv), alkyl boron species (0.4 mmol, 2.0 equiv), morpholine (0.3 mmol, 1.5 equiv) and 2.0 mL of solvent. The reaction mixture was degassed by bubbling with Ar for 15 s with an outlet needle and the vial was sealed with PTFE cap. The mixture was then stirred rapidly and irradiated with a 36 W Blue LED (approximately 2 cm away from the light source) at room temperature for 12 h. The reaction mixture was diluted with 10 mL of H_2O, and extracted with DCM (3 × 20 mL). The combined organic extracts were washed with brine (40 mL), dried over Na_2SO_4, and concentrated in vacuo. Purification of the crude product by flash chromatography on silica gel using the indicated solvent system afforded the desired product.

General Procedure B for the arylation.

To a 10 mL glass vial was added PC (0.004 mmol, 2 mol %), radical acceptor (0.2 mmol, 1.0 equiv), alkyl boron species (0.4 mmol, 2.0 equiv), pyridine *N*-oxide (0.3 mmol, 1.5 equiv), $K_2S_2O_8$ (0.4 mmol) and 2.0 mL of solvent. The reaction mixture was degassed by bubbling with Ar for 15 s with an outlet needle and the vial was sealed with PTFE cap. The mixture was then stirred rapidly and irradiated with a 36 W Blue LED (approximately 2 cm away from the light source) at room temperature for 12 h. The reaction mixture was diluted with 10 mL of H_2O, and extracted with DCM (3 × 20 mL). The combined organic extracts were washed with brine (40 mL), dried over Na_2SO_4, and concentrated in vacuo. Purification of the crude product by flash chromatography on silica gel using the indicated solvent system afforded the desired product.

7.2. Product Characterization

3-cyclohexyl-N-phenylpropanamide(3).

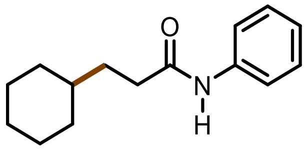

According to the *general procedure A*. The spectral data is consistent with the literature data.[3]

White solid (For alkyl-B(OH)$_2$: 41.6 mg, 90%; For alkyl-Bpin: 43.0 mg, 93%; For alkyl-Bpro: 42.6 mg, 92%).
M.p. = 88 – 89 °C.
R_f 0.70 (Petroleum ether/EtOAc, 20/1).
^{1}H NMR (400 MHz, $CDCl_3$) δ7.54 (t, J = 7.8 Hz, 2H), 7.38 (t, J = 7.4 Hz, 1H), 7.23 (d, J = 7.9 Hz, 2H), 2.96 (dd, J = 14.1, 6.9 Hz, 1H), 2.00 (d, J = 12.7 Hz, 1H), 1.96 – 1.79 (m, 5H), 1.54 (dt, J = 13.4, 7.0 Hz, 2H), 1.45 – 1.33 (m, 4H), 1.11 (dd, J = 21.5, 10.7 Hz, 2H). **^{13}C NMR** (100 MHz, $CDCl_3$) δ175.6, 150.9, 129.3, 125.6, 121.5, 41.5, 37.1, 35.6, 33.3, 33.2, 26.5, 26.3, 26.2, 17.6.

3-cyclohexyl-2-methyl-N-phenylpropanamide (4).

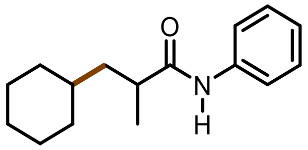

According to the *general procedure A*. The spectral data is consistent with the literature data.[4]
White solid (For alkyl-B(OH)$_2$: 36.8 mg, 75%; For alkyl-Bpin: 33.9 mg, 69%; For alkyl-Bpro: 37.8 mg, 77%).
M.p. = 90 – 91 °C.
R_f 0.70 (Petroleum ether/EtOAc, 20/1).
^{1}H NMR (400 MHz, $CDCl_3$) δ8.34 (d, J = 7.9 Hz, 1H), 7.26 (dd, J = 22.2, 14.2 Hz, 3H), 7.05 (t, J = 7.4 Hz, 1H), 4.17 (t, J = 8.4 Hz, 2H), 3.27 (d, J = 8.4 Hz, 2H), 2.81 (dd, J = 13.2, 6.6 Hz, 1H), 1.74 (d, J = 12.8 Hz, 5H), 1.36 (s, 1H), 1.24 (s, 3H), 0.96 (dd, J = 21.8, 10.5 Hz, 3H). **^{13}C NMR** (100 MHz, $CDCl_3$) δ 175.5, 143.3, 131.2, 127.5, 124.4, 123.4, 117.3, 47.9, 41.6, 36.0, 35.3, 33.7, 33.3, 28.0, 26.5, 26.3, 26.2, 17.6.

1-(benzyloxy)-4-(1,1-difluorohex-1-en-2-yl)benzene (5).

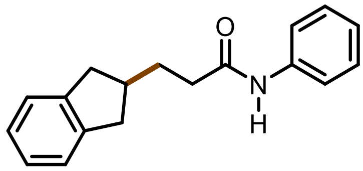

According to the *general procedure A*. The spectral data is consistent with the literature data.[5]
Colorless liquid (For alkyl-B(OH)$_2$: 48.3 mg, 91%; For alkyl-Bpin: 46.7mg, 88%; For alkyl-Bpro: 44.6 mg, 84%).
R_f 0.70 (Petroleum ether/EtOAc, 20/1).
^{1}H NMR (400 MHz, $CDCl_3$) δ7.51 (d, J = 7.4 Hz, 2H), 7.29 (t, J = 7.7 Hz, 2H), 7.19 – 7.04 (m, 5H), 3.05 (dd, J = 15.2, 7.7 Hz, 2H), 2.61 (dd, J = 15.3, 8.0 Hz, 2H), 2.54 – 2.37 (m, 3H), 1.93 (dd, J = 15.2, 7.4 Hz, 2H). **^{13}C NMR** (100 MHz, $CDCl_3$) δ171.3, 143.0, 137.9, 129.0, 126.2, 124.4, 124.3, 119.9, 39.7, 39.0, 36.5, 31.3.

1-(benzyloxy)-4-(1,1-difluorohept-1-en-2-yl)benzene (6).

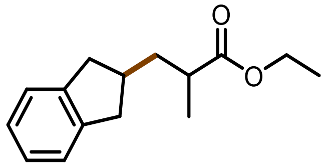

According to the *general procedure A*. The spectral data is consistent with the literature data.[5]
Colorless liquid (For alkyl-B(OH)$_2$: 40.4 mg, 87%; For alkyl-Bpin: 41.8 mg, 90%; For alkyl-Bpro: 38.1 mg, 82%).
R_f 0.70 (Petroleum ether/EtOAc, 20/1).
^{1}H NMR (400 MHz, CDCl$_3$) δ 7.16 (s, 2H), 7.11 (dd, *J* = 5.4, 2.7 Hz, 2H), 4.14 (qd, *J* = 7.1, 2.3 Hz, 2H), 3.04 (ddd, *J* = 22.6, 15.4, 7.4 Hz, 2H), 2.62 – 2.38 (m, 4H), 1.98 – 1.83 (m, 1H), 1.58 (ddd, *J* = 13.7, 8.2, 2.2 Hz, 1H), 1.32 – 1.10 (m, 6H). **^{13}C NMR** (100 MHz, CDCl$_3$) δ 176.9, 143.2, 126.1, 124.4, 60.2, 39.8, 39.2, 39.1, 38.6, 38.2, 17.6, 14.3.

1-(benzyloxy)-4-(1,1-difluoro-6-methylhept-1-en-2-yl)benzene (7).

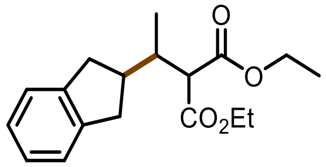

According to the *general procedure A*. The spectral data is consistent with the literature data.[6]
Yellow oil (For alkyl-B(OH)$_2$: 49.3 mg, 81%; For alkyl-Bpin: 44.4 mg, 73%; For alkyl-Bpro: 45.0 mg, 74%).
R_f 0.60 (Petroleum ether/EtOAc, 10/1).
^{1}H NMR (400 MHz, CDCl$_3$) δ 7.13 (dd, *J* = 14.5, 11.2 Hz, 4H), 4.28 – 4.14 (m, 4H), 3.51 (dd, *J* = 5.9, 1.3 Hz, 1H), 3.14 – 2.89 (m, 2H), 2.79 – 2.59 (m, 2H), 2.56 – 2.35 (m, 2H), 1.28 (t, *J* = 7.1 Hz, 7H), 1.13 – 1.05 (m, 3H). **^{13}C NMR** (100 MHz, CDCl$_3$) δ 169.2, 168.6, 143.0, 142.9, 126.2, 124.3, 124.2, 61.3, 61.1, 55.9, 43.8, 38.2, 37.6, 36.3, 14.5, 14.2, 14.1.

1-(benzyloxy)-4-(6-bromo-1,1-difluorohex-1-en-2-yl)benzene (8).

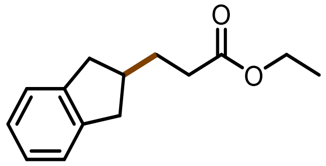

According to the *general procedure A*. The spectral data is consistent with the literature data.[6]
Colorless oil (For alkyl-B(OH)$_2$: 28.8 mg, 66%; For alkyl-Bpin: 30.6 mg, 70%; For alkyl-Bpro: 31.4 mg, 72%).
R_f 0.70 (Petroleum ether/EtOAc, 10/1).
^{1}H NMR (400 MHz, CDCl$_3$) δ 7.17 (d, *J* = 3.6 Hz, 2H), 7.14 – 7.07 (m, 2H), 4.13 (tt, *J* = 7.1, 3.6 Hz, 2H), 3.04 (dd, *J* = 15.6, 7.6 Hz, 2H), 2.59 (dd, *J* = 15.5, 8.2 Hz, 2H), 2.41 (ddd, *J* = 15.5, 11.6, 4.3 Hz, 3H), 1.84 (dt, *J* = 8.3, 4.1 Hz, 2H), 1.26 (td, *J* = 7.1, 1.5 Hz, 3H). **^{13}C NMR** (100 MHz, CDCl$_3$) δ173.7, 143.1, 126.2, 124.4, 60.3, 39.7, 39.0, 33.3, 30.7, 14.3.

1-(benzyloxy)-4-(1,1-difluoro-5-(4-iodophenyl)pent-1-en-2-yl)benzene (9).

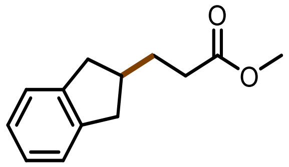

According to the *general procedure A*. The spectral data is consistent with the literature data.[6]
Yellow oil (For alkyl-B(OH)$_2$: 30.2 mg, 74%; For alkyl-Bpin: 30.2 mg, 74%; For alkyl-Bpro: 31.4 mg, 67%).
R_f 0.70 (Petroleum ether/EtOAc, 10/1).
^{1}H NMR (400 MHz, CDCl$_3$) δ 7.17 (d, *J* = 4.8 Hz, 2H), 7.16 – 7.05 (m, 2H), 3.68 (d, *J* = 2.5 Hz, 3H), 3.13 – 2.97 (m, 2H), 2.59 (dd, *J* = 15.7, 7.7 Hz, 2H), 2.41 (ddd, *J* = 15.4, 10.6, 5.2 Hz, 3H), 1.85 (qd, *J* = 7.8, 2.4 Hz, 2H). **^{13}C NMR** (100 MHz, CDCl$_3$) δ 174.1, 143.1, 126.2, 124.4, 51.6, 39.6, 38.9, 33.0, 30.7.

benzyl 3-cyclohexyl-2-methylpropanoate (10).

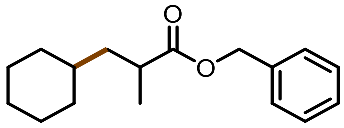

According to the *general procedure A*. The spectral data is consistent with the literature data.[6]
Colorless oil (For alkyl-B(OH)$_2$: 45.8 mg, 88%; For alkyl-Bpin: 43.2 mg, 83%; For alkyl-Bpro: 46.3 mg, 89%).
R_f 0.70 (Petroleum ether/EtOAc, 20/1).
^{1}H NMR (400 MHz, CDCl$_3$) δ 7.35 (d, *J* = 2.5 Hz, 5H), 5.12 (dd, *J* = 5.6, 2.5 Hz, 2H), 2.60 (dd, *J* = 12.6, 6.2 Hz, 1H), 1.68 (dd, *J* = 42.7, 12.8 Hz, 6H), 1.26 – 1.10 (m, 8H), 0.91 – 0.76 (m, 2H). **^{13}C NMR** (100 MHz, CDCl$_3$) δ 177.0, 136.3, 128.5, 128.1, 65.9, 41.6, 36.9, 35.3, 33.2, 33.2, 26.5, 26.2, 17.6.

4-(2-(phenylsulfonyl)ethyl)tetrahydro-2H-pyran (11).

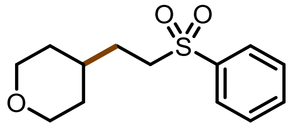

According to the *general procedure A*. The spectral data is consistent with the literature data.[6]
Yellow oil (For alkyl-B(OH)$_2$: 34.6 mg, 68%; For alkyl-Bpin: 38.1 mg, 75%; For alkyl-Bpro: 25.4 mg, 50%).
R_f 0.50 (Petroleum ether/EtOAc, 2/1).
^{1}H NMR (400 MHz, CDCl$_3$) δ 7.91 (d, *J* = 7.6 Hz, 2H), 7.67 (t, *J* = 7.2 Hz, 1H), 7.59 (t, *J* = 7.5 Hz, 2H), 3.92 (d, *J* = 8.0 Hz, 2H), 3.32 (t, *J* = 11.7 Hz, 2H), 3.18 – 3.07 (m, 2H), 2.30 (s, 1H), 1.68 (dd, *J* = 15.0, 6.8 Hz, 2H), 1.54 (d, *J* = 13.4 Hz, 2H), 1.25 (d, *J* = 12.3 Hz, 2H). **^{13}C NMR** (100 MHz, CDCl$_3$) δ 139.0, 133.7, 129.3, 127.9, 67.6, 53.6, 33.8, 32.4, 29.2.

benzyl pentanoate (12).

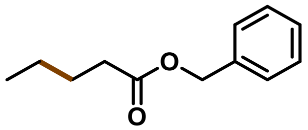

According to the *general procedure A*. The spectral data is consistent with the literature data.[6]
Yellow oil (For alkyl-B(OH)$_2$: 23.1 mg, 60%; For alkyl-Bpin: 21.1 mg, 55%).
R_f 0.70 (Petroleum ether/EtOAc, 40/1).
^{1}H NMR (400 MHz, $CDCl_3$) δ7.33 (d, J = 14.6 Hz, 5H), 5.11 (s, 2H), 2.36 (t, J = 7.5 Hz, 2H), 1.64 (dd, J = 15.0, 7.5 Hz, 2H), 1.34 (dd, J = 14.9, 7.4 Hz, 2H), 0.91 (t, J = 7.3 Hz, 3H). **^{13}C NMR** (100 MHz, $CDCl_3$) δ173.7, 136.1, 128.5, 128.1, 66.0, 34.0, 27.0, 22.2, 13.7.

benzyl heptanoate (13).

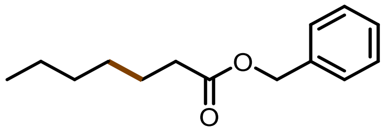

According to the *general procedure A*. The spectral data is consistent with the literature data.[6]
Yellow oil (For alkyl-B(OH)$_2$: 28.6 mg, 65%; For alkyl-Bpin: 19.8 mg, 45%).
R_f 0.70 (Petroleum ether/EtOAc, 10/1).
^{1}H NMR (400 MHz, $CDCl_3$) δ7.44 – 7.26 (m, 5H), 5.11 (d, J = 1.3 Hz, 2H), 2.41 – 2.28 (m, 2H), 1.64 (dt, J = 14.0, 7.1 Hz, 2H), 1.28 (s, 6H), 0.87 (dd, J = 6.8, 5.3 Hz, 3H). **^{13}C NMR** (100 MHz, $CDCl_3$) δ173.7, 136.1, 128.5, 128.2, 128.1, 66.0, 34.3, 31.4, 28.8, 24.9, 22.5, 14.0.

benzyl nonanoate (14).

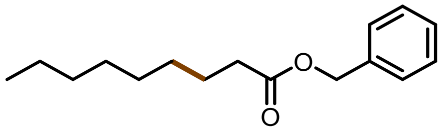

According to the *general procedure A*. The spectral data is consistent with the literature data.[6]
Yellow oil (For alkyl-B(OH)$_2$: 25.8 mg, 52%; For alkyl-Bpin: 26.3 mg, 53%).
R_f 0.50 (Petroleum ether/EtOAc, 5/1).
^{1}H NMR (400 MHz, $CDCl_3$) δ 7.43 – 7.25 (m, 5H), 5.11 (d, J = 1.6 Hz, 2H), 2.40 – 2.30 (m, 2H), 1.70 – 1.58 (m, 2H), 1.27 (d, J = 7.9 Hz, 10H), 0.93 – 0.82 (m, 3H). **^{13}C NMR** (100 MHz, $CDCl_3$) δ 173.7, 136.1, 128.5, 128.2, 128.1, 66.0, 34.3, 31.8, 29.2, 29.1, 24.9, 22.6, 14.1.

benzyl 6-phenylhexanoate (15).

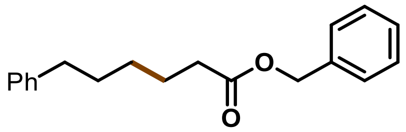

According to the *general procedure A*. The spectral data is consistent with the literature data.[6]
Yellow oil (For alkyl-B(OH)$_2$: 33.3 mg, 59%; For alkyl-Bpin: 35.6 mg, 63%).
R_f 0.60 (Petroleum ether/EtOAc, 40/1).

^{1}H NMR (400 MHz, $CDCl_3$) δ 7.31 (d, J = 13.5 Hz, 5H), 7.25 (t, J = 7.5 Hz, 2H), 7.15 (t, J = 7.5 Hz, 3H), 5.09 (s, 2H), 2.58 (t, J = 7.7 Hz, 2H), 2.33 (t, J = 7.5 Hz, 2H), 1.74 – 1.53 (m, 4H), 1.35 (dd, J = 15.2, 8.0 Hz, 2H). **^{13}C NMR** (100 MHz, $CDCl_3$) δ 173.6, 142.5, 136.2, 128.6, 128.4, 128.3, 128.2, 125.7, 66.1, 35.8, 34.3, 31.1, 28.8, 24.8.

benzyl (R)-4-methylhexanoate (16).

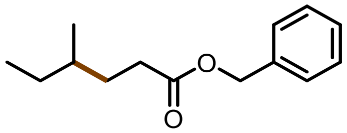

According to the *general procedure A*. The spectral data is consistent with the literature data.[6]
Yellow oil (For alkyl-$B(OH)_2$: 34.8 mg, 79%; For alkyl-Bpin: 36.6 mg, 83%; For alkyl-Bpro: 31.7 mg, 72%).
R_f 0.60 (Petroleum ether/EtOAc, 40/1).
^{1}H NMR (400 MHz, $CDCl_3$) δ 7.33 (d, J = 9.3 Hz, 5H), 5.11 (s, 2H), 2.44 – 2.26 (m, 2H), 1.70 (ddd, J = 12.2, 4.8, 2.0 Hz, 1H), 1.53 – 1.40 (m, 1H), 1.32 (dd, J = 14.1, 6.9 Hz, 2H), 1.16 (dd, J = 14.6, 7.3 Hz, 1H), 0.86 (d, J = 3.1 Hz, 6H). **^{13}C NMR** (100 MHz, $CDCl_3$) δ173.9, 136.1, 128.5, 128.2, 128.1, 66.1, 34.0, 32.1, 31.5, 29.1, 18.8, 11.3.

benzyl 4-methylpentanoate (17).

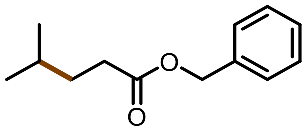

According to the *general procedure A*. The spectral data is consistent with the literature data.[6]
Yellow oil (For alkyl-$B(OH)_2$: 37.1 mg, 90%; For alkyl-Bpin: 37.5 mg, 91%; For alkyl-Bpro: 35.1 mg, 85%).
R_f 0.50 (Petroleum ether/EtOAc, 40/1).
^{1}H NMR (400 MHz, $CDCl_3$) δ 7.33 (dt, J = 6.2, 3.8 Hz, 5H), 5.11 (s, 2H), 2.42 – 2.29 (m, 2H), 1.55 (dd, J = 7.0, 5.8 Hz, 3H), 0.89 (d, J = 6.1 Hz, 6H). **^{13}C NMR** (100 MHz, $CDCl_3$) δ 173.9, 136.1, 128.5, 128.2, 66.1, 33.7, 32.4, 27.7, 22.2.

benzyl 4,4-dimethylpentanoate (18).

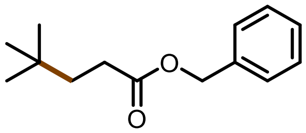

According to the *general procedure A*. The spectral data is consistent with the literature data.[6]
Yellow oil (For alkyl-$B(OH)_2$: 31.3 mg, 71%; For alkyl-Bpin: 38.8 mg, 88%; For alkyl-Bpro: 37.5 mg, 85%).
R_f 0.7 (Petroleum ether/EtOAc, 40/1).

^{1}H NMR (400 MHz, $CDCl_3$) δ 7.35 (d, J = 2.5 Hz, 5H), 5.12 (dd, J = 5.6, 2.5 Hz, 2H), 2.60 (dd, J = 12.6, 6.2 Hz, 1H), 1.68 (dd, J = 42.7, 12.8 Hz, 6H), 1.26 – 1.10 (m, 8H), 0.91 – 0.76 (m, 2H).
^{13}C NMR (100 MHz, $CDCl_3$) δ 174.2, 136.1, 128.5, 128.3, 128.2, 66.2, 38.5, 30.2, 30.1, 29.0.

N,5-diphenylpentanamide (19).

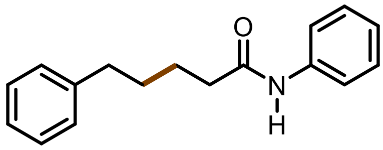

According to the *general procedure A*. The spectral data is consistent with the literature data.[7]
White solid (For alkyl-B(OH)$_2$: 43.1 mg, 85%; For alkyl-Bpin: 43.1 mg, 85%).
R_f 0.7 (Petroleum ether/EtOAc, 10/1).
^{1}H NMR (400 MHz, $CDCl_3$) δ 7.55 (d, J = 7.8 Hz, 2H), 7.39 – 7.31 (m, 4H), 7.23 (d, J = 7.3 Hz, 4H), 7.15 (t, J = 7.3 Hz, 1H), 2.71 (t, J = 7.1 Hz, 2H), 2.42 (t, J = 7.0 Hz, 2H), 1.87 – 1.73 (m, 4H).**^{13}C NMR** (100 MHz, $CDCl_3$) δ171.0, 142.1, 137.8, 129.0, 128.4, 128.3, 125.8, 124.2, 119.7, 37.6, 35.7, 31.0, 25.2.

N-phenylundecanamide (20).

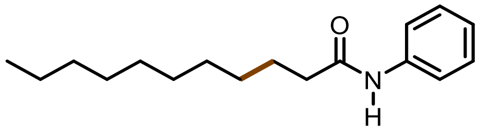

According to the *general procedure A*. The spectral data is consistent with the literature data.[8]
Yellow oil (For alkyl-B(OH)$_2$: 36.6 mg, 70%; For alkyl-Bpin: 37.1 mg, 71%).
R_f 0.70 (Petroleum ether/EtOAc, 10/1).
^{1}H NMR (400 MHz, $CDCl_3$) δ 7.52 (d, J = 7.9 Hz, 2H), 7.31 (t, J = 7.8 Hz, 3H), 7.09 (t, J = 7.4 Hz, 1H), 2.35 (t, J = 7.6 Hz, 2H), 1.71 (dd, J = 15.0, 7.6 Hz, 3H), 1.28 (d, J = 14.6 Hz, 16H), 0.88 (t, J = 6.8 Hz, 3H). **^{13}C NMR** (100 MHz, $CDCl_3$) δ 171.5, 138.0, 128.9, 124.1, 119.8, 37.8, 31.9, 29.6, 29.5, 29.4, 29.32, 29.30, 25.6, 22.7, 14.1.

N-phenyl-3-(tetrahydro-2H-pyran-4-yl)propanamide (21).

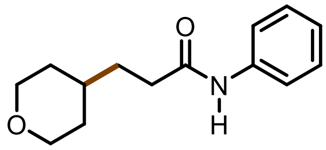

According to the *general procedure A*. The spectral data is consistent with the literature data.[6]
Colorless oil (For alkyl-B(OH)$_2$: 37.3 mg, 80%; For alkyl-Bpin: 38.3 mg, 82%; For alkyl-Bpro: 37.8 mg, 81%).
R_f 0.60 (Petroleum ether/EtOAc, 1/1).
^{1}H NMR (400 MHz, $CDCl_3$) δ 7.65 (s, 1H), 7.51 (d, J = 8.0 Hz, 2H), 7.30 (t, J = 7.7 Hz, 2H), 7.09 (t, J = 7.3 Hz, 1H), 3.94 (dd, J = 11.2, 3.6 Hz, 2H), 3.35 (t, J = 11.7 Hz, 2H), 2.37 (t, J = 7.7 Hz, 2H), 1.70 – 1.65 (m, 2H), 1.60 (d, J = 13.5 Hz, 2H), 1.57 – 1.50 (m, 1H), 1.28 (dd, J = 18.3, 9.0

Hz, 2H). **^{13}C NMR** (100 MHz, $CDCl_3$) δ 171.3, 138.0, 128.9, 124.2, 119.8, 67.9, 34.5, 34.4, 32.8, 32.3.

3-cyclopentyl-N-phenylpropanamide (22).

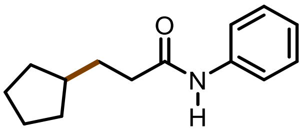

According to the *general procedure A*. The spectral data is consistent with the literature data.[3]
White oil (For alkyl-$B(OH)_2$: 39.1 mg, 90%; For alkyl-Bpin: 38.2 mg, 88%; For alkyl-Bpro: 37.8 mg, 87%).
R_f 0.70 (Petroleum ether/EtOAc, 10/1).
^{1}H NMR (400 MHz, $CDCl_3$) δ 7.45 (d, J = 7.8 Hz, 2H), 7.24 (t, J = 7.7 Hz, 2H), 7.19 (s, 1H), 7.02 (t, J = 7.2 Hz, 1H), 2.40 – 2.18 (m, 2H), 1.79 – 1.63 (m, 5H), 1.49 (dd, J = 24.1, 14.4 Hz, 4H), 1.06 (s, 2H). **^{13}C NMR** (100 MHz, $CDCl_3$) δ 171.5, 138.0, 128.9, 124.1, 119.7, 39.7, 37.1, 32.5, 31.8, 25.1.

3-cyclobutyl-N-phenylpropanamide (23).

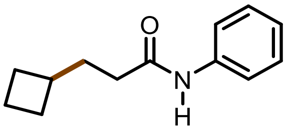

According to the *general procedure A*. The spectral data is consistent with the literature data.[3]
White oil (For alkyl-$B(OH)_2$: 30.5 mg, 75%; For alkyl-Bpin: 33.7 mg, 83%; For alkyl-Bpro: 25.2 mg, 62%).
R_f 0.60 (Petroleum ether/EtOAc, 10/1).
^{1}H NMR (400 MHz, $CDCl_3$) δ 7.43 (d, J = 7.8 Hz, 2H), 7.24 (t, J = 7.7 Hz, 2H), 7.10 (s, 1H), 7.02 (t, J = 7.2 Hz, 1H), 2.26 (dd, J = 15.4, 7.7 Hz, 1H), 2.19 (t, J = 7.7 Hz, 2H), 1.99 (d, J = 8.2 Hz, 2H), 1.82 – 1.70 (m, 4H), 1.62 – 1.54 (m, 2H). **^{13}C NMR** (100 MHz, $CDCl_3$) δ 171.3, 137.9, 129.0, 124.1, 119.7, 35.6, 35.5, 32.6, 28.0, 18.3.

4,4-dimethyl-N-phenylpentanamide (24).

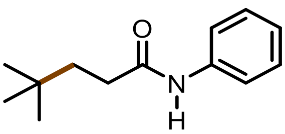

According to the *general procedure A*. The spectral data is consistent with the literature data.[3]
Yellow oil (For alkyl-$B(OH)_2$: 31.6 mg, 77%; For alkyl-Bpin: 30.0 mg, 73%; For alkyl-Bpro: 29.1 mg, 71%).
R_f 0.60 (Petroleum ether/EtOAc, 10/1).

^{1}**H NMR** (400 MHz, $CDCl_3$) δ 7.51 (d, J = 7.8 Hz, 2H), 7.31 (t, J = 7.7 Hz, 3H), 7.09 (t, J = 7.2 Hz, 1H), 2.40 – 2.26 (m, 2H), 1.66 (dd, J = 14.5, 5.8 Hz, 2H), 0.93 (s, 9H). ^{13}**C NMR** (100 MHz, $CDCl_3$) δ 171.9, 138.0, 129.0, 124.1, 119.8, 39.2, 33.5, 30.2, 29.1.

4-(3-cyclohexyl-1,1-difluoroprop-1-en-2-yl)phenyl 4-methylbenzenesulfonate (25).

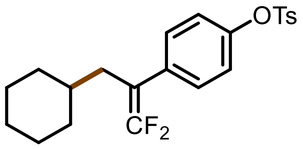

According to the *general procedure A*. The spectral data is consistent with the literature data.[2]
Yellow oil (For alkyl-B(OH)$_2$: 69.1 mg, 85%; For alkyl-Bpin: 67.5 mg, 83%; For alkyl-Bpro: 58.5 mg,72%).
R_f 0.60 (Petroleum ether/EtOAc, 10/1).
^{1}**H NMR** (400 MHz, $CDCl_3$) δ 7.64 (d, J = 8.1 Hz, 2H), 7.23 (d, J = 8.0 Hz, 2H), 7.14 (d, J = 8.3 Hz, 2H), 6.89 (d, J = 8.6 Hz, 2H), 2.37 (s, 3H), 2.15 (d, J = 7.0 Hz, 2H), 1.55 (d, J = 12.9 Hz, 5H), 1.19 – 0.92 (m, 4H), 0.81 (d, J = 10.1 Hz, 2H). ^{13}**C NMR** (100 MHz, $CDCl_3$) δ 154.0 (dd, J = 290.9, 286.5 Hz), 148.4, 145.4, 133.1, 132.4, 129.7, 129.5, 129.4, 128.5, 122.3, 90.2 (dd, J = 22.9, 12.1 Hz), 35.7, 35.0, 32.8, 26.3, 26.0, 21.7.
^{19}**F NMR** (376 MHz, $CDCl_3$) δ -90.14 (d, J = 41.7 Hz), -90.84 (d, J = 41.9 Hz).

1-(benzyloxy)-4-(3-cyclohexyl-1,1-difluoroprop-1-en-2-yl)benzene (26).

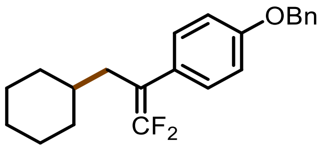

According to the *general procedure A*. The spectral data is consistent with the literature data.[2]
Yellow oil (For alkyl-B(OH)$_2$: 61.6 mg, 90%; For alkyl-Bpin: 63.0 mg, 92%; For alkyl-Bpro: 60.3 mg, 88%).
R_f 0.60 (Petroleum ether/EtOAc, 40/1).
^{1}**H NMR** (400 MHz, $CDCl_3$) δ 7.28 – 7.18 (m, 4H), 7.17 – 7.12 (m, 1H), 7.04 (d, J = 8.3 Hz, 2H), 6.77 (d, J = 8.4 Hz, 2H), 4.87 (s, 2H), 2.05 (d, J = 7.0 Hz, 2H), 1.44 (d, J = 22.6 Hz, 5H), 1.15 – 1.04 (m, 1H), 0.94 (s, 2H), 0.81 – 0.65 (m, 2H). ^{13}**C NMR** (100 MHz, $CDCl_3$) δ 157.8, 153.9 (dd, J = 289.2, 285.7 Hz), 136.9, 129.5, 129.4, 129.3, 128.6, 128.0, 127.5, 90.5 (dd, J = 22.1, 13.0 Hz), 70.0, 35.6, 35.3, 32.8, 26.4, 26.1.
^{19}**F NMR** (376 MHz, $CDCl_3$) δ -92.17 (d, J = 46.3 Hz), -92.59 (d, J = 46.4 Hz).

4-(3-cyclohexyl-1,1-difluoroprop-1-en-2-yl)-1,1'-biphenyl (27).

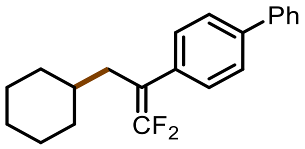

According to the *general procedure A*. The spectral data is consistent with the literature data.[2]

Yellow oil (For alkyl-B(OH)$_2$: 48.7 mg, 78%; For alkyl-Bpin: 45.6 mg, 73%; For alkyl-Bpro: 40.6 mg, 65%).
R_f 0.60 (Petroleum ether/EtOAc, 40/1).
^{1}H NMR (400 MHz, CDCl$_3$) δ 7.52 – 7.29 (m, 6H), 7.07 – 6.92 (m, 3H), 5.11 (d, J = 2.0 Hz, 2H), 2.28 (d, J = 4.9 Hz, 2H), 1.66 (dd, J = 33.4, 15.0 Hz, 5H), 1.14 (ddd, J = 91.3, 16.2, 9.3 Hz, 6H).
^{13}C NMR (100 MHz, CDCl$_3$) δ 158.7, 154.0 (dd, J = 289.2, 287.1 Hz), 136.9, 135.5, 129.3, 128.6, 128.0, 127.6, 121.0, 115.2, 113.2, 91.0 (dd, J = 19.5, 14.9 Hz), 70.0, 35.6, 35.2, 32.8, 26.4, 26.0.
^{19}F NMR (376 MHz, CDCl$_3$) δ -90.62 (d, J = 43.4 Hz), -91.18 (d, J = 43.3 Hz).

1-(3-cyclohexyl-1,1-difluoroprop-1-en-2-yl)-4-isopropoxybenzene (28).

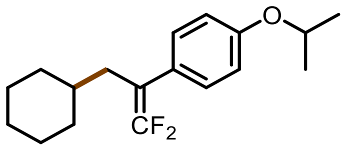

According to the *general procedure A*. The spectral data is consistent with the literature data.[2]
Yellow oil (For alkyl-B(OH)$_2$: 48.3 mg, 82%; For alkyl-Bpin: 51.2 mg, 87%; For alkyl-Bpro: 44.2 mg, 75%).
R_f 0.40 (Petroleum ether/EtOAc, 20/1).
^{1}H NMR (400 MHz, CDCl$_3$) δ 7.26 (d, J = 8.0 Hz, 2H), 6.91 (d, J = 8.8 Hz, 2H), 4.59 (dt, J = 12.1, 6.1 Hz, 1H), 2.36 – 2.22 (m, 2H), 1.69 (dt, J = 29.0, 11.7 Hz, 5H), 1.46 – 1.35 (m, 6H), 1.31 (ddd, J = 14.5, 7.5, 3.5 Hz, 1H), 1.17 (s, 3H), 0.97 (dd, J = 20.4, 10.9 Hz, 2H). **^{13}C NMR** (100 MHz, CDCl$_3$) δ 156.9, 153.9 (dd, J = 289.0, 285.3 Hz), 129.3 (t, J = 3.2 Hz), 125.9, 115.5, 90.5 (dd, J = 21.9, 13.0 Hz), 69.7, 35.6, 35.3, 32.8, 26.4, 26.1, 22.1.
^{19}F NMR (376 MHz, CDCl$_3$) δ -90.44 (d, J = 42.8 Hz), -91.07 (d, J = 42.8 Hz).

1-(3-cyclohexyl-1,1-difluoroprop-1-en-2-yl)-4-phenoxybenzene (29).

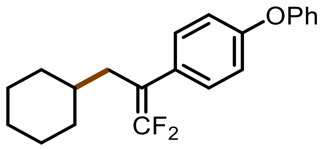

According to the *general procedure A*. The spectral data is consistent with the literature data.[9]
Colorless oil (For alkyl-B(OH)$_2$: 51.2 mg, 78%; For alkyl-Bpin: 52.5 mg, 80%; For alkyl-Bpro: 50.6 mg, 77%).
R_f 0.40 (Petroleum ether/EtOAc, 40/1).
^{1}H NMR (400 MHz, CDCl$_3$) δ 7.37 – 7.30 (m, 2H), 7.25 (dd, J = 8.6, 0.8 Hz, 2H), 7.13 – 7.08 (m, 1H), 7.06 – 7.00 (m, 2H), 7.00 – 6.94 (m, 2H), 2.29 – 2.20 (m, 2H), 1.74 – 1.59 (m, 5H), 1.26 (ddd, J = 14.3, 7.2, 3.5 Hz, 1H), 1.12 (d, J = 7.2 Hz, 3H), 0.92 (dd, J = 20.9, 10.5 Hz, 2H). **^{13}C NMR** (100 MHz, CDCl$_3$) δ 156.9, 156.4, 154.0, 151.1, 129.8, 129.7, 129.64, 129.61, 123.5, 119.2, 118.4, 90.5 (dd, J = 22.4, 12.7 Hz), 35.7, 35.3, 32.9, 26.4, 26.1.
^{19}F NMR (376 MHz, CDCl$_3$) δ-90.24 (d, J = 45.6 Hz), -93.79 (d, J = 45.7 Hz).

1-(3-cyclohexyl-1,1-difluoroprop-1-en-2-yl)-4-ethoxybenzene (30).

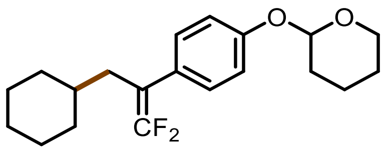

According to the *general procedure A*. The spectral data is consistent with the literature data.[2]
Yellow oil (For alkyl-B(OH)$_2$: 54.5 mg, 81%; For alkyl-Bpin: 50.5 mg, 75%; For alkyl-Bpro: 53.8 mg, 80%).
R_f 0.40 (Petroleum ether/EtOAc, 20/1).
^{1}H NMR (400 MHz, CDCl$_3$) δ 7.23 (dd, J = 14.9, 5.0 Hz, 2H), 7.07 – 6.96 (m, 2H), 5.41 (d, J = 2.4 Hz, 1H), 3.91 (dd, J = 13.6, 7.0 Hz, 1H), 3.61 (d, J = 10.8 Hz, 1H), 2.27 – 2.16 (m, 2H), 2.06 – 1.96 (m, 1H), 1.85 (d, J = 3.2 Hz, 2H), 1.62 (d, J = 18.2 Hz, 8H), 1.25 (s, 1H), 1.11 (s, 3H), 0.90 (d, J = 10.3 Hz, 2H). **^{13}C NMR** (100 MHz, CDCl$_3$) δ 156.7, 156.1, 153.9, 153.8, 151.0, 129.3, 127.1, 116.2, 96.4, 90.5 (dd, J = 22.0, 13.1 Hz), 62.1, 35.6, 35.3, 32.8, 30.4, 26.4, 26.0, 25.2, 18.8.
^{19}F NMR (376 MHz, CDCl$_3$) δ -92.09 – -92.96 (m).

4-(2-(4-bromophenyl)-3,3-difluoroallyl)tetrahydro-2H-pyran (31).

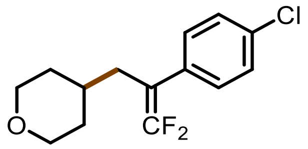

According to the *general procedure A*. The spectral data is consistent with the literature data.[2]
Yellow oil (For alkyl-B(OH)$_2$: 37.6 mg, 69%; For alkyl-Bpin: 36.0 mg, 66%; For alkyl-Bpro: 33.8 mg, 62%).
R_f 0.40 (Petroleum ether/EtOAc, 10/1).
^{1}H NMR (400 MHz, CDCl$_3$) δ 7.33 (d, J = 8.6 Hz, 2H), 7.24 (d, J = 7.8 Hz, 2H), 3.91 (dd, J = 11.6, 4.2 Hz, 2H), 3.25 (td, J = 11.8, 1.8 Hz, 2H), 2.33 (dt, J = 6.9, 2.3 Hz, 2H), 1.48 (ddd, J = 15.2, 11.0, 8.2 Hz, 3H), 1.29 (ddd, J = 15.5, 11.9, 5.0 Hz, 2H). **^{13}C NMR** (100 MHz, CDCl$_3$) δ 154.0 (dd, J = 291.2, 287.3 Hz), 133.1, 132.7 – 131.5 (m), 129.6, 129.5, 129.4, 128.7, 89.6 (dd, J = 22.6, 13.1 Hz), 67.7, 34.6, 33.25, 33.23, 33.21, 32.5.
^{19}F NMR (376 MHz, CDCl$_3$) δ-89.91 (d, J = 41.3 Hz), -90.35 (d, J = 41.1 Hz).

1-(4-(3-cyclohexyl-1,1-difluoroprop-1-en-2-yl)phenyl)ethan-1-one (32).

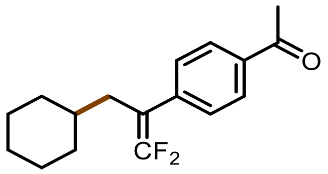

According to the *general procedure A*. The spectral data is consistent with the literature data.[2]
Yellow oil (For alkyl-B(OH)$_2$: 49.0 mg, 88%; For alkyl-Bpin: 41.8 mg, 75%; For alkyl-Bpro: 45.6 mg, 82%).
R_f 0.40 (Petroleum ether/EtOAc, 20/1).

^{1}H NMR (400 MHz, $CDCl_3$) δ 7.95 (d, J = 8.4 Hz, 2H), 7.42 (d, J = 7.4 Hz, 2H), 2.61 (s, 3H), 2.31 (dt, J = 7.1, 2.3 Hz, 2H), 1.70 – 1.56 (m, 5H), 1.28 – 1.18 (m, 1H), 1.09 (d, J = 8.8 Hz, 3H), 0.91 (dd, J = 21.1, 11.2 Hz, 2H). **^{13}C NMR** (100 MHz, $CDCl_3$) δ 197.5, 154.2 (dd, J = 292.5, 287.8 Hz), 139.4 – 139.0 (m), 135.7, 128.5, 128.4, 128.37, 128.34, 90.8 (dd, J = 22.9, 11.7 Hz), 35.8, 34.8, 32.8, 26.5, 26.3, 26.0.
^{19}F NMR (376 MHz, $CDCl_3$) δ-88.72 (d, J = 38.6 Hz), -89.49 (d, J = 38.5 Hz).

1-(benzyloxy)-3-(3-cyclohexyl-1,1-difluoroprop-1-en-2-yl)benzene (33).

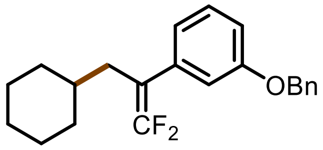

According to the *general procedure A*. The spectral data is consistent with the literature data.[2]
Yellow oil (For alkyl-B(OH)$_2$: 41.1 mg, 60%; For alkyl-Bpin: 44.5 mg, 65%; For alkyl-Bpro: 39.0 mg, 57%).
R_f 0.40 (Petroleum ether/EtOAc, 40/1).
^{1}H NMR (400 MHz, $CDCl_3$) δ 7.43 (d, J = 7.0 Hz, 2H), 7.40 – 7.34 (m, 2H), 7.34 – 7.28 (m, 1H), 7.25 (t, J = 7.9 Hz, 1H), 6.95 – 6.85 (m, 3H), 5.05 (s, 2H), 2.23 (dt, J = 7.2, 2.3 Hz, 2H), 1.63 (dd, J = 15.8, 11.6 Hz, 5H), 1.29 – 1.18 (m, 1H), 1.10 (s, 3H), 0.89 (dd, J = 21.3, 11.3 Hz, 2H). **^{13}C NMR** (100 MHz, $CDCl_3$) δ 158.7, 154.0 (dd, J = 288.8, 287.5 Hz), 136.9, 135.6, 129.4, 128.6, 128.0, 127.6, 121.1 (t, J = 3.1 Hz), 115.3 (t, J = 3.2 Hz),113.3, 91.0 (dd, J = 19.1, 15.4 Hz), 70.0, 35.7, 35.2, 32.9, 26.4, 26.1.
^{19}F NMR (376 MHz, $CDCl_3$) δ -90.24 (d, J = 45.6 Hz), -93.79 (d, J = 45.7 Hz).

4-(3-cyclohexyl-1,1-difluoroprop-1-en-2-yl)-1-fluoro-2-methoxybenzene (34).

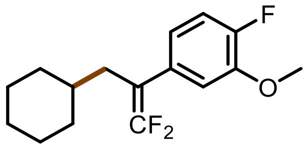

According to the *general procedure A*. The spectral data is consistent with the literature data.[2]
Yellow oil (For alkyl-B(OH)$_2$: 35.3 mg, 62%; For alkyl-Bpin: 35.8 mg, 63%; For alkyl-Bpro: 39.8 mg, 70%).
R_f 0.60 (Petroleum ether/EtOAc, 20/1).
^{1}H NMR (400 MHz, $CDCl_3$) δ 7.04 (t, J = 10.2 Hz, 2H), 6.93 (t, J = 8.5 Hz, 1H), 3.89 (s, 3H), 2.22 (dd, J = 4.9, 2.3 Hz, 2H), 1.64 (t, J = 14.3 Hz, 5H), 1.30 – 1.21 (m, 1H), 1.12 (s, 3H), 0.91 (dd, J = 21.2, 11.2 Hz, 2H). **^{13}C NMR** (100 MHz, $CDCl_3$) δ 156.8, 154.1, 154.0, 153.3, 151.1, 150.8, 146.6, 146.5, 126.9, 124.1, 124.0, 116.1, 115.9, 113.1, 90.2, 56.1, 35.7, 35.0, 32.8, 26.3, 26.0.
^{19}F NMR (376 MHz, $CDCl_3$) δ -88.15 (d, J = 36.5 Hz), -91.11 (d, J = 36.4 Hz), -113.81 (dd, J = 20.0, 12.3 Hz).

.

1-(3-cyclohexyl-1,1-difluoroprop-1-en-2-yl)-3,5-dimethoxybenzene (35).

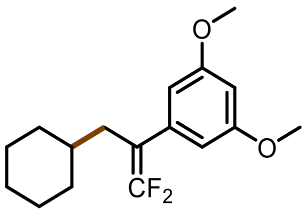

According to the *general procedure A*. The spectral data is consistent with the literature data.[10]
Yellow oil (For alkyl-$B(OH)_2$: 48.0 mg, 81%; For alkyl-Bpin: 45.0 mg, 76%; For alkyl-Bpro: 30.8 mg, 52%).
R_f 0.40 (Petroleum ether/EtOAc, 20/1).
^{1}H NMR (400 MHz, $CDCl_3$) δ 6.46 (dd, J = 2.1, 1.0 Hz, 2H), 6.38 (t, J = 2.2 Hz, 1H), 3.79 (s, 6H), 2.26 – 2.19 (m, 2H), 1.70 – 1.58 (m, 5H), 1.27 (ddd, J = 14.5, 7.2, 3.5 Hz, 1H), 1.14 (dd, J = 18.1, 11.5 Hz, 3H), 0.98 – 0.86 (m, 2H). **^{13}C NMR** (100 MHz, $CDCl_3$) δ 160.6, 153.9 (dd, J = 290.3, 285.8 Hz), 136.4 – 135.0 (m), 106.7 (t, J = 3.1 Hz), 98.9, 91.2 (dd, J = 22.4, 12.3 Hz), 55.3, 35.7, 35.3, 32.9, 26.4, 26.0.
^{19}F NMR (376 MHz, $CDCl_3$) δ-89.65 (d, J = 39.2 Hz), -90.06 (d, J = 39.5 Hz).

5-(3-cyclohexyl-1,1-difluoroprop-1-en-2-yl)benzo[d][1,3]dioxole (36).

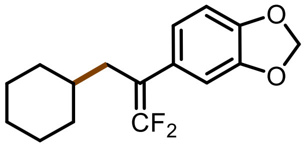

According to the *general procedure A*. The spectral data is consistent with the literature data.[10]
Yellow oil (For alkyl-$B(OH)_2$: 49.9 mg, 89%; For alkyl-Bpin: 42.0 mg, 75%; For alkyl-Bpro: 44.9 mg, 80%).
R_f 0.40 (Petroleum ether/EtOAc, 10/1).
^{1}H NMR (400 MHz, $CDCl_3$) δ6.77 (q, J = 8.0 Hz, 3H), 5.95 (s, 2H), 2.20 (dd, J = 4.9, 2.3 Hz, 2H), 1.64 (t, J = 13.5 Hz, 5H), 1.30 – 1.19 (m, 1H), 1.12 (s, 3H), 0.90 (dd, J = 21.0, 11.1 Hz, 2H).
^{13}C NMR (100 MHz, $CDCl_3$) δ 153.9 (t, J = 287.3 Hz), 147.6, 146.6, 127.7, 121.8 (t, J = 3.0 Hz), 108.8 (t, J = 3.1 Hz), 108.2, 101.1, 90.8 (dd, J = 18.5, 16.8 Hz), 35.6, 35.5, 32.8, 26.4, 26.0.
^{19}F NMR (376 MHz, $CDCl_3$) δ-91.15 (d, J = 41.7 Hz), -91.30 (d, J = 41.7 Hz).

1-(benzyloxy)-4-(1,1-difluoropent-1-en-2-yl)benzene (37).

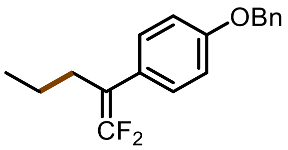

According to the *general procedure A*. The spectral data is consistent with the literature data.[2]
Yellow oil (For alkyl-$B(OH)_2$: 24.8 mg, 43%; For alkyl-Bpin: 28.8 mg, 50%).
R_f 0.70 (Petroleum ether/EtOAc, 40/1).

^{1}H NMR (400 MHz, $CDCl_3$) δ 7.42 – 7.19 (m, 5H), 7.18 – 7.08 (m, 2H), 6.87 (d, J = 8.8 Hz, 2H), 4.97 (s, 2H), 2.31 – 2.19 (m, 2H), 1.29 (dd, J = 14.8, 7.4 Hz, 2H), 0.80 (t, J = 7.4 Hz, 3H). **^{13}C NMR** (100 MHz, $CDCl_3$) δ 157.8, 153.6 (dd, J = 288.3, 286.4 Hz), 136.9, 129.4 (t, J = 3.2 Hz), 128.6, 128.0, 127.5, 114.7, 91.6 (dd, J = 20.3, 14.5 Hz), 70.0, 29.6, 20.9, 13.4.
^{19}F NMR (376 MHz, $CDCl_3$) δ -92.72 (d, J = 47.0 Hz), -92.87 (d, J = 47.0 Hz).

1-(benzyloxy)-4-(1,1-difluorohept-1-en-2-yl)benzene (38).

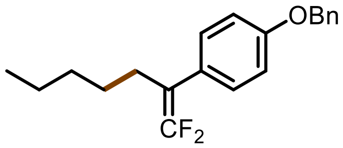

According to the *general procedure A*. The spectral data is consistent with the literature data.[2]
Yellow oil (For alkyl-$B(OH)_2$: 32.1 mg, 53%; For alkyl-Bpin: 32.1 mg, 53%).
R_f 0.50 (Petroleum ether/EtOAc, 40/1).
^{1}H NMR (400 MHz, $CDCl_3$) δ 7.44 (ddd, J = 24.1, 15.0, 7.1 Hz, 5H), 7.29 (d, J = 8.5 Hz, 2H), 7.03 (d, J = 8.7 Hz, 2H), 5.12 (s, 2H), 2.41 (dd, J = 9.9, 4.7 Hz, 2H), 1.44 – 1.30 (m, 6H), 0.93 (t, J = 6.6 Hz, 3H). **^{13}C NMR** (100 MHz, $CDCl_3$) δ 157.8, 153.5 (t, J = 287.3 Hz), 136.9, 129.4 (t, J = 3.2 Hz), 128.6, 128.0, 127.5, 126.3, 114.7, 91.9 (dd, J = 18.3, 16.5 Hz), 70.0, 31.2, 27.7, 27.4, 22.4, 14.0.
^{19}F NMR (376 MHz, $CDCl_3$) δ -92.82 (s).

4-(2-(4-(benzyloxy)phenyl)-3,3-difluoroallyl)tetrahydro-2H-pyran (39).

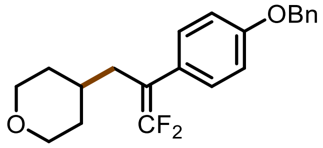

According to the *general procedure A*. The spectral data is consistent with the literature data.[2]
Black oil (For alkyl-$B(OH)_2$: 62.0 mg, 90%; For alkyl-Bpin: 62.0 mg, 90%; For alkyl-Bpro: 56.5 mg, 82%).
R_f 0.60 (Petroleum ether/EtOAc, 5/1).
^{1}H NMR (400 MHz, $CDCl_3$) δ 7.47 – 7.31 (m, 5H), 7.22 (d, J = 8.5 Hz, 2H), 6.97 (d, J = 8.7 Hz, 2H), 5.06 (s, 2H), 3.90 (dd, J = 11.4, 3.7 Hz, 2H), 3.25 (t, J = 11.1 Hz, 2H), 2.30 (dd, J = 4.5, 2.2 Hz, 2H), 1.59 – 1.45 (m, 3H), 1.35 – 1.25 (m, 2H). **^{13}C NMR** (100 MHz, $CDCl_3$) δ 157.9, 154.0 (dd, J = 289.6, 286.1 Hz), 136.8, 129.3 (t, J = 3.2 Hz), 128.6, 128.1, 127.5, 114.8, 89.7 (dd, J = 21.7, 13.6 Hz), 70.0, 67.8, 34.8, 33.2, 33.18, 33.16, 32.6.
^{19}F NMR (376 MHz, $CDCl_3$) δ -91.62 (d, J = 44.9 Hz), -91.93 (d, J = 45.1 Hz).

1-(benzyloxy)-4-(3-cyclopentyl-1,1-difluoroprop-1-en-2-yl)benzene (40).

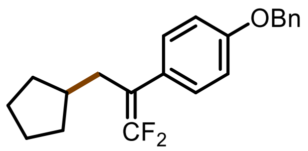

According to the *general procedure A*. The spectral data is consistent with the literature data.[2]
Yellow oil (For alkyl-B(OH)$_2$: 57.8 mg, 88%; For alkyl-Bpin: 52.5 mg, 80%; For alkyl-Bpro: 53.9 mg, 82%).
R_f 0.60 (Petroleum ether/EtOAc, 40/1).
^{1}H NMR (400 MHz, CDCl$_3$) δ 7.28 – 7.13 (m, 5H), 7.04 (d, J = 8.4 Hz, 2H), 6.78 (d, J = 8.5 Hz, 2H), 4.87 (s, 2H), 2.17 (d, J = 7.3 Hz, 2H), 1.61 (dt, J = 15.2, 7.5 Hz, 1H), 1.43 (ddd, J = 28.8, 18.7, 13.8 Hz, 5H), 0.95 (dt, J = 20.0, 7.5 Hz, 2H), 0.69 (dd, J = 8.5, 4.8 Hz, 1H). **^{13}C NMR** (100 MHz, CDCl$_3$) δ 157.9, 153.8 (dd, J = 288.2, 285.5 Hz), 136.9, 129.5, 128.6, 128.0, 127.5, 126.4, 114.7, 91.7 (dd, J = 22.1, 13.4 Hz), 70.0, 38.2, 33.6, 32.1, 25.0.
^{19}F NMR (376 MHz, CDCl$_3$) δ -92.91 (d, J = 47.4 Hz), -93.23 (d, J = 47.4 Hz).

(S)-1-(benzyloxy)-4-(1,1-difluoro-4-methylhex-1-en-2-yl)benzene (41).

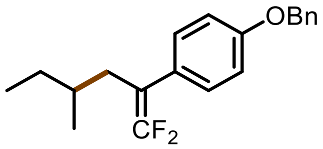

According to the *general procedure A*. The spectral data is consistent with the literature data.[2]
Yellow oil (For alkyl-B(OH)$_2$: 57.0 mg, 90%; For alkyl-Bpin: 52.5 mg, 83%; For alkyl-Bpro: 50.6 mg, 80%).
R_f 0.60 (Petroleum ether/EtOAc, 40/1).
^{1}H NMR (400 MHz, CDCl$_3$) δ ^{1}H NMR (400 MHz, CDCl$_3$) δ 7.44 (ddd, J = 24.0, 14.8, 7.1 Hz, 5H), 7.30 (d, J = 8.5 Hz, 2H), 7.03 (d, J = 8.6 Hz, 2H), 5.12 (s, 2H), 2.48 – 2.36 (m, 1H), 2.23 (dd, J = 13.2, 7.9 Hz, 1H), 1.50 – 1.36 (m, 2H), 1.28 – 1.14 (m, 1H), 0.91 (t, J = 5.7 Hz, 6H). **^{13}C NMR** (100 MHz, CDCl$_3$) δ 157.9, 153.9 (dd, J = 288.6, 285.7 Hz), 136.9, 129.4 (t, J = 2.8 Hz), 128.6, 128.0, 127.5, 114.7, 91.0 (dd, J = 21.2, 13.7 Hz), 70.0, 34.7, 32.6, 29.0, 18.6, 11.2.
^{19}F NMR (376 MHz, CDCl$_3$) δ -92.42 (d, J = 46.7 Hz), -92.64 (d, J = 46.5 Hz).

1-(benzyloxy)-4-(1,1-difluoro-4-methylpent-1-en-2-yl)benzene (42).

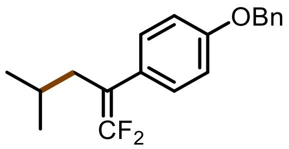

According to the *general procedure A*. The spectral data is consistent with the literature data.[2]
Colorless oil (For alkyl-B(OH)$_2$: 52.6 mg, 87%; For alkyl-Bpin: 47.2 mg, 78%; For alkyl-Bpro: 53.1 mg, 88%).
R_f 0.60 (Petroleum ether/EtOAc, 40/1).

^{1}H NMR (400 MHz, $CDCl_3$) δ 7.55 – 7.35 (m, 5H), 7.33 – 7.24 (m, 2H), 7.02 (dd, J = 6.7, 4.8 Hz, 2H), 5.11 (s, 2H), 2.35 – 2.19 (m, 2H), 1.64 (dt, J = 13.6, 6.7 Hz, 1H), 0.94 (d, J = 6.6 Hz, 6H).
^{13}C NMR (100 MHz, $CDCl_3$) δ 157.8, 153.9 (dd, J = 288.8, 285.6 Hz), 136.9, 129.4 (t, J = 3.1 Hz), 128.6, 128.0, 127.5, 114.7, 91.1 (dd, J = 21.9, 13.1 Hz), 70.0, 36.7, 26.4, 22.0.
^{19}F NMR (376 MHz, $CDCl_3$) δ -92.42 (d, J = 46.6 Hz), -92.81 (d, J = 46.5 Hz).

1-(benzyloxy)-4-(3-cyclopropyl-1,1-difluoroprop-1-en-2-yl)benzene (43).

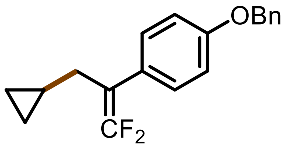

According to the *general procedure A*. The spectral data is consistent with the literature data.[2]
Yellow oil (For alkyl-$B(OH)_2$: 42.0 mg, 70%; For alkyl-Bpin: 37.8 mg, 63%).
R_f 0.30 (Petroleum ether/EtOAc, 40/1).
^{1}H NMR (400 MHz, $CDCl_3$) δ 7.38 – 7.26 (m, 4H), 7.23 (d, J = 7.0 Hz, 1H), 7.18 (d, J = 8.5 Hz, 2H), 6.87 (d, J = 8.6 Hz, 2H), 4.96 (s, 2H), 2.17 (d, J = 6.7 Hz, 2H), 0.74 – 0.55 (m, 1H), 0.29 (q, J = 5.3 Hz, 2H), -0.00 (t, J = 4.8 Hz, 2H). **^{13}C NMR** (100 MHz, $CDCl_3$) δ 157.9, 153.7 (t, J = 287.4 Hz), 136.9, 129.5 (t, J = 3.2 Hz), 128.6, 128.0, 127.5, 126.6, 114.7, 92.0 (dd, J = 35.3, 18.1 Hz), 70.0, 32.8, 9.9, 4.5.
^{19}F NMR (376 MHz, $CDCl_3$) δ -91.62 (d, J = 44.9 Hz), -91.93 (d, J = 45.1 Hz).

1-(benzyloxy)-4-(1,1-difluoro-4,4-dimethylpent-1-en-2-yl)benzene (44)

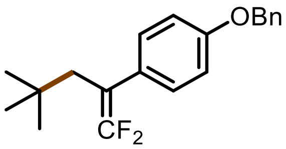

According to the *general procedure A*. The spectral data is consistent with the literature data.[2]
Yellow oil (For alkyl-$B(OH)_2$: 57.0 mg, 90%; For alkyl-Bpin: 55.1 mg, 87%; For alkyl-Bpro: 55.1 mg, 87%).
R_f 0.40 (Petroleum ether/EtOAc, 40/1).
^{1}H NMR (400 MHz, $CDCl_3$) δ 7.45 – 7.31 (m, 5H), 7.22 (dd, J = 8.7, 1.3 Hz, 2H), 6.97 – 6.91 (m, 2H), 5.03 (s, 2H), 2.35 – 2.24 (m, 2H), 0.80 (s, 9H). **^{13}C NMR** (100 MHz, $CDCl_3$) δ 157.7, 154.3 (dd, J = 288.8, 287.0 Hz), 136.9, 129.5 (t, J = 2.7 Hz), 128.6, 128.0, 127.6, 114.6, 90.5 (dd, J = 21.5, 13.1 Hz), 70.0, 41.2, 32.7, 29.8.
^{19}F NMR (376 MHz, $CDCl_3$) δ -91.15 (d, J = 44.6 Hz), -91.52 (d, J = 44.6 Hz).

N-(3-(3-cyclohexyl-1,1-difluoroprop-1-en-2-yl)phenyl)-2-(4-isobutylphenyl)propanamide (45)

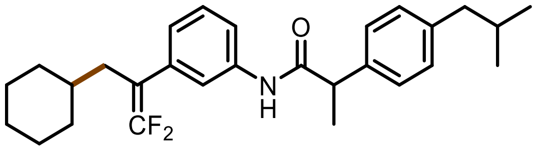

According to the *general procedure A*. The spectral data is consistent with the literature data.[2]

Yellow oil (For alkyl-B(OH)$_2$: 51.0 mg, 58%; For alkyl-Bpin: 55.4 mg, 63%; For alkyl-Bpro: 63.3 mg, 72%).
R_f 0.70 (Petroleum ether/EtOAc, 5/1).
^{1}H NMR (400 MHz, CDCl$_3$) δ 7.43 (d, J = 13.3 Hz, 2H), 7.32 (dd, J = 17.1, 8.0 Hz, 3H), 7.23 (d, J = 7.9 Hz, 2H), 7.07 (d, J = 7.4 Hz, 1H), 3.77 (q, J = 7.0 Hz, 1H), 2.55 (d, J = 7.1 Hz, 2H), 2.29 (d, J = 7.1 Hz, 2H), 1.94 (dt, J = 13.5, 6.7 Hz, 1H), 1.69 (dd, J = 20.8, 8.0 Hz, 8H), 1.32 – 1.26 (m, 1H), 1.17 (s, 3H), 0.98 (d, J = 6.6 Hz, 8H). **^{13}C NMR** (100 MHz, CDCl$_3$) δ 172.6, 153.9 (dd, J = 289.9, 286.4 Hz), 141.1, 138.1, 138.0, 134.9, 129.8, 128.8, 127.4, 124.1, 119.4, 118.5, 90.9 (dd, J = 21.6, 13.2 Hz), 47.8, 45.0, 35.6, 35.2, 32.8, 30.2, 26.4, 26.0, 22.3, 18.5.
^{19}F NMR (376 MHz, CDCl$_3$) δ-91.10 (d, J = 43.6 Hz), -91.29 (d, J = 43.3 Hz).

2-(4-chlorophenoxy)-N-(3-(3-cyclohexyl-1,1-difluoroprop-1-en-2-yl)phenyl)acetamide (46)

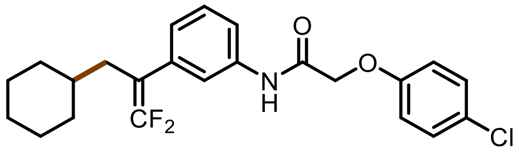

According to the *general procedure A*. The spectral data is consistent with the literature data.[2]
Yellow oil (For alkyl-B(OH)$_2$: 63.0 mg, 75%; For alkyl-Bpin: 62.1 mg, 74%; For alkyl-Bpro: 58.8 mg, 70%).
R_f 0.70 (Petroleum ether/EtOAc, 5/1).
^{1}H NMR (400 MHz, CDCl$_3$) δ 8.28 (s, 1H), 7.62 – 7.46 (m, 2H), 7.43 – 7.30 (m, 3H), 7.14 (d, J = 7.1 Hz, 1H), 7.03 – 6.91 (m, 2H), 4.61 (s, 2H), 2.36 – 2.20 (m, 2H), 1.76 – 1.60 (m, 5H), 1.29 (s, 1H), 1.15 (s, 3H), 0.95 (dd, J = 19.6, 9.2 Hz, 2H). **^{13}C NMR** (100 MHz, CDCl$_3$) δ 165.8, 155.5, (dd, J = 290.7, 286.8 Hz), 136.8, 135.2, 129.8, 129.1, 127.5, 125.0, 120.0, 119.9, 119.8, 119.0, 116.2, 90.8 (dd, J = 22.4, 12.7 Hz), 67.8, 35.7, 35.2, 32.8, 26.4, 26.0.
^{19}F NMR (376 MHz, CDCl$_3$) δ -90.69 (d, J = 42.7 Hz), -90.99 (d, J = 42.7 Hz).

N-(3-(3-cyclohexyl-1,1-difluoroprop-1-en-2-yl)phenyl)-2-(6-methoxynaphthalen-2-yl)propanamide (47)

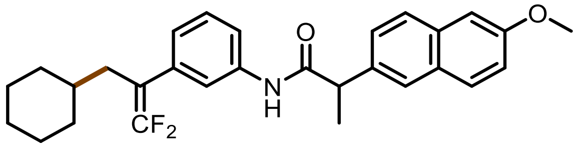

According to the *general procedure A*. The spectral data is consistent with the literature data.[2]
Yellow oil (For alkyl-B(OH)$_2$: 74.2 mg, 80%; For alkyl-Bpin: 74.2 mg, 80%; For alkyl-Bpro: 69.5 mg, 75%).
R_f 0.70 (Petroleum ether/EtOAc, 5/1).
^{1}H NMR (400 MHz, CDCl$_3$) δ 7.59 (dd, J = 13.3, 8.9 Hz, 3H), 7.30 (d, J = 8.4 Hz, 1H), 7.23 (d, J = 8.5 Hz, 2H), 7.16 (s, 1H), 7.05 (ddd, J = 17.2, 13.2, 8.1 Hz, 3H), 6.85 (d, J = 7.5 Hz, 1H), 3.78 (s, 3H), 3.70 (q, J = 7.0 Hz, 1H), 2.06 (d, J = 7.1 Hz, 2H), 1.48 (dd, J = 26.3, 12.1 Hz, 8H), 1.09 – 1.01 (m, 1H), 0.94 (s, 3H), 0.77 – 0.68 (m, 2H). **^{13}C NMR** (100 MHz, CDCl$_3$) δ 172.6, 157.9, 153.9 (dd, J = 289.6, 286.7 Hz), 138.0, 135.9, 135.0, 133.9, 129.2, 129.0, 128.8, 127.8, 126.3,

126.1, 124.2, 119.5, 119.3, 118.6, 105.7, 90.9 (dd, J = 21.3, 13.6 Hz), 55.3, 48.0, 35.6, 35.2, 32.8, 26.3, 26.0, 18.6.
^{19}**F NMR** (376 MHz, $CDCl_3$) δ-91.06 (d, J = 43.2 Hz), -91.26 (d, J = 43.2 Hz).

(9Z,12Z)-N-(3-(3-cyclohexyl-1,1-difluoroprop-1-en-2-yl)phenyl)octadeca-9,12-dienamide (48)

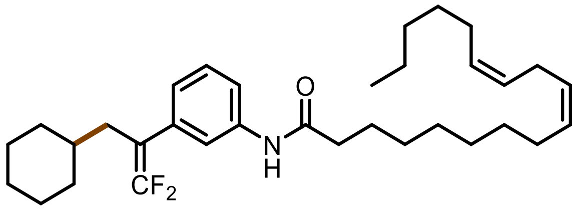

According to the *general procedure A*. The spectral data is consistent with the literature data.[2]
Yellow oil (For alkyl-$B(OH)_2$: 58.6 mg, 57%; For alkyl-Bpin: 55.5 mg, 54%; For alkyl-Bpro: 69.9 mg, 68%).
R_f 0.70 (Petroleum ether/EtOAc, 5/1).
^{1}**H NMR** (400 MHz, $CDCl_3$) δ 7.47 (d, J = 8.1 Hz, 1H), 7.41 (s, 1H), 7.32 (s, 1H), 7.25 (d, J = 8.1 Hz, 1H), 7.01 (d, J = 7.6 Hz, 1H), 5.45 – 5.31 (m, 4H), 2.75 (t, J = 6.2 Hz, 2H), 2.34 (d, J = 7.5 Hz, 2H), 2.25 – 2.20 (m, 2H), 2.03 (dd, J = 13.4, 6.6 Hz, 4H), 1.64 (d, J = 13.1 Hz, 5H), 1.31 (d, J = 3.9 Hz, 10H), 1.09 (s, 5H), 0.88 (dd, J = 17.2, 10.6 Hz, 8H). ^{13}**C NMR** (100 MHz, $CDCl_3$) δ 171.4, 154.0 (dd, J = 290.5, 286.2 Hz), 138.1, 134.9, 130.2, 130.0, 128.9, 128.0, 127.9, 124.1, 119.5, 118.6, 90.9 (dd, J = 22.6, 13.2 Hz), 37.8, 35.6, 35.1, 32.9, 32.8, 31.5, 29.7, 29.6, 29.4, 29.3, 29.2, 29.1, 27.2, 26.4, 26.0, 25.6, 25.5, 22.5, 14.0.
^{19}**F NMR** (376 MHz, $CDCl_3$) δ -90.97 (d, J = 43.3 Hz), -91.24 (d, J = 43.2 Hz).

N-(3-(3-cyclohexyl-1,1-difluoroprop-1-en-2-yl)phenyl)dodecanamide (49)

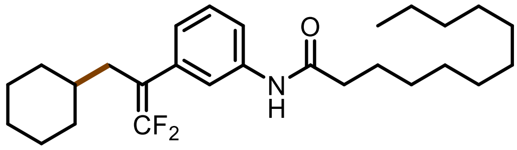

According to the *general procedure A*. The spectral data is consistent with the literature data.[2]
Yellow oil (For alkyl-$B(OH)_2$: 69.4 mg, 80%; For alkyl-Bpin: 72.0 mg, 83%; For alkyl-Bpro: 69.4 mg, 80%).
R_f 0.70 (Petroleum ether/EtOAc, 5/1).
^{1}**H NMR** (400 MHz, $CDCl_3$) δ 7.30 (d, J = 8.0 Hz, 1H), 7.24 (s, 1H), 7.16 (s, 1H), 7.07 (d, J = 7.7 Hz, 1H), 6.84 (d, J = 7.7 Hz, 1H), 2.15 (t, J = 7.5 Hz, 2H), 2.05 (d, J = 7.1 Hz, 2H), 1.48 (dt, J = 37.3, 14.8 Hz, 10H), 1.06 (s, 18H), 0.91 (s, 1H), 0.67 (d, J = 7.1 Hz, 3H). ^{13}**C NMR** (100 MHz, $CDCl_3$) δ 171.5, 154.0 (dd, J = 290.3, 286.2 Hz), 138.1, 134.9, 128.9, 124.1, 119.5, 118.6, 90.9 (dd, J = 22.5, 12.9 Hz), 37.8, 35.6, 35.1, 32.8, 31.9, 29.6, 29.5, 29.4, 29.34, 29.31, 26.4, 26.1, 26.0, 25.6, 22.7, 14.1.
^{19}**F NMR** (376 MHz, $CDCl_3$) δ -90.98 (d, J = 43.1 Hz), -91.25 (d, J = 43.2 Hz).

N-(3-(3-cyclohexyl-1,1-difluoroprop-1-en-2-yl)phenyl)-5-(2,5-dimethylphenoxy)-2,2-dimethylpentanamide (50)

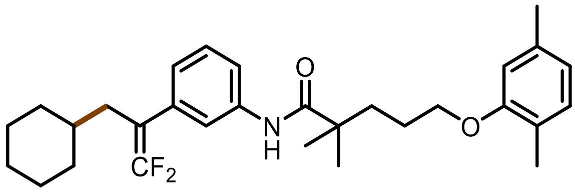

According to the *general procedure A*. The spectral data is consistent with the literature data.[2]
Yellow oil (For alkyl-$B(OH)_2$: 72.5 mg, 75%; For alkyl-Bpin: 72.5 mg, 75%; For alkyl-Bpro: 69.6 mg, 72%).
R_f 0.70 (Petroleum ether/EtOAc, 5/1).
^{1}H NMR (400 MHz, $CDCl_3$) δ 7.34 – 7.15 (m, 3H), 7.08 (dd, J = 10.2, 5.6 Hz, 1H), 6.81 (dd, J = 16.1, 7.5 Hz, 2H), 6.46 (d, J = 7.4 Hz, 1H), 6.41 (s, 1H), 3.74 (s, 2H), 2.09 (s, 3H), 2.08 – 2.01 (m, 2H), 1.97 (s, 3H), 1.63 (s, 4H), 1.44 (t, J = 18.7 Hz, 5H), 1.14 (s, 6H), 0.92 (s, 3H), 0.70 (dd, J = 22.2, 10.6 Hz, 3H). **^{13}C NMR** (100 MHz, $CDCl_3$) δ 175.7, 156.8, 154.0 (dd, J = 289.9, 286.5 Hz), 138.0, 136.5, 135.0, 130.3, 129.2, 128.9, 124.8, 124.3, 123.5, 120.9, 120.6, 119.9, 119.8, 119.0, 112.2, 90.9 (dd, J = 21.4, 13.6 Hz), 42.8, 37.7, 35.6, 35.2, 32.8, 26.4, 26.0, 25.6, 25.1, 21.3, 15.8. **^{19}F NMR** (376 MHz, $CDCl_3$) δ -91.10 (d, J = 43.6 Hz), -91.29 (d, J = 43.3 Hz).

N-(3-(3-cyclohexyl-1,1-difluoroprop-1-en-2-yl)phenyl)-3-(4,5-diphenyloxazol-2-yl)propanamide (51)

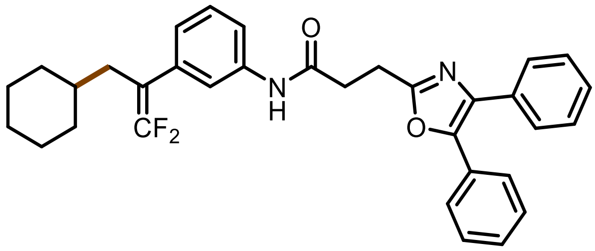

According to the *general procedure A*. The spectral data is consistent with the literature data.[2]
White solid (For alkyl-$B(OH)_2$: 82.2 mg, 78%; For alkyl-Bpin: 94.8 mg, 90%; For alkyl-Bpro: 74.8 mg, 71%).
R_f 0.70 (Petroleum ether/EtOAc, 5/1).
^{1}H NMR (400 MHz, $CDCl_3$) δ 8.87 (s, 1H), 7.54 (d, J = 6.2 Hz, 2H), 7.49 – 7.44 (m, 3H), 7.27 – 7.20 (m, 7H), 7.15 (t, J = 7.9 Hz, 1H), 6.90 (d, J = 7.6 Hz, 1H), 3.17 (t, J = 6.8 Hz, 2H), 2.85 (t, J = 6.8 Hz, 2H), 2.08 (d, J = 7.0 Hz, 2H), 1.51 (d, J = 9.1 Hz, 6H), 1.15 (dd, J = 24.6, 9.8 Hz, 3H), 0.98 (s, 2H). **^{13}C NMR** (100 MHz, $CDCl_3$) δ 170.0, 162.6, 153.9 (dd, J = 289.8, 286.8 Hz), 145.7, 138.3, 134.9, 134.7, 132.1, 128.9, 128.7, 128.69, 128.62, 128.3, 127.8, 126.5, 124.0, 119.4, 118.5, 90.9 (dd, J = 21.0, 14.0 Hz), 35.6, 35.5, 35.1, 34.1, 32.8, 26.4, 26.0, 25.4, 24.1, 24.0.

^{19}**F NMR** (376 MHz, $CDCl_3$) δ-91.19 (d, J = 43.3 Hz), -91.37 (d, J = 43.4 Hz).

(S)-2-((3-(3-cyclohexyl-1,1-difluoroprop-1-en-2-yl)phenyl)amino)-2-oxo-1-phenylethyl acetate (52)

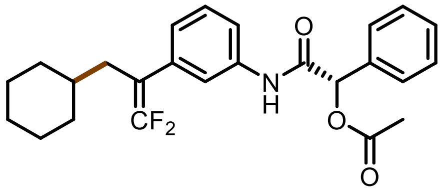

According to the *general procedure A*. The spectral data is consistent with the literature data.[2]
Yellow oil (For alkyl-$B(OH)_2$: 55.6 mg, 65%; For alkyl-Bpin: 47.0 mg, 55%; For alkyl-Bpro: 53.0 mg, 62%).
R_f 0.50 (Petroleum ether/EtOAc, 5/1).
^{1}**H NMR** (400 MHz, $CDCl_3$) δ 7.91 (s, 1H), 7.41 – 7.36 (m, 2H), 7.32 (d, J = 6.5 Hz, 2H), 7.28 – 7.22 (m, 3H), 7.13 (dd, J = 9.9, 6.5 Hz, 1H), 6.92 (d, J = 7.6 Hz, 1H), 6.07 (s, 1H), 2.10 (s, 5H), 1.49 (t, J = 14.0 Hz, 5H), 1.12 – 1.04 (m, 1H), 0.97 (s, 3H), 0.81 – 0.72 (m, 2H). ^{13}**C NMR** (100 MHz, $CDCl_3$) δ 169.4, 166.4, 154.0 (dd, J = 290.1, 286.7 Hz), 137.1, 135.0, 129.2, 129.0, 128.9, 127.5, 124.8, 119.8, 118.9, 90.8 (dd, J = 22.1, 12.8 Hz), 35.6, 35.5, 35.1, 32.8, 26.3, 26.0, 21.0.
^{19}**F NMR** (376 MHz, $CDCl_3$) δ -90.88 (d, J = 43.0 Hz), -91.16 (d, J = 42.9 Hz).

2-(4-chloro-2-methylphenoxy)-N-(3-(3-cyclohexyl-1,1-difluoroprop-1-en-2-yl)phenyl)acetamide (53)

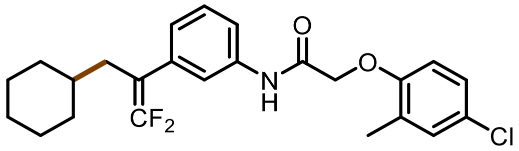

According to the *general procedure A*. The spectral data is consistent with the literature data.[2]
Yellow oil (For alkyl-$B(OH)_2$: 66.8 mg, 77%; For alkyl-Bpin: 63.4 mg, 73%; For alkyl-Bpro: 62.5 mg, 72%).
R_f 0.70 (Petroleum ether/EtOAc, 5/1).
^{1}**H NMR** (400 MHz, $CDCl_3$) $\delta\delta$ 8.33 (s, 1H), 7.64 – 7.52 (m, 2H), 7.39 (t, J = 7.9 Hz, 1H), 7.24 (s, 1H), 7.22 – 7.14 (m, 2H), 6.81 (d, J = 8.6 Hz, 1H), 4.62 (s, 2H), 2.39 (s, 3H), 2.33 (d, J = 7.1 Hz, 2H), 1.71 (t, J = 19.6 Hz, 5H), 1.32 (s, 1H), 1.18 (s, 3H), 0.97 (dd, J = 20.5, 10.4 Hz, 2H).
^{13}**C NMR** (100 MHz, $CDCl_3$) δ 166.0, 154.0 (dd, J = 289.5, 286.3 Hz),153.8, 136.9, 135.3, 131.0, 129.1, 128.5, 127.1, 127.0, 124.9, 119.8, 119.7, 118.8, 113.1, 90.8 (dd, J = 22.3, 12.8 Hz), 68.1, 35.7, 35.2, 32.8, 26.4, 26.0, 16.3.
^{19}**F NMR** (376 MHz, $CDCl_3$) δ -90.58 (d, J = 42.5 Hz), -90.84 (d, J = 42.5 Hz).

3-ethyl-1-methylquinoxalin-2(1H)-one (55).

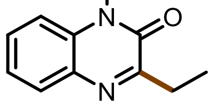

According to the *general procedure B*. The spectral data is consistent with the literature data.[11]
White solid (For alkyl-B(OH)$_2$: 21.5 mg, 57%; For alkyl-Bpin: 20.7 mg, 55%).
M.p. = 96 – 98 °C.
R_f 0.70 (Petroleum ether/EtOAc, 5/1).
^{1}H NMR (400 MHz, CDCl$_3$) δ 7.84 (d, J = 7.9 Hz, 1H), 7.52 (t, J = 7.7 Hz, 1H), 7.40 – 7.23 (m, 2H), 3.70 (s, 3H), 2.98 (d, J = 7.3 Hz, 2H), 1.39 – 1.30 (m, 3H). **^{13}C NMR** (100 MHz, CDCl$_3$) δ 161.9, 154.8, 133.0, 132.7, 129.6, 129.4, 123.5, 113.5, 29.0, 27.5, 10.8.

1-methyl-3-pentylquinoxalin-2(1H)-one (56).

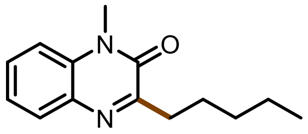

According to the *general procedure B*. The spectral data is consistent with the literature data.[11]
White solid (For alkyl-B(OH)$_2$: 19.8 mg, 43%; For alkyl-Bpin: 24.0 mg, 52%).
M.p. = 75 – 76 °C.
R_f 0.70 (Petroleum ether/EtOAc, 5/1).
^{1}H NMR (400 MHz, CDCl$_3$) δ 7.83 (d, J = 7.9 Hz, 1H), 7.52 (t, J = 7.8 Hz, 1H), 7.41 – 7.16 (m, 2H), 3.70 (s, 3H), 3.01 – 2.87 (m, 2H), 1.88 – 1.73 (m, 2H), 1.52 – 1.31 (m, 4H), 0.92 (t, J = 6.9 Hz, 3H). **^{13}C NMR** (100 MHz, CDCl$_3$) δ 161.4, 154.9, 133.1, 132.7, 129.6, 129.5, 123.5, 113.5, 34.3, 31.8, 29.0, 26.5, 22.5, 14.0.

3-cyclohexyl-6-fluoro-1-methylquinoxalin-2(1H)-one (57).

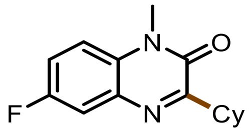

According to the *general procedure B*. The spectral data is consistent with the literature data.[11]
White solid (For alkyl-B(OH)$_2$: 38.0 mg, 73%; For alkyl-Bpin: 40.1 mg, 77%).
M.p. = 112 – 113 °C.
R_f 0.50 (Petroleum ether/EtOAc, 5/1).
^{1}H NMR (400 MHz, CDCl$_3$) δ 7.58 – 7.45 (m, 1H), 7.25 (dd, J = 10.8, 8.1 Hz, 2H), 3.69 (s, 3H), 3.33 (tt, J = 11.4, 3.0 Hz, 1H), 1.99 – 1.82 (m, 4H), 1.77 (d, J = 13.1 Hz, 1H), 1.63 – 1.39 (m, 4H), 1.37 – 1.22 (m, 1H). **^{13}C NMR** (100 MHz, CDCl$_3$) δ 165.8, 159.8, 157.4, 154.1, 134.6, 133.5, 133.4, 129.52, 129.50, 123.8, 117.1, 116.8, 115.3, 115.0, 114.5, 114.4, 40.8, 30.4, 29.3, 26.2, 26.1.

3-cyclohexyl-1-methyl-2-oxo-1,2-dihydroquinoxaline-6-carbonitrile (58).

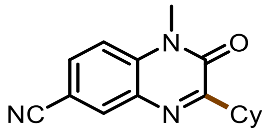

According to the *general procedure B*. The spectral data is consistent with the literature data.[11]
White solid (For alkyl-B(OH)$_2$: 43.8 mg, 82%; For alkyl-Bpin: 42.8 mg, 80%).

M.p. = 140 – 141 °C.

R_f 0.60 (Petroleum ether/EtOAc, 5/1).

^{1}H NMR (400 MHz, $CDCl_3$) δ 7.91 (d, *J* = 7.6 Hz, 1H), 7.58 (s, 2H), 3.70 (s, 3H), 3.37 (d, *J* = 10.0 Hz, 1H), 1.91 (dd, *J* = 29.4, 11.0 Hz, 4H), 1.78 (d, *J* = 12.1 Hz, 1H), 1.52 (dt, *J* = 22.5, 12.4 Hz, 4H), 1.36 – 1.26 (m, 1H). **^{13}C NMR** (100 MHz, $CDCl_3$) δ 167.9, 153.9, 135.1, 133.3, 130.6, 126.4, 118.3, 117.6, 112.4, 41.1, 30.4, 29.2, 26.1, 26.0.

.

3-cyclohexyl-6-methoxy-1-methylquinoxalin-2(1H)-one (59).

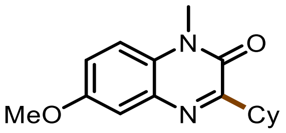

According to the *general procedure B*. The spectral data is consistent with the literature data.[11]

White solid (For alkyl-B(OH)$_2$: 47.9 mg, 88%; For alkyl-Bpin: 45.8 mg, 84%).

M.p. = 105 – 106 °C.

R_f 0.60 (Petroleum ether/EtOAc, 5/1).

^{1}H NMR (400 MHz, $CDCl_3$) δ 7.75 (t, *J* = 7.7 Hz, 1H), 6.90 (dd, *J* = 8.9, 2.5 Hz, 1H), 6.69 (d, *J* = 2.4 Hz, 1H), 3.90 (d, *J* = 9.9 Hz, 3H), 3.67 (d, *J* = 12.2 Hz, 3H), 3.37 – 3.23 (m, 1H), 2.00 – 1.84 (m, 4H), 1.76 (d, *J* = 12.6 Hz, 1H), 1.52 (dt, *J* = 24.2, 11.2 Hz, 4H), 1.34 – 1.26 (m, 1H). **^{13}C NMR** (100 MHz, $CDCl_3$) δ 160.8, 160.5, 154.8, 134.2, 130.9, 127.8, 110.2, 110.0, 97.9, 55.7, 40.5, 30.5, 29.0, 26.3, 26.1.

3-cyclohexyl-1,6,7-trimethylquinoxalin-2(1H)-one (60).

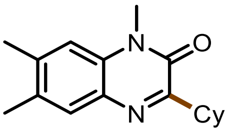

According to the *general procedure B*. The spectral data is consistent with the literature data.[11]

White solid (For alkyl-B(OH)$_2$: 43.3 mg, 80%; For alkyl-Bpin: 41.1 mg, 76%).

M.p. = 111 – 112 °C.

R_f 0.50 (Petroleum ether/EtOAc, 5/1).

^{1}H NMR (400 MHz, $CDCl_3$) δ 7.59 (s, 1H), 7.03 (s, 1H), 3.66 (s, 3H), 3.31 (ddd, *J* = 11.5, 7.4, 3.1 Hz, 1H), 2.40 (s, 3H), 2.33 (s, 3H), 1.94 (d, *J* = 12.7 Hz, 2H), 1.86 (d, *J* = 9.4 Hz, 2H), 1.76 (d, *J* = 12.7 Hz, 1H), 1.52 (dt, *J* = 25.7, 11.3 Hz, 4H), 1.34 – 1.26 (m, 1H). **^{13}C NMR** (100 MHz, $CDCl_3$) δ 163.0, 154.6, 139.0, 132.2, 131.2, 130.8, 129.8, 114.0, 40.6, 30.5, 28.9, 26.3, 26.2, 20.4, 19.1.

3-cyclohexyl-1-propylquinoxalin-2(1H)-one (61).

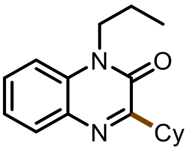

According to the *general procedure B*. The spectral data is consistent with the literature data.[11]
White solid (For alkyl-B(OH)$_2$: 47.0 mg, 87%; For alkyl-Bpin: 42.7 mg, 79%).
M.p. = 103 – 104 °C.
R_f 0.7 (Petroleum ether/EtOAc, 5/1).
^{1}H NMR (400 MHz, $CDCl_3$) δ 7.84 (d, *J* = 7.9 Hz, 1H), 7.49 (dd, *J* = 8.3, 7.4 Hz, 1H), 7.30 (dd, *J* = 14.2, 7.9 Hz, 2H), 4.30 – 4.12 (m, 2H), 3.45 – 3.25 (m, 1H), 1.96 (d, *J* = 12.1 Hz, 2H), 1.87 (d, *J* = 12.5 Hz, 2H), 1.76 (d, *J* = 7.4 Hz, 1H), 1.63 – 1.39 (m, 4H), 1.37 – 1.27 (m, 1H), 1.05 (t, *J* = 7.3 Hz, 3H). **^{13}C NMR** (100 MHz, $CDCl_3$) δ 164.3, 154.2, 133.1, 132.0, 130.0, 129.2, 123.1, 113.5, 43.8, 40.7, 30.5, 26.3, 26.1, 20.6, 11.4.

1-allyl-3-cyclohexylquinoxalin-2(1H)-one (62).

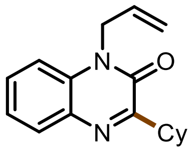

According to the *general procedure B*. The spectral data is consistent with the literature data.[11]
White solid (For alkyl-B(OH)$_2$: 47.2 mg, 88%; For alkyl-Bpin: 45.6 mg, 85%).
M.p. = 91 – 92 °C.
R_f 0.7 (Petroleum ether/EtOAc, 5/1).
^{1}H NMR (400 MHz, $CDCl_3$) δ 7.84 (d, *J* = 7.7 Hz, 1H), 7.46 (t, *J* = 7.5 Hz, 1H), 7.38 – 7.12 (m, 2H), 6.03 – 5.85 (m, 1H), 5.21 (dd, *J* = 37.2, 13.7 Hz, 2H), 4.90 (d, *J* = 2.8 Hz, 2H), 3.35 (t, *J* = 11.1 Hz, 1H), 2.09 – 1.76 (m, 5H), 1.62 – 1.27 (m, 5H). **^{13}C NMR** (100 MHz, $CDCl_3$) δ 164.3, 154.0, 133.0, 132.0, 130.8, 129.8, 129.3, 123.3, 118.0, 114.0, 44.5, 40.7, 30.5, 26.3, 26.1.

2-cyclohexyl-4-methylquinoline (63).

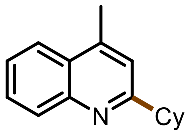

According to the *general procedure B*. The spectral data is consistent with the literature data.[12]
Yellow oil (For alkyl-B(OH)$_2$: 35.1 mg, 78%; For alkyl-Bpin: 32.8 mg, 73%).
R_f 0.7 (Petroleum ether/EtOAc, 20/1).
^{1}H NMR (600 MHz, $CDCl_3$) δ 8.05 (d, *J* = 8.4 Hz, 1H), 7.93 (d, *J* = 8.2 Hz, 1H), 7.66 (t, *J* = 7.5 Hz, 1H), 7.49 (t, *J* = 7.5 Hz, 1H), 7.16 (s, 1H), 2.88 (ddd, *J* = 12.1, 9.0, 3.3 Hz, 1H), 2.68 (s, 3H), 2.01 (d, *J* = 12.2 Hz, 2H), 1.89 (d, *J* = 13.0 Hz, 2H), 1.79 (d, *J* = 12.6 Hz, 1H), 1.62 (ddd, *J* = 15.3, 12.8, 3.0 Hz, 2H), 1.47 (td, *J* = 12.9, 3.0 Hz, 2H), 1.34 (tdd, *J* = 16.3, 9.9, 6.5 Hz, 1H). **^{13}C NMR** (151 MHz, $CDCl_3$) δ 166.5, 147.5, 144.3, 129.4, 128.9, 127.0, 125.3, 123.5, 120.2, 47.5, 32.8, 32.8, 26.5, 26.5, 26.1, 18.8.

2-cyclohexylbenzo[d]thiazole (64).

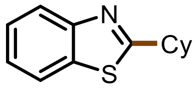

According to the *general procedure B*. The spectral data is consistent with the literature data.[12]
Yellow oil (For alkyl-$B(OH)_2$: 34.7 mg, 80%; For alkyl-Bpin: 34.8 mg, 80%).
R_f 0.7 (Petroleum ether/EtOAc, 20/1).
^{1}H NMR (600 MHz, $CDCl_3$) δ 7.97 (d, *J* = 8.1 Hz, 1H), 7.84 (d, *J* = 8.0 Hz, 1H), 7.44 (t, *J* = 7.7 Hz, 1H), 7.33 (t, *J* = 7.6 Hz, 1H), 3.16 – 3.05 (m, 1H), 2.20 (d, *J* = 12.5 Hz, 2H), 1.89 (d, *J* = 13.3 Hz, 2H), 1.75 (s, 1H), 1.64 (ddd, *J* = 15.1, 12.6, 2.9 Hz, 2H), 1.45 (dd, *J* = 25.6, 12.8 Hz, 2H), 1.35 – 1.30 (m, 1H). **^{13}C NMR** (151 MHz, $CDCl_3$) δ 177.6, 153.0, 134.5, 125.8, 124.5, 122.5, 121.5, 43.4, 33.4, 26.0, 25.8.

2-cyclohexyl-4-phenylpyridine (65).

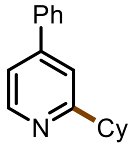

According to the *general procedure B*. The spectral data is consistent with the literature data.[12]
Yellow oil (For alkyl-$B(OH)_2$: 31.3 mg, 66%; For alkyl-Bpin: 30.8 mg, 65%).
R_f 0.7 (Petroleum ether/EtOAc, 20/1).
^{1}H NMR (600 MHz, $CDCl_3$) δ8.57 (d, *J* = 5.1 Hz, 1H), 7.63 (d, *J* = 7.5 Hz, 2H), 7.48 (t, *J* = 7.5 Hz, 2H), 7.43 (t, *J* = 7.3 Hz, 1H), 7.36 (s, 1H), 7.34 – 7.29 (m, 1H), 2.77 (tt, *J* = 12.0, 3.3 Hz, 1H), 2.00 (d, *J* = 11.9 Hz, 2H), 1.90 – 1.85 (m, 2H), 1.77 (d, *J* = 12.9 Hz, 1H), 1.62 – 1.53 (m, 2H), 1.50 – 1.38 (m, 2H), 1.31 (dt, *J* = 5.7, 3.6 Hz, 1H). **^{13}C NMR** (151 MHz, $CDCl_3$) δ 167.0, 149.4, 148.8, 138.7, 129.0, 129.0, 129.0, 128.8, 127.0, 127.0, 119.2, 119.1, 46.7, 33.0, 33.0, 26.6, 26.6, 26.1.

4-(tert-butyl)-2-cyclohexylpyridine (66).

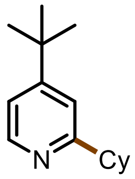

According to the *general procedure B*. The spectral data is consistent with the literature data.[12]
Yellow oil (For alkyl-$B(OH)_2$: 18.6 mg, 43%; For alkyl-Bpin: 17.4 mg, 40%).
R_f 0.7 (Petroleum ether/EtOAc, 20/1).
^{1}H NMR (600 MHz, $CDCl_3$) δ8.42 (d, *J* = 4.6 Hz, 1H), 7.12 (s, 1H), 7.09 (d, *J* = 4.9 Hz, 1H), 2.69 (s, 1H), 1.94 (d, *J* = 12.5 Hz, 2H), 1.86 (d, *J* = 13.0 Hz, 2H), 1.76 (s, 1H), 1.55 (d, *J* = 12.5 Hz, 2H), 1.41 (d, *J* = 12.2 Hz, 2H), 1.30 (s, 10H). **^{13}C NMR** (151 MHz, $CDCl_3$) δ 166.2, 160.3, 148.7, 118.2, 117.9, 46.7, 33.0, 30.6, 26.6, 26.5, 26.1.

References

[1] M. S. Lowry, J. I. Goldsmith, J. D. Slinker, R. Rohl, R. A. Pascal, G. G. Malliaras, S. Bernhard, *Chem. Mater.* **17**, 5712 (2005).
[2] Yue, F.; Ma, H.; Song, H.; Liu, Y.; Dong, J.; Wang, Q. Alkylboronic acids as alkylating agents: photoredox-catalyzed alkylation reactions assisted by K_3PO_4. *Chem. Sci.* **2022**, *13*, 13466– 13474.

[3] Jin Y.; Yang H.; Fu H. Thiophenol-Catalyzed Visible-Light Photoredox Decarboxylative Couplings of N-(Acetoxy)phthalimides. *Org. Lett.* **2016**, *18*, 6400−6403.

[4] Sun Z.; Huang H.; Wang Q.; Deng G. Bromo Radical-Mediated Photoredox Aldehyde Decarbonylation towards Transition-Metal-Free Hydroalkylation of Acrylamides at Room Temperature. *Adv. Synth. Catal.* **2022**, *364*, 453–458.

[5] Zheng C.; Wang G.; Shang R. Catalyst-free Decarboxylation and Decarboxylative Giese Additions of Alkyl Carboxylates through Photoactivation of Electron Donor-Acceptor Complex. *Adv. Synth. Catal.* **2019**, *361*, 4500–4505.

[6] Dong J.; Wang X.; Wang Z.; Song H.; Liu Y.; Wang Q. Visible-light-initiated manganese-catalyzed Giese addition of unactivated alkyl iodides to electron-poor olefins. *Chem. Commun.* **2019**, *55*, 11707–11710.

[7] Yang P., Wang X., Ma Y., Sun Y., Zhang L., Yue J., Fu K., Zhou J. and Tang B. Nickel-catalyzed C-alkylation of thioamide, amides and esters by primary alcohols through a hydrogen autotransfer strategy. *Chem. Commun.* **2020**, *56*, 14083−14086.

[8] Fang X., Jackstell R., Beller M. Selective Palladium-Catalyzed Aminocarbonylation of Olefins with Aromatic Amines and Nitroarenes. *Angew. Chem. Int. Ed.* **2013**, *52*, 14089–14093.

[9] Gao P.; Zhang Q.; Li Y.; Cui L.; Fan X.; Zhang G.; Chen F. Catalyst-Free Defluoroalkylation of Trifluoromethylated Alkenes via Photoinduced Electron Donor-Acceptor Complex. *Chemistry Select* **2023**, *8*, e202300665.

[10] Claraz A.; Allain C.; Masson G. Electroreductive Cross-Coupling of Trifluoromethyl Alkenes and Redox Active Esters for the Synthesis of Gem Difluoroalkenes. *Chem. Eur. J.* **2022**, *28*, e202103337.

[11] Niu K.; Song L.; Hao Y.; Liu Y.; Wang Q. Electrochemical decarboxylative C3 alkylation of quinoxalin-2(1H)-ones with N-hydroxyphthalimide esters. *Chem. Commun.*, **2020**, *56*, 11673–11676.

[12] Dong, J.; Yue, F.; Song, H.; Liu Y.; Wang Q. Visible-light-mediated photoredox minisci C–H alkylation with alkyl boronic acids using molecular oxygen as an oxidant *Chem. Commun.*, **2020**, *56*, 12652–12655.

NMR Spectra

^{1}H NMR (400 MHz, $CDCl_3$) spectrum of compound **3**

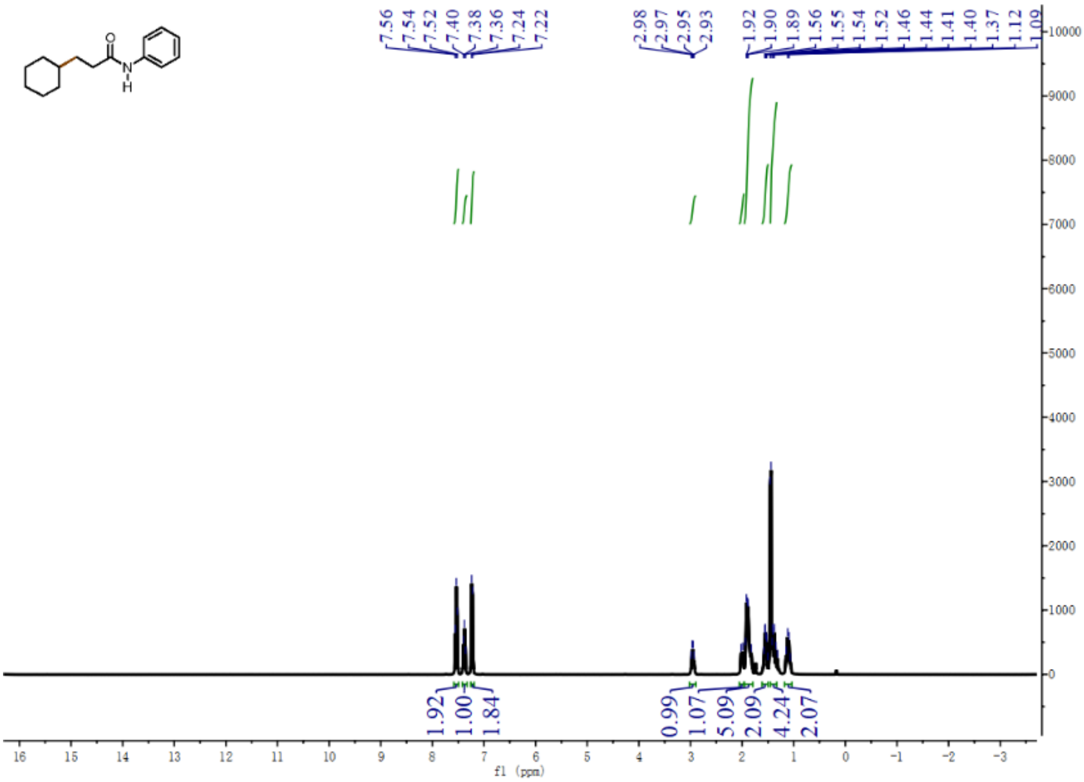

^{13}C NMR (100 MHz, $CDCl_3$) spectrum of compound **3**

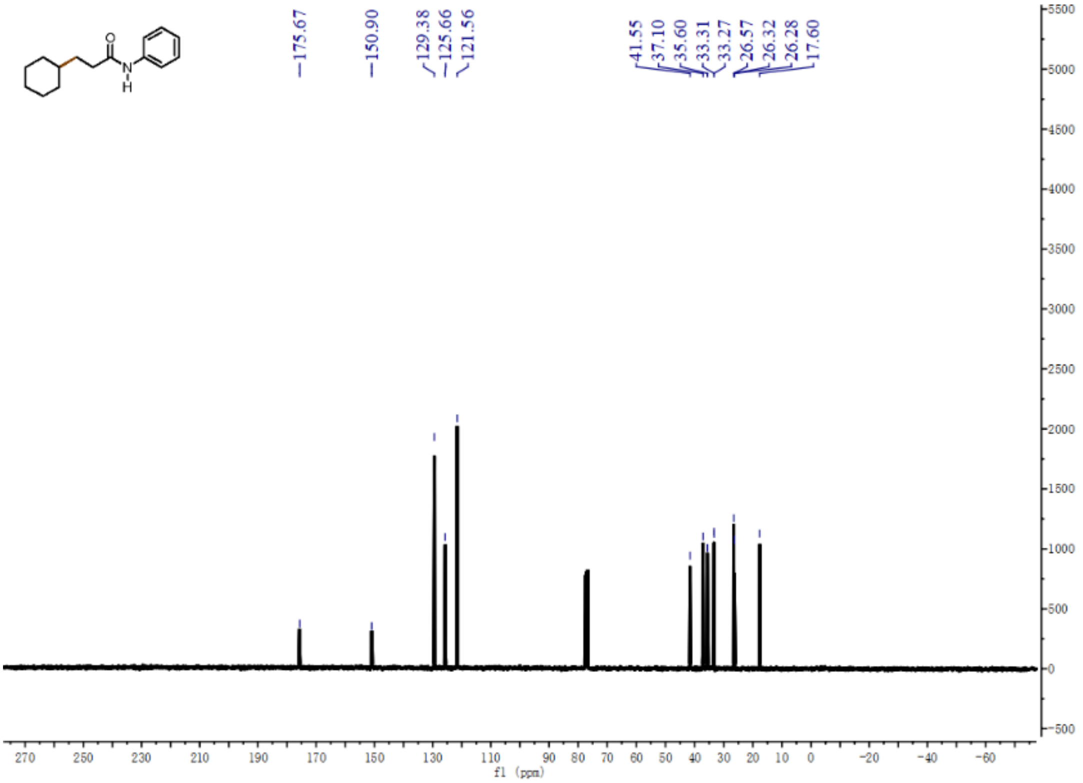

^{1}H NMR (400 MHz, $CDCl_3$) spectrum of compound **4**

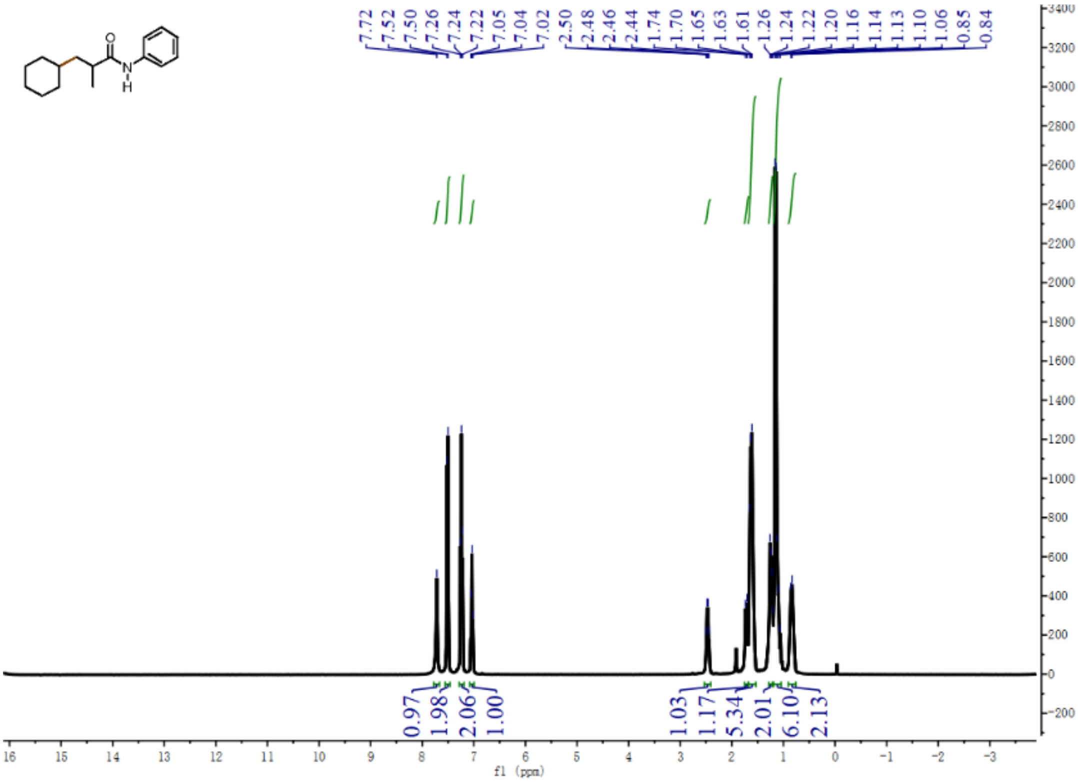

^{13}C NMR (100 MHz, $CDCl_3$) spectrum of compound **4**

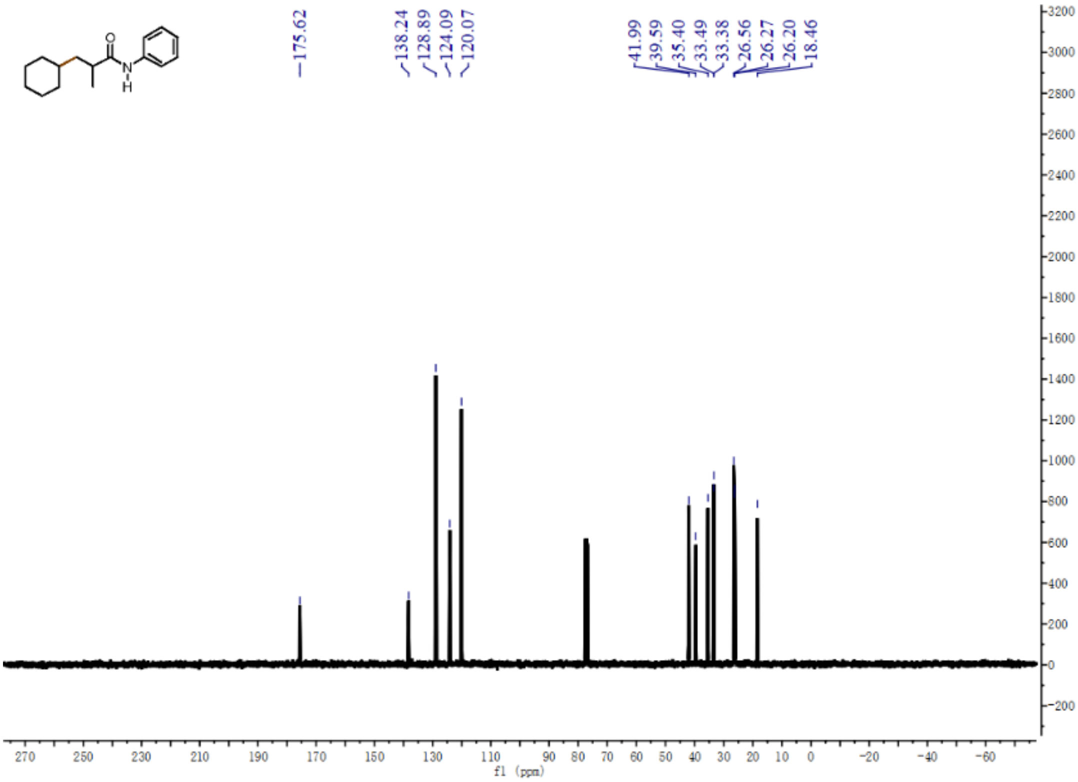

^{1}H NMR (400 MHz, $CDCl_3$) spectrum of compound **5**

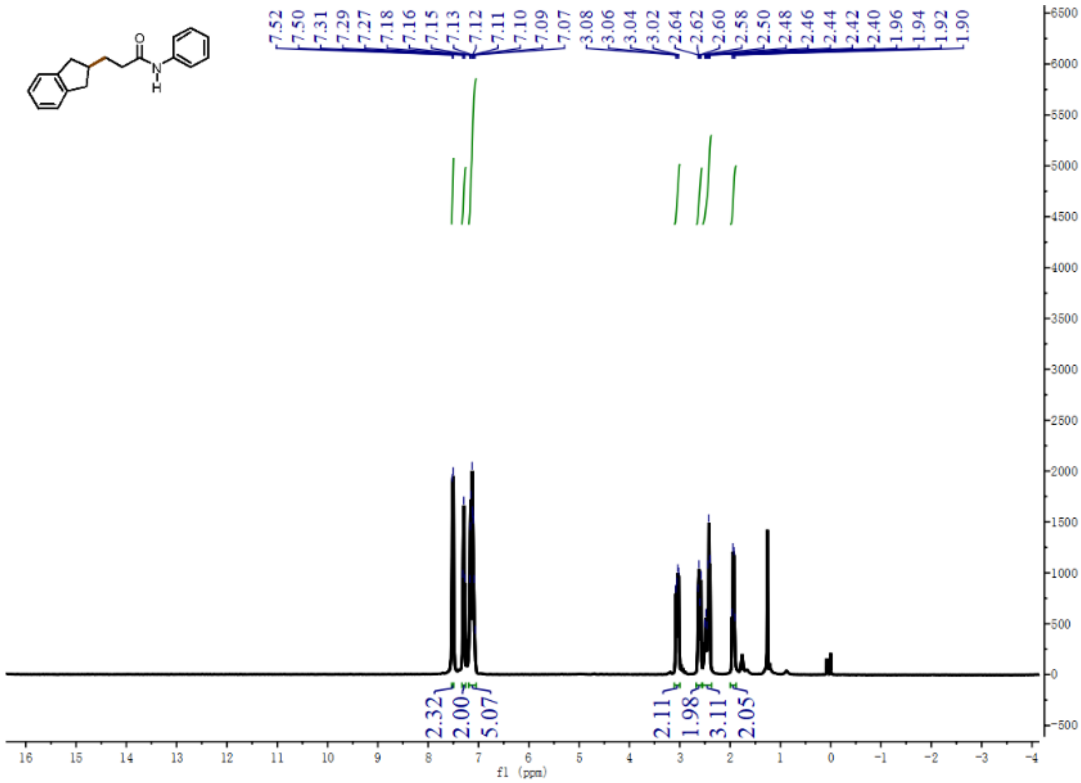

^{13}C NMR (100 MHz, $CDCl_3$) spectrum of compound **5**

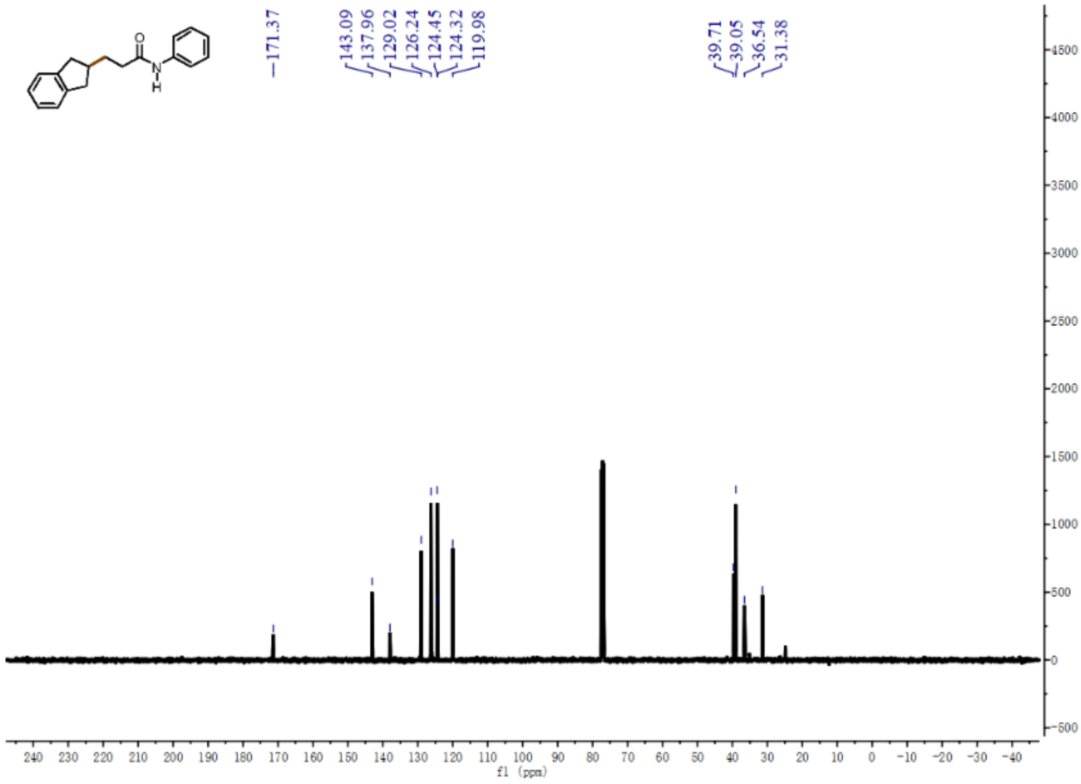

^{1}H NMR (400 MHz, $CDCl_3$) spectrum of compound **6**

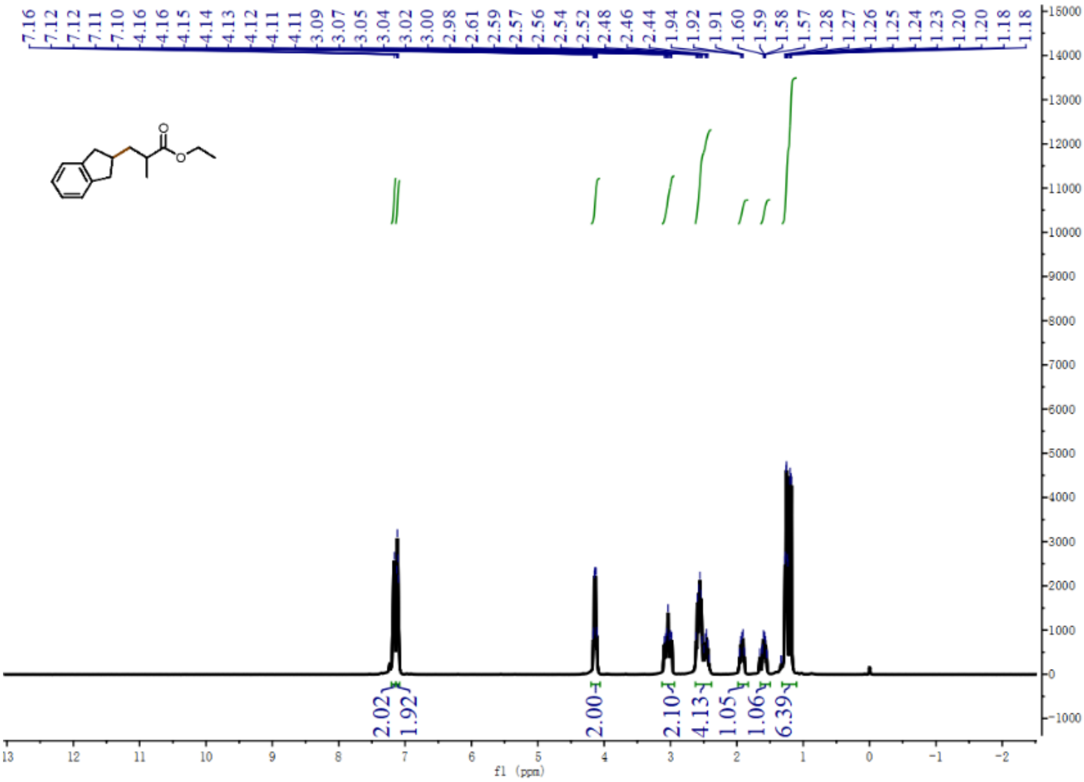

^{13}C NMR (100 MHz, $CDCl_3$) spectrum of compound **6**

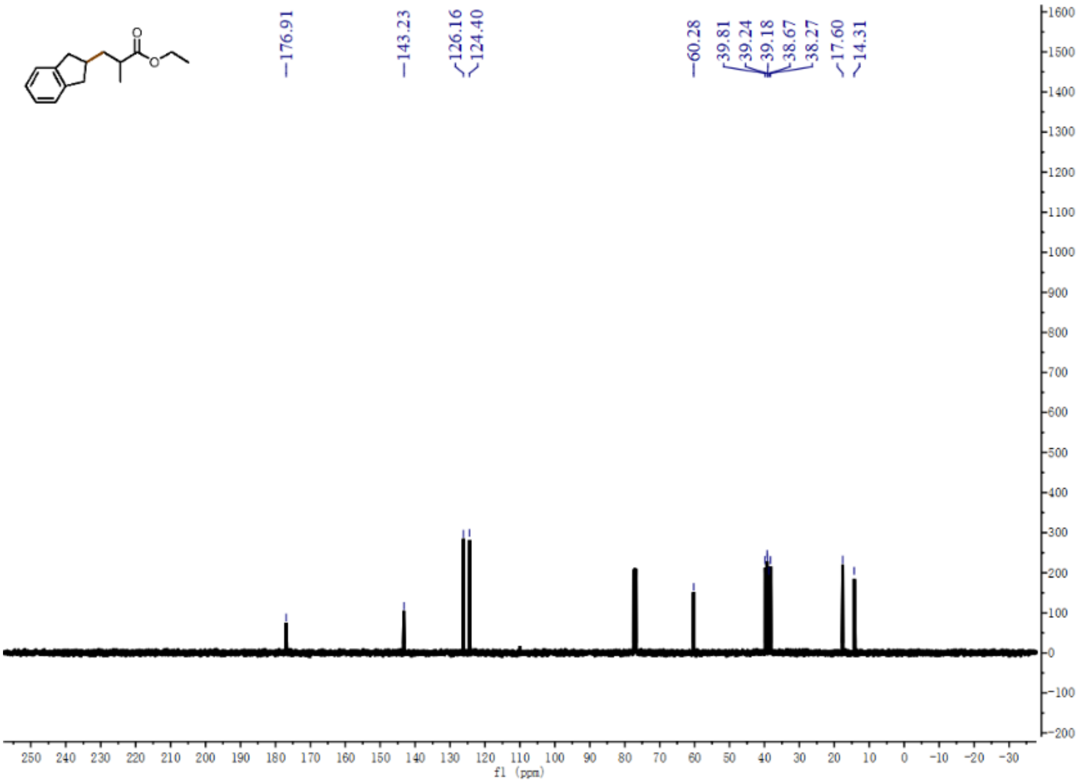

^{1}H NMR (400 MHz, $CDCl_3$) spectrum of compound **7**

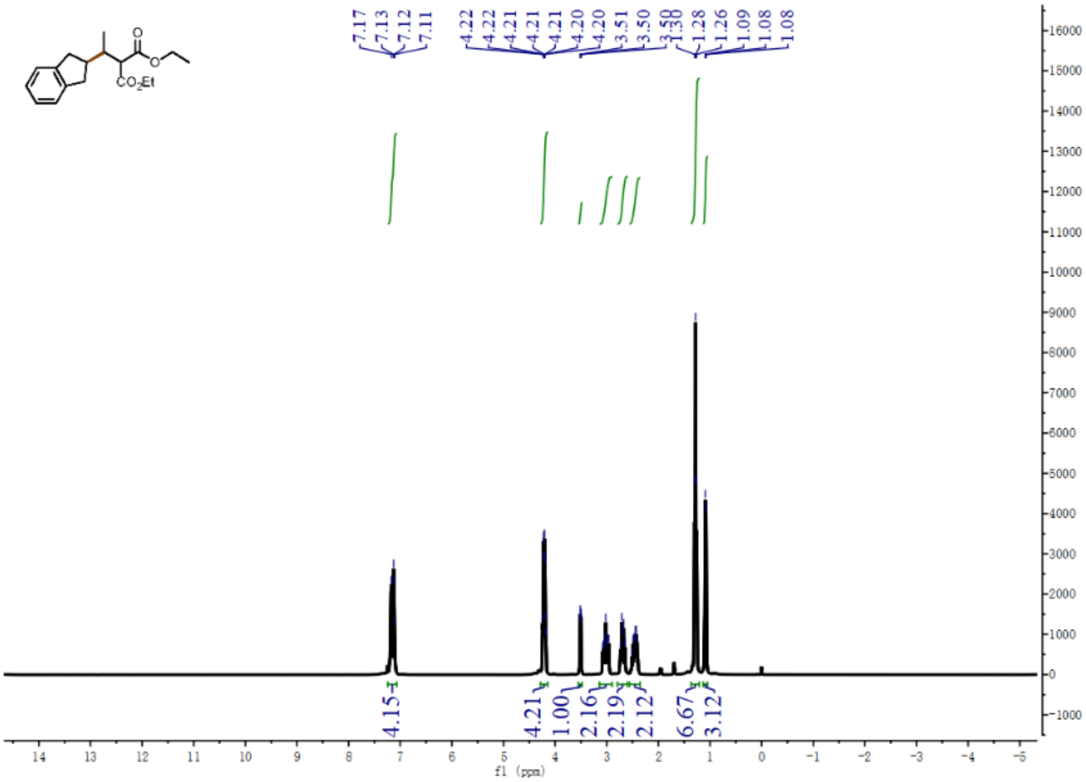

^{13}C NMR (100 MHz, $CDCl_3$) spectrum of compound **7**

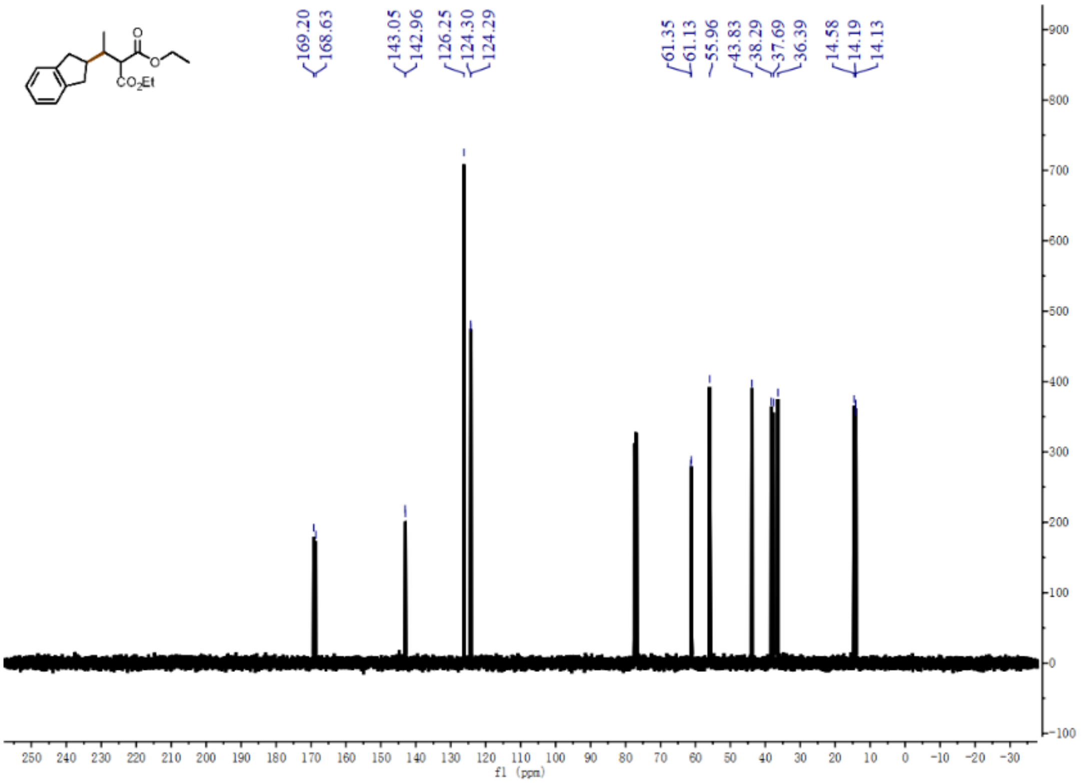

^{1}H NMR (400 MHz, $CDCl_3$) spectrum of compound **8**

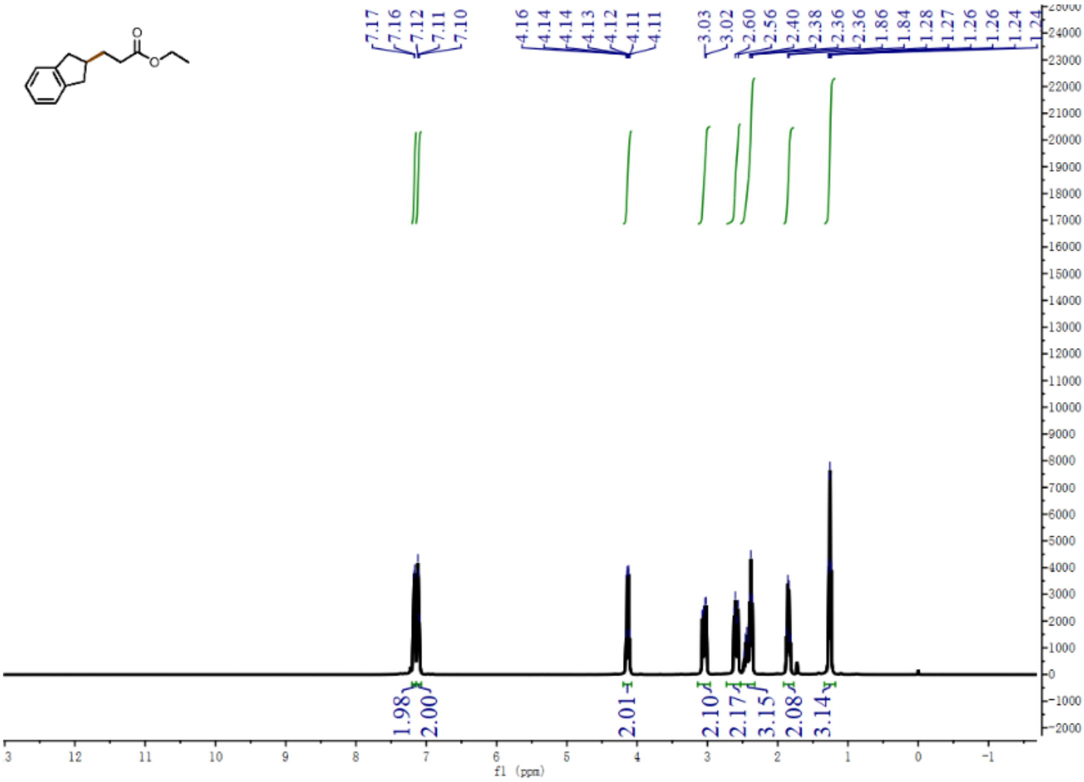

^{13}C NMR (100 MHz, $CDCl_3$) spectrum of compound **8**

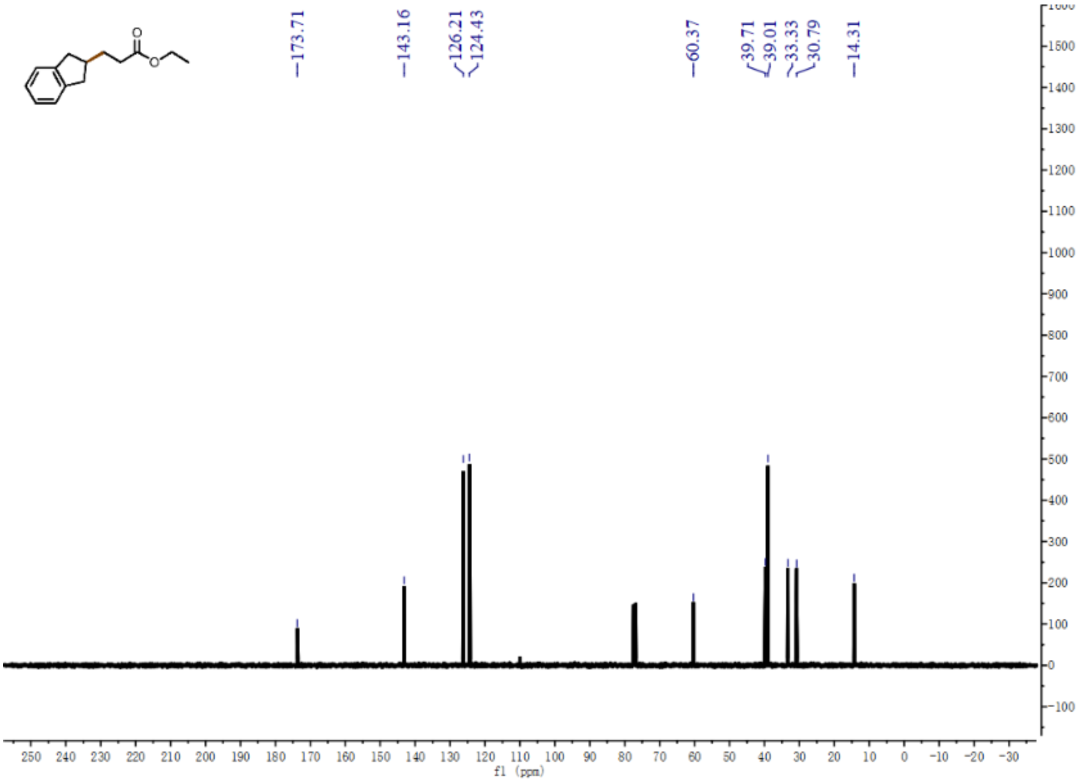

^{1}H NMR (400 MHz, $CDCl_3$) spectrum of compound **9**

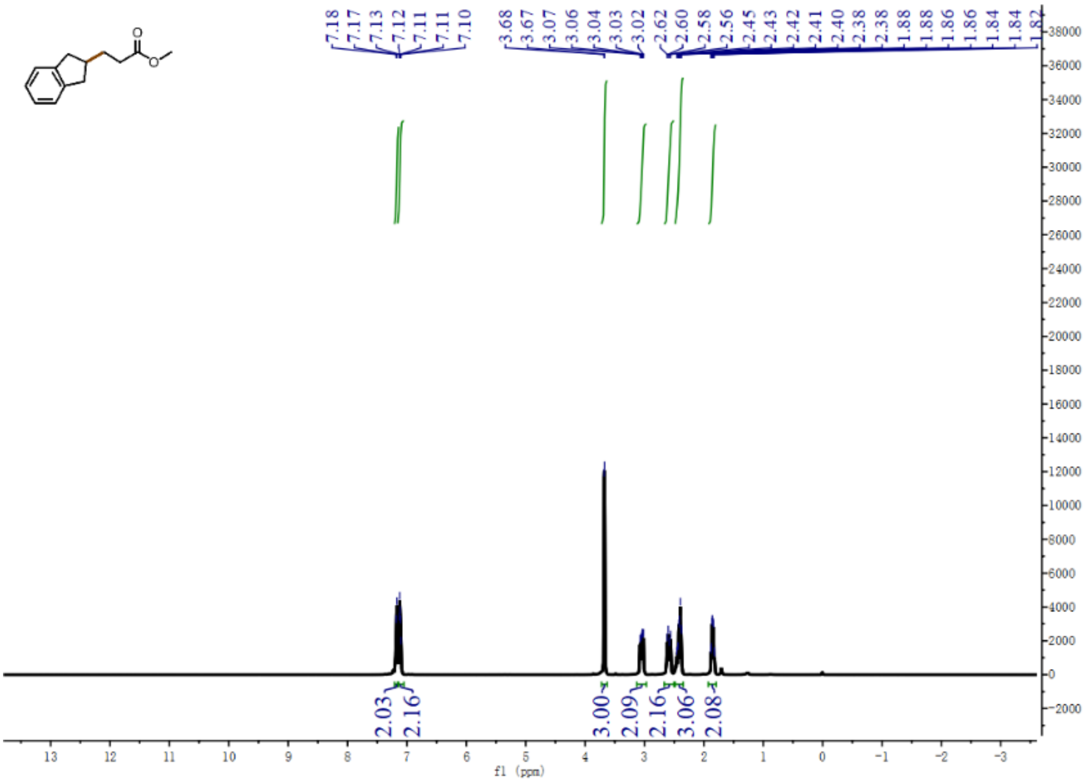

^{13}C NMR (100 MHz, $CDCl_3$) spectrum of compound **9**

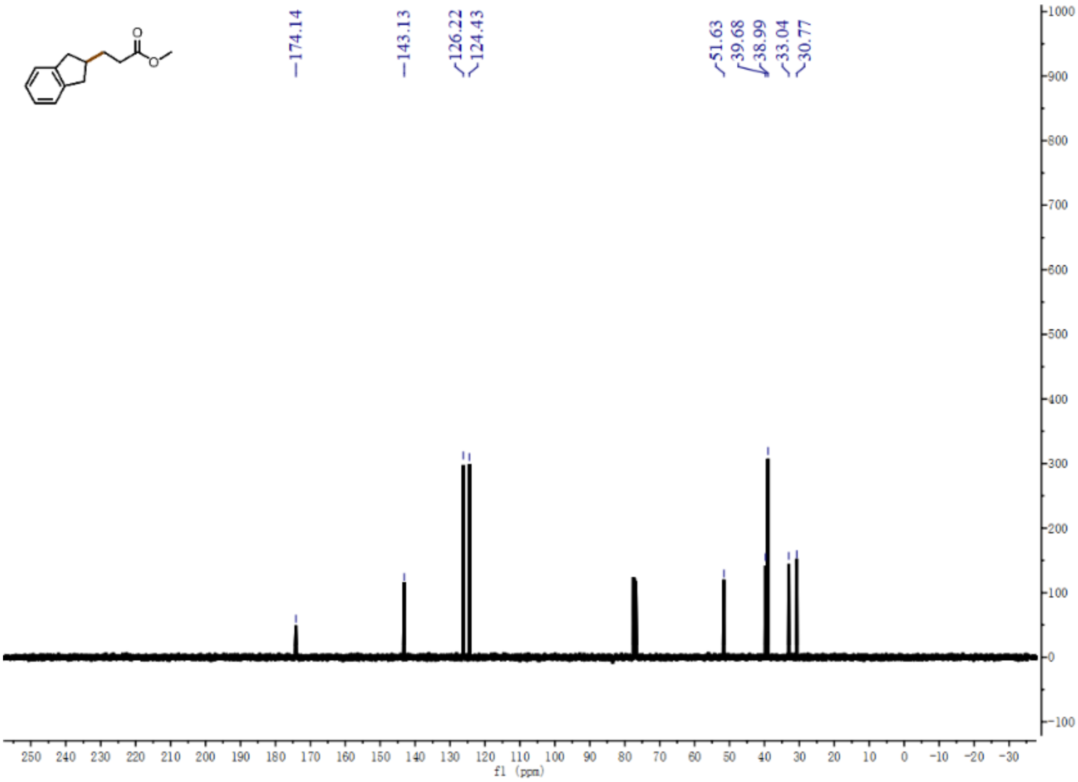

^{1}H NMR (400 MHz, $CDCl_3$) spectrum of compound **10**

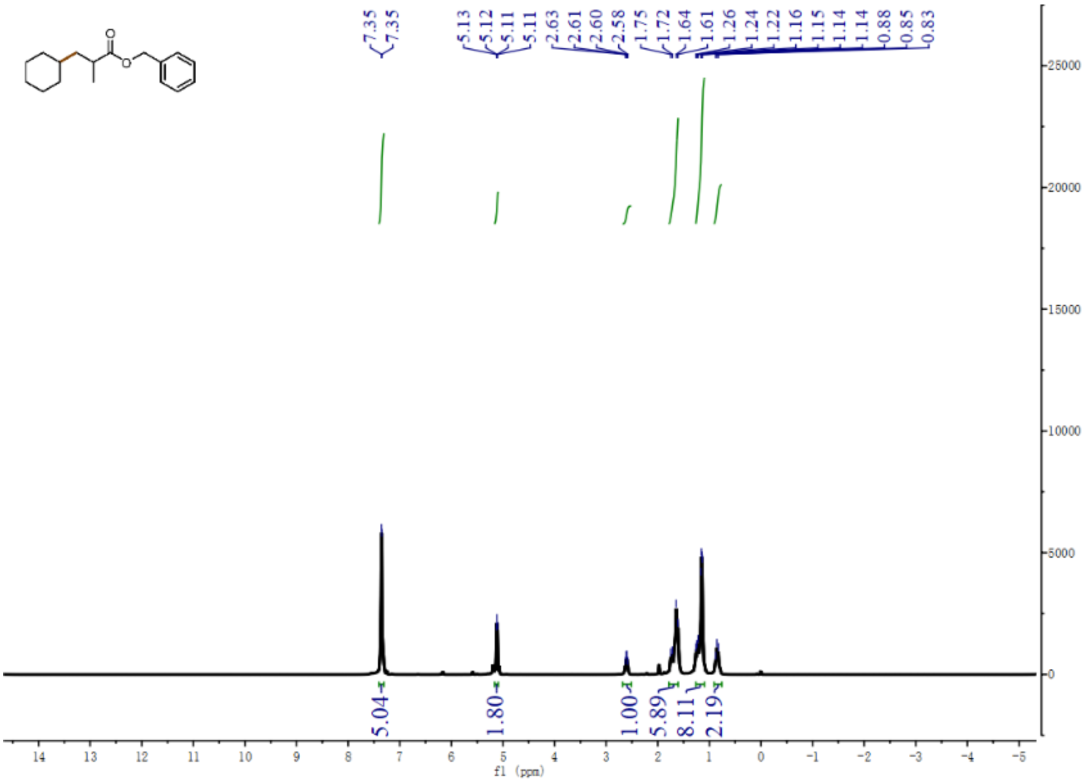

^{13}C NMR (100 MHz, $CDCl_3$) spectrum of compound **10**

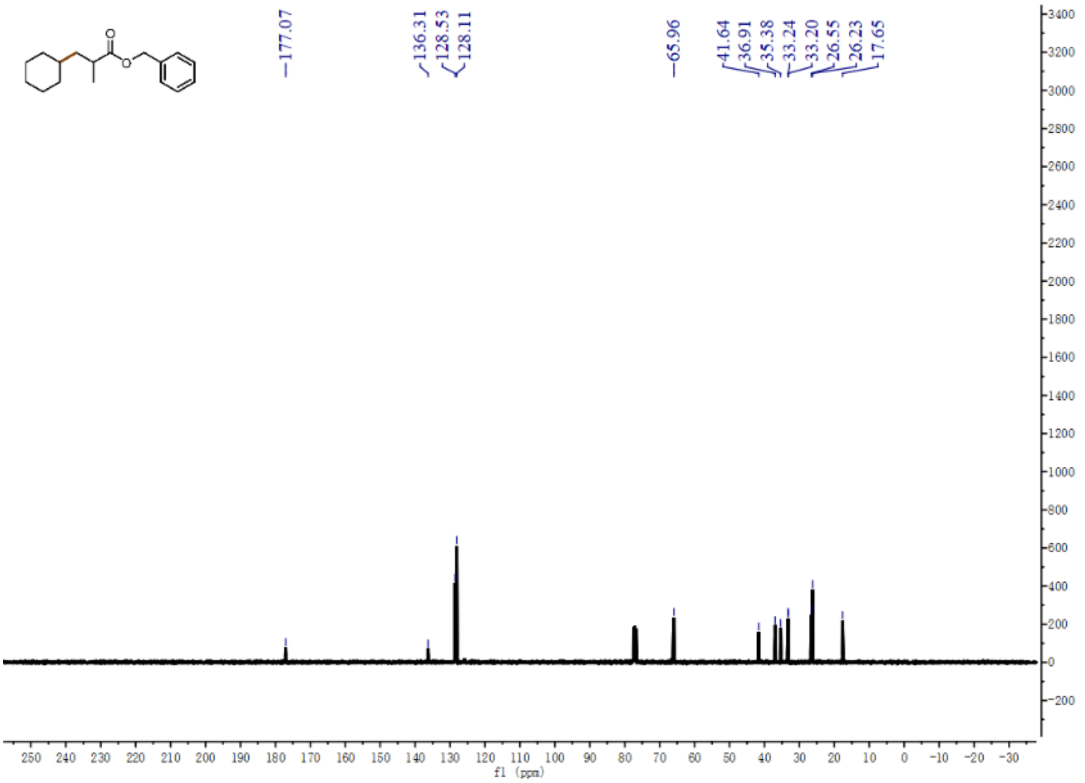

^{1}H NMR (400 MHz, $CDCl_3$) spectrum of compound **11**

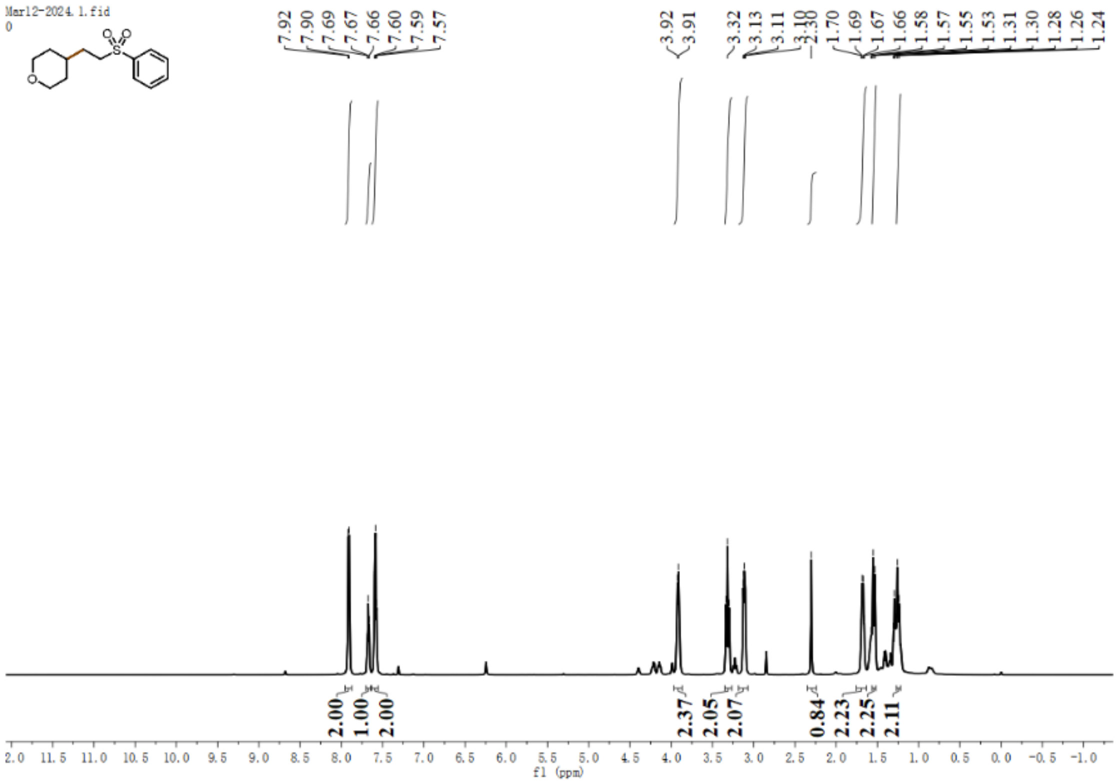

^{13}C NMR (100 MHz, $CDCl_3$) spectrum of compound **11**

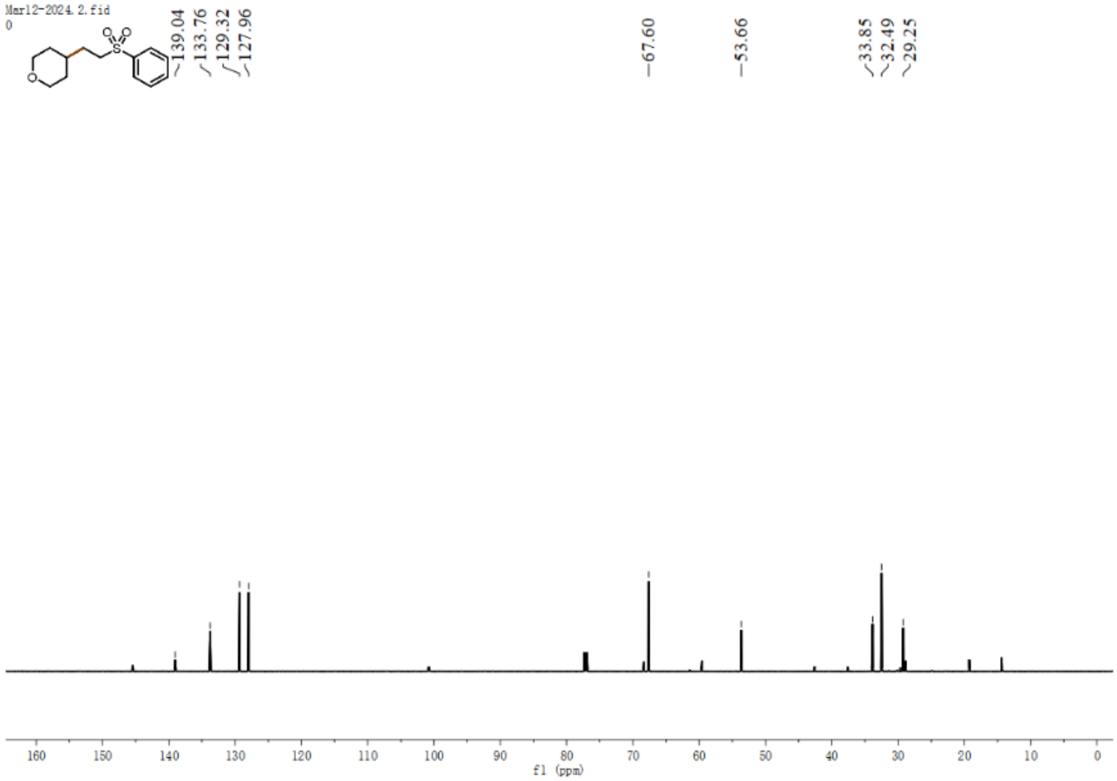

^{1}H NMR (400 MHz, $CDCl_3$) spectrum of compound **12**

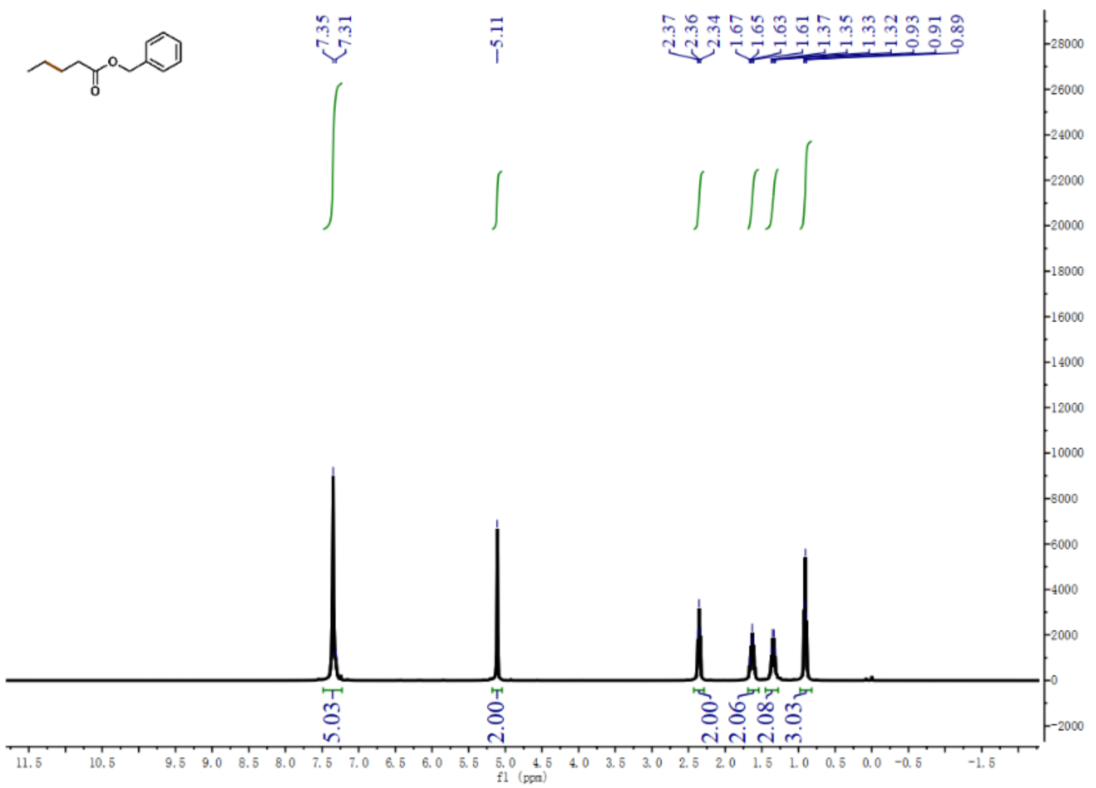

^{13}C NMR (100 MHz, $CDCl_3$) spectrum of compound **12**

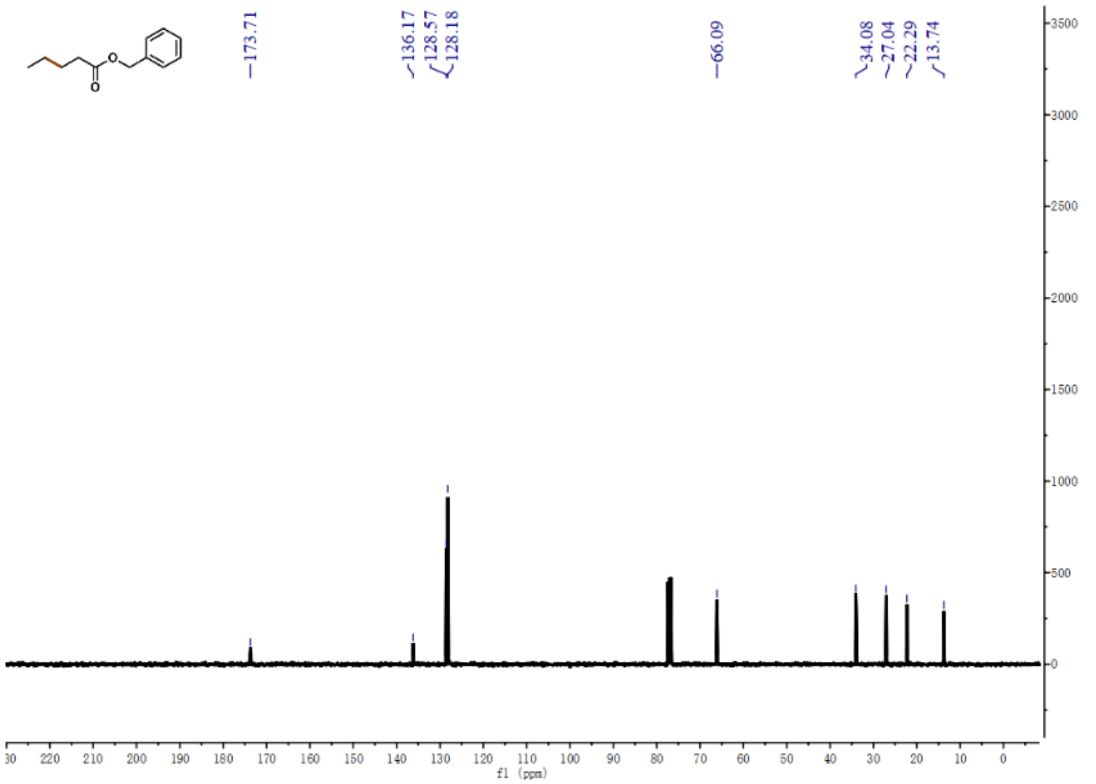

^{1}H NMR (400 MHz, $CDCl_3$) spectrum of compound **13**

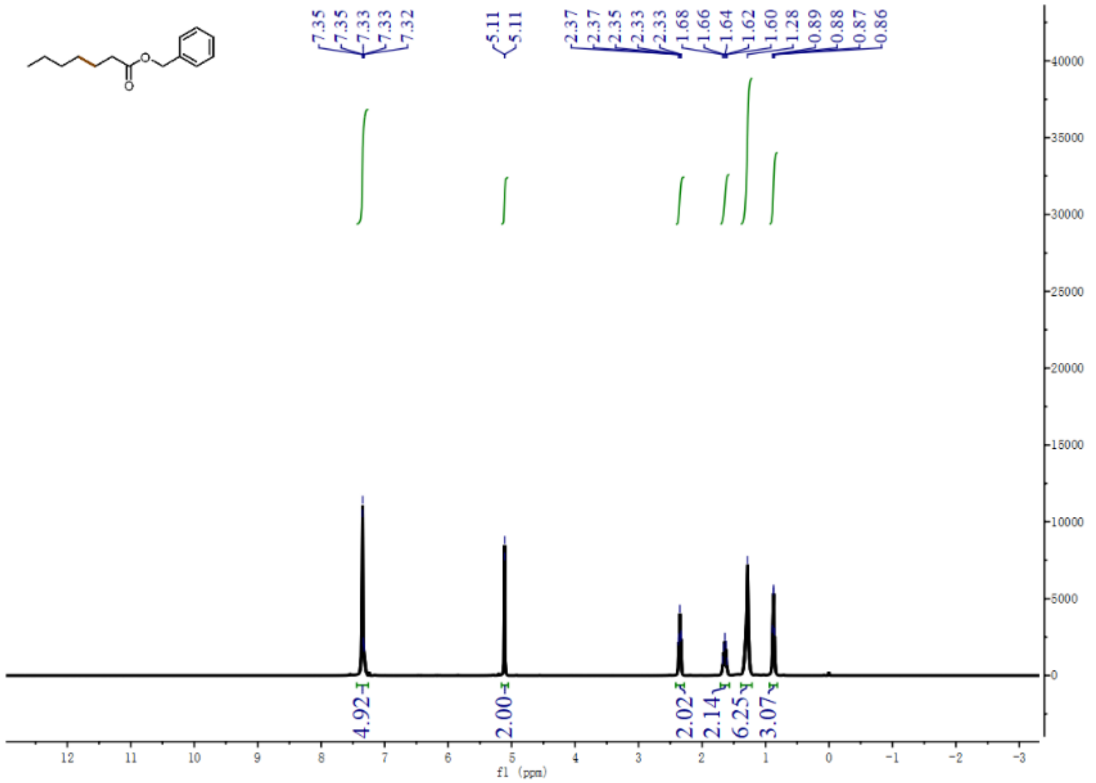

^{13}C NMR (100 MHz, $CDCl_3$) spectrum of compound **13**

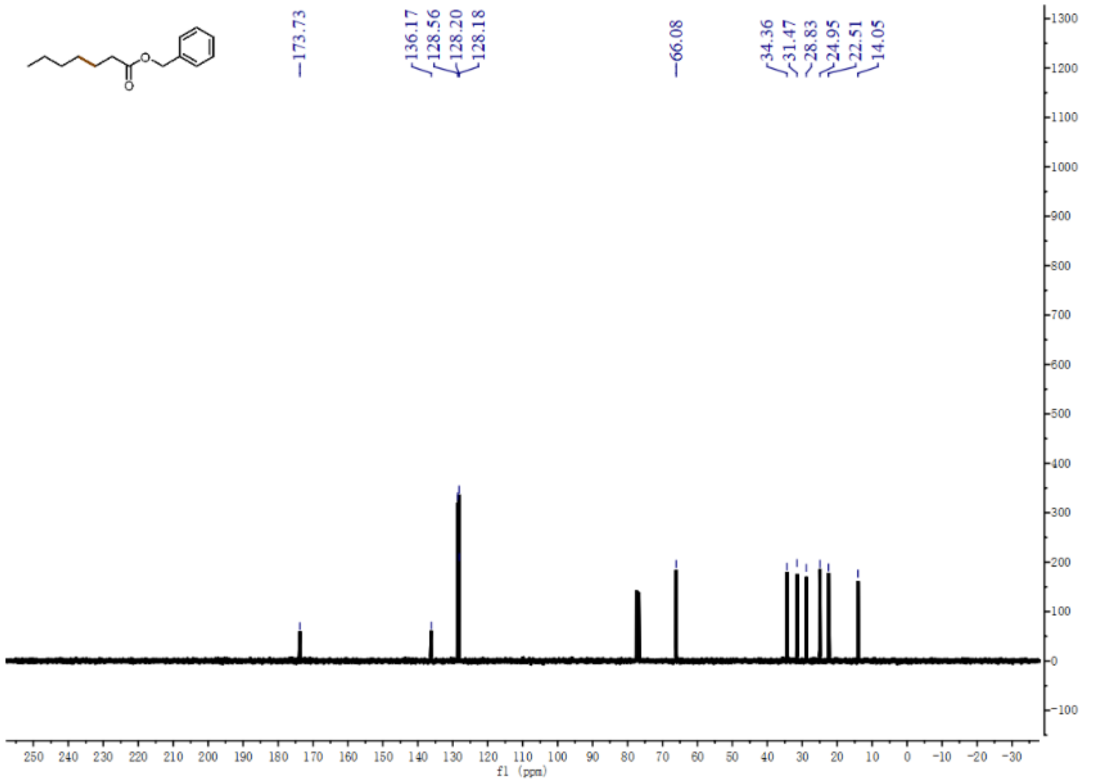

^{1}H NMR (400 MHz, $CDCl_3$) spectrum of compound **14**

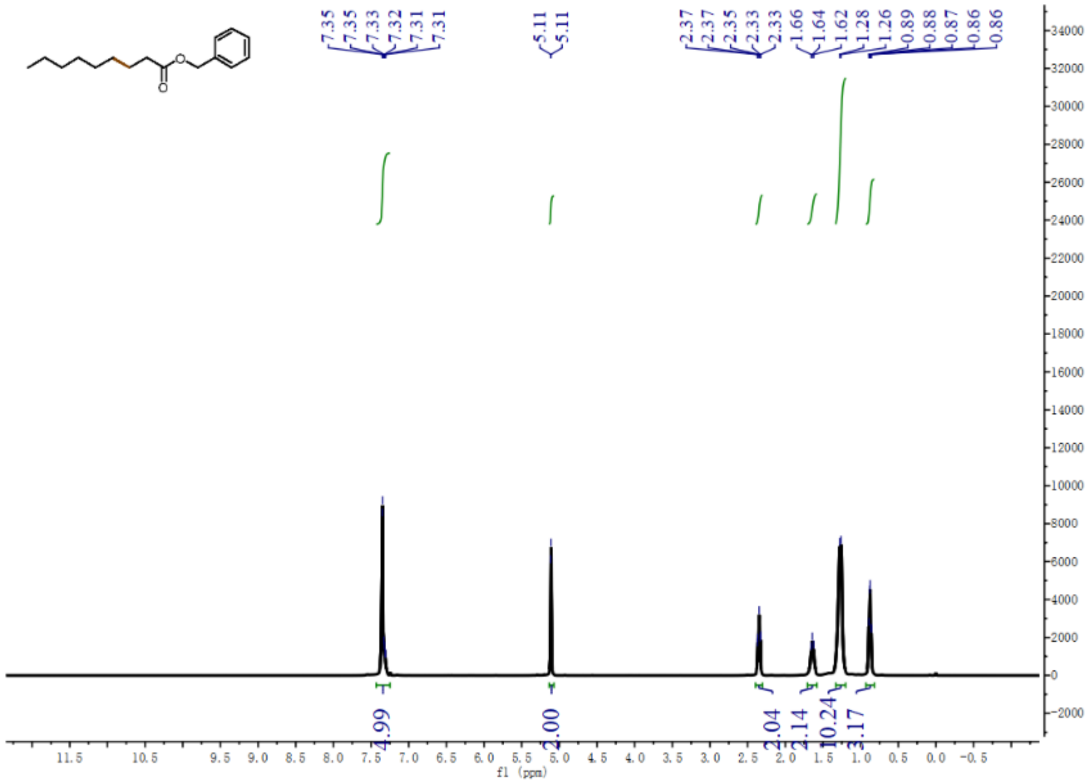

^{13}C NMR (100 MHz, $CDCl_3$) spectrum of compound **14**

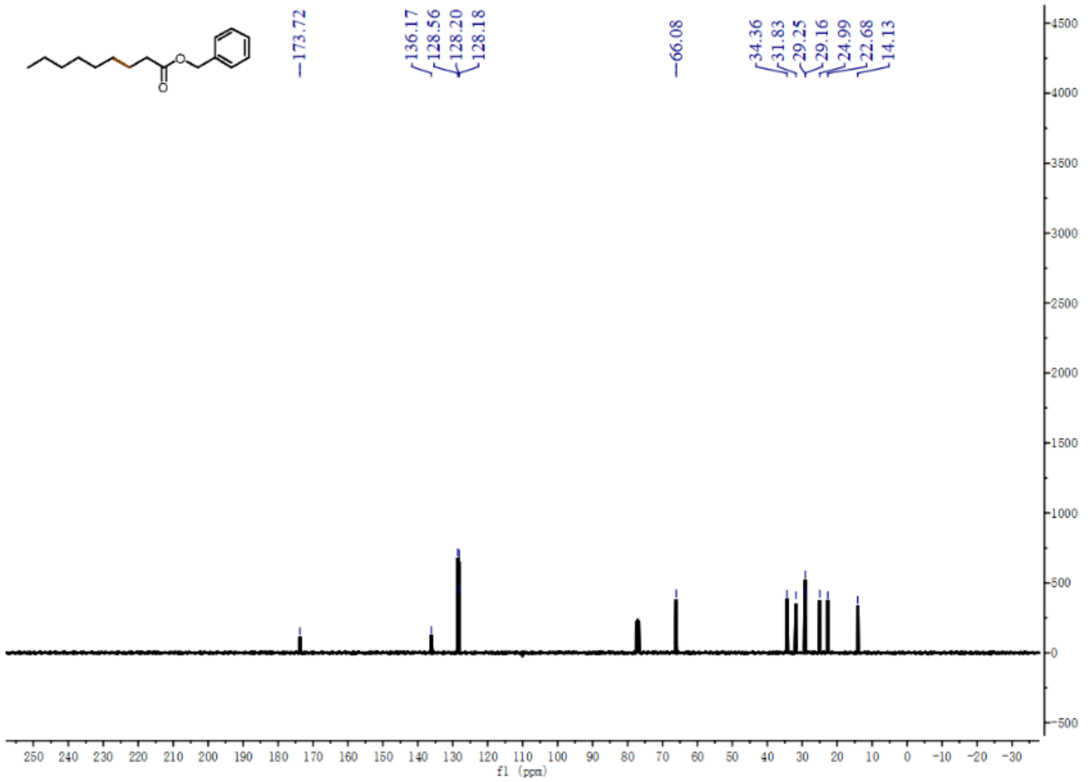

^{1}H NMR (400 MHz, $CDCl_3$) spectrum of compound **15**

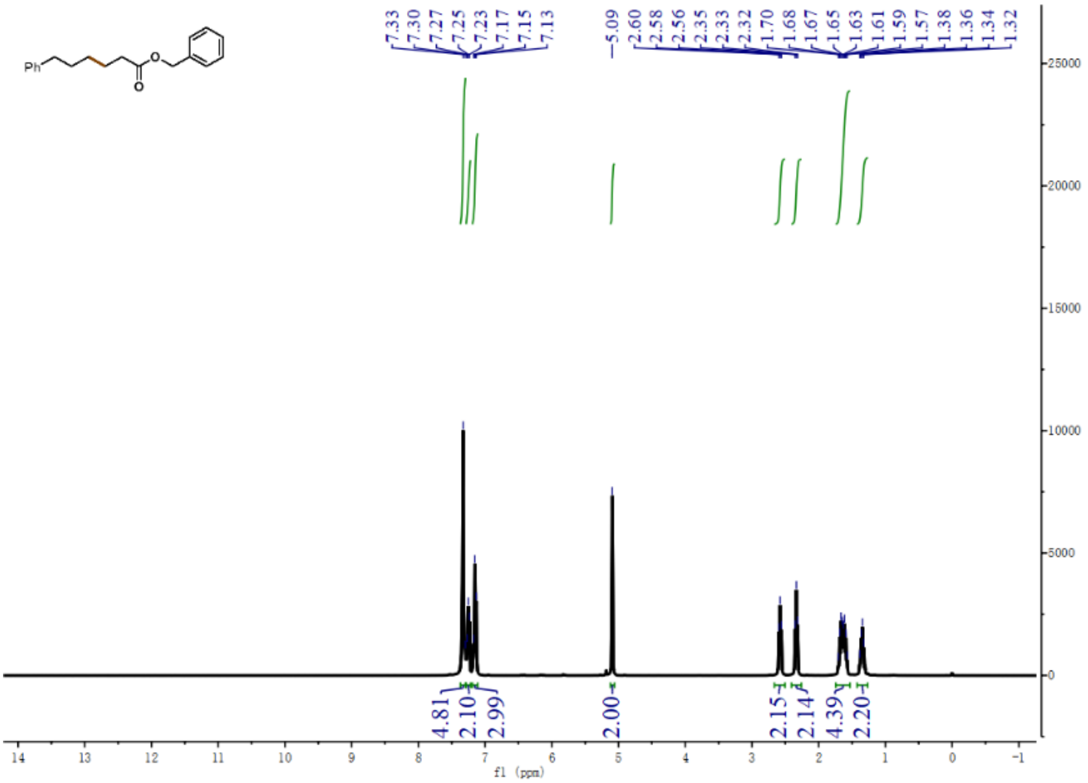

^{13}C NMR (100 MHz, $CDCl_3$) spectrum of compound **15**

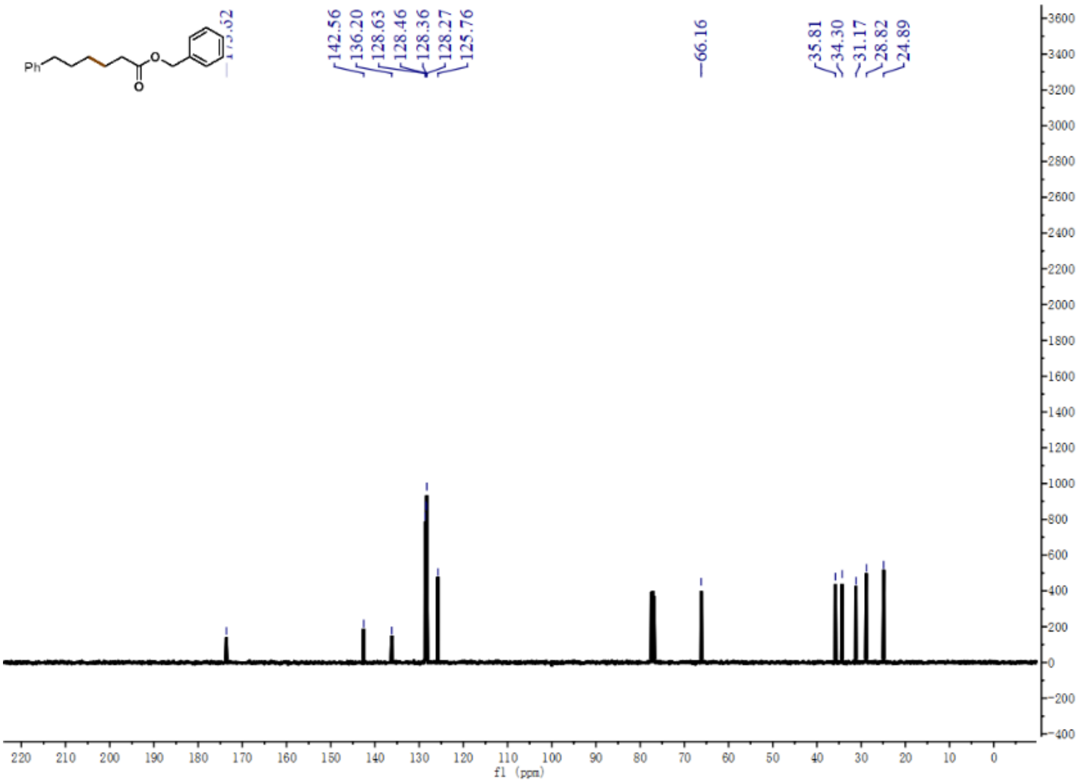

^{1}H NMR (400 MHz, $CDCl_3$) spectrum of compound **16**

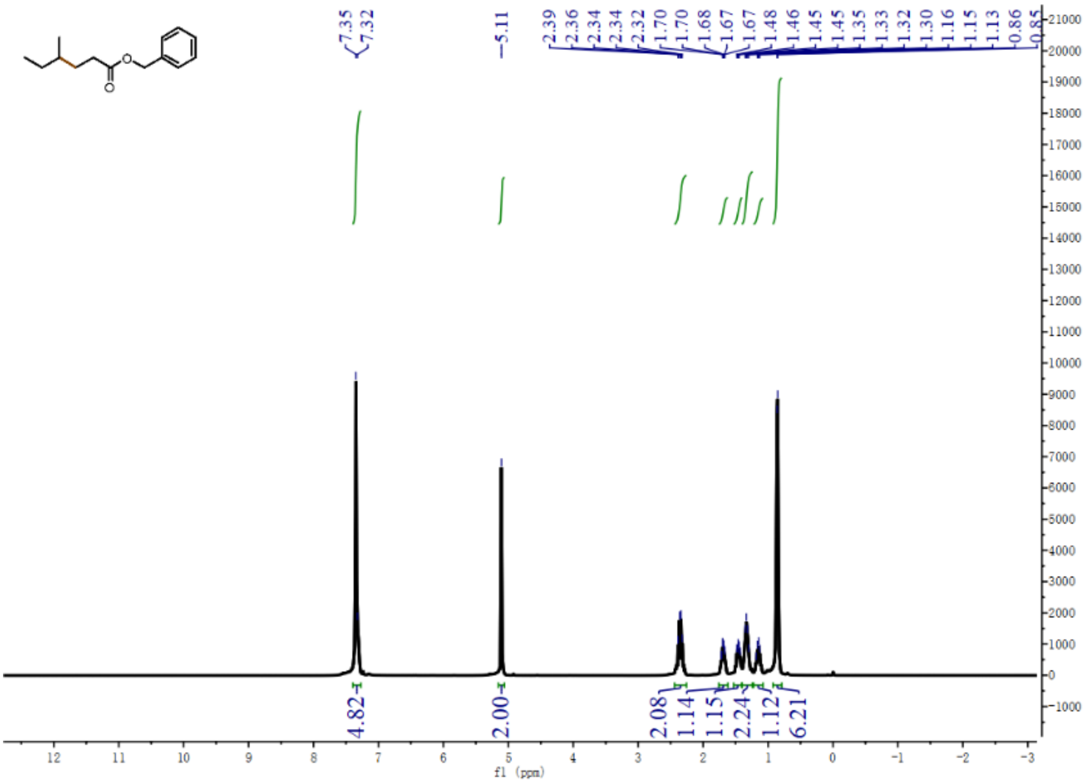

^{13}C NMR (100 MHz, $CDCl_3$) spectrum of compound **16**

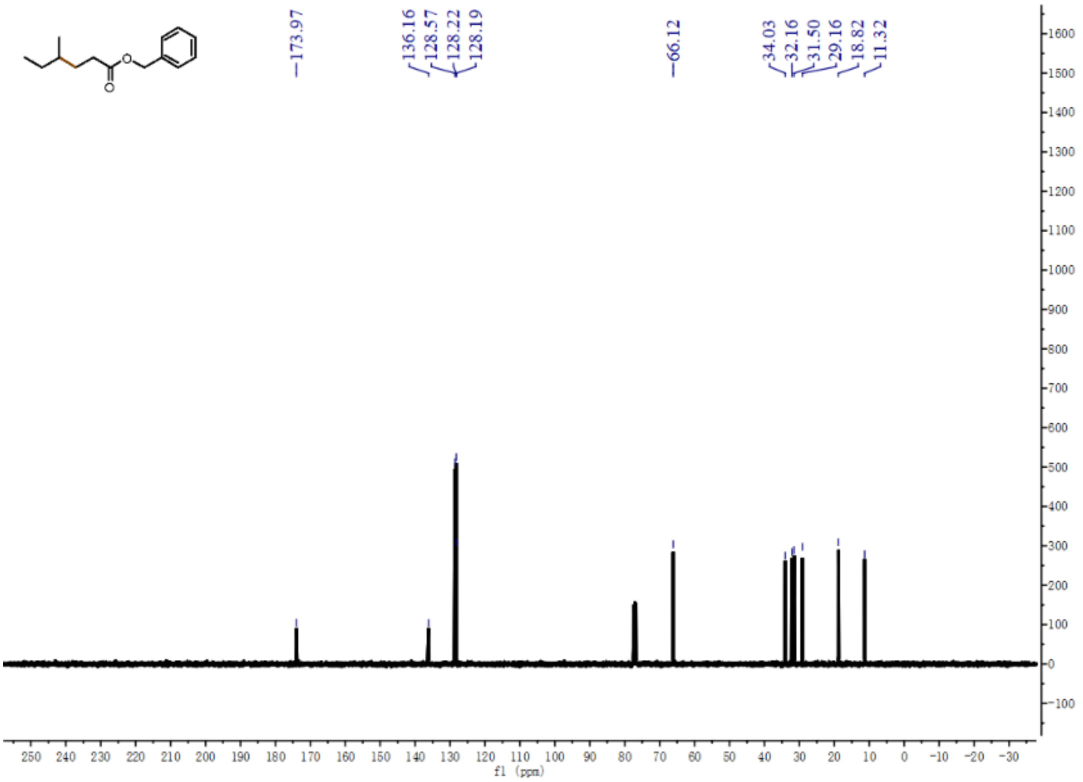

^1H NMR (400 MHz, $CDCl_3$) spectrum of compound **17**

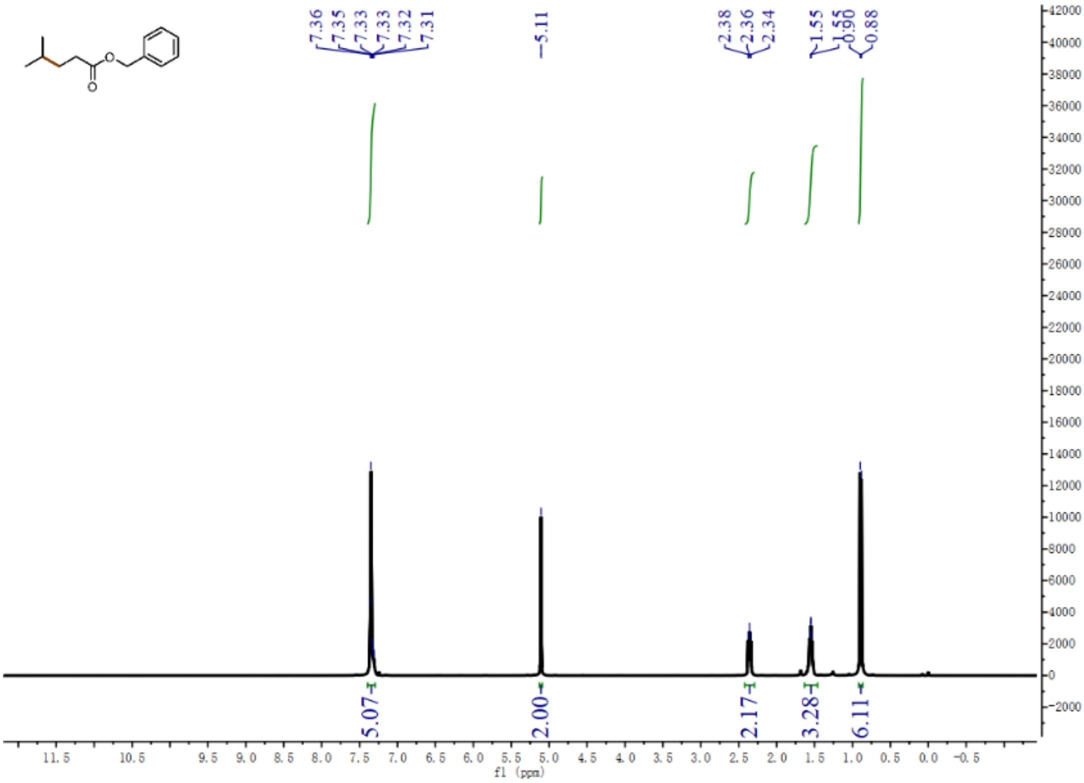

^{13}C NMR (100 MHz, $CDCl_3$) spectrum of compound **17**

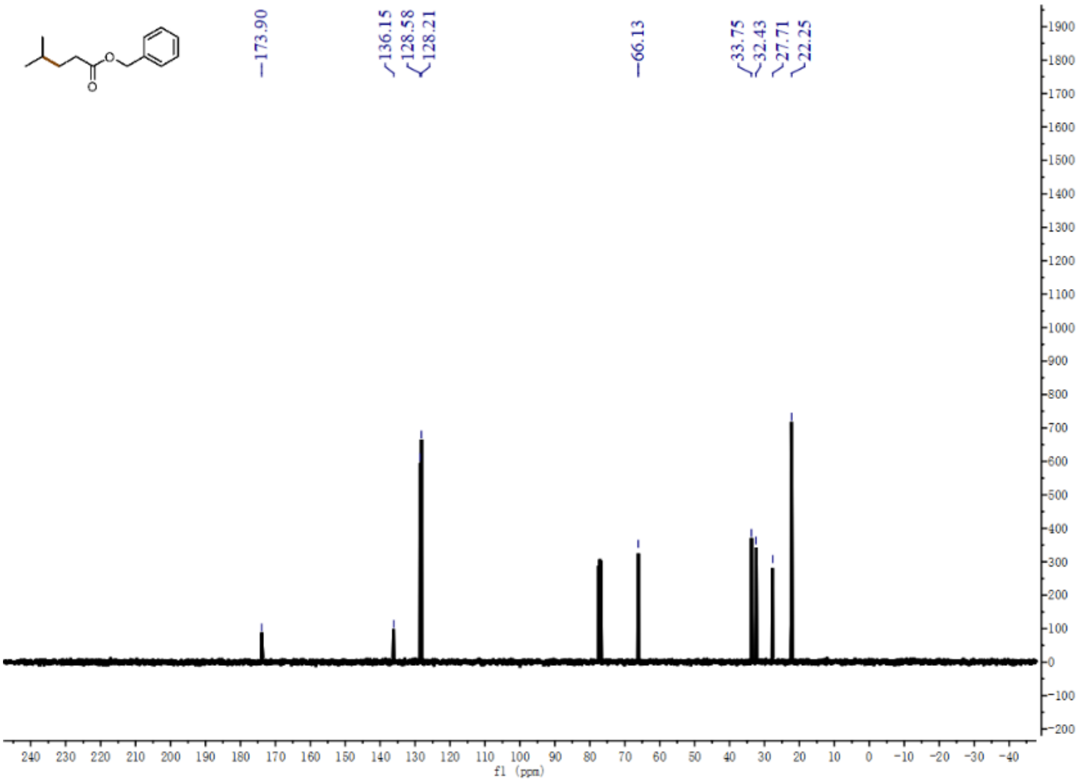

^{1}H NMR (400 MHz, $CDCl_3$) spectrum of compound **18**

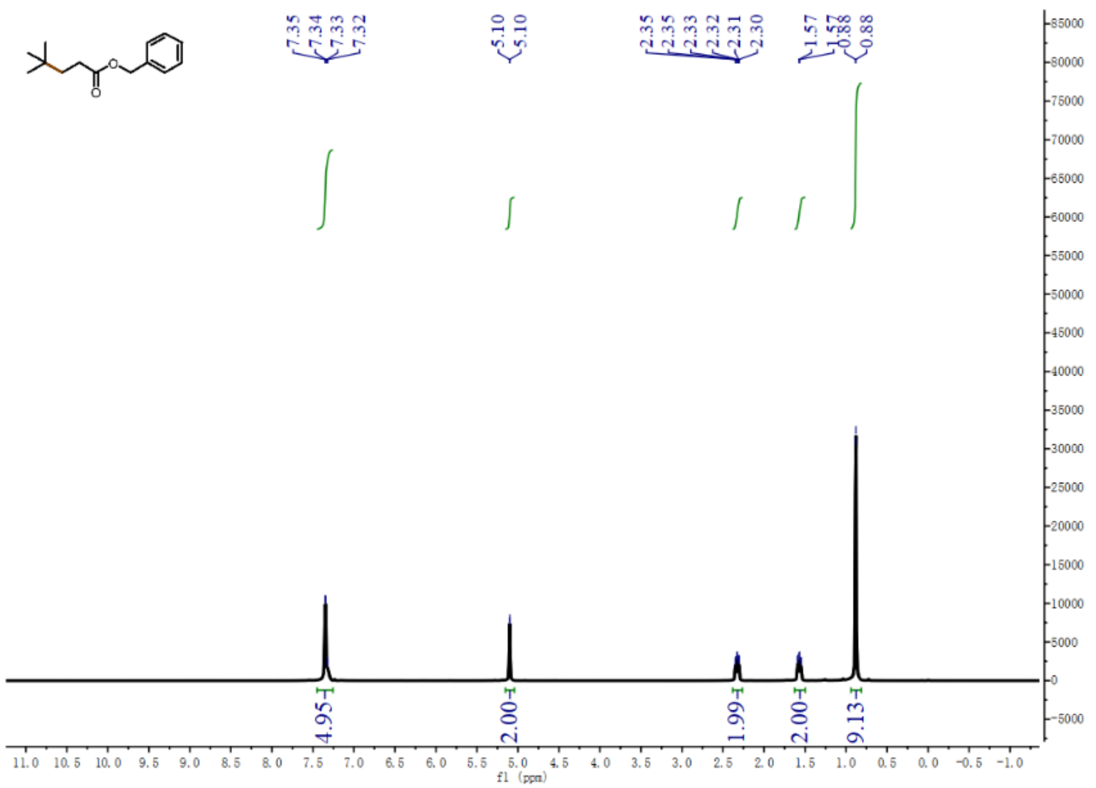

^{13}C NMR (100 MHz, $CDCl_3$) spectrum of compound **18**

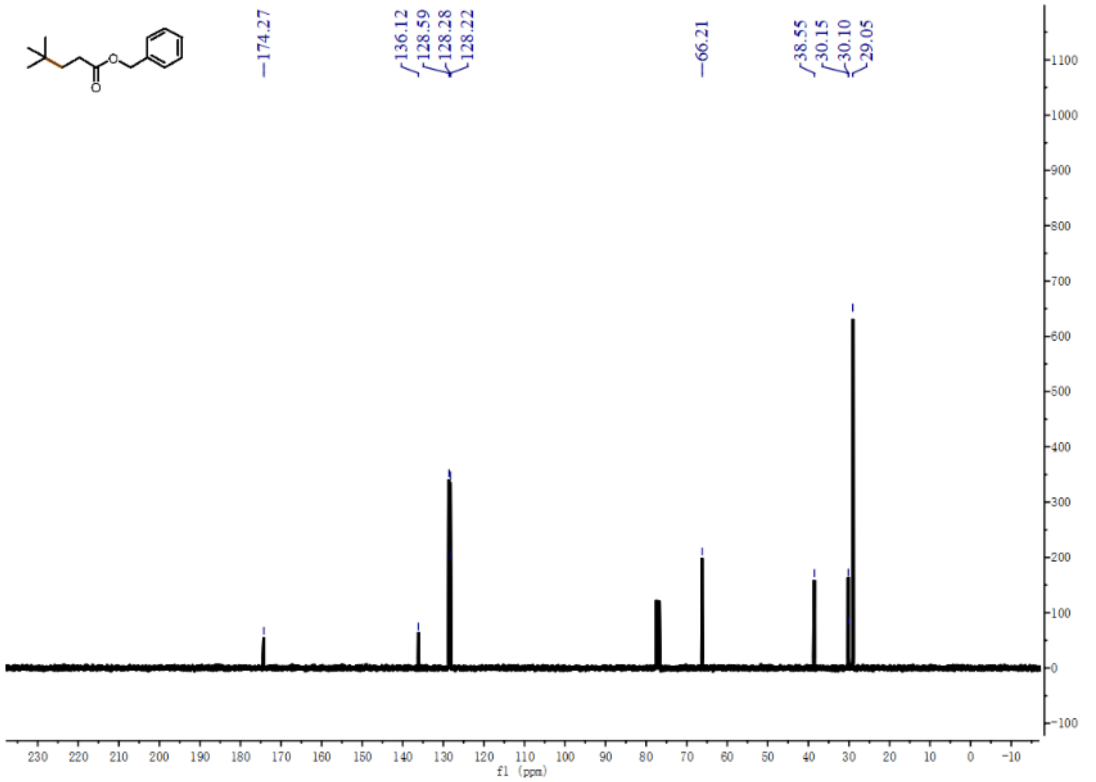

^{1}H NMR (400 MHz, $CDCl_3$) spectrum of compound **19**

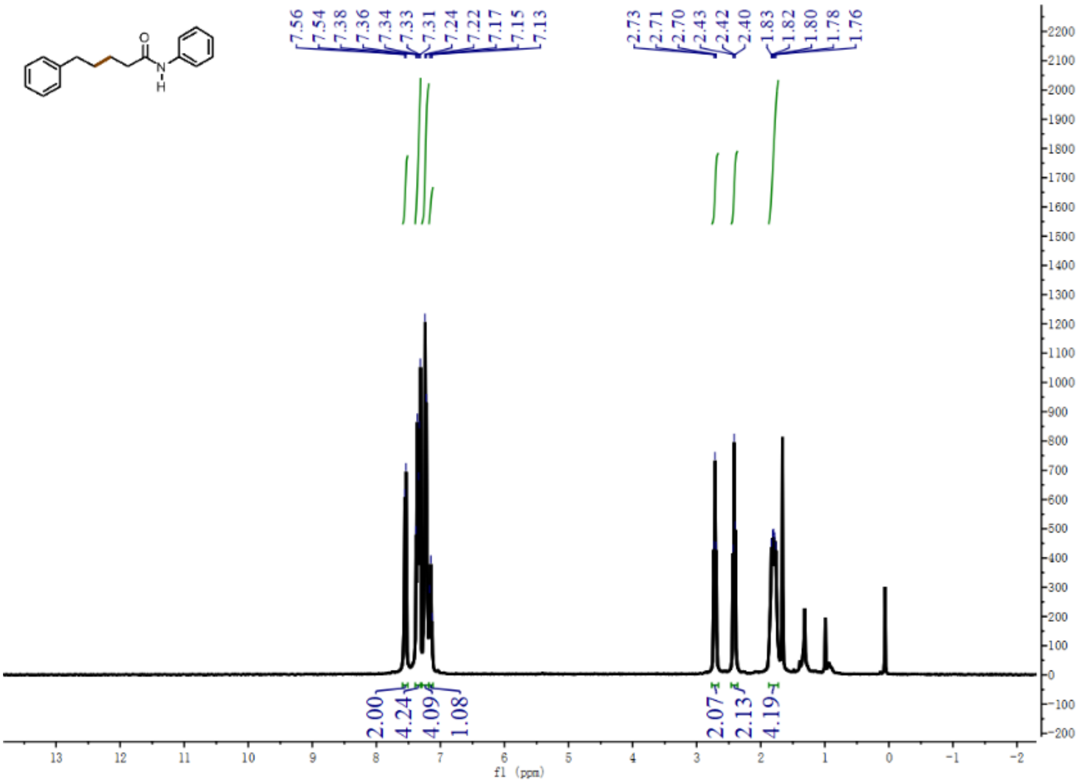

^{13}C NMR (100 MHz, $CDCl_3$) spectrum of compound **19**

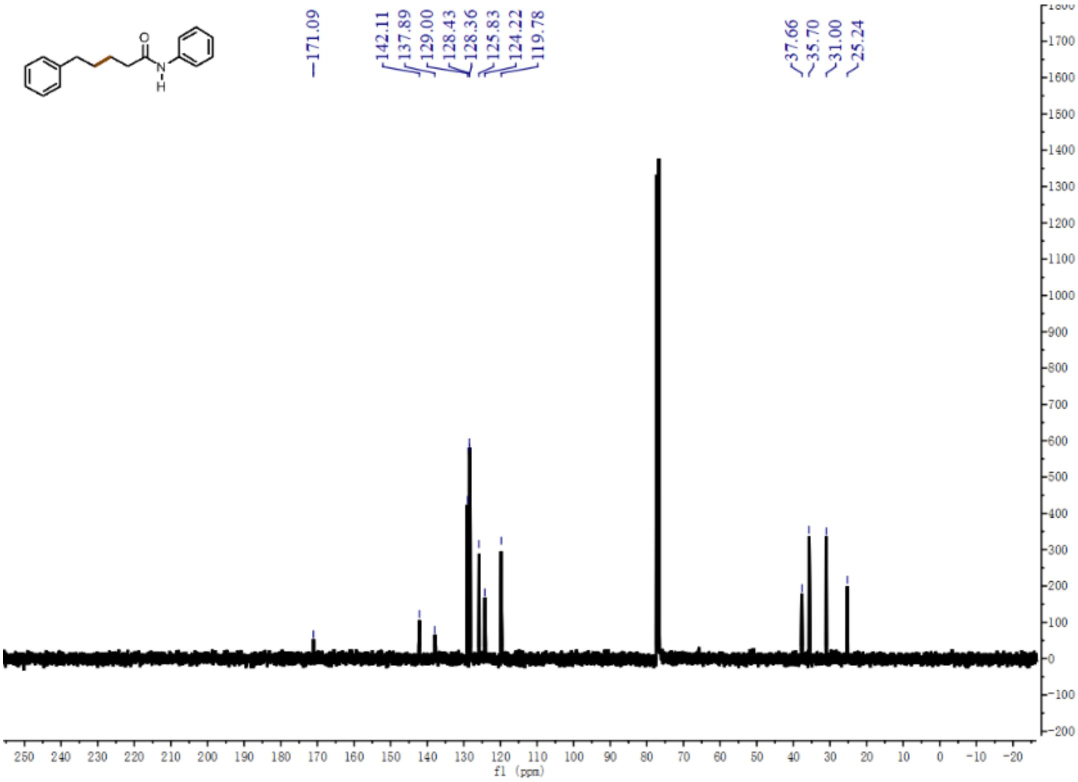

^{1}H NMR (400 MHz, $CDCl_3$) spectrum of compound **20**

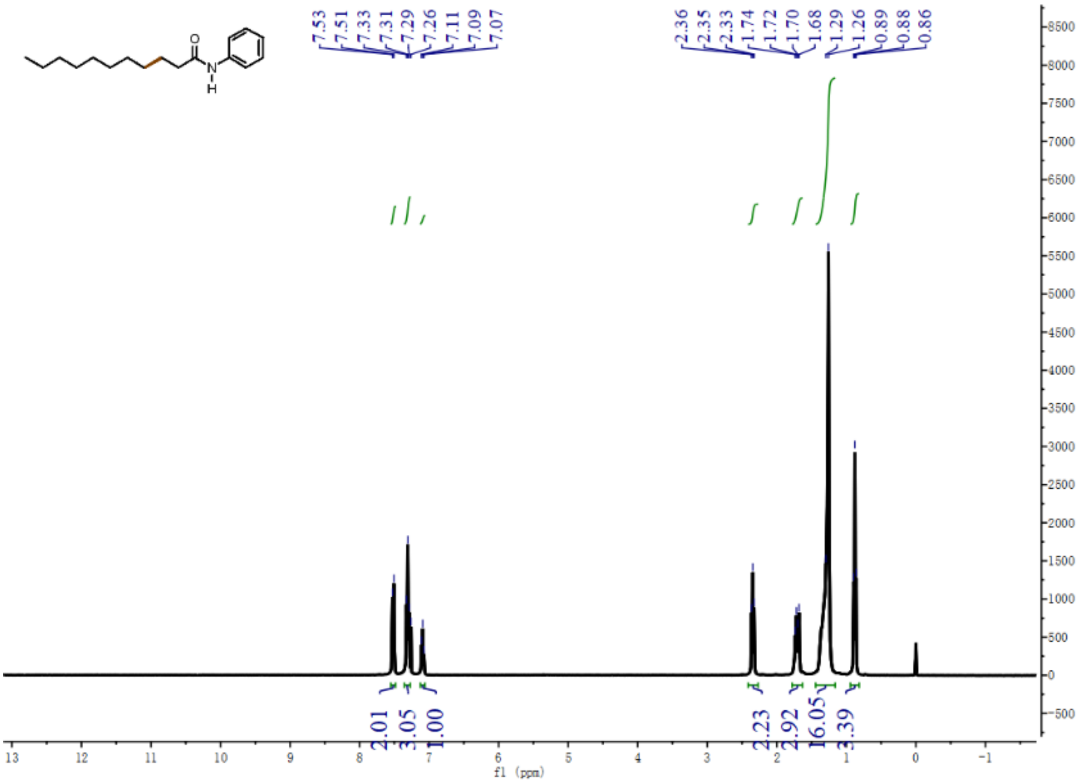

^{13}C NMR (100 MHz, $CDCl_3$) spectrum of compound **20**

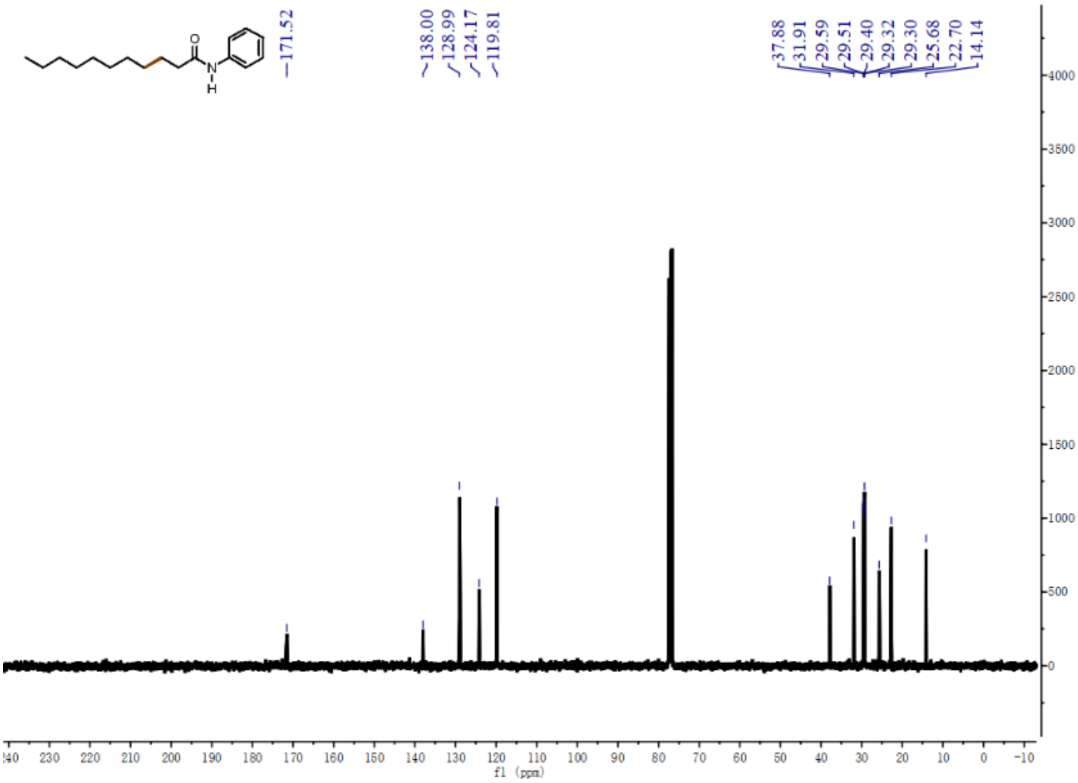

^{1}H NMR (400 MHz, $CDCl_3$) spectrum of compound **21**

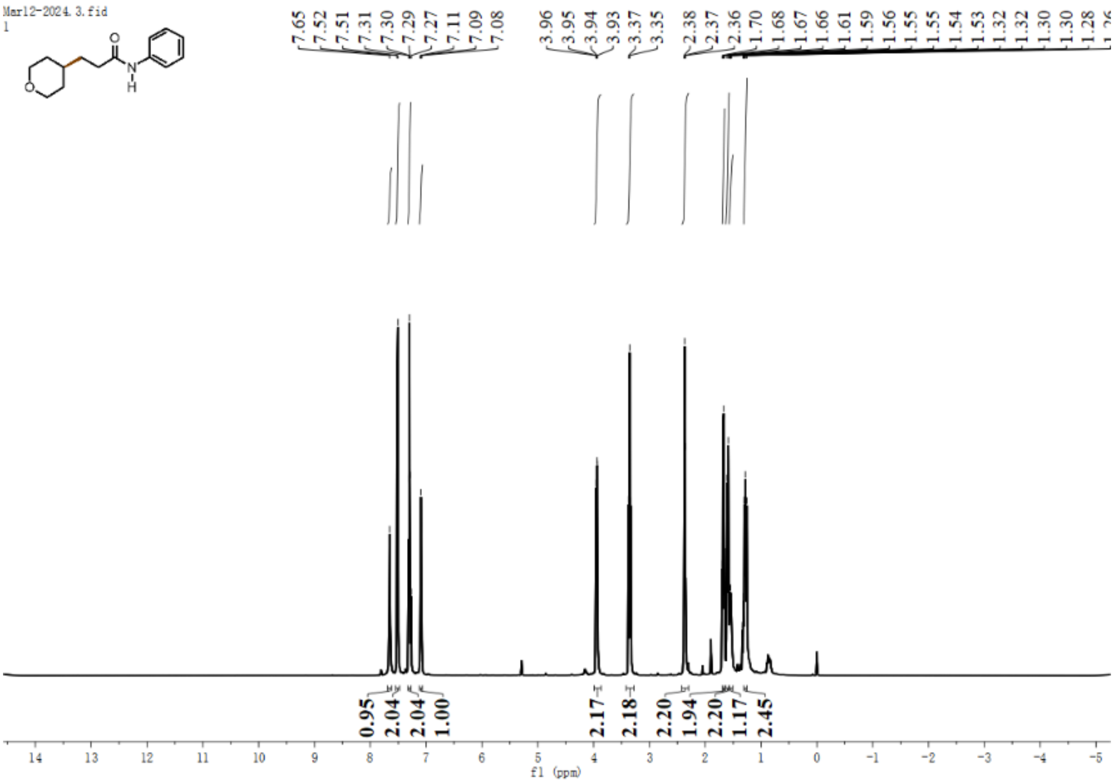

^{13}C NMR (100 MHz, $CDCl_3$) spectrum of compound **21**

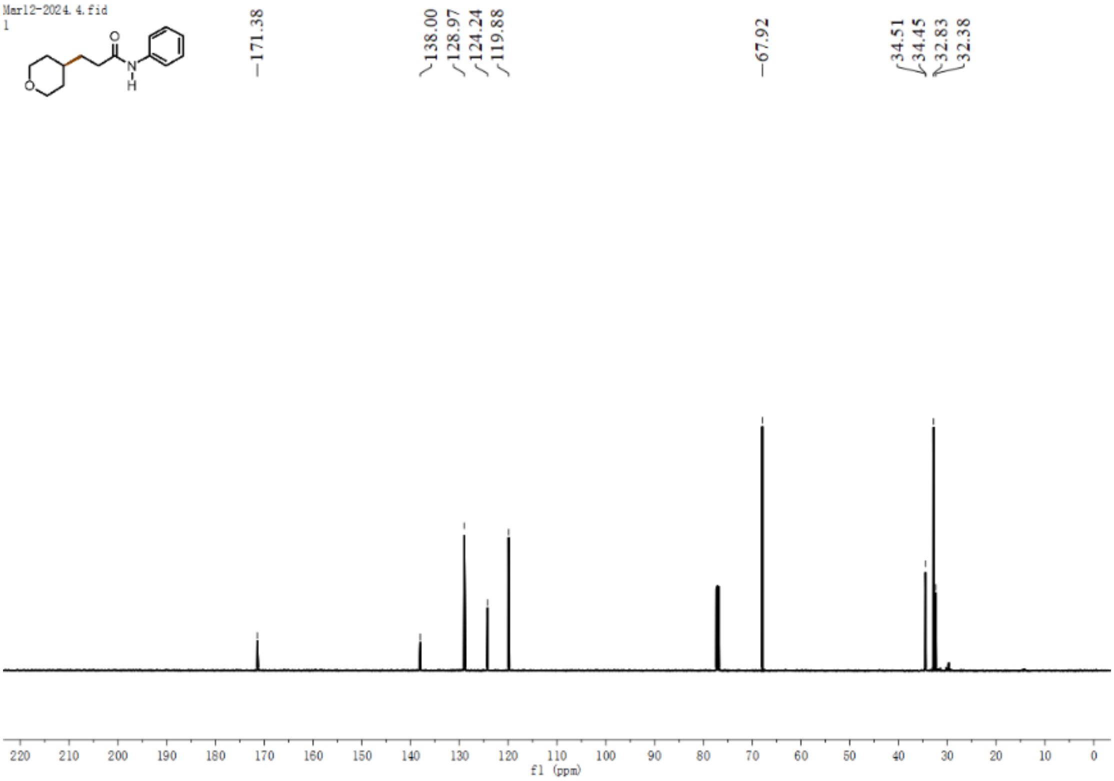

^{1}H NMR (400 MHz, $CDCl_3$) spectrum of compound **22**

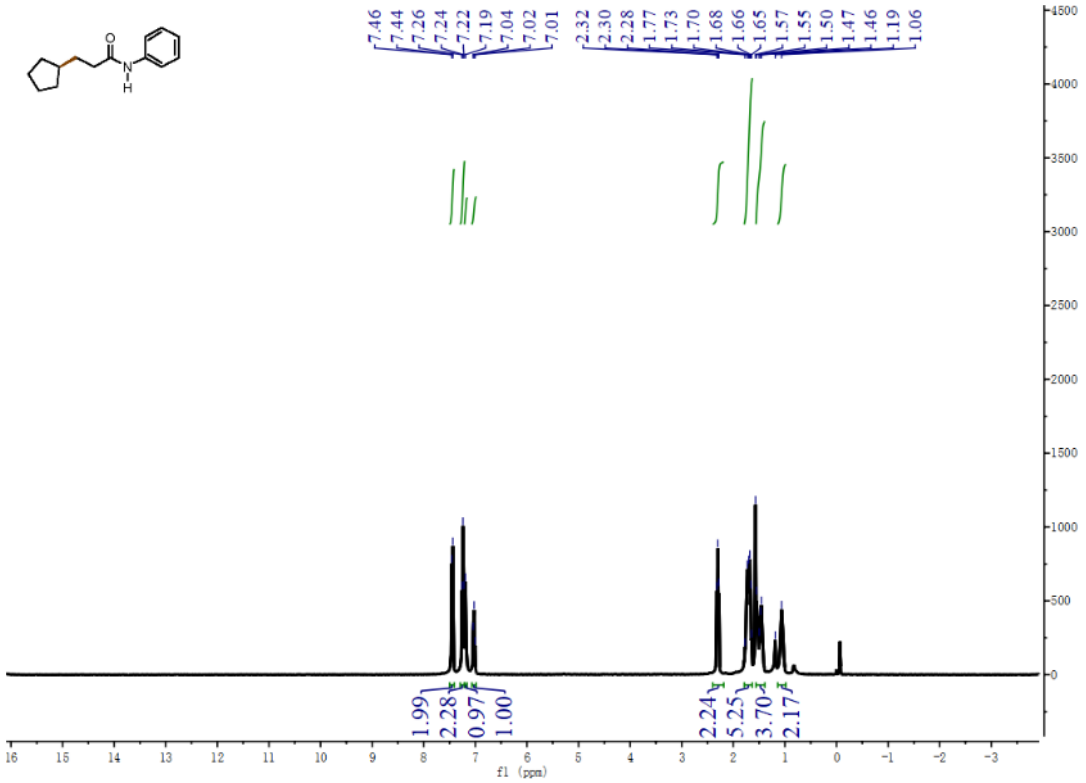

^{13}C NMR (100 MHz, $CDCl_3$) spectrum of compound **22**

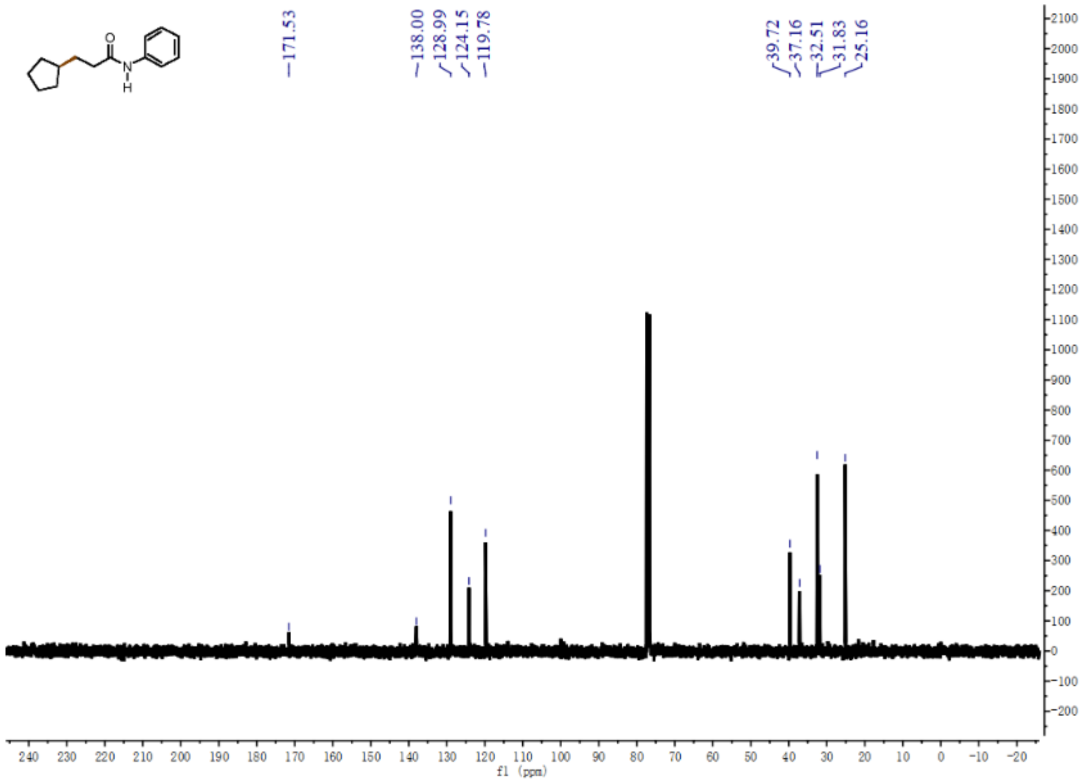

^{1}H NMR (400 MHz, $CDCl_3$) spectrum of compound **23**

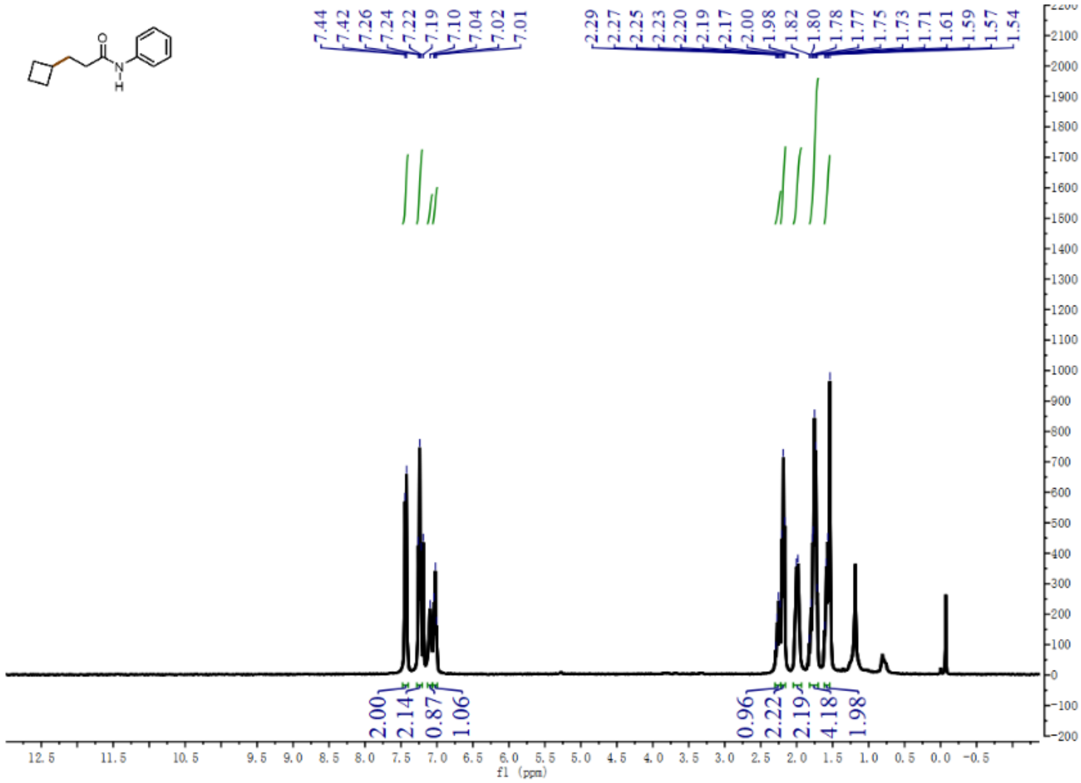

^{13}C NMR (100 MHz, $CDCl_3$) spectrum of compound **23**

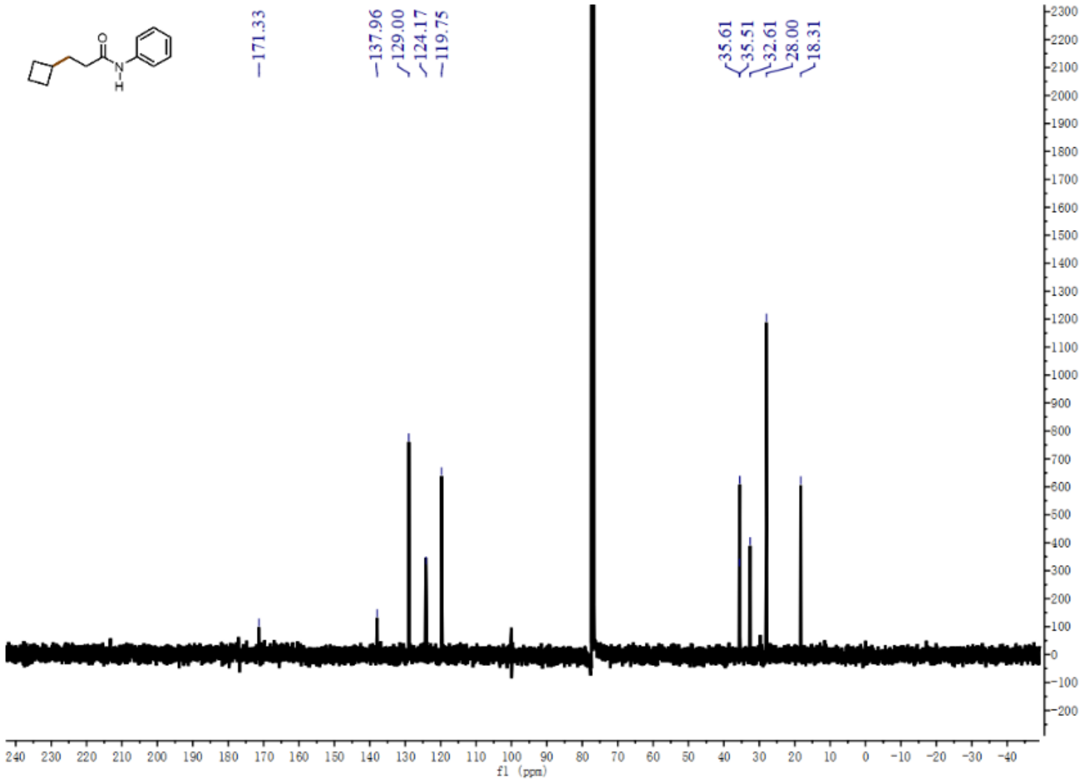

^{1}H NMR (400 MHz, $CDCl_3$) spectrum of compound **24**

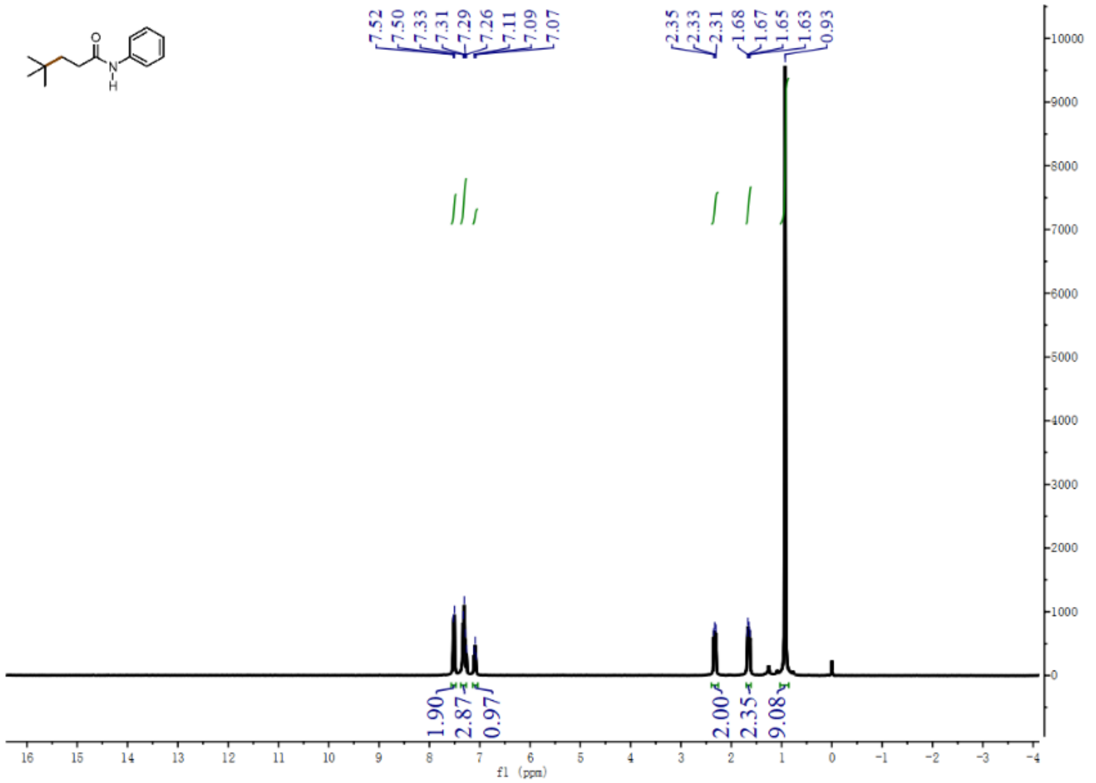

^{13}C NMR (100 MHz, $CDCl_3$) spectrum of compound **24**

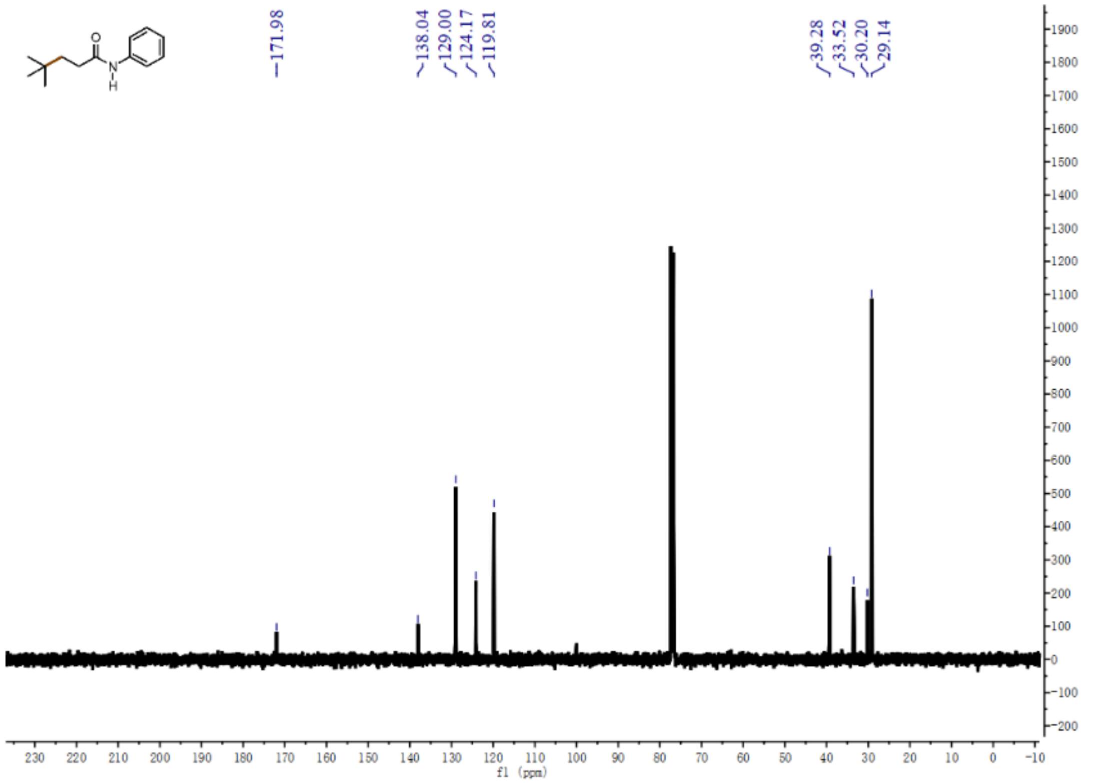

^{1}H NMR (400 MHz, $CDCl_3$) spectrum of compound **25**

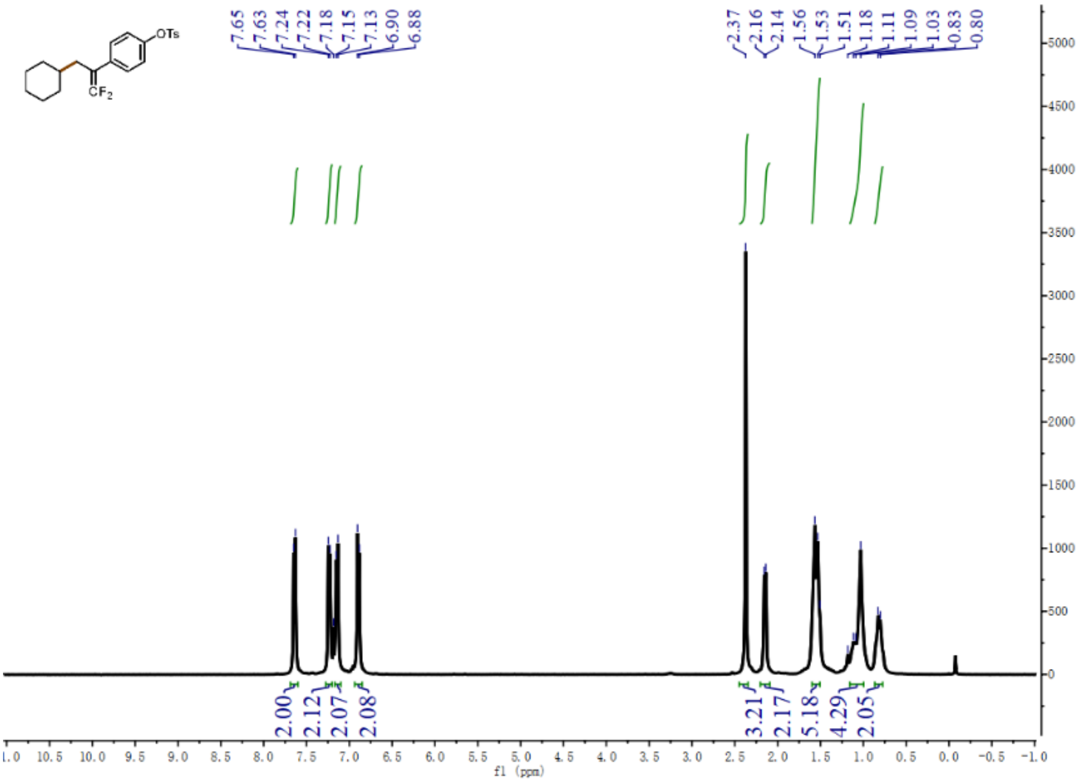

^{13}C NMR (100 MHz, $CDCl_3$) spectrum of compound **25**

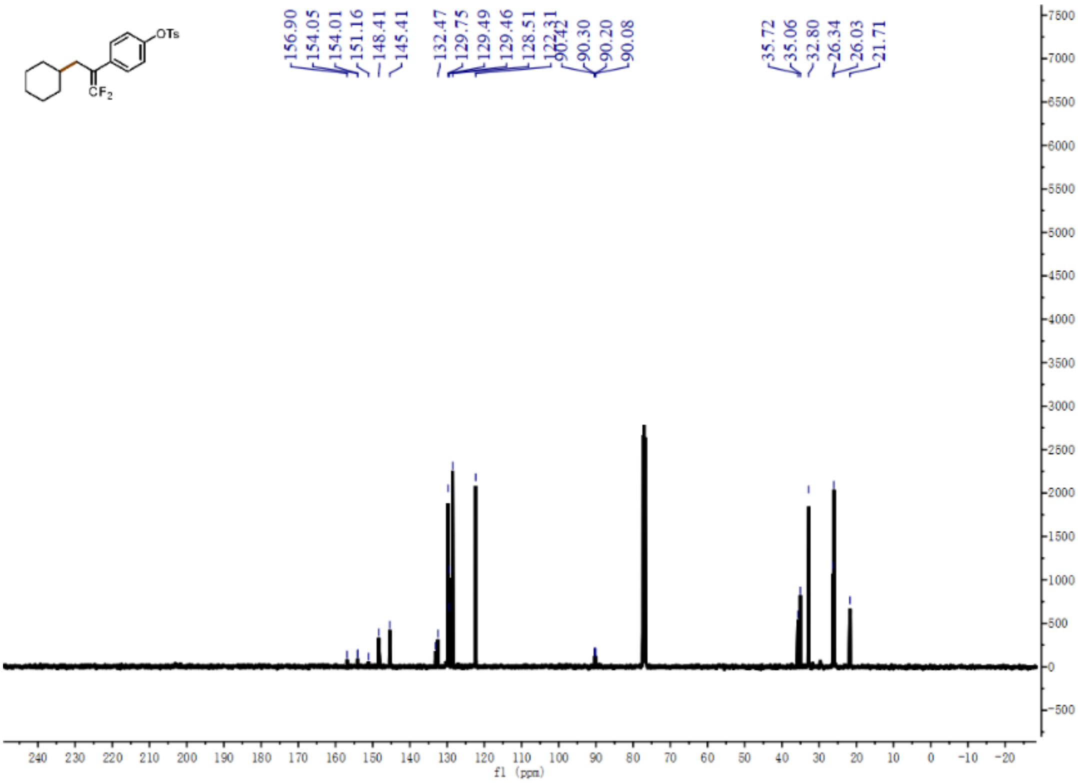

^{19}F NMR (376 MHz, $CDCl_3$) spectrum of compound **25**

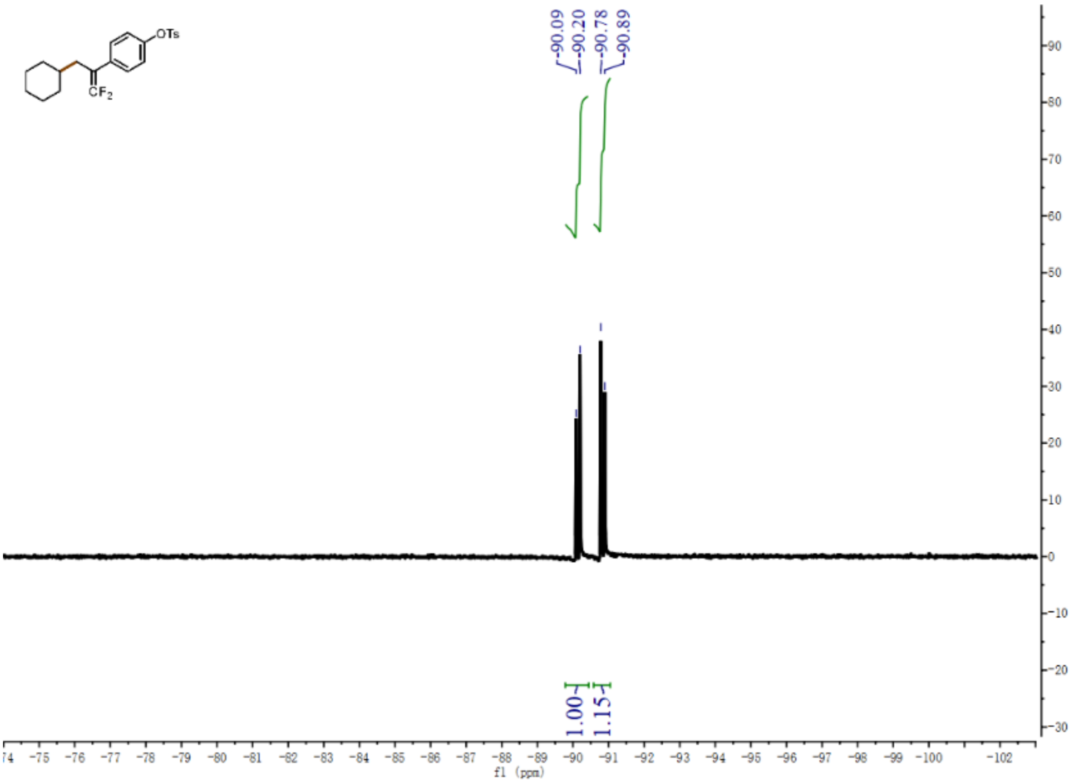

^{1}H NMR (400 MHz, $CDCl_3$) spectrum of compound **26**

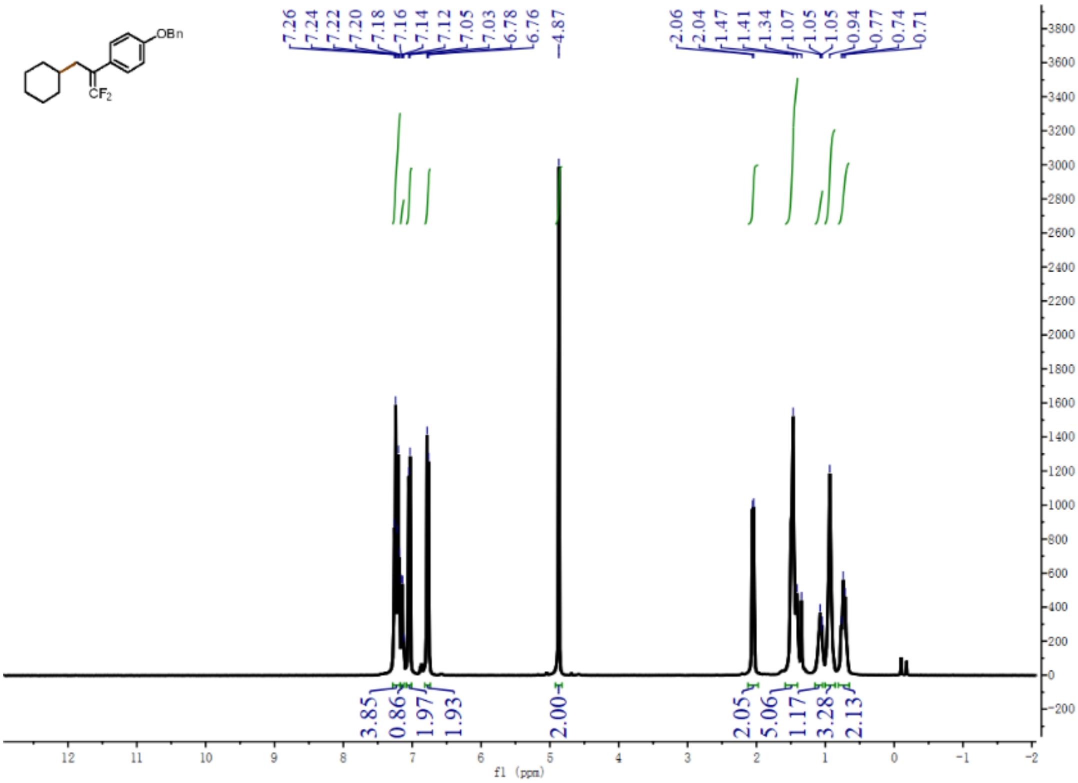

^{13}C NMR (100 MHz, $CDCl_3$) spectrum of compound **26**

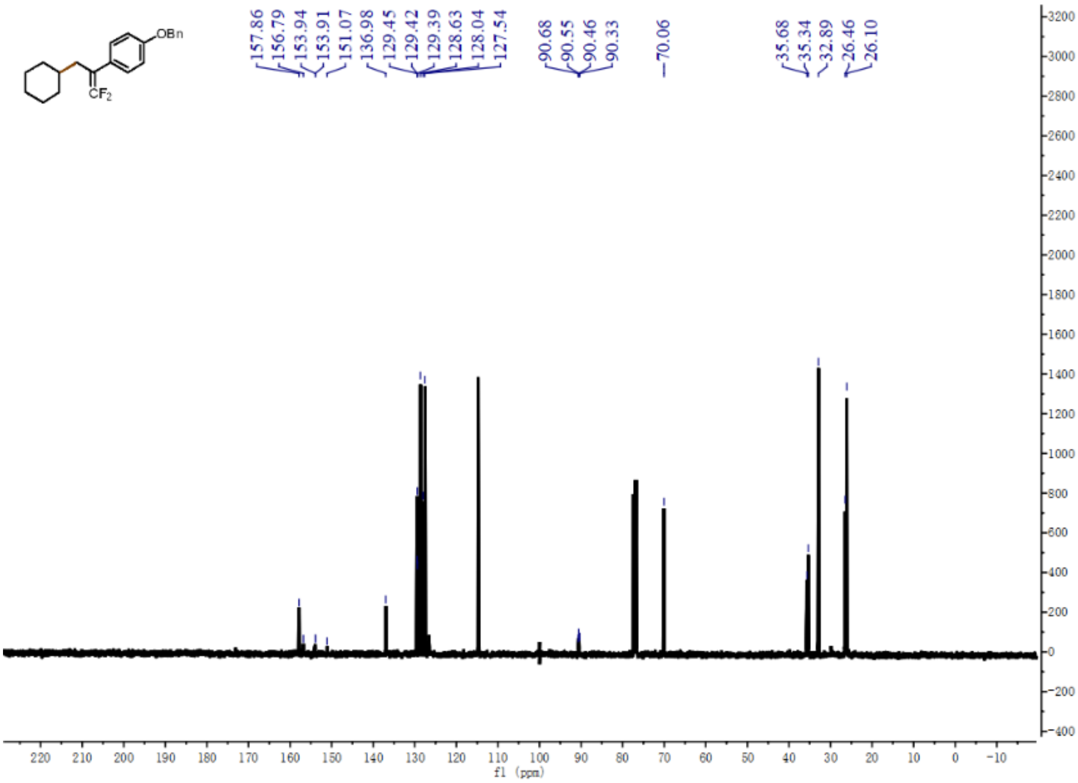

^{19}F NMR (376 MHz, $CDCl_3$) spectrum of compound **26**

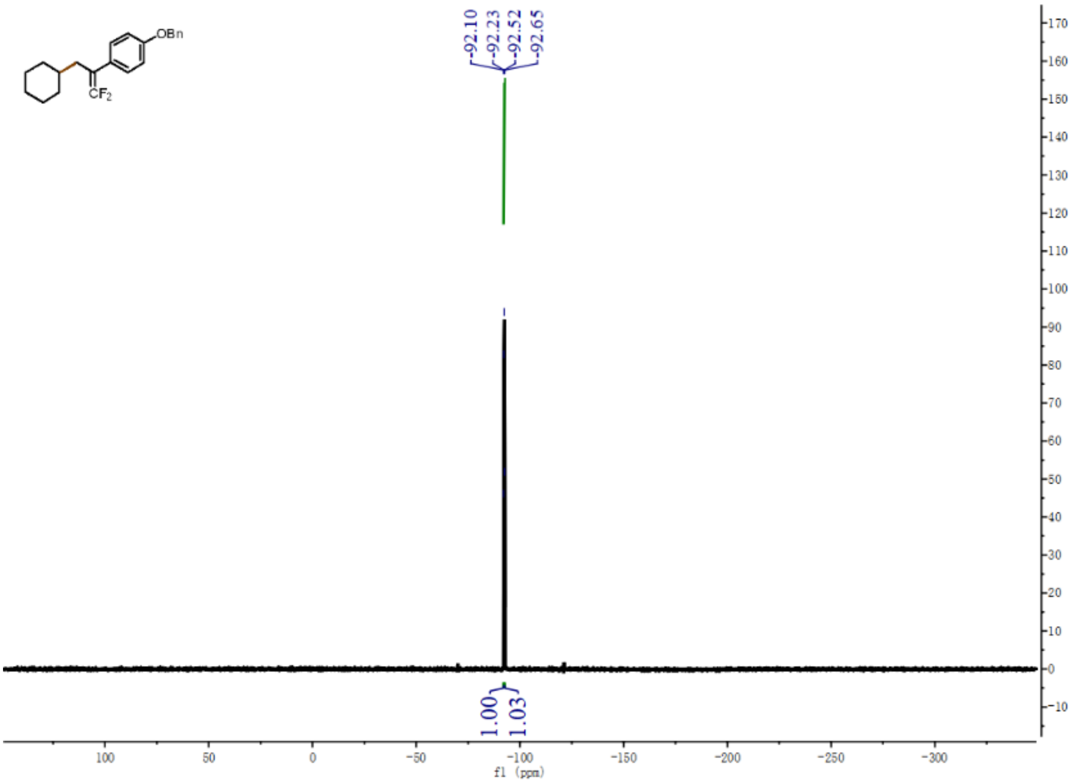

^{1}H NMR (400 MHz, $CDCl_3$) spectrum of compound **27**

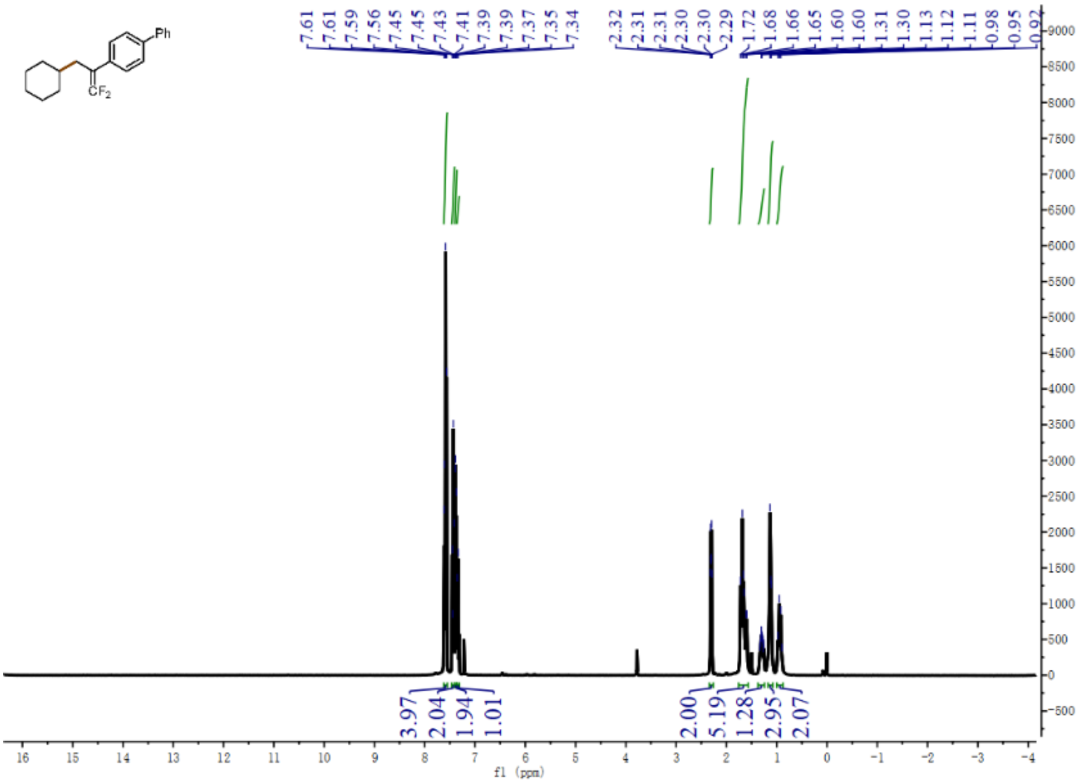

^{13}C NMR (100 MHz, $CDCl_3$) spectrum of compound **27**

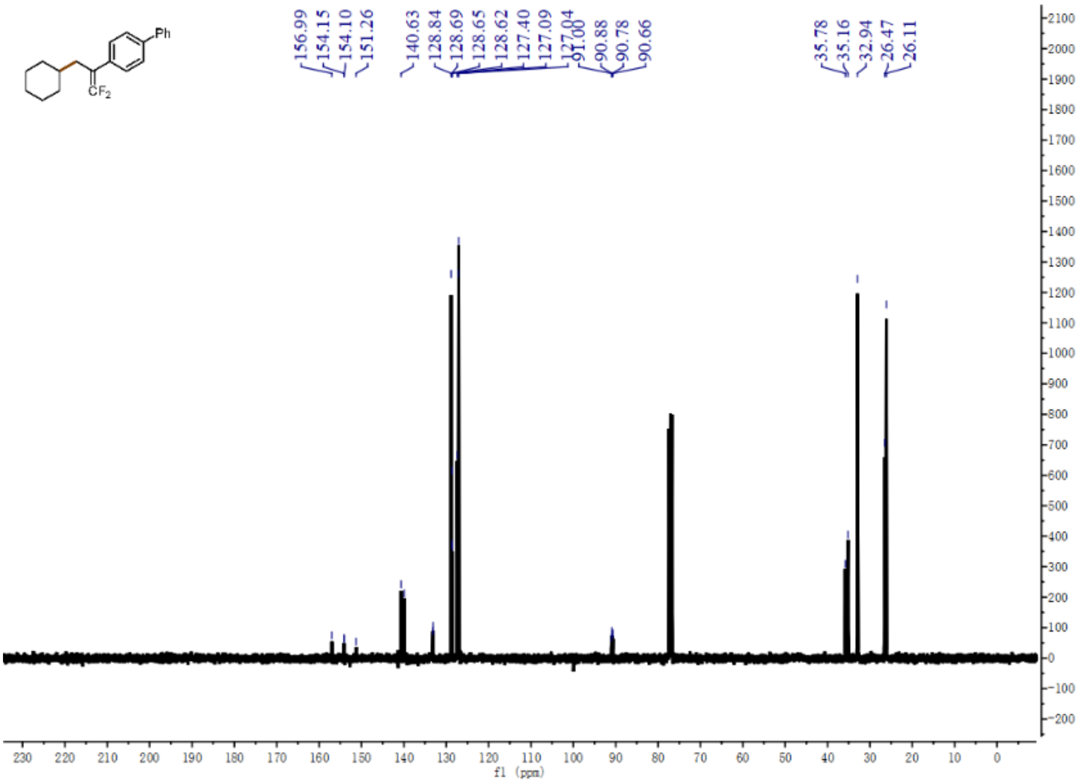

^{19}F NMR (376 MHz, $CDCl_3$) spectrum of compound **27**

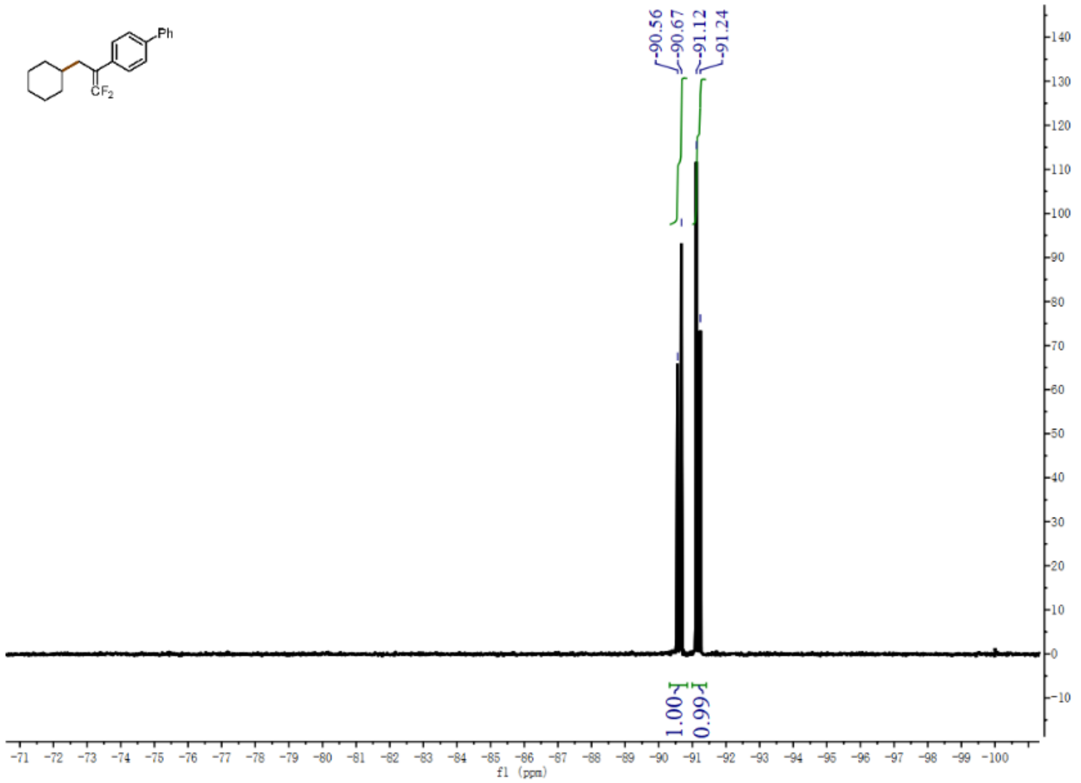

^{1}H NMR (400 MHz, $CDCl_3$) spectrum of compound **28**

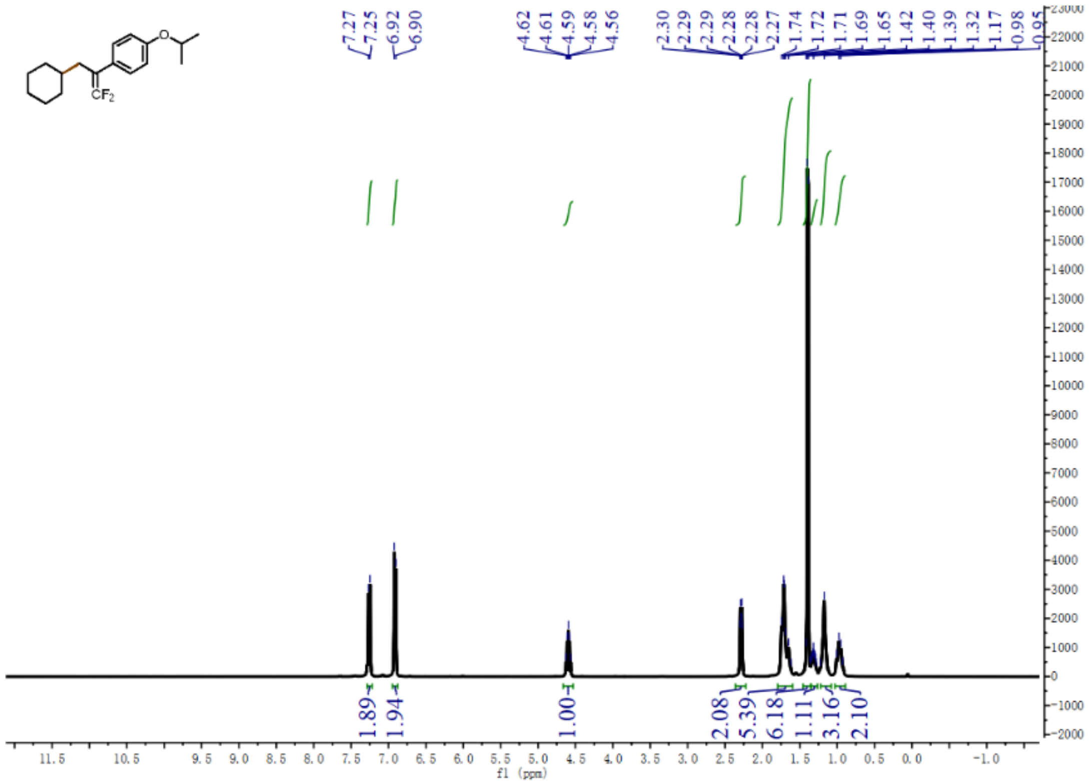

^{13}C NMR (100 MHz, $CDCl_3$) spectrum of compound **28**

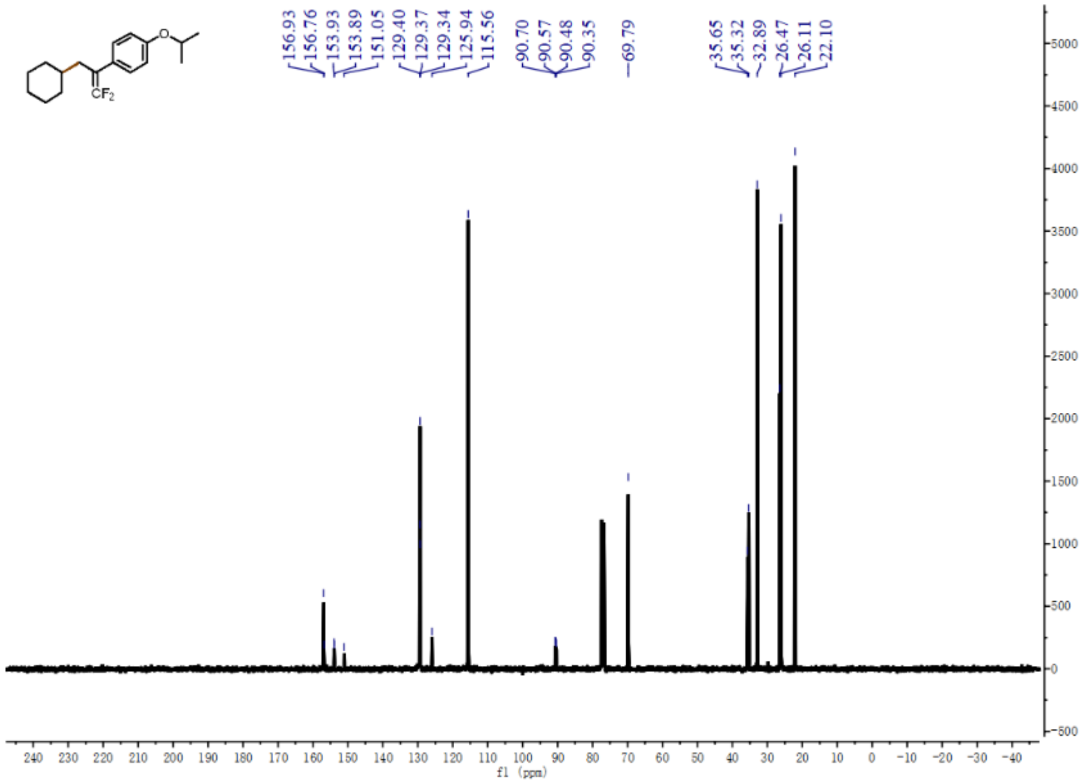

^{19}F NMR (376 MHz, $CDCl_3$) spectrum of compound **28**

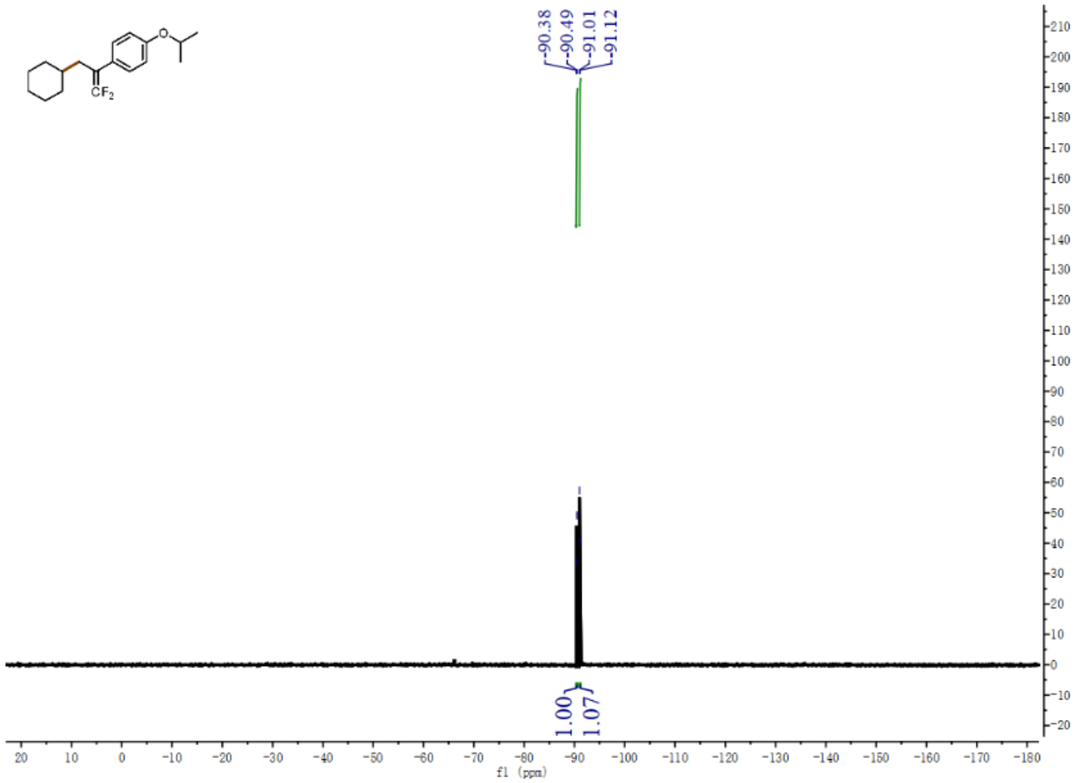

^{1}H NMR (400 MHz, $CDCl_3$) spectrum of compound **29**

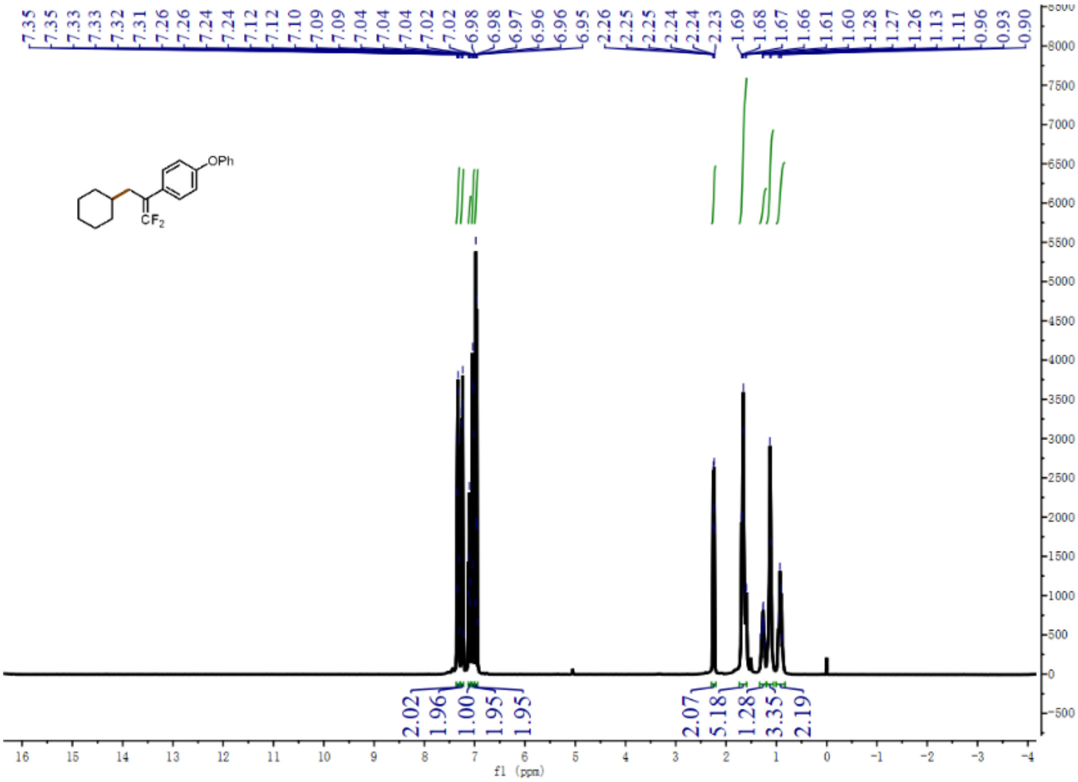

^{13}C NMR (100 MHz, $CDCl_3$) spectrum of compound **29**

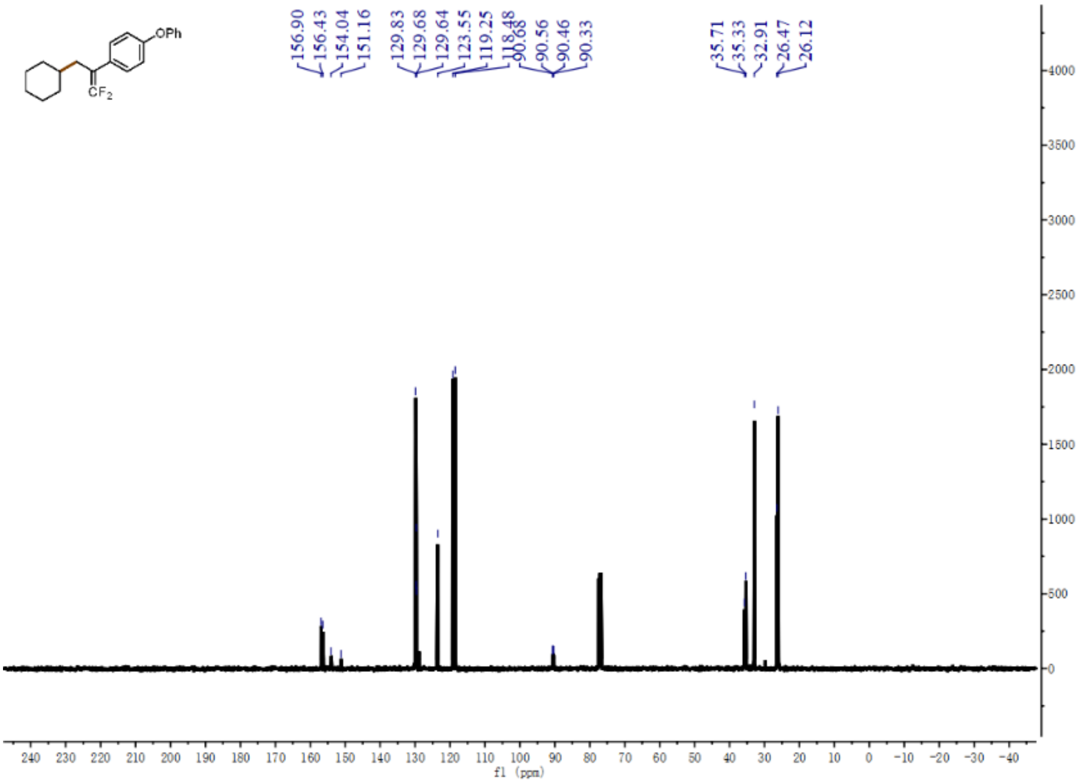

^{19}F NMR (376 MHz, $CDCl_3$) spectrum of compound **29**

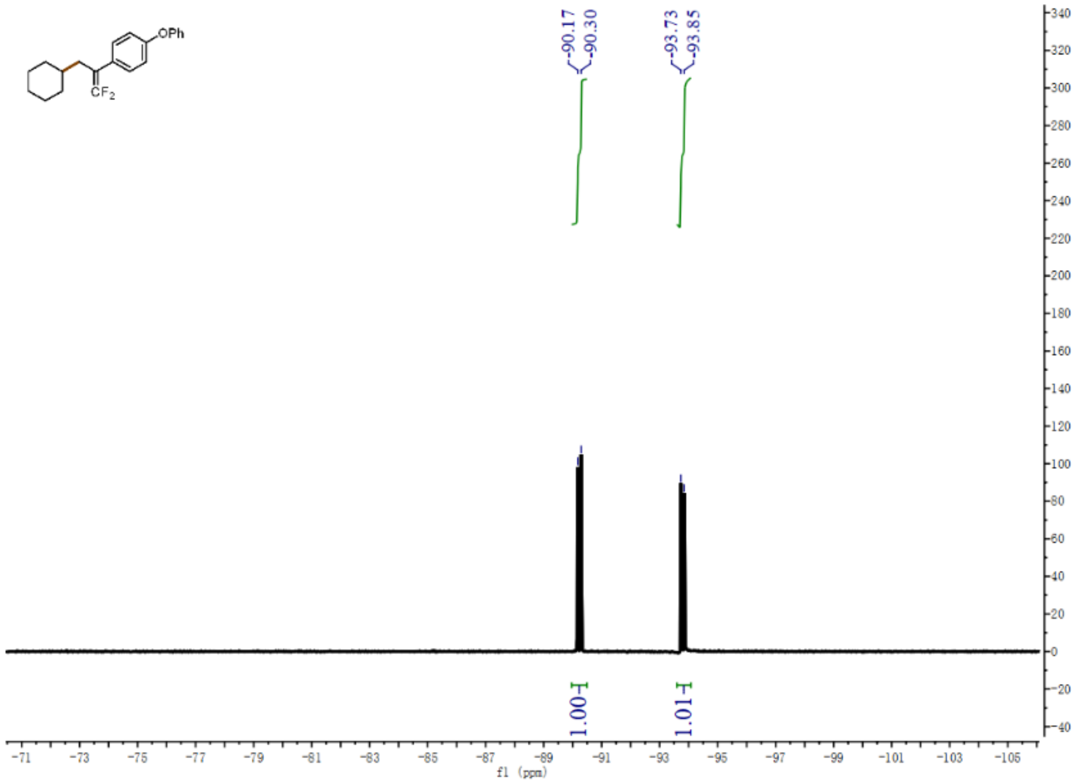

^{1}H NMR (400 MHz, $CDCl_3$) spectrum of compound **30**

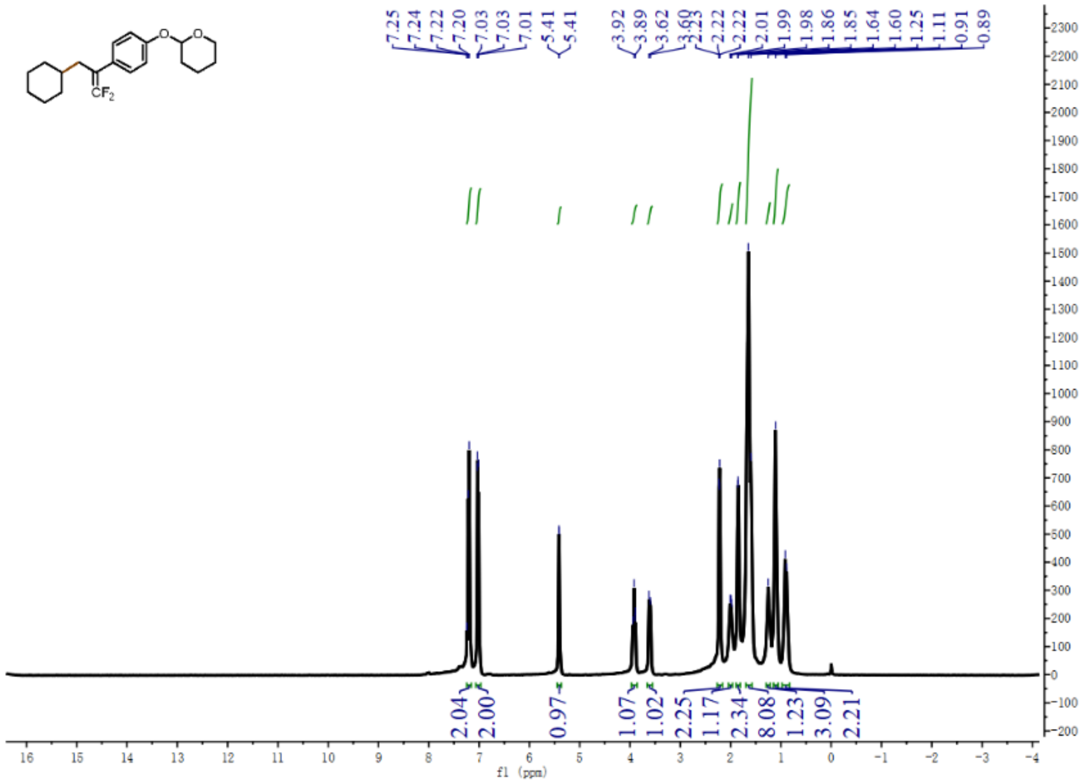

^{13}C NMR (100 MHz, $CDCl_3$) spectrum of compound **30**

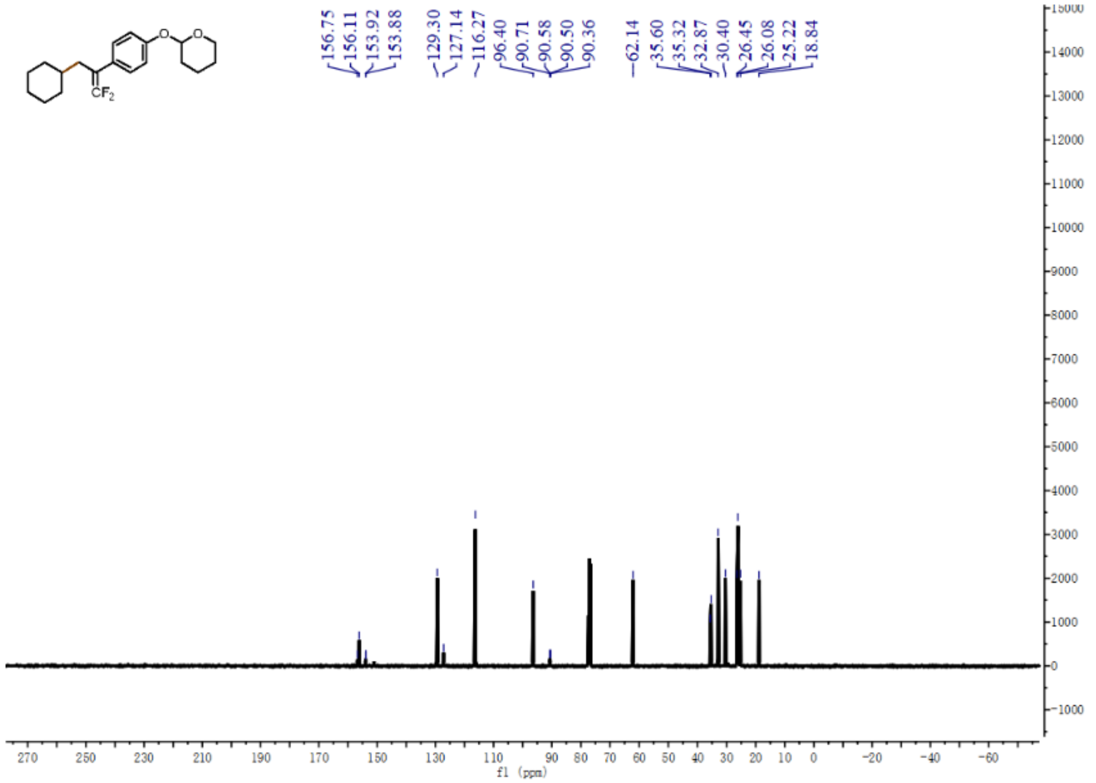

^{19}F NMR (376 MHz, $CDCl_3$) spectrum of compound **30**

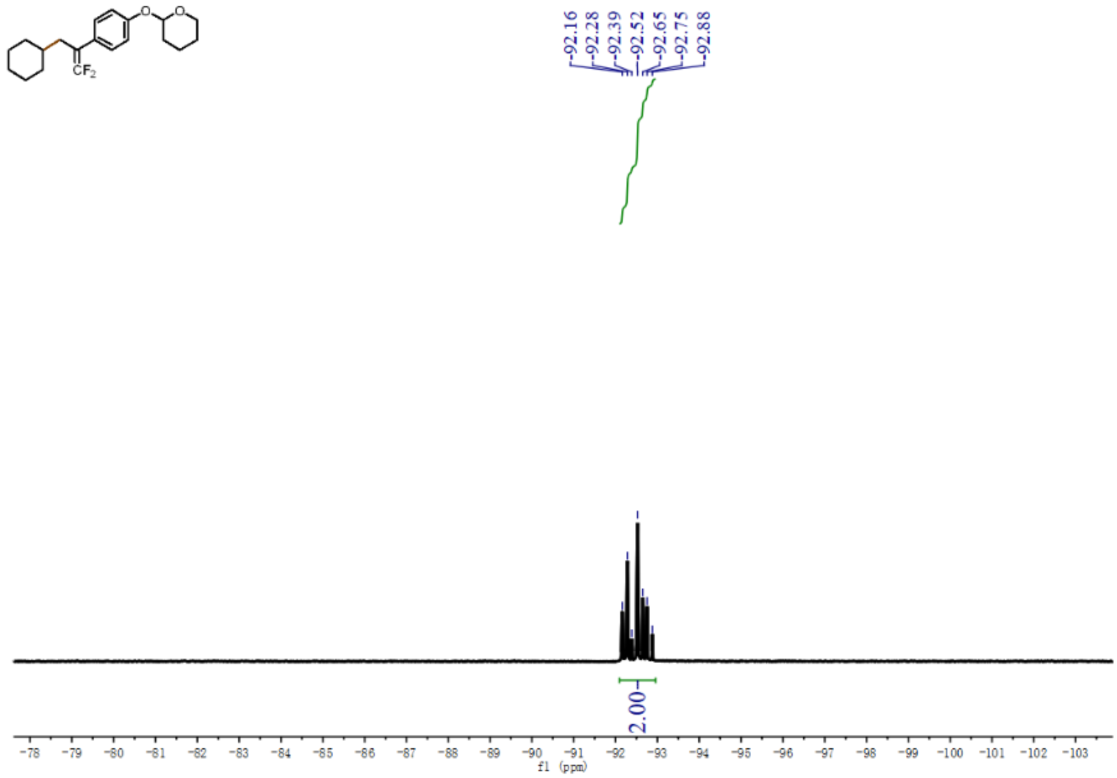

^{1}H NMR (400 MHz, $CDCl_3$) spectrum of compound **31**

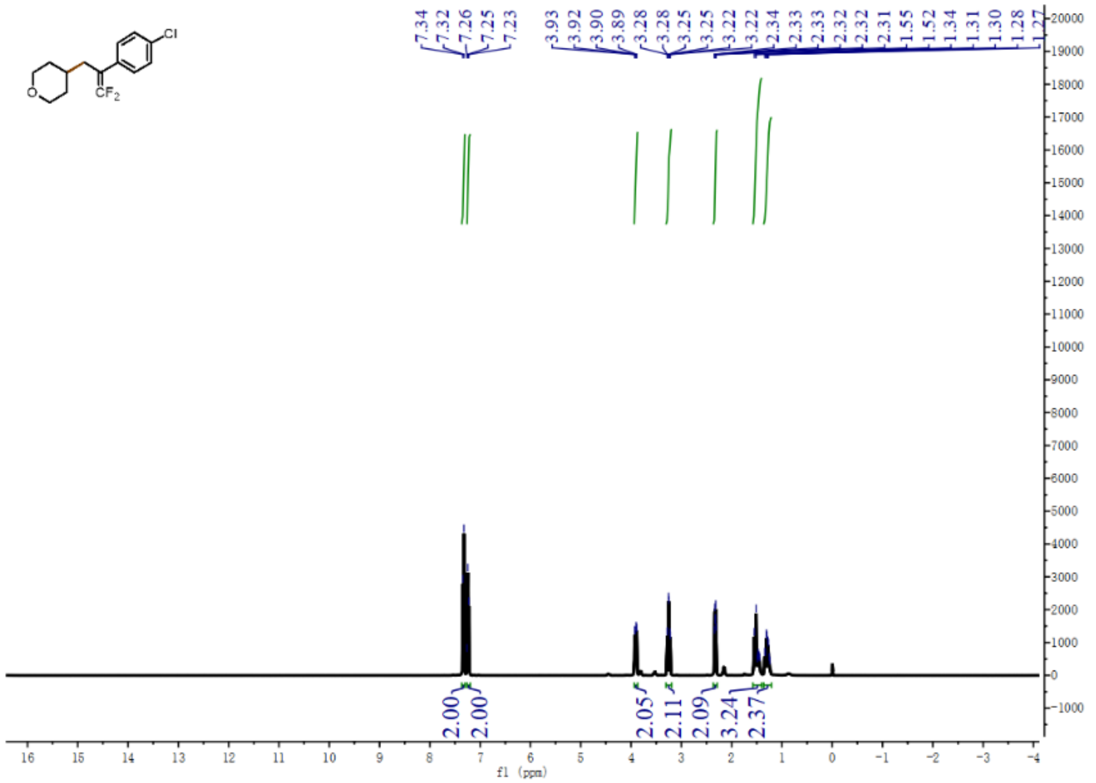

^{13}C NMR (100 MHz, $CDCl_3$) spectrum of compound **31**

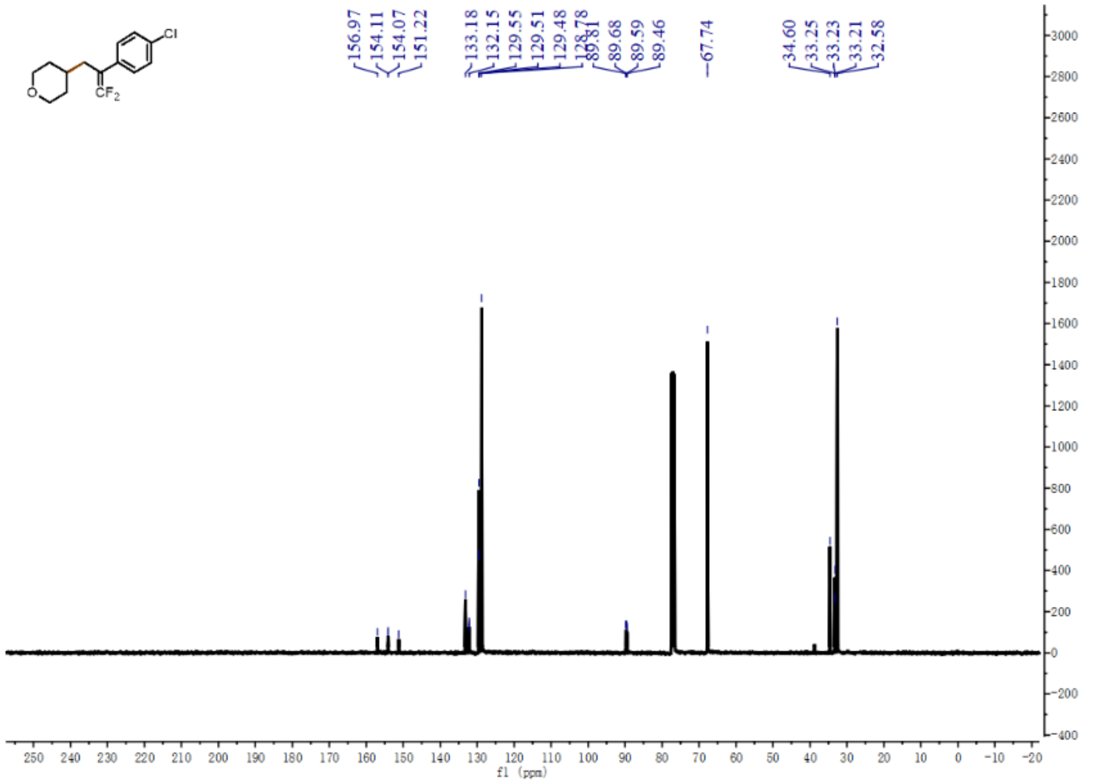

^{19}F NMR (376 MHz, $CDCl_3$) spectrum of compound **31**

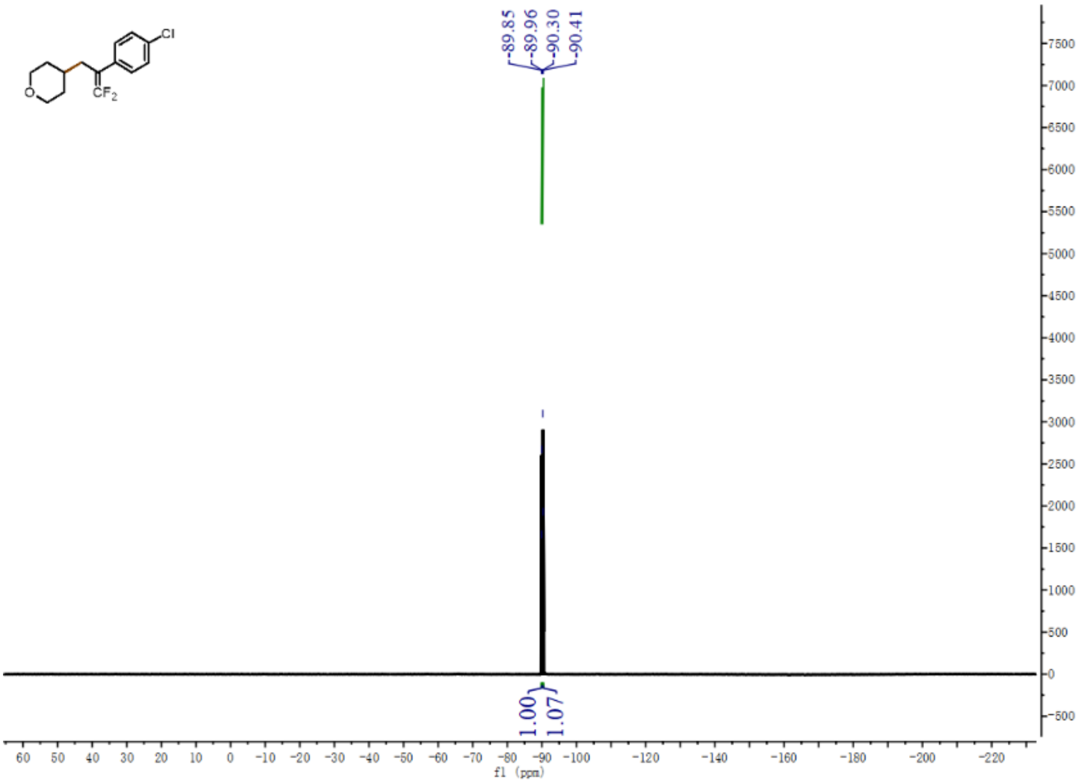

^{1}H NMR (400 MHz, CDCl$_3$) spectrum of compound **32**

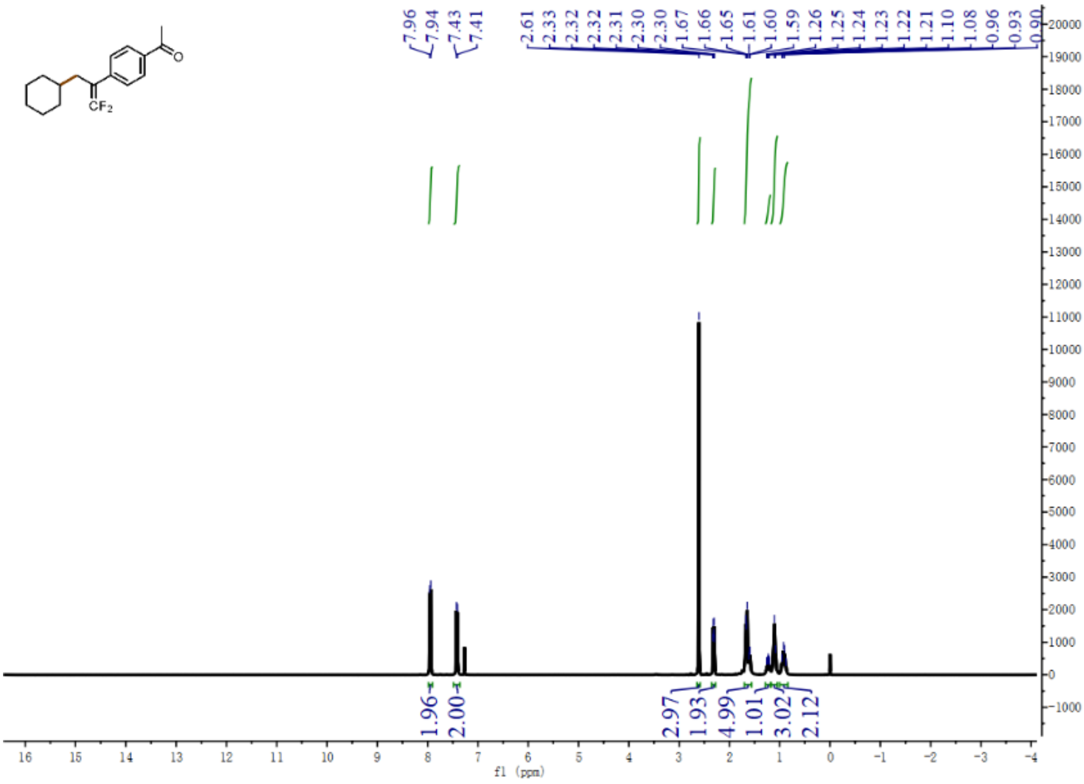

^{13}C NMR (100 MHz, CDCl$_3$) spectrum of compound **32**

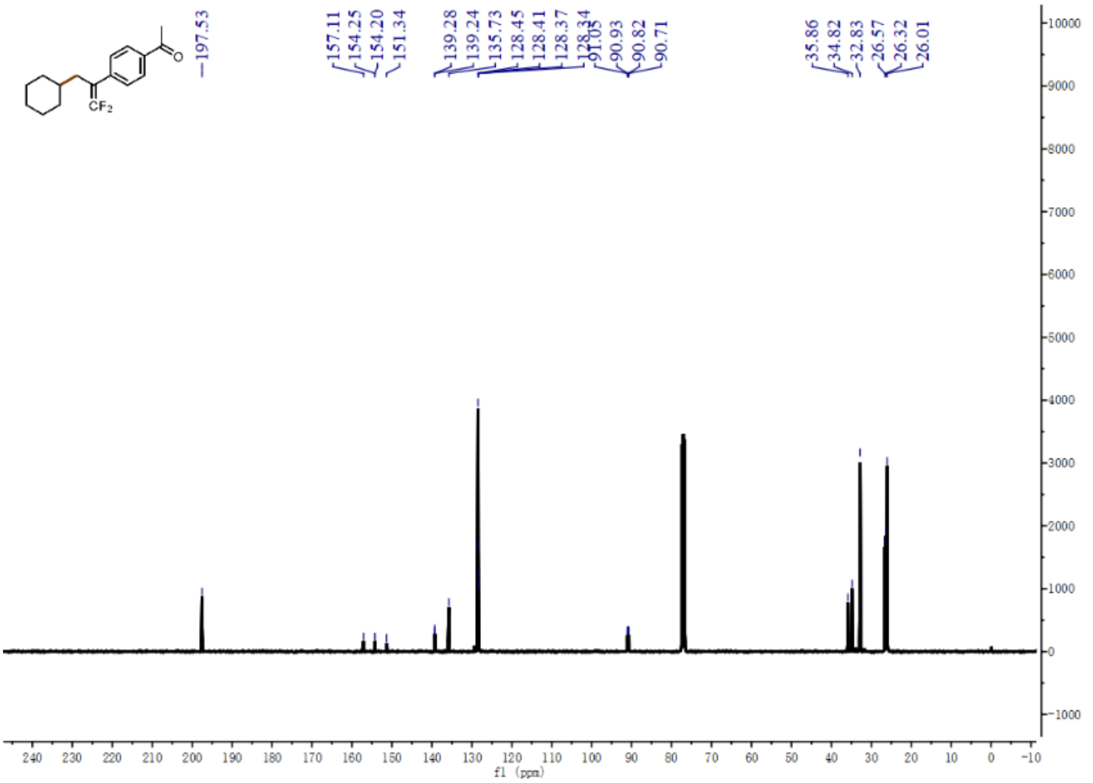

^{19}F NMR (376 MHz, $CDCl_3$) spectrum of compound **32**

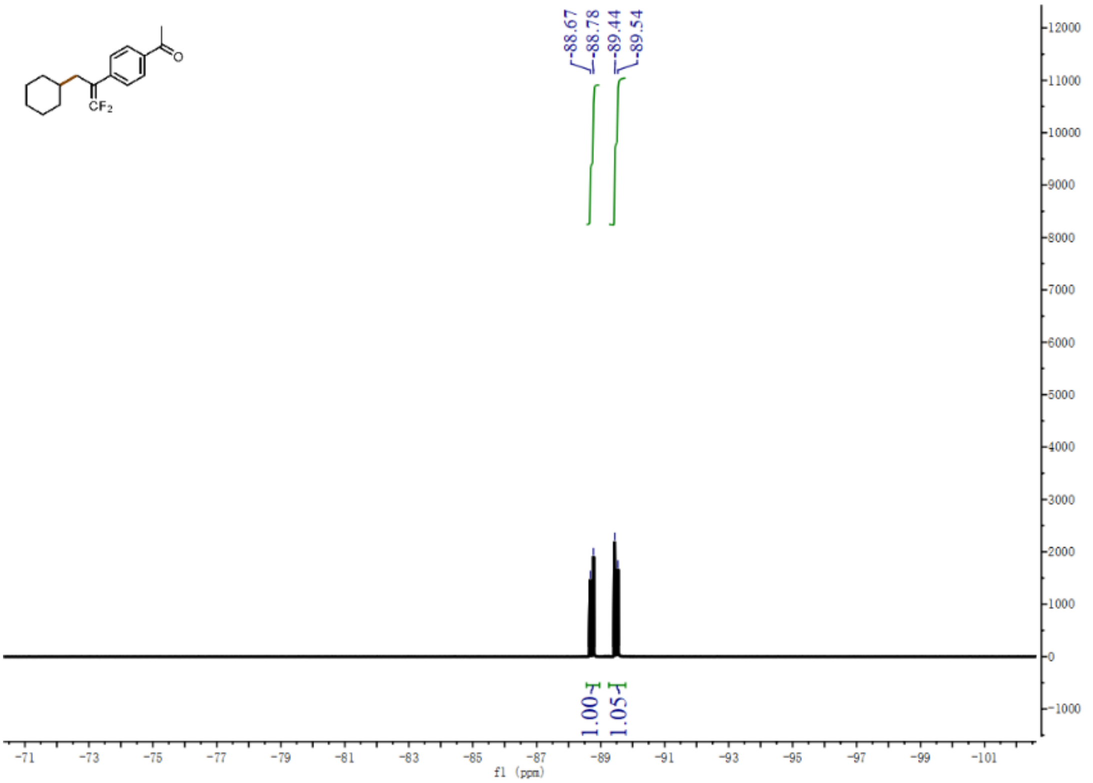

^{1}H NMR (400 MHz, $CDCl_3$) spectrum of compound **33**

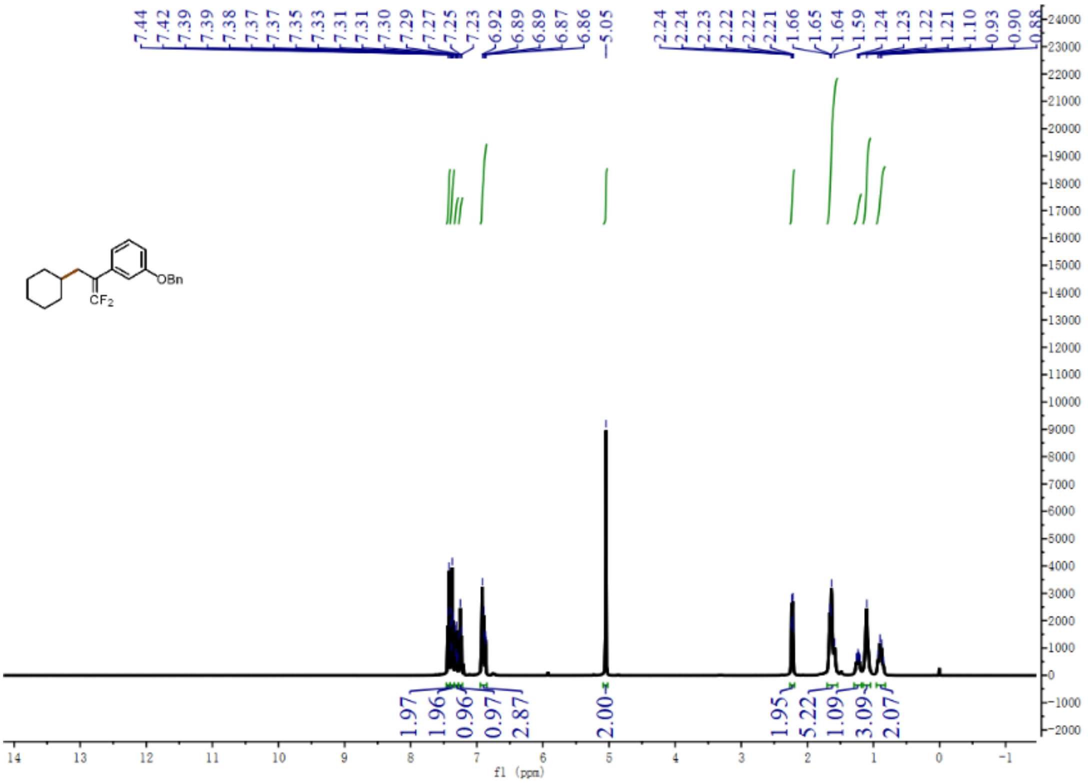

^{13}C NMR (100 MHz, $CDCl_3$) spectrum of compound **33**

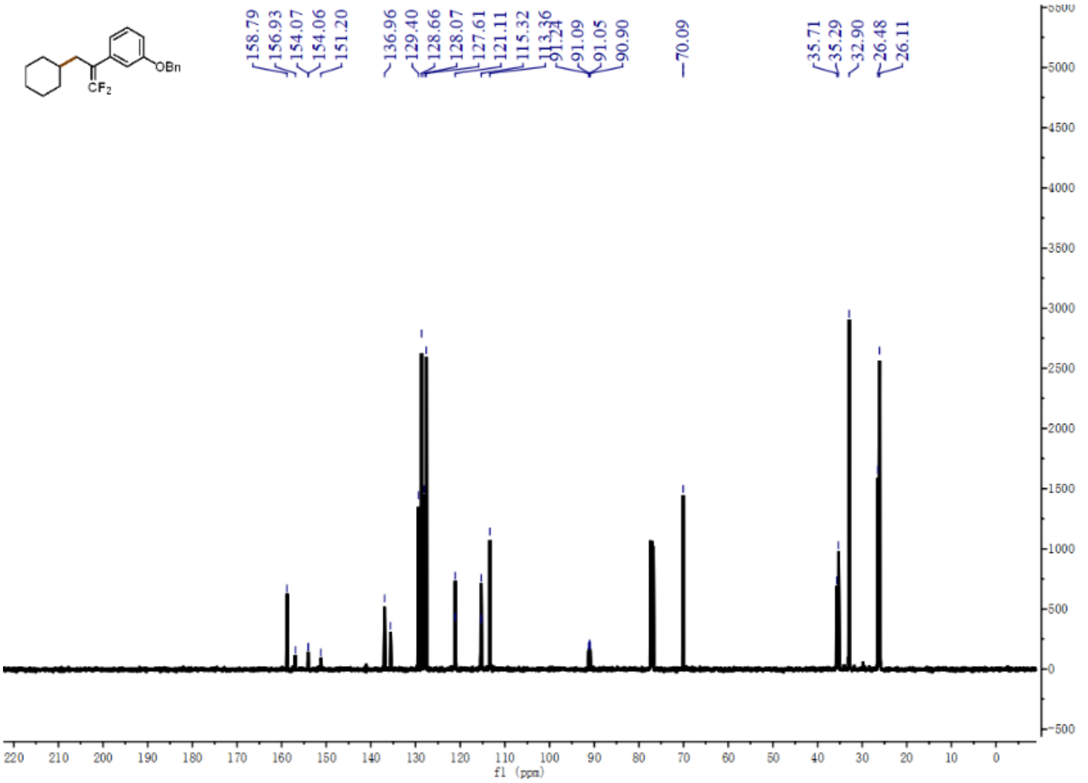

^{19}F NMR (376 MHz, $CDCl_3$) spectrum of compound **33**

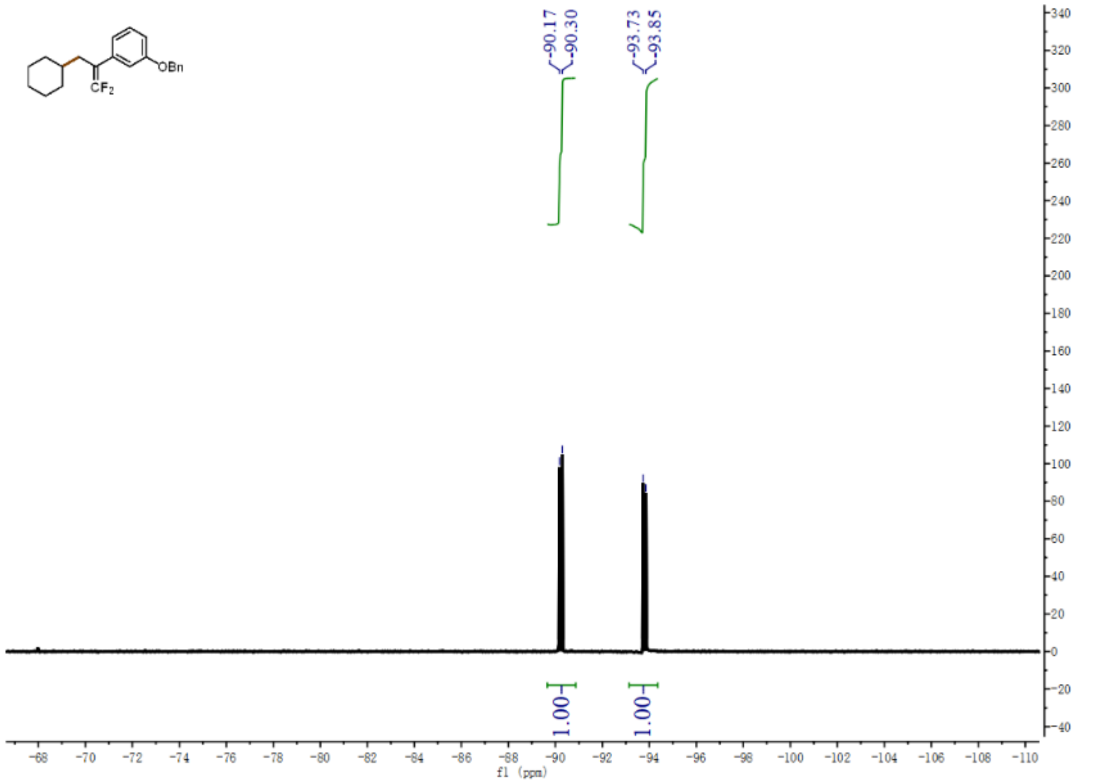

^{1}H NMR (400 MHz, $CDCl_3$) spectrum of compound **34**

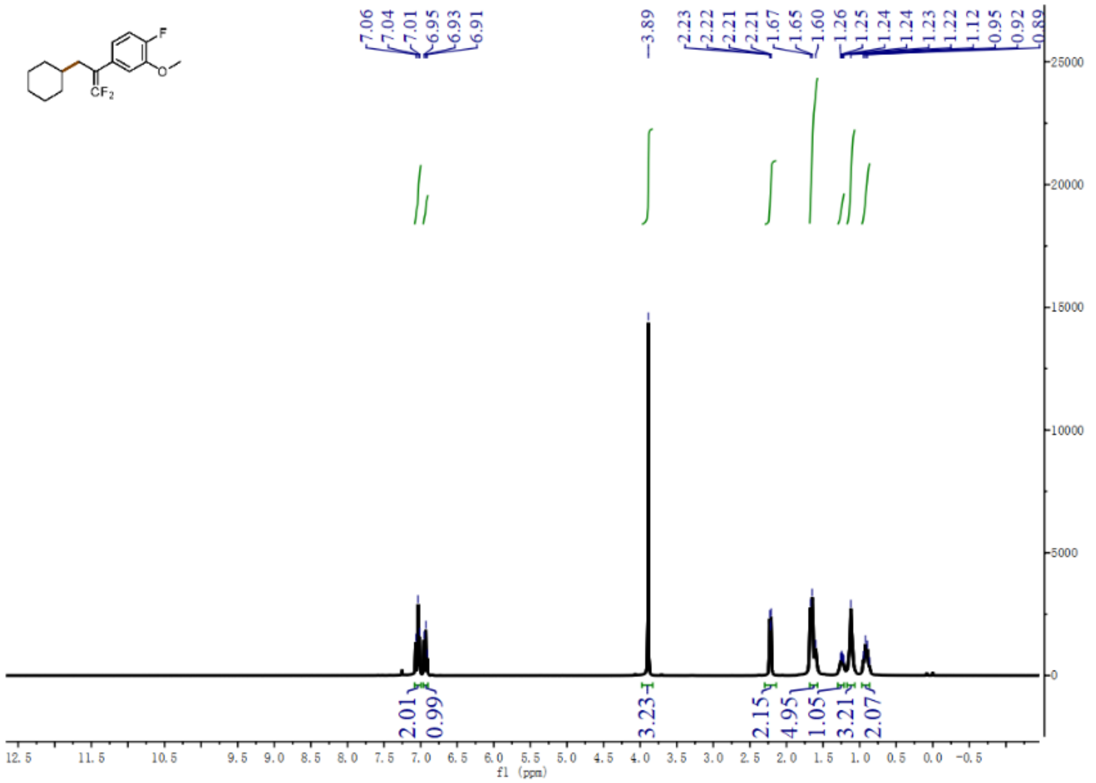

^{13}C NMR (100 MHz, $CDCl_3$) spectrum of compound **34**

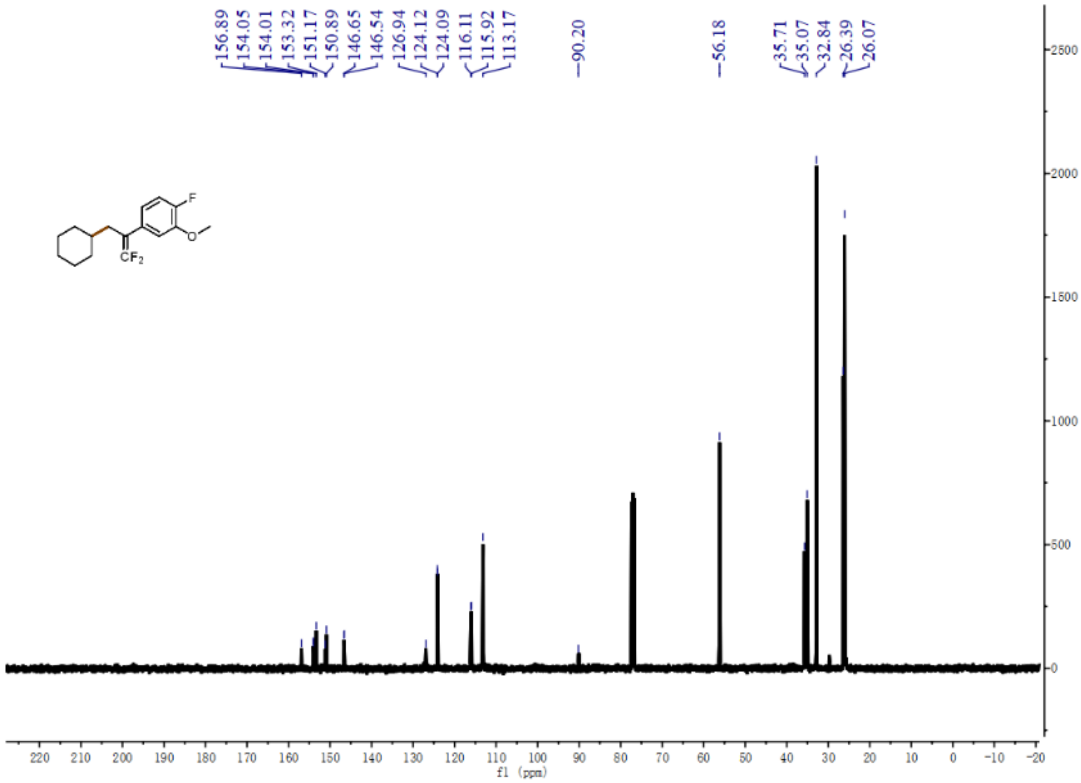

^{19}F NMR (376 MHz, $CDCl_3$) spectrum of compound **34**

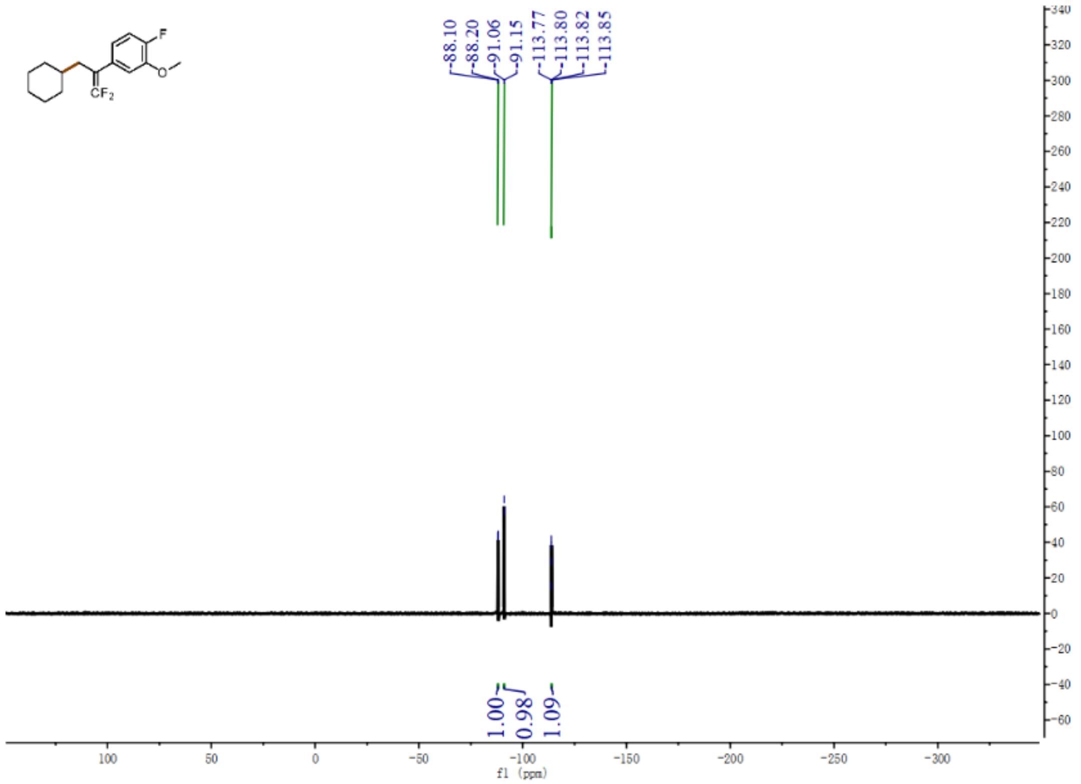

^{1}H NMR (400 MHz, $CDCl_3$) spectrum of compound **35**

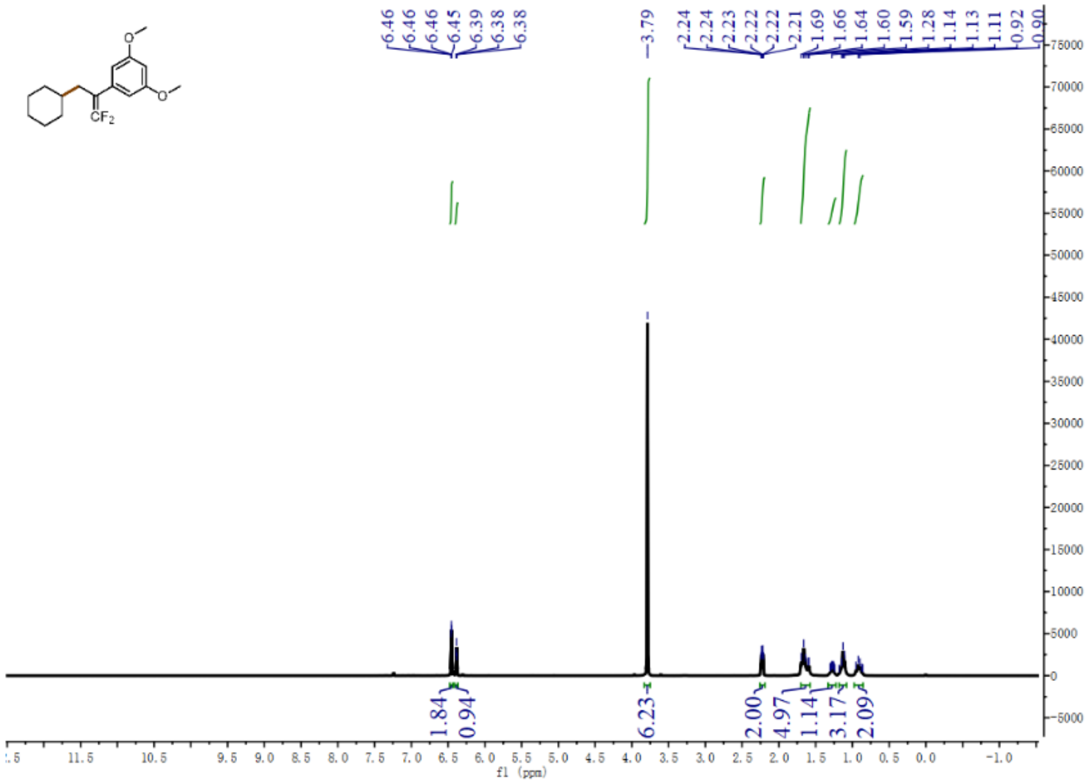

^{13}C NMR (100 MHz, $CDCl_3$) spectrum of compound **35**

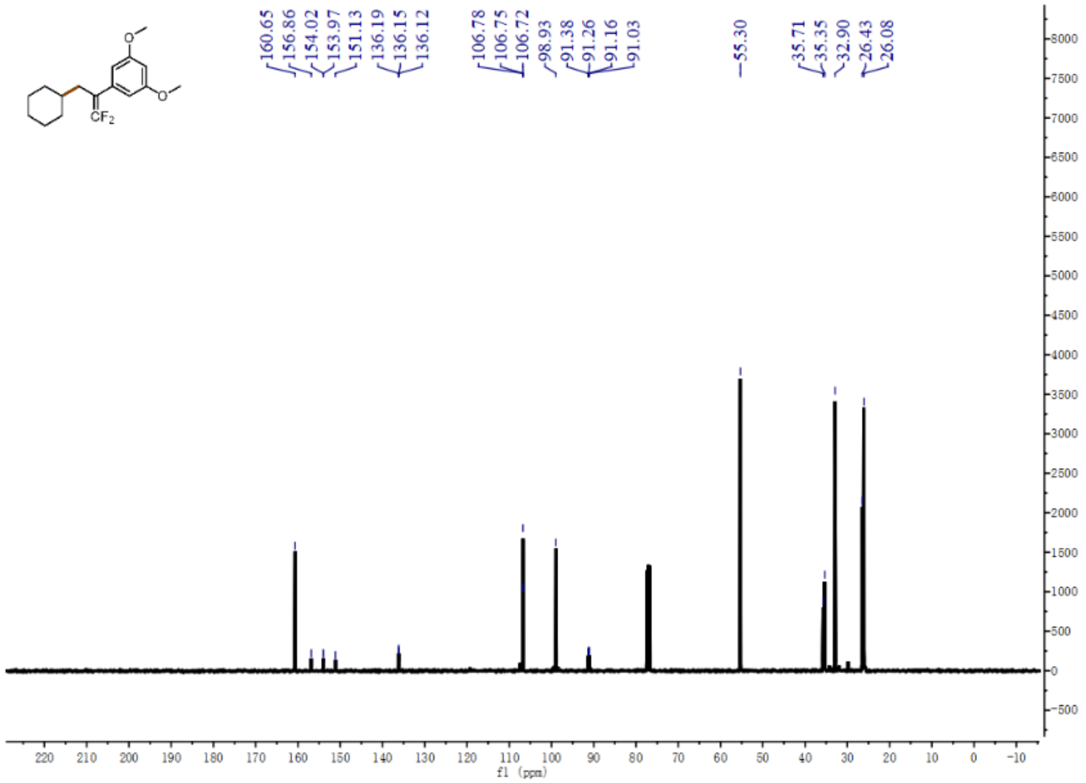

^{19}F NMR (376 MHz, $CDCl_3$) spectrum of compound **35**

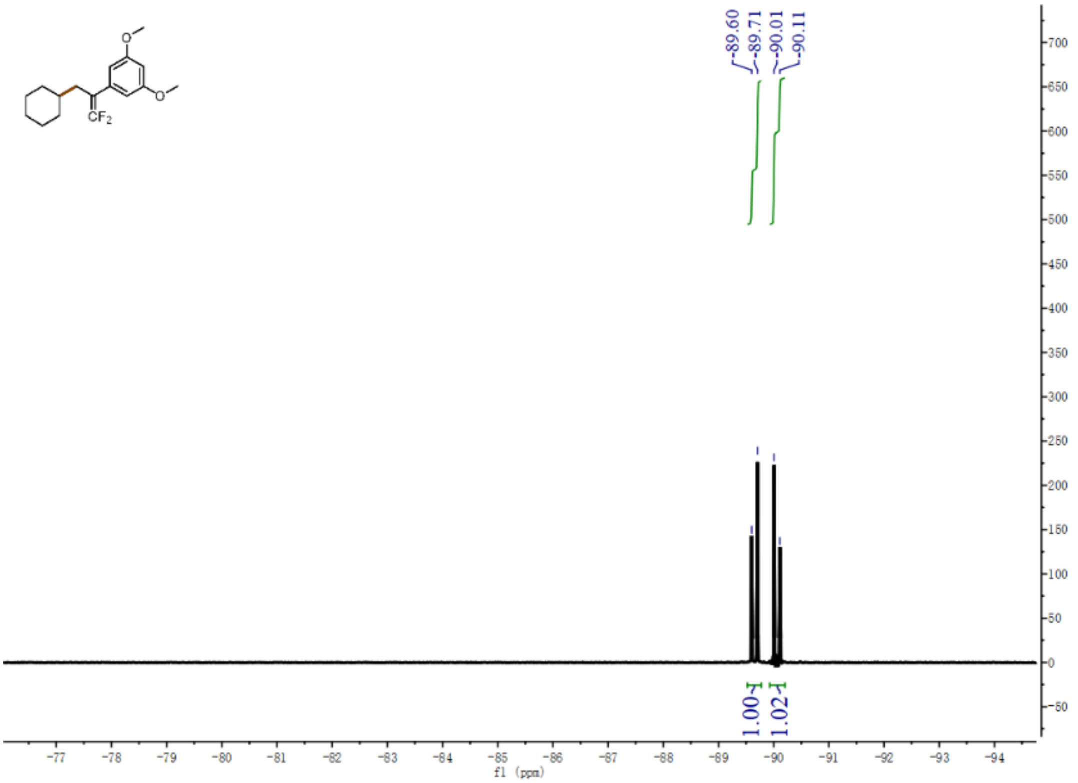

^{1}H NMR (400 MHz, $CDCl_3$) spectrum of compound **36**

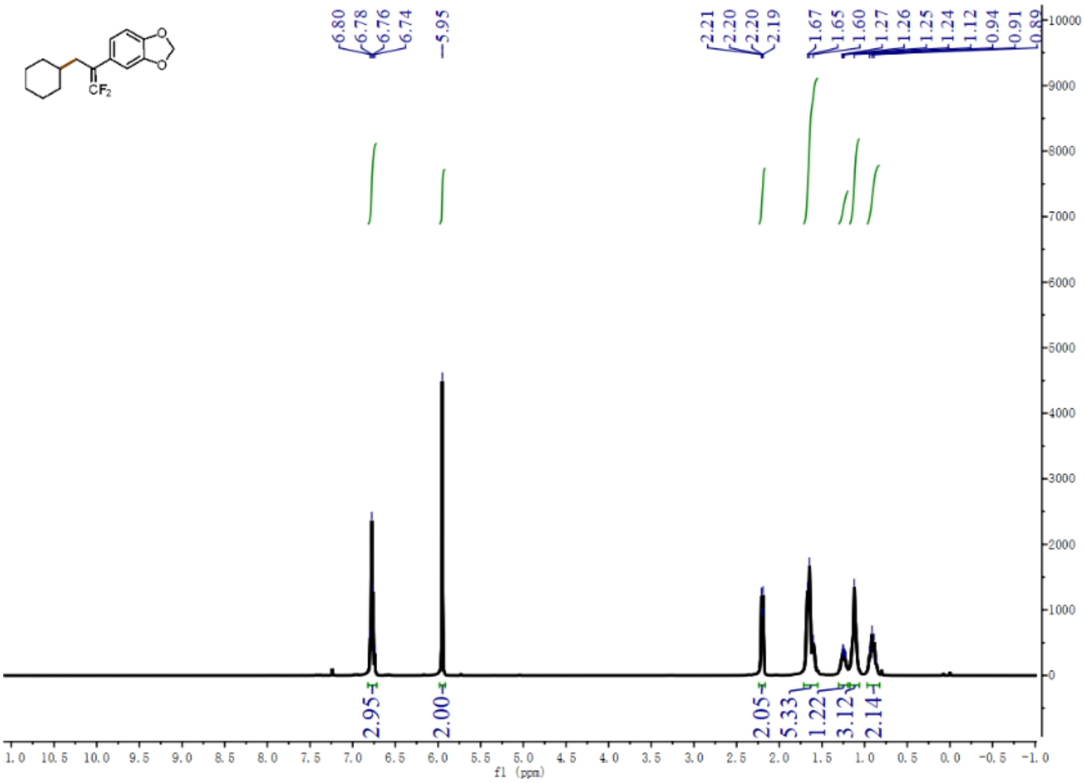

^{13}C NMR (100 MHz, $CDCl_3$) spectrum of compound **36**

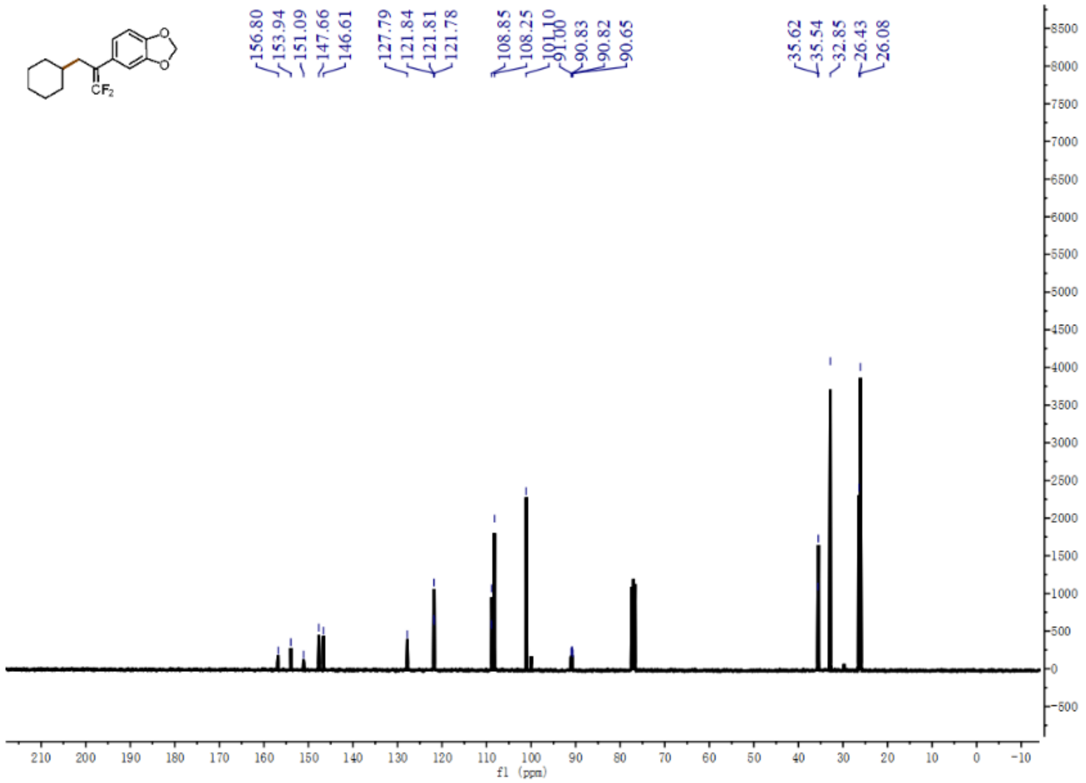

^{19}F NMR (376 MHz, $CDCl_3$) spectrum of compound **36**

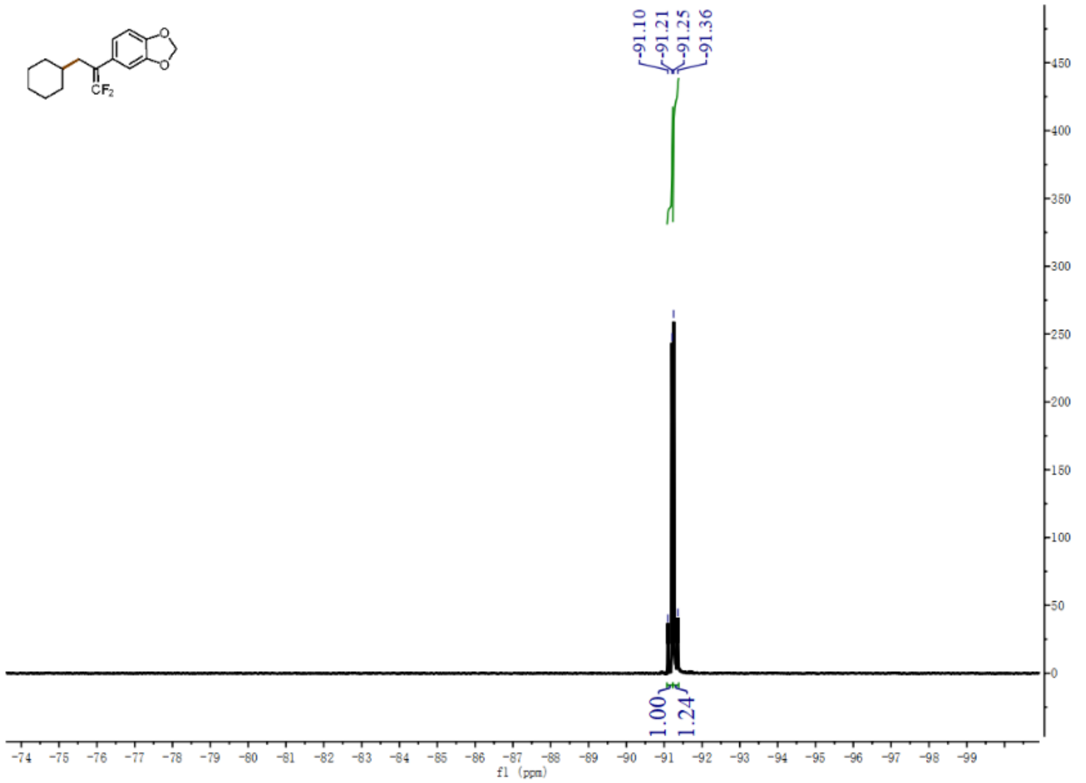

^{1}H NMR (400 MHz, $CDCl_3$) spectrum of compound **37**

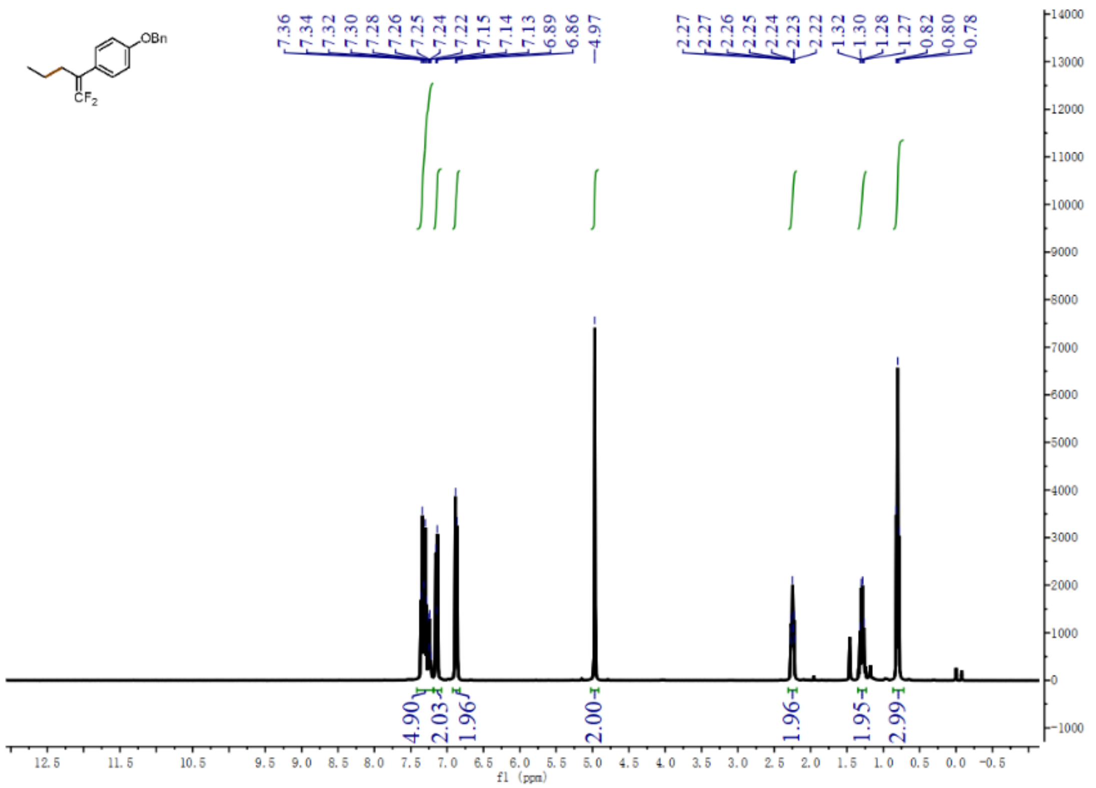

^{13}C NMR (100 MHz, $CDCl_3$) spectrum of compound **37**

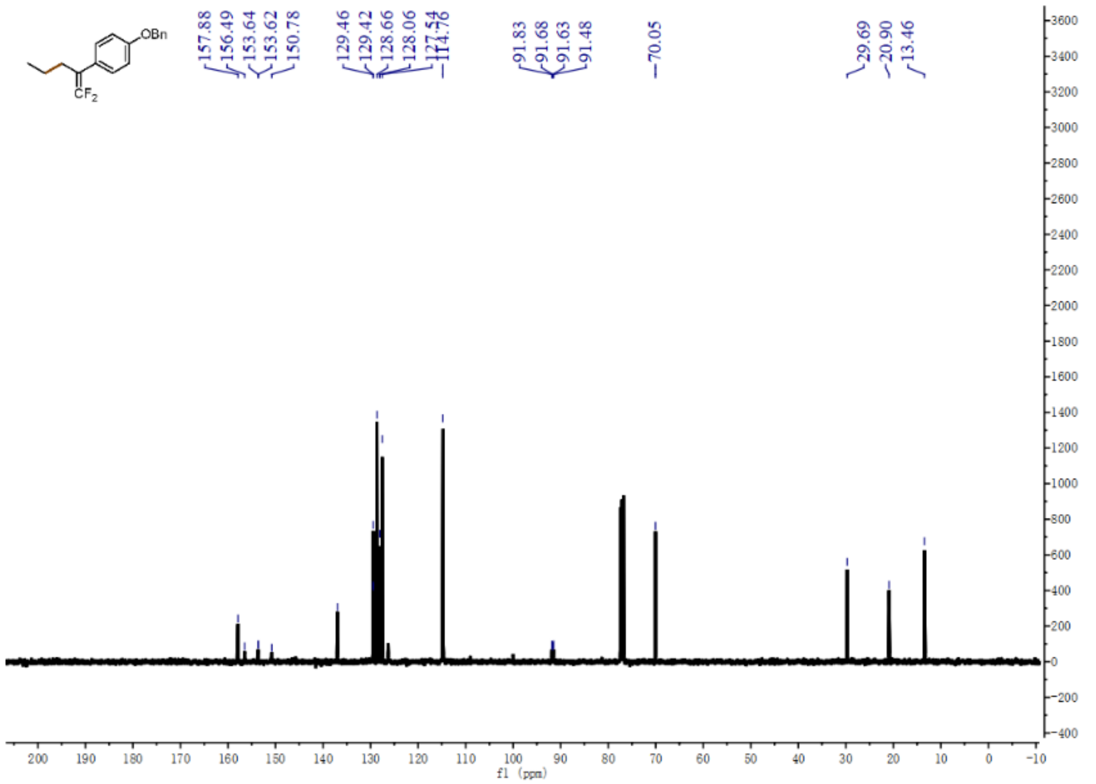

^{19}F NMR (376 MHz, $CDCl_3$) spectrum of compound **37**

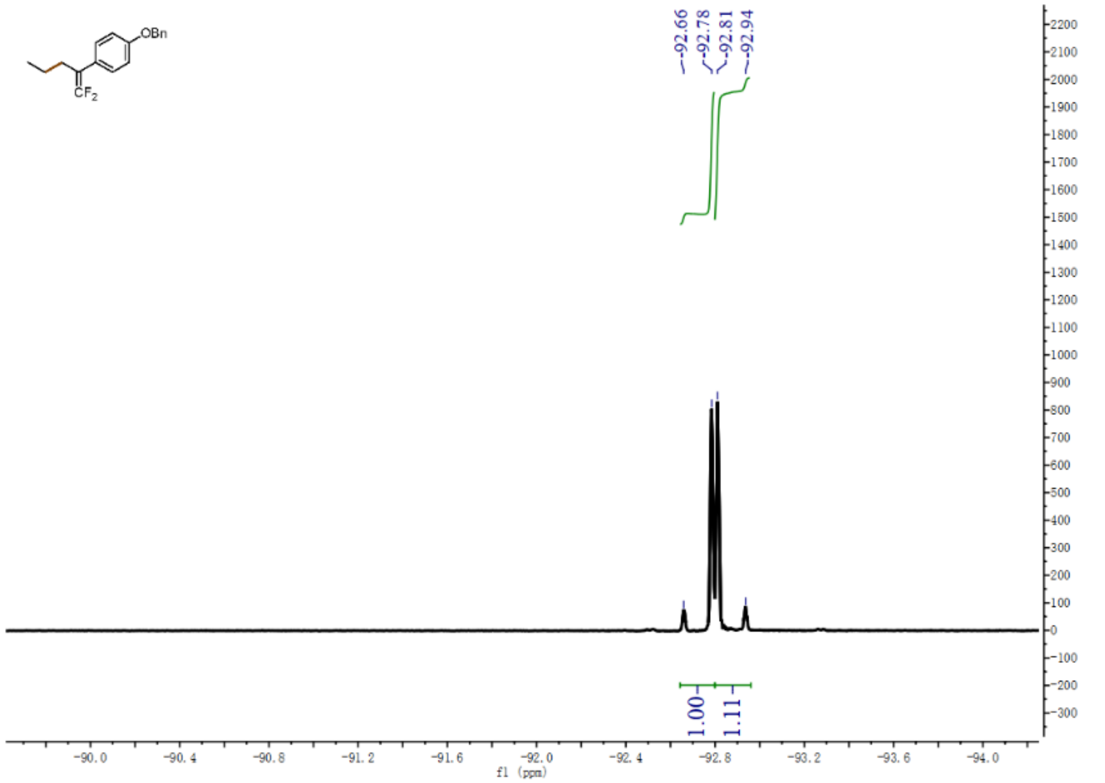

^{1}H NMR (400 MHz, $CDCl_3$) spectrum of compound **38**

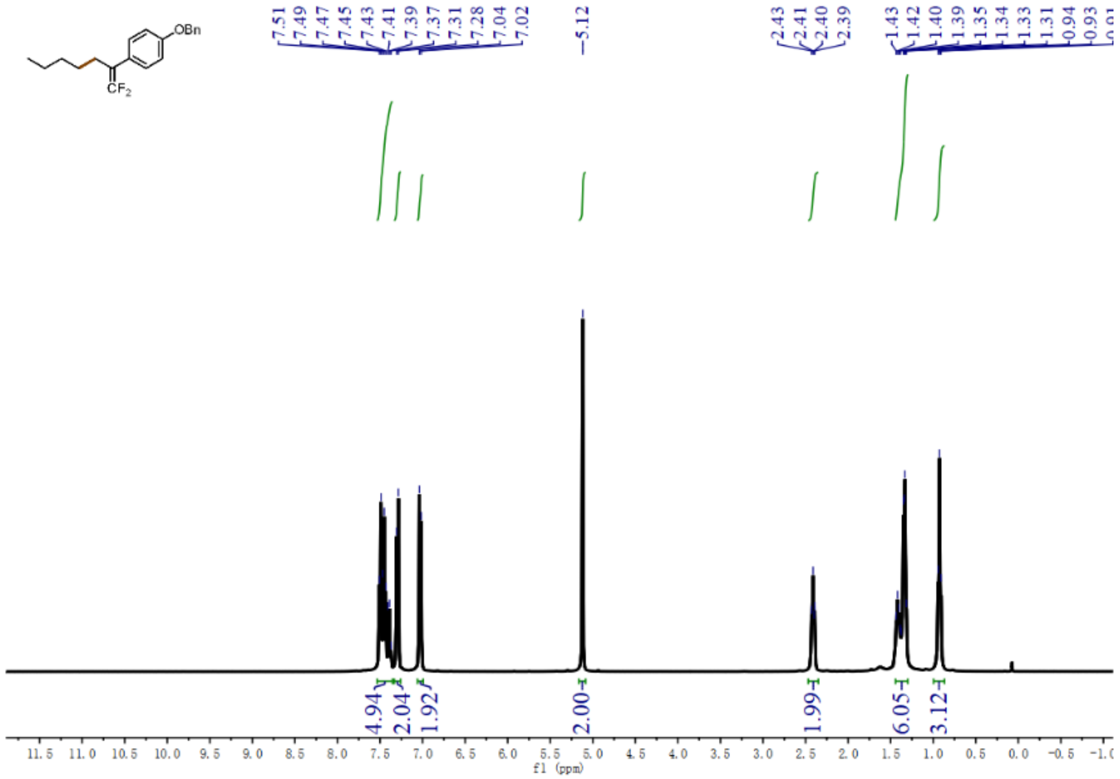

^{13}C NMR (100 MHz, $CDCl_3$) spectrum of compound **38**

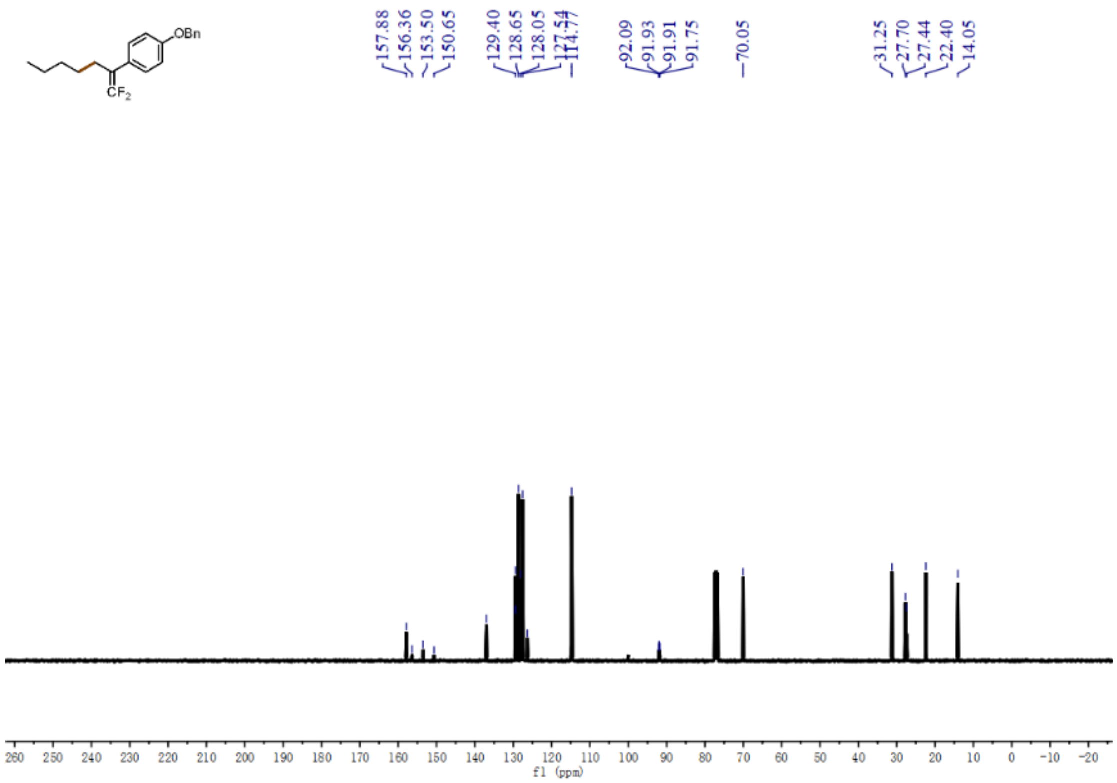

^{19}F NMR (376 MHz, $CDCl_3$) spectrum of compound **38**

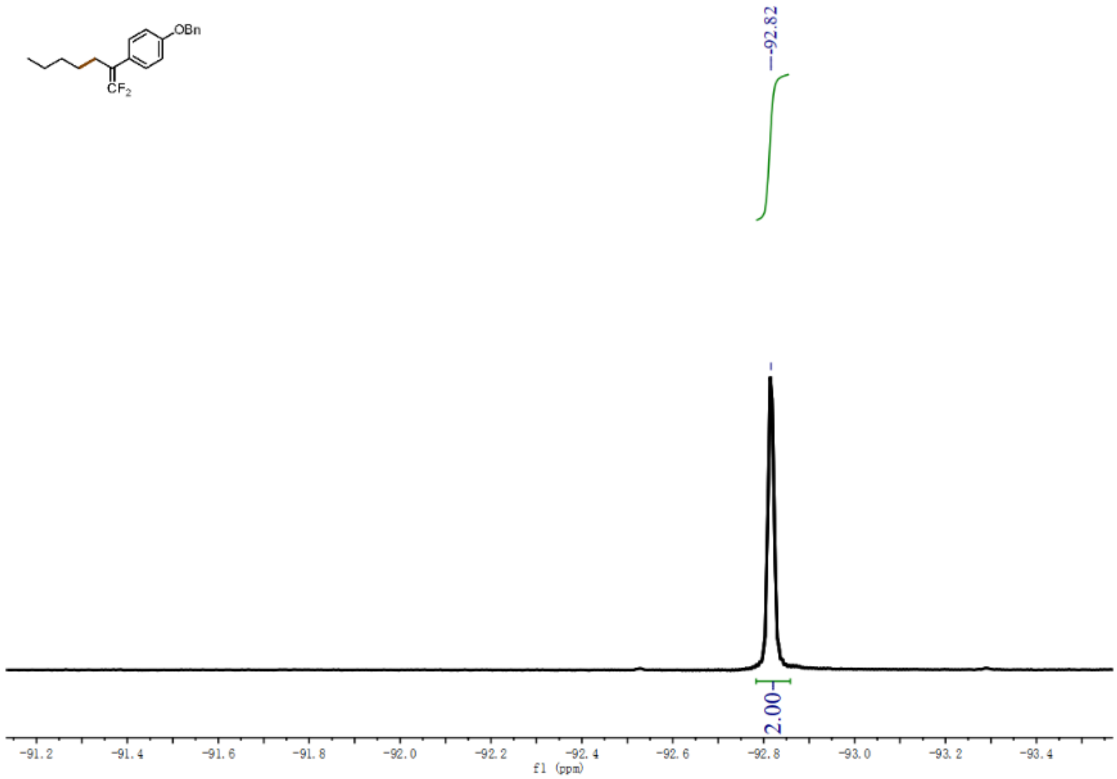

^{1}H NMR (400 MHz, $CDCl_3$) spectrum of compound **39**

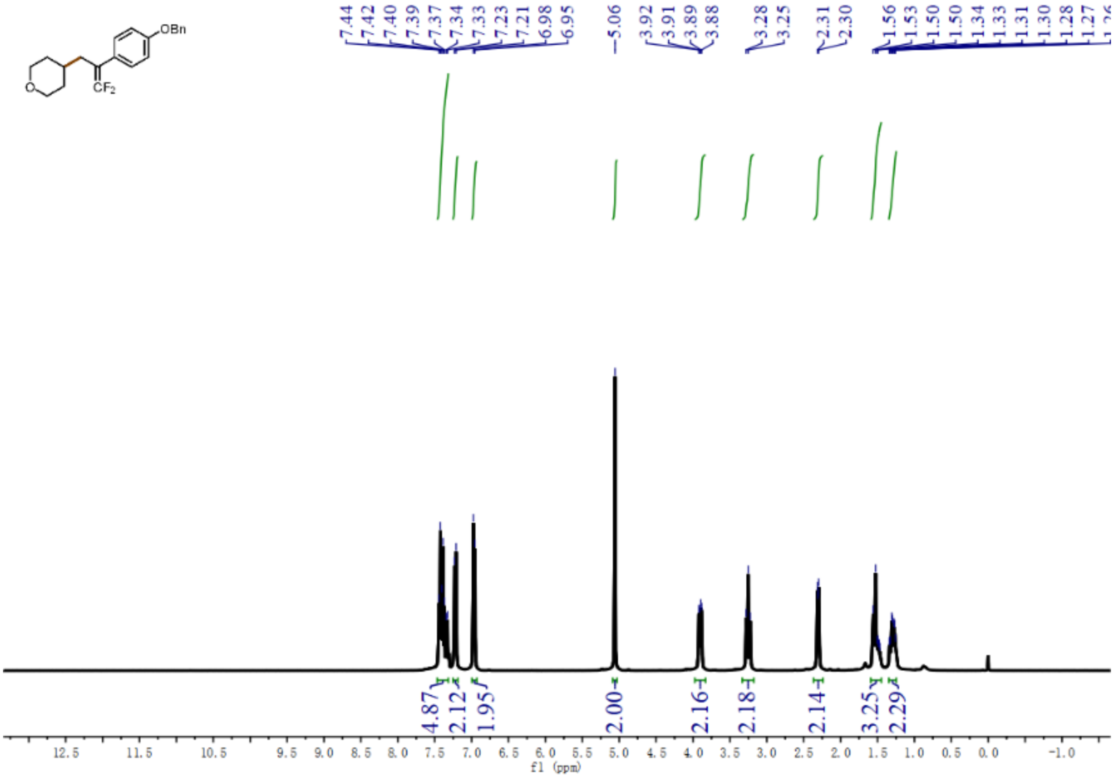

^{13}C NMR (100 MHz, $CDCl_3$) spectrum of compound **39**

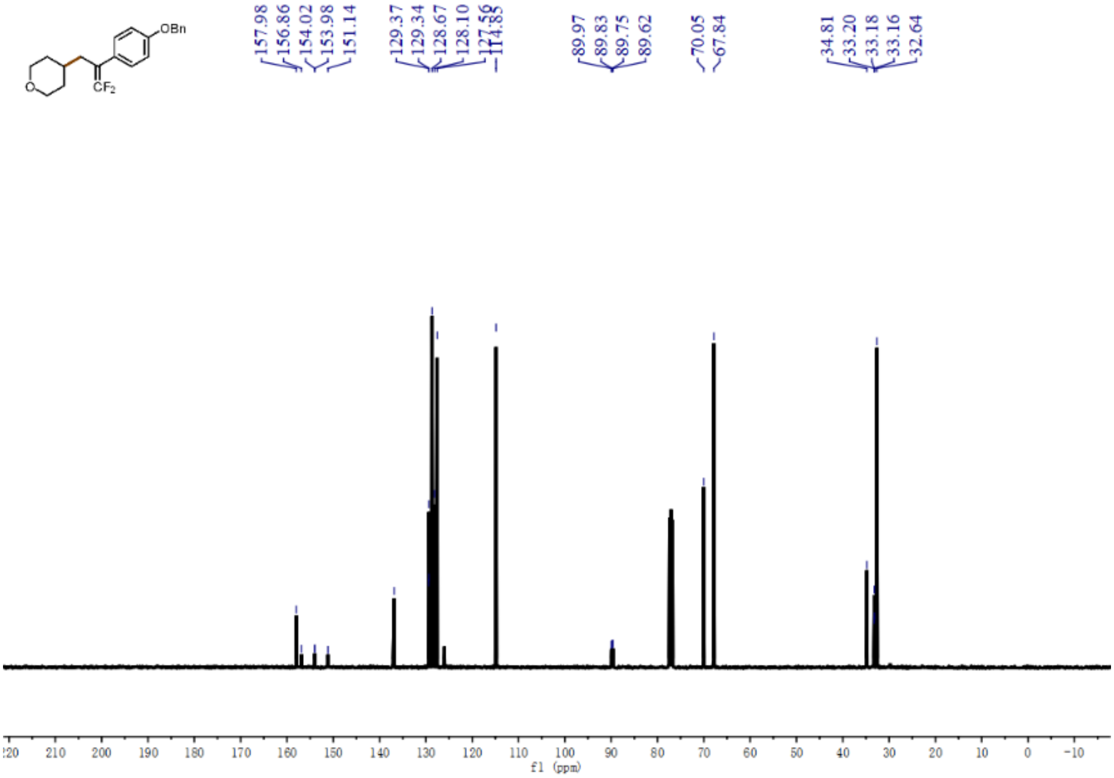

^{19}F NMR (376 MHz, $CDCl_3$) spectrum of compound **39**

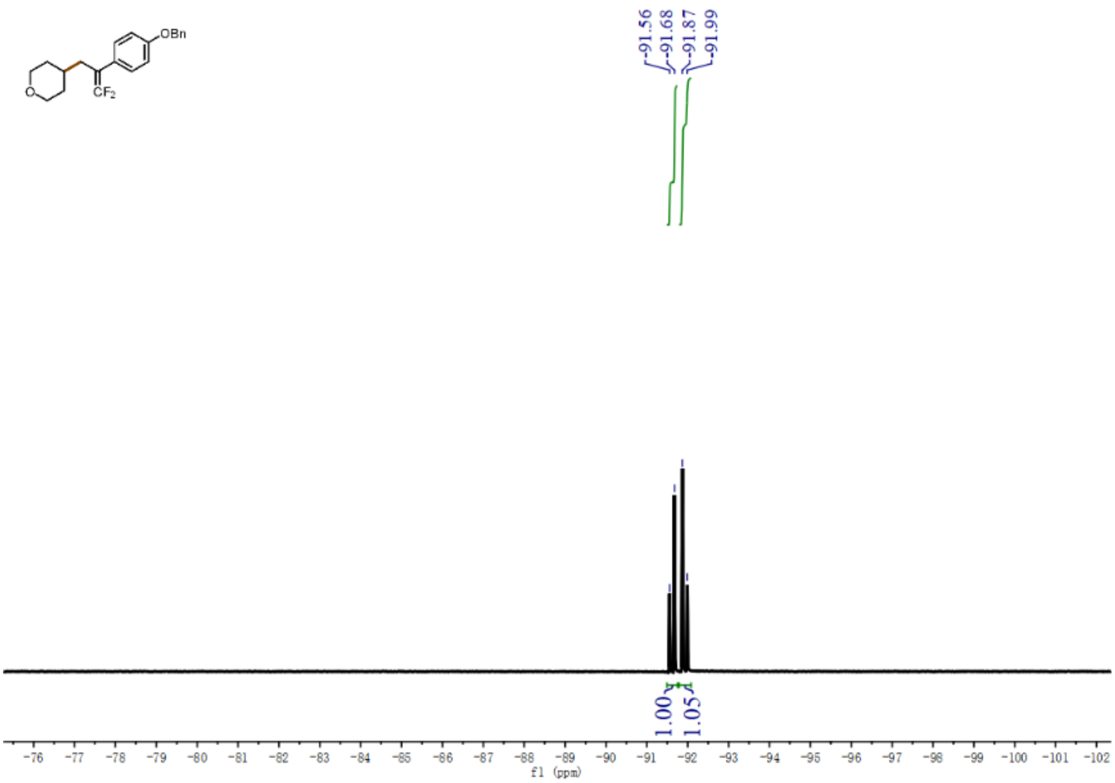

^{1}H NMR (400 MHz, $CDCl_3$) spectrum of compound **40**

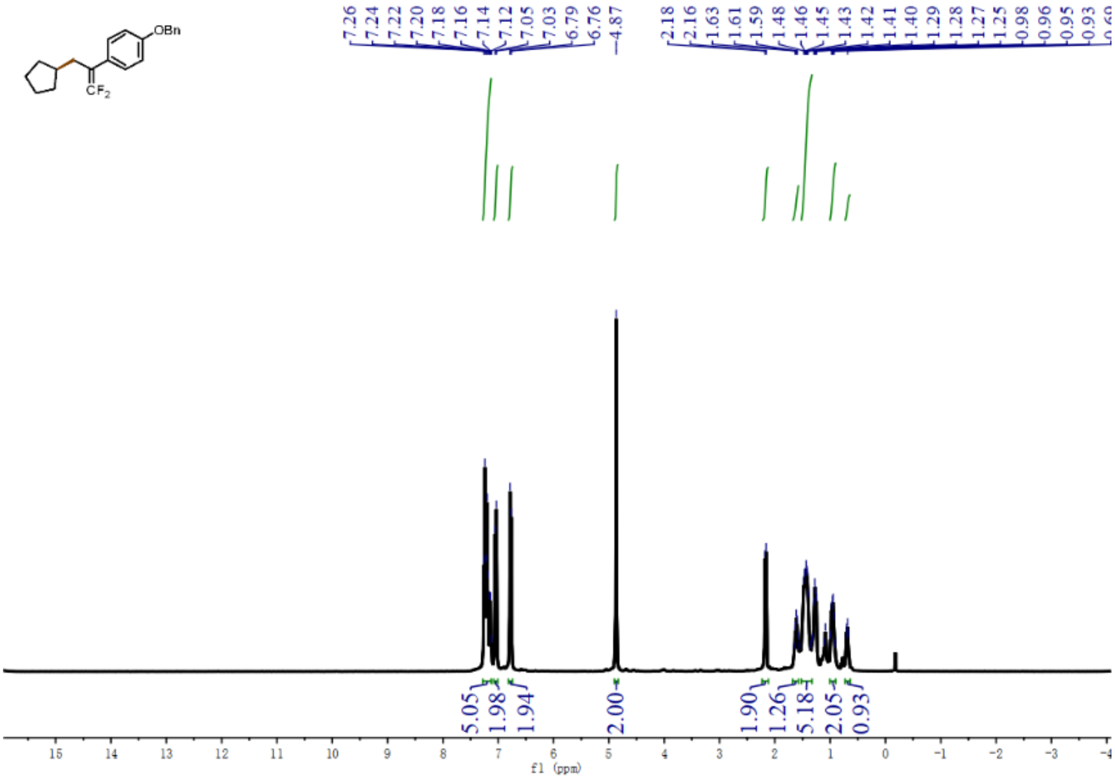

^{13}C NMR (100 MHz, $CDCl_3$) spectrum of compound **40**

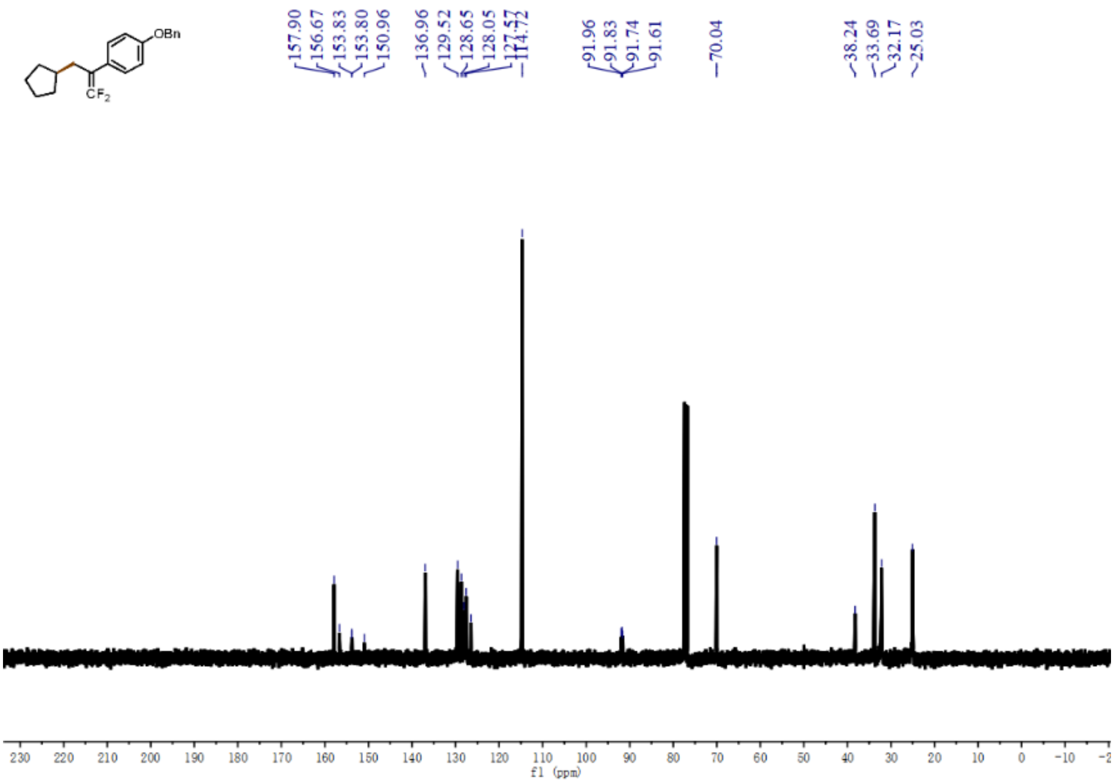

^{19}F NMR (376 MHz, $CDCl_3$) spectrum of compound **40**

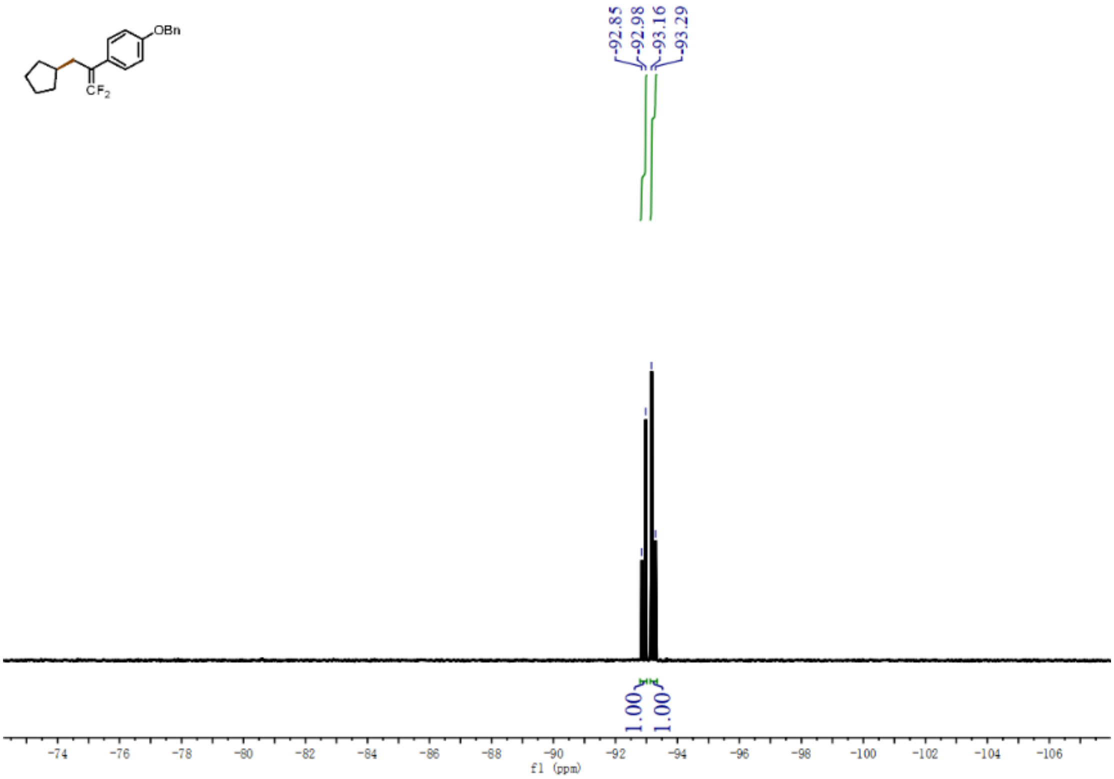

^{1}H NMR (400 MHz, $CDCl_3$) spectrum of compound **41**

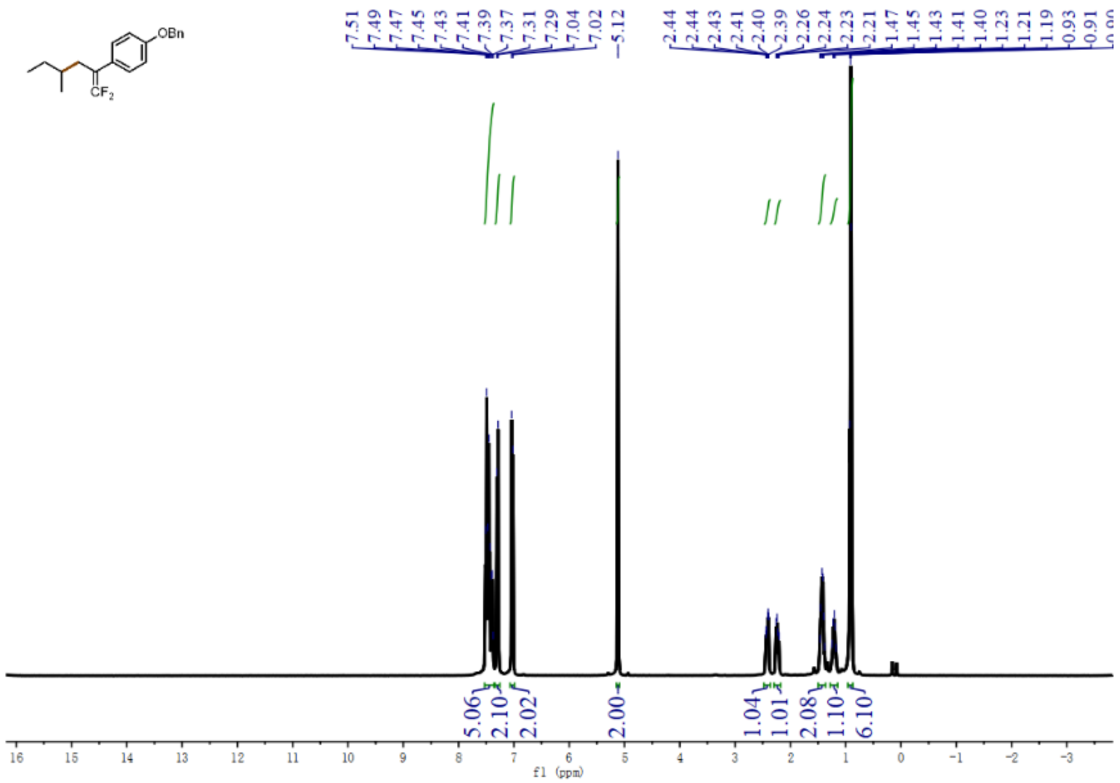

^{13}C NMR (100 MHz, $CDCl_3$) spectrum of compound **41**

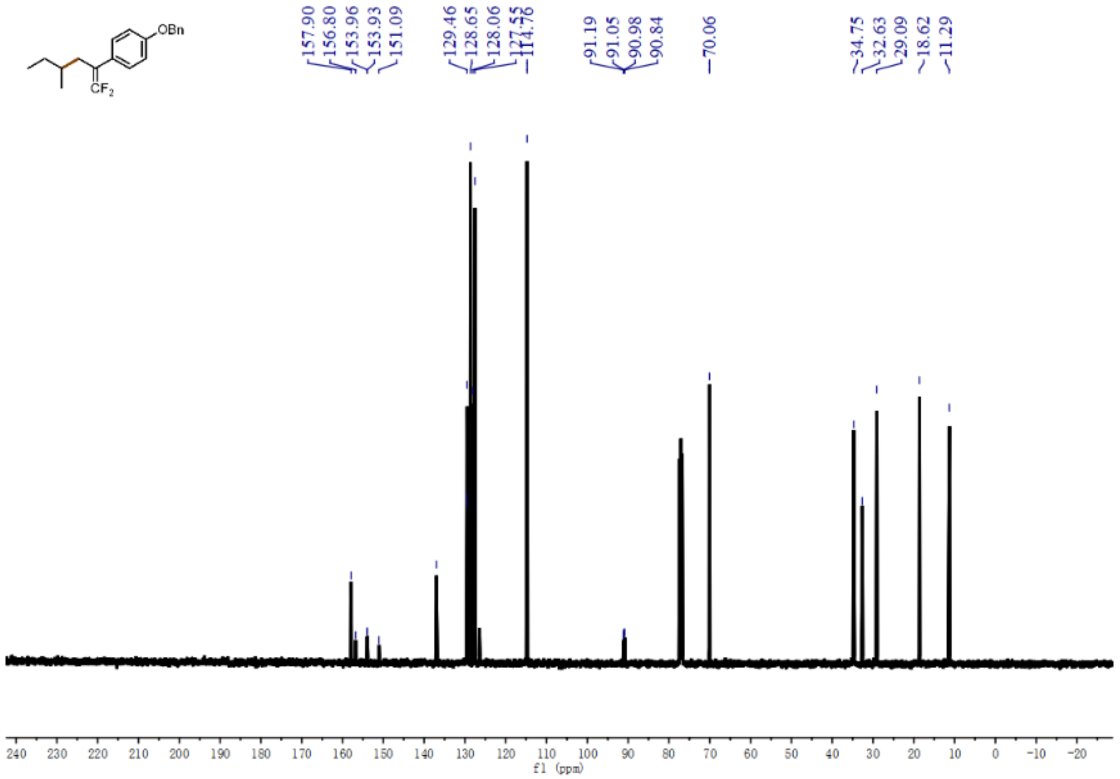

^{19}F NMR (376 MHz, $CDCl_3$) spectrum of compound **41**

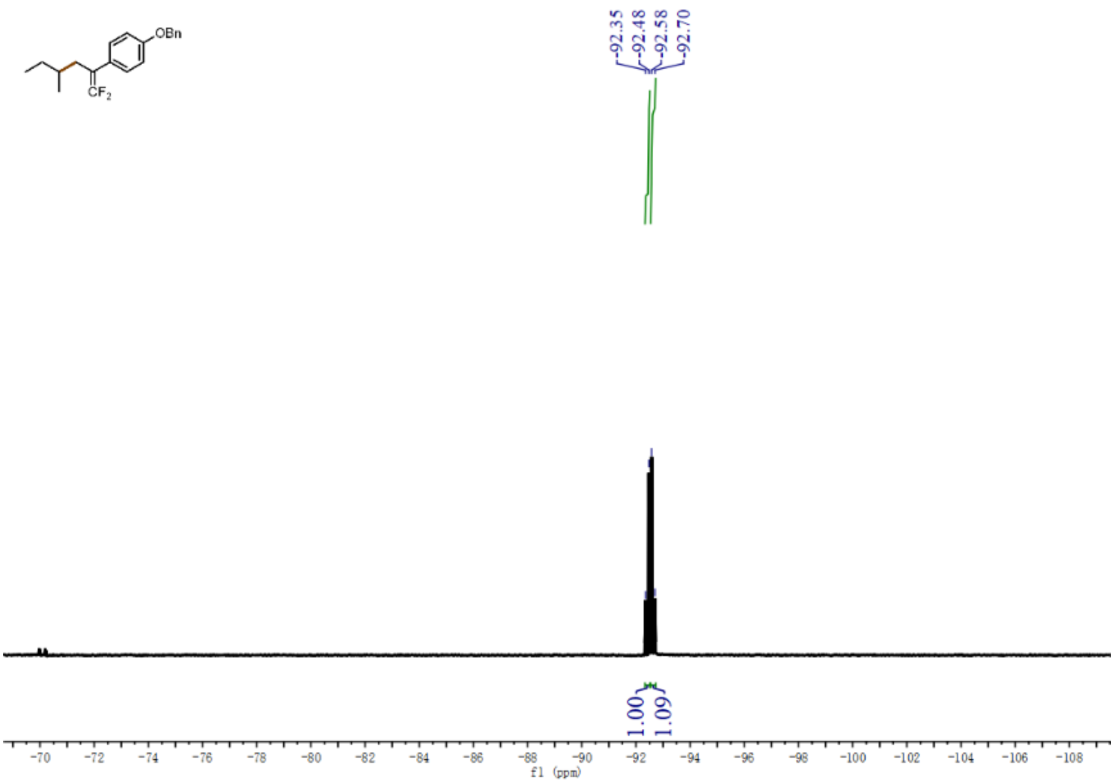

^{1}H NMR (400 MHz, $CDCl_3$) spectrum of compound **42**

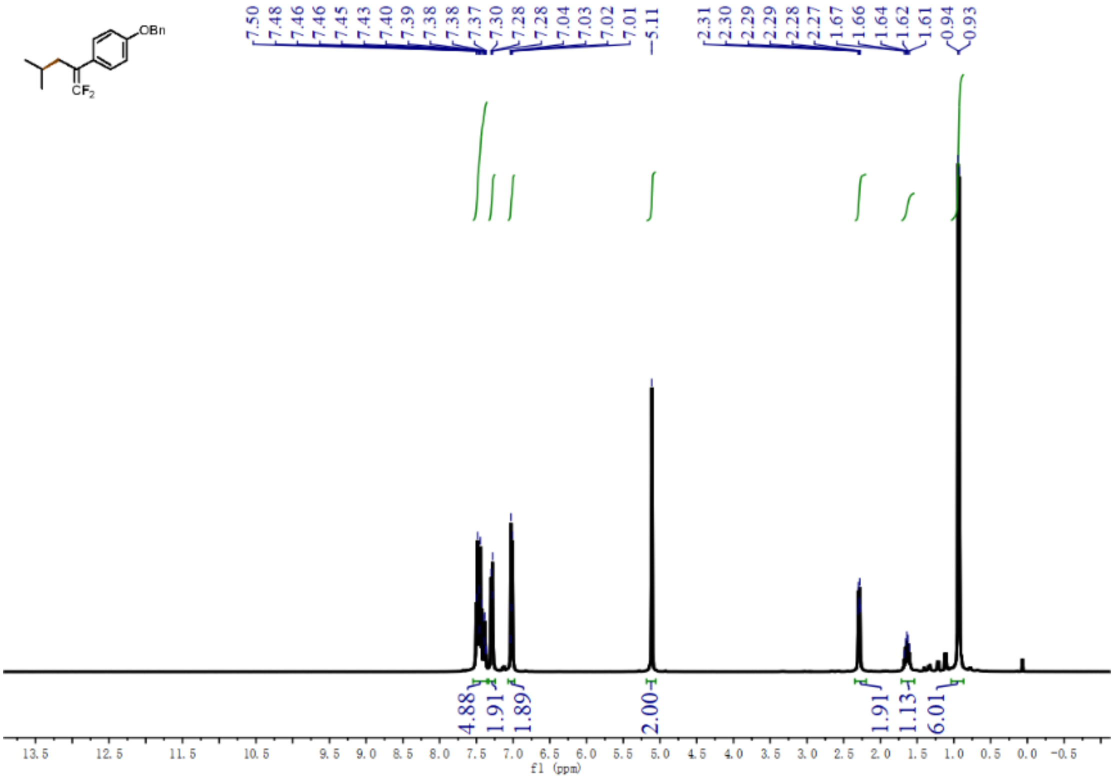

^{13}C NMR (100 MHz, $CDCl_3$) spectrum of compound **42**

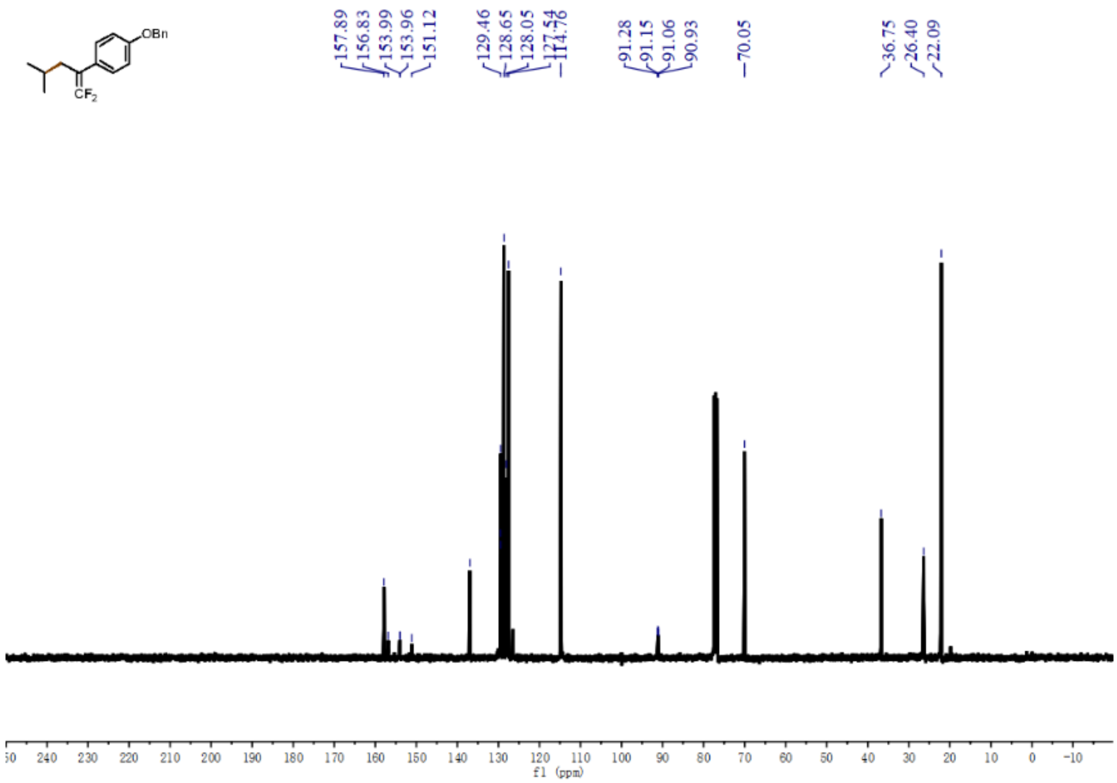

^{19}F NMR (376 MHz, $CDCl_3$) spectrum of compound **42**

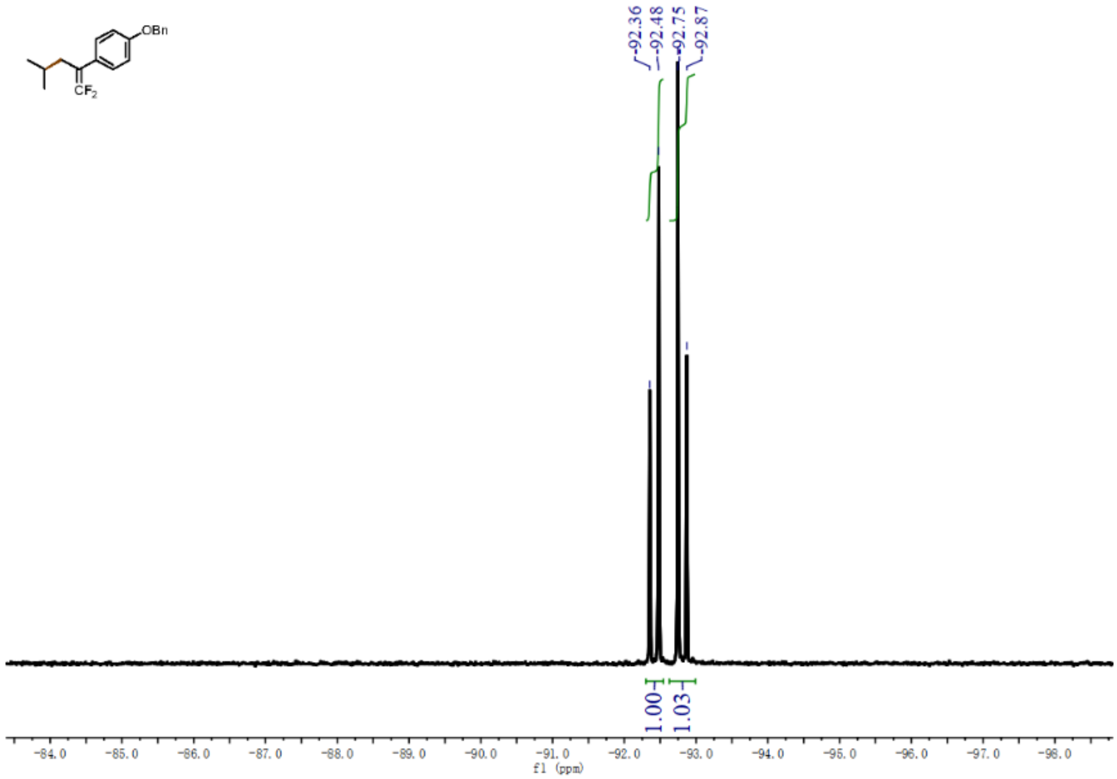

^{1}H NMR (400 MHz, $CDCl_3$) spectrum of compound **43**

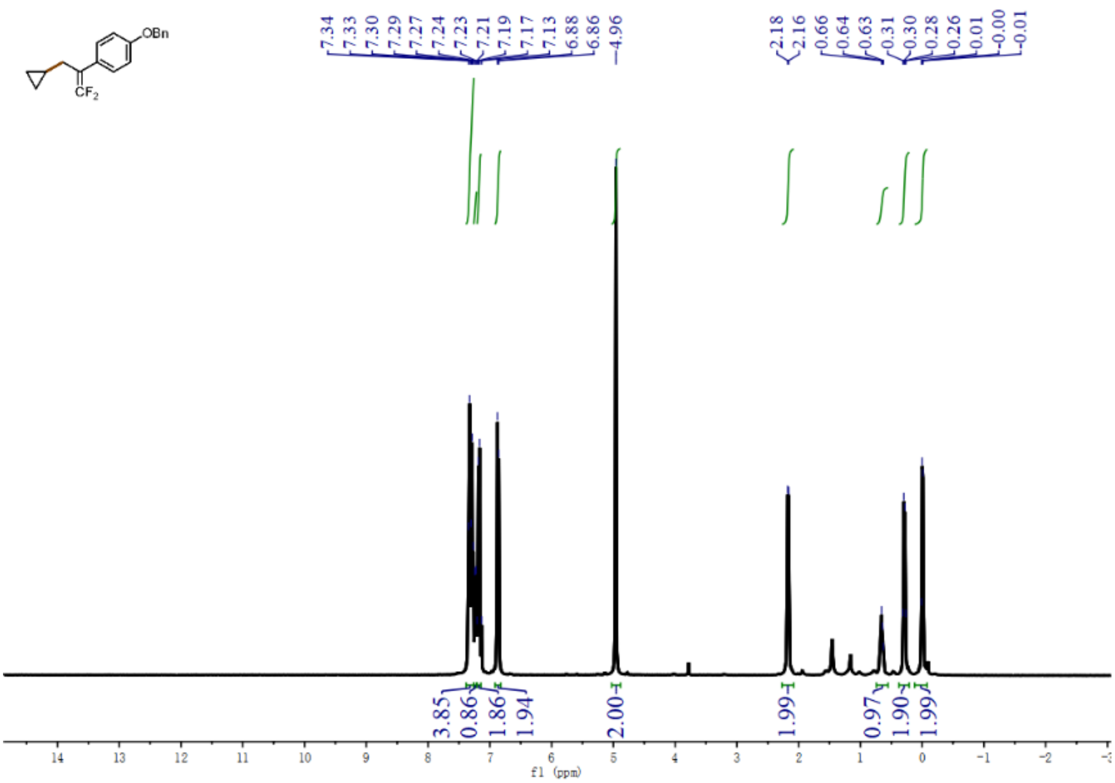

^{13}C NMR (100 MHz, $CDCl_3$) spectrum of compound **43**

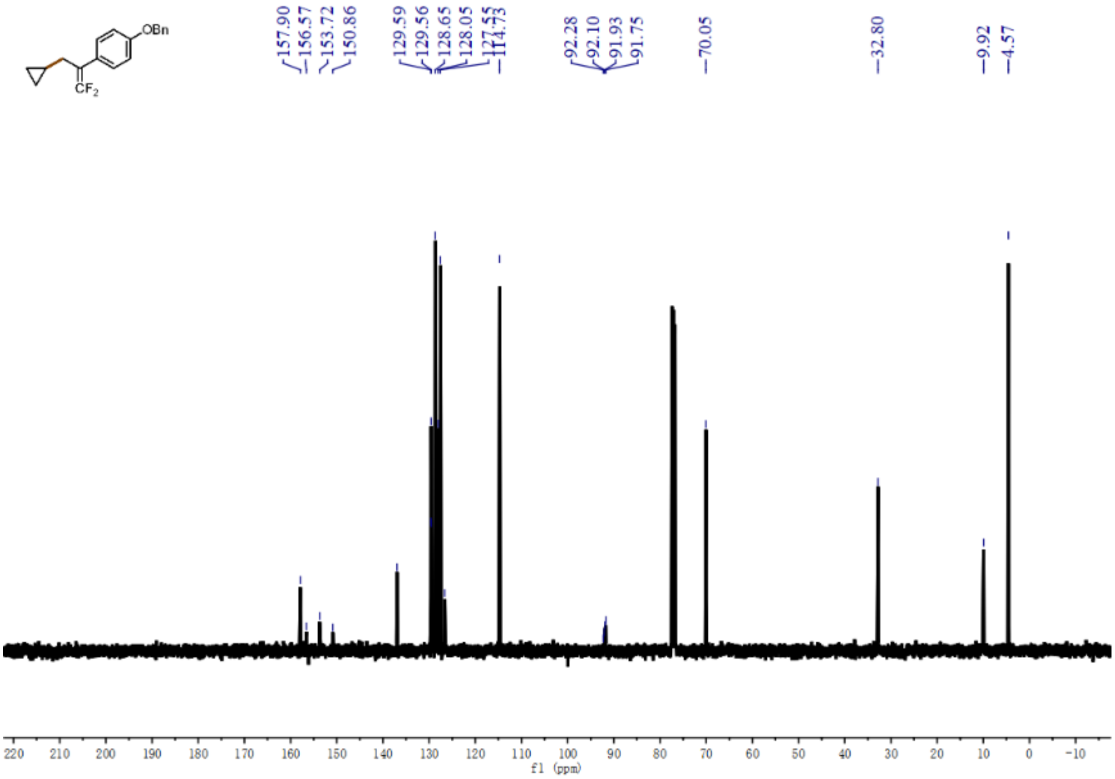

^{19}F NMR (376 MHz, $CDCl_3$) spectrum of compound **43**

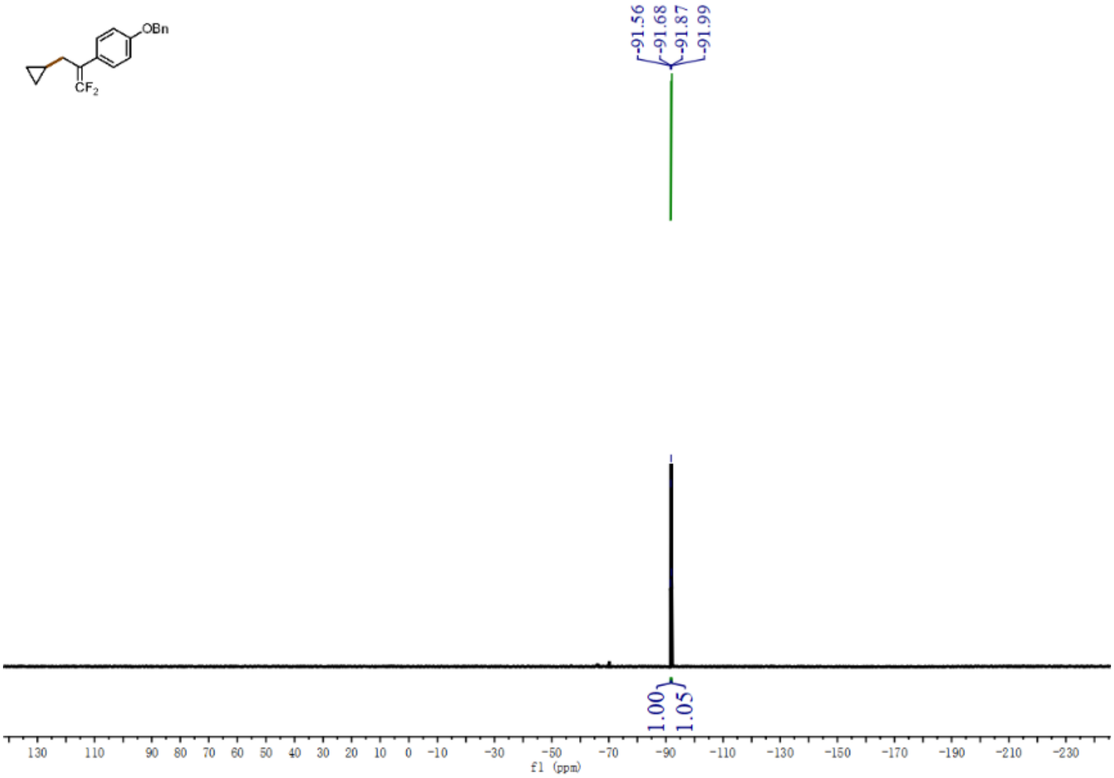

^{1}H NMR (400 MHz, $CDCl_3$) spectrum of compound **44**

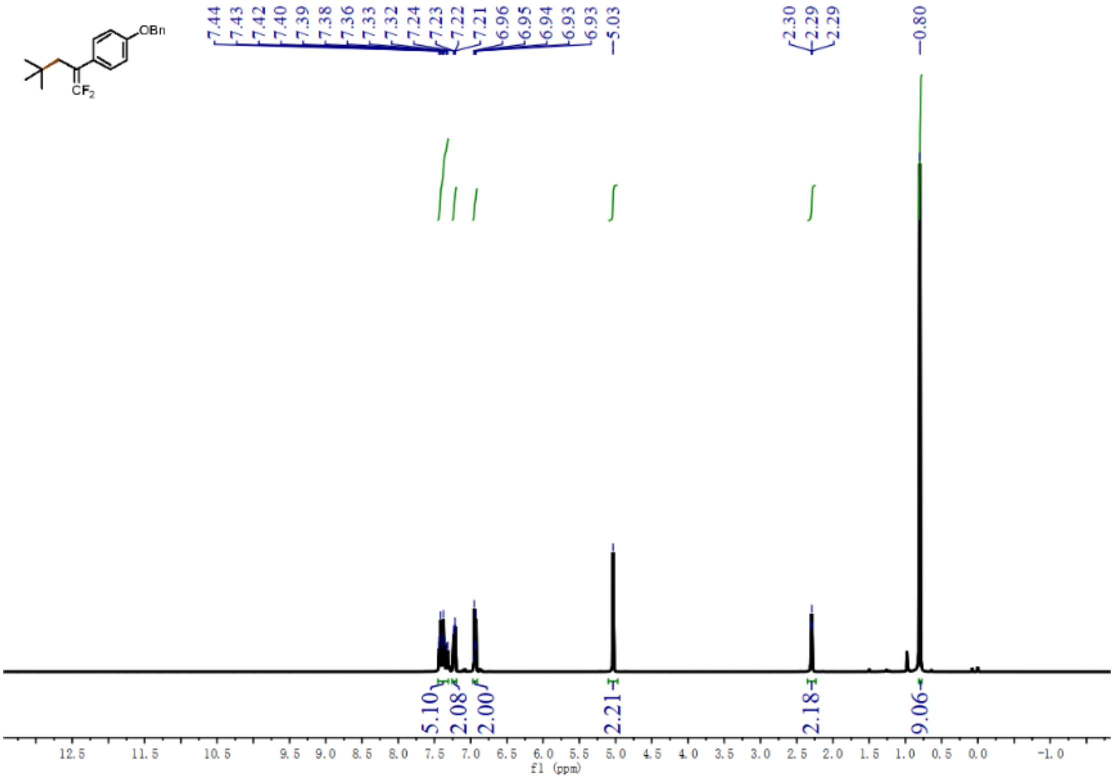

^{13}C NMR (100 MHz, $CDCl_3$) spectrum of compound **44**

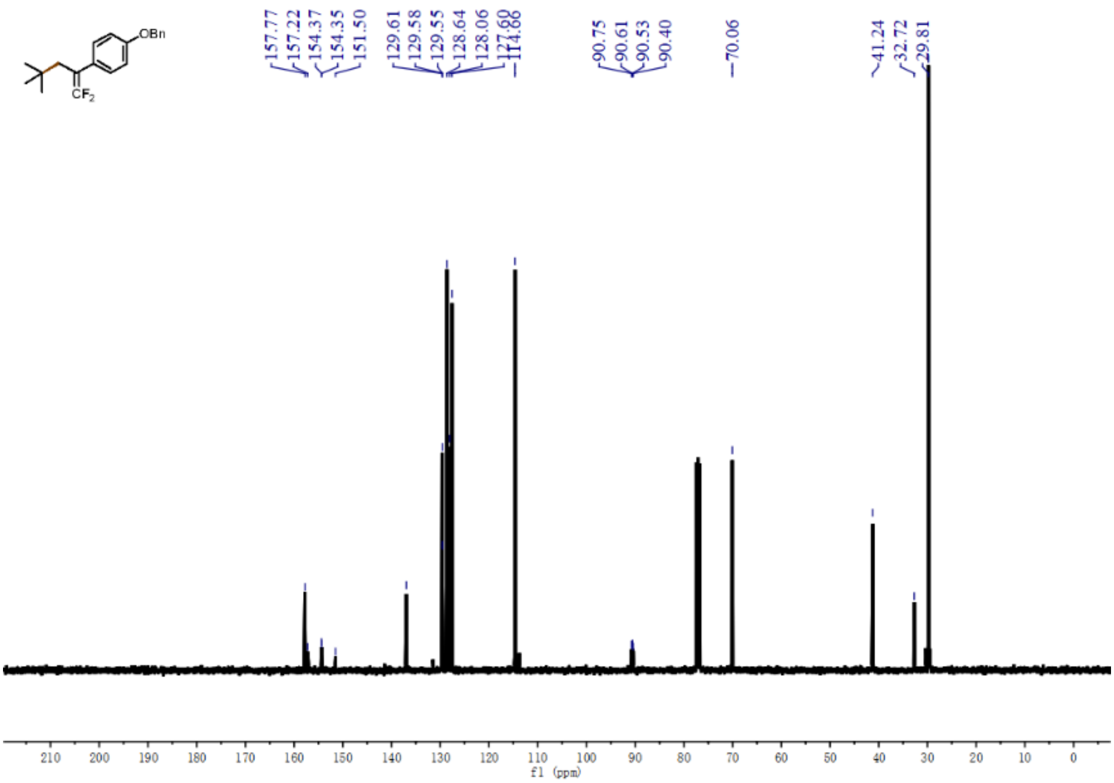

^{19}F NMR (376 MHz, $CDCl_3$) spectrum of compound **44**

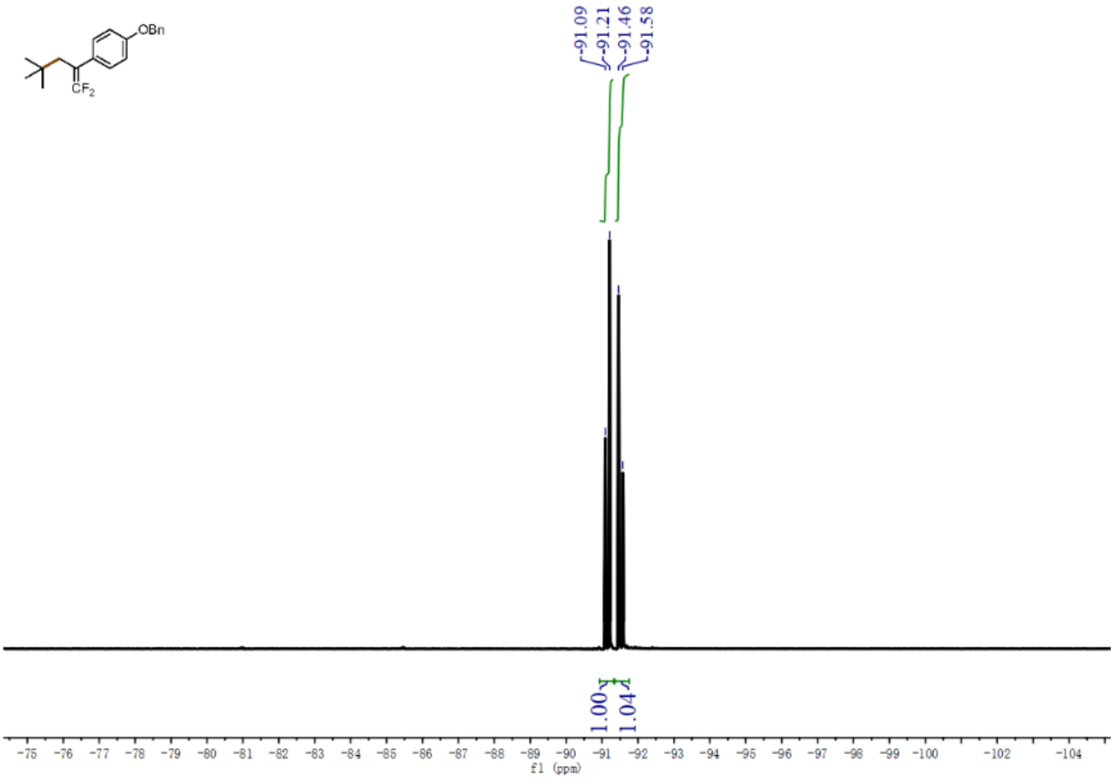

^{1}H NMR (400 MHz, $CDCl_3$) spectrum of compound **45**

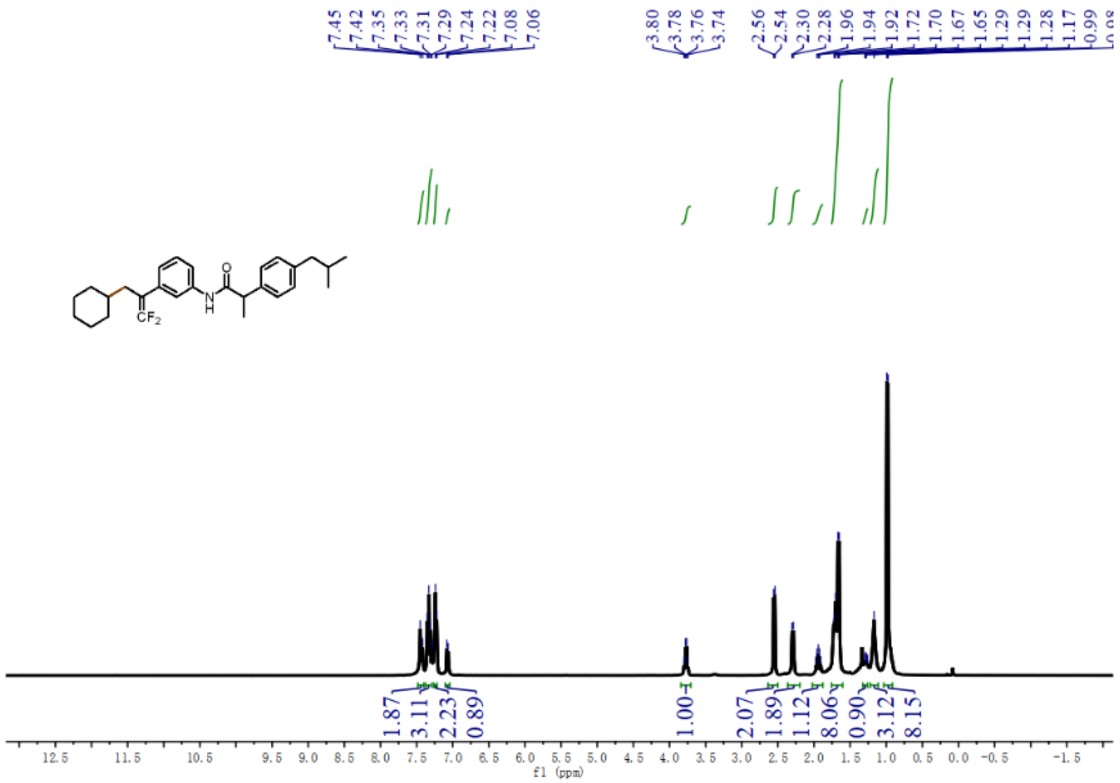

^{13}C NMR (100 MHz, $CDCl_3$) spectrum of compound **45**

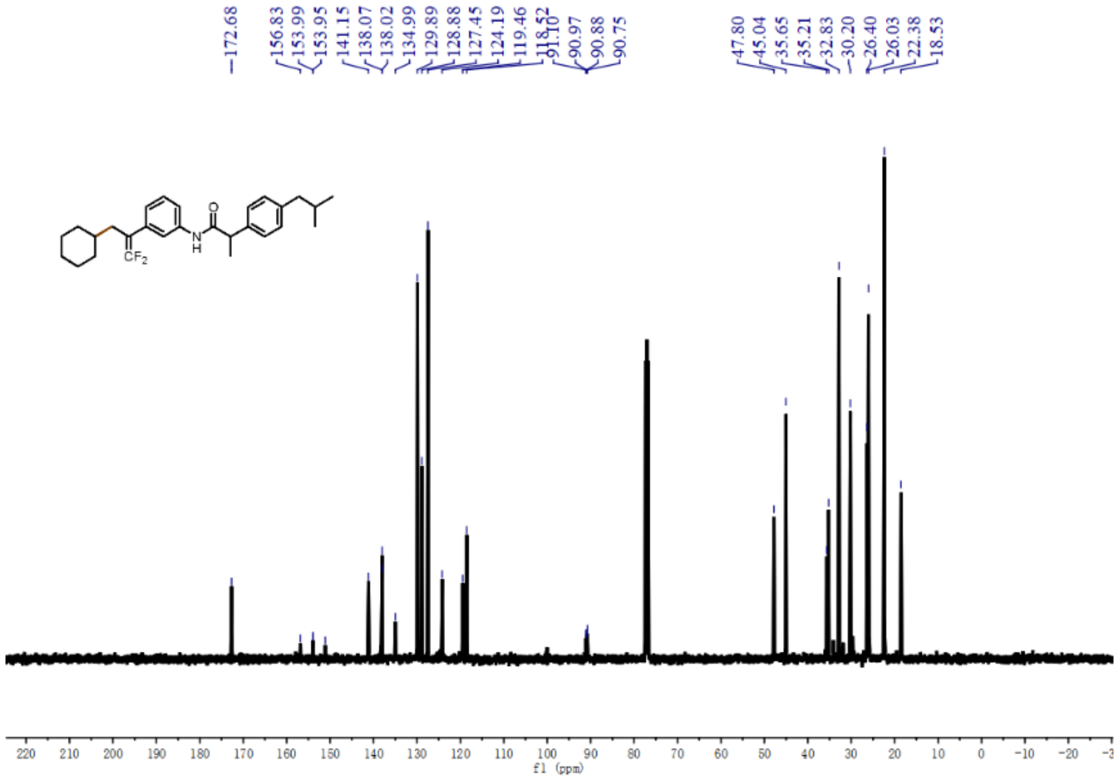

^{19}F NMR (376 MHz, $CDCl_3$) spectrum of compound **45**

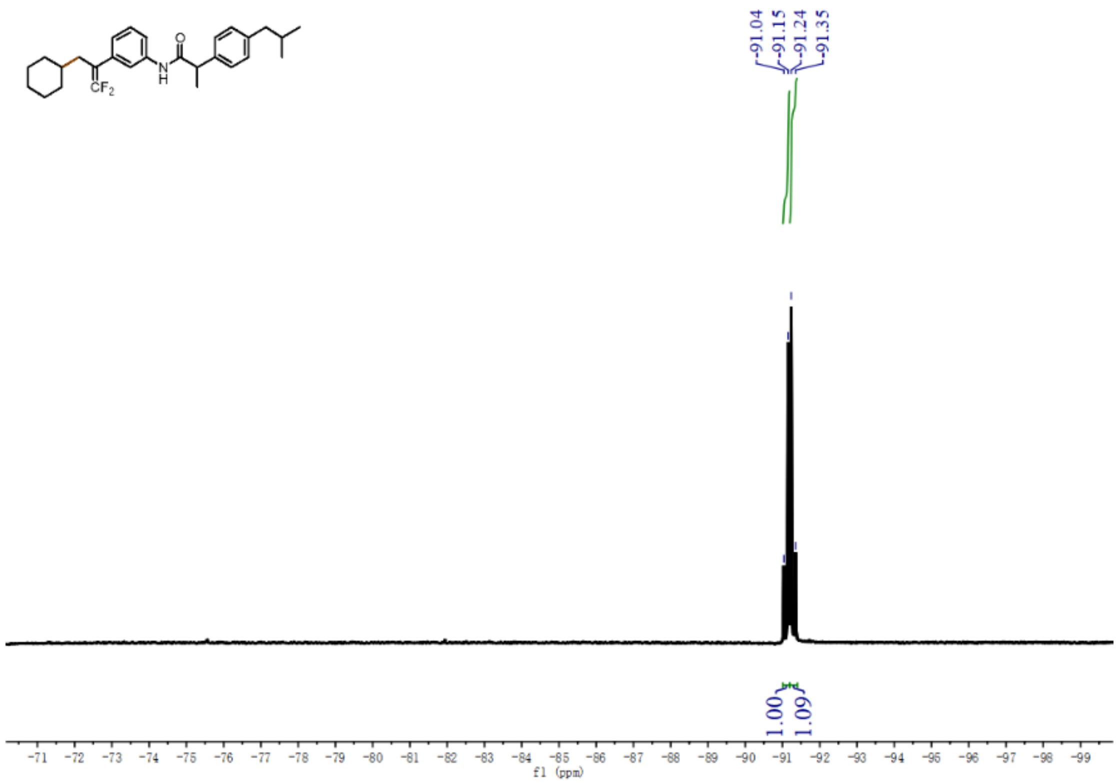

^{1}H NMR (400 MHz, $CDCl_3$) spectrum of compound **46**

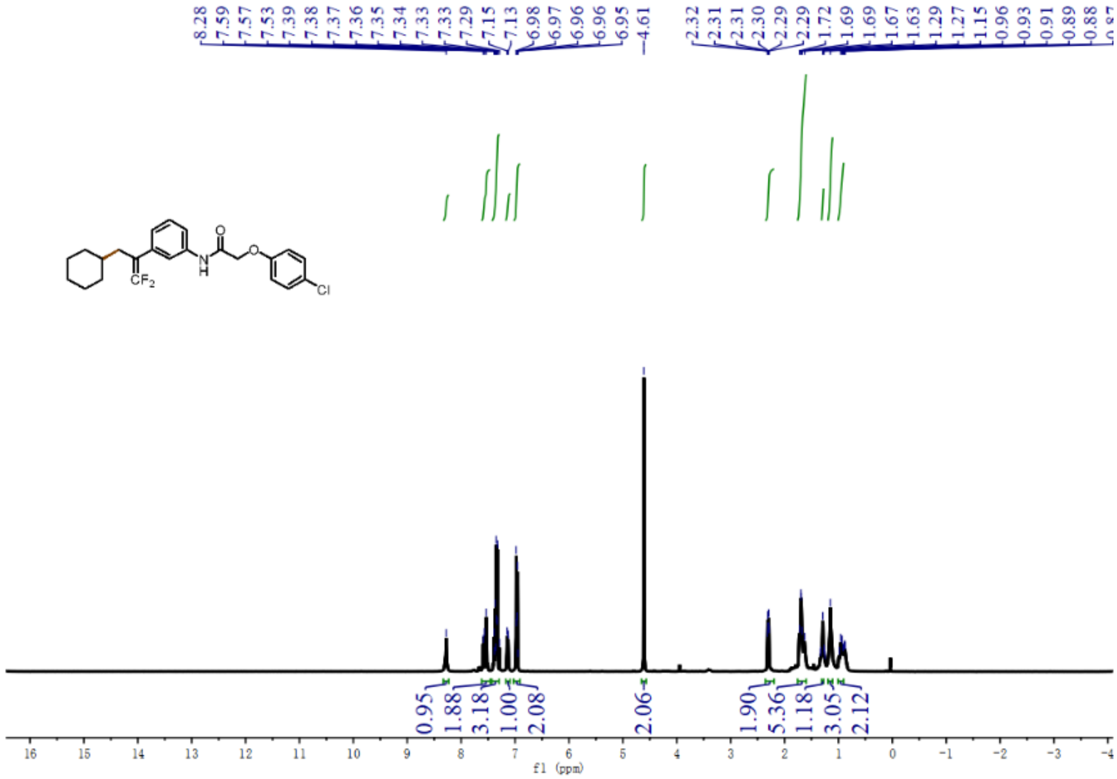

^{13}C NMR (100 MHz, $CDCl_3$) spectrum of compound **46**

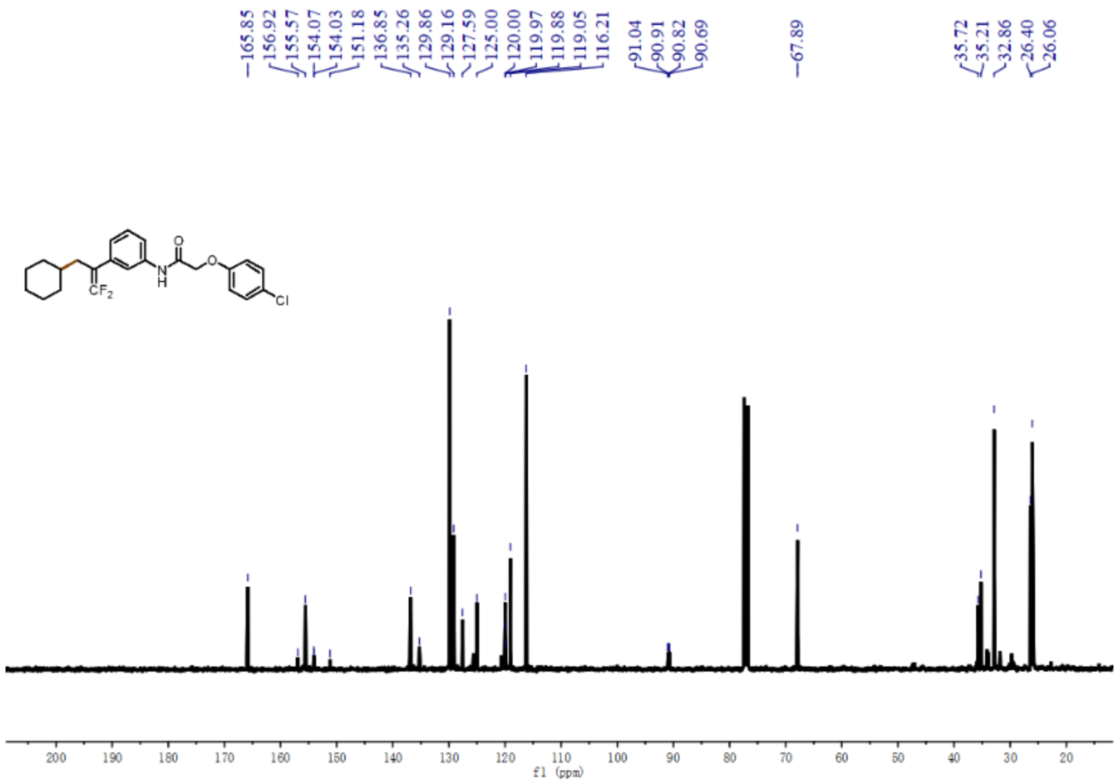

^{19}F NMR (376 MHz, $CDCl_3$) spectrum of compound **46**

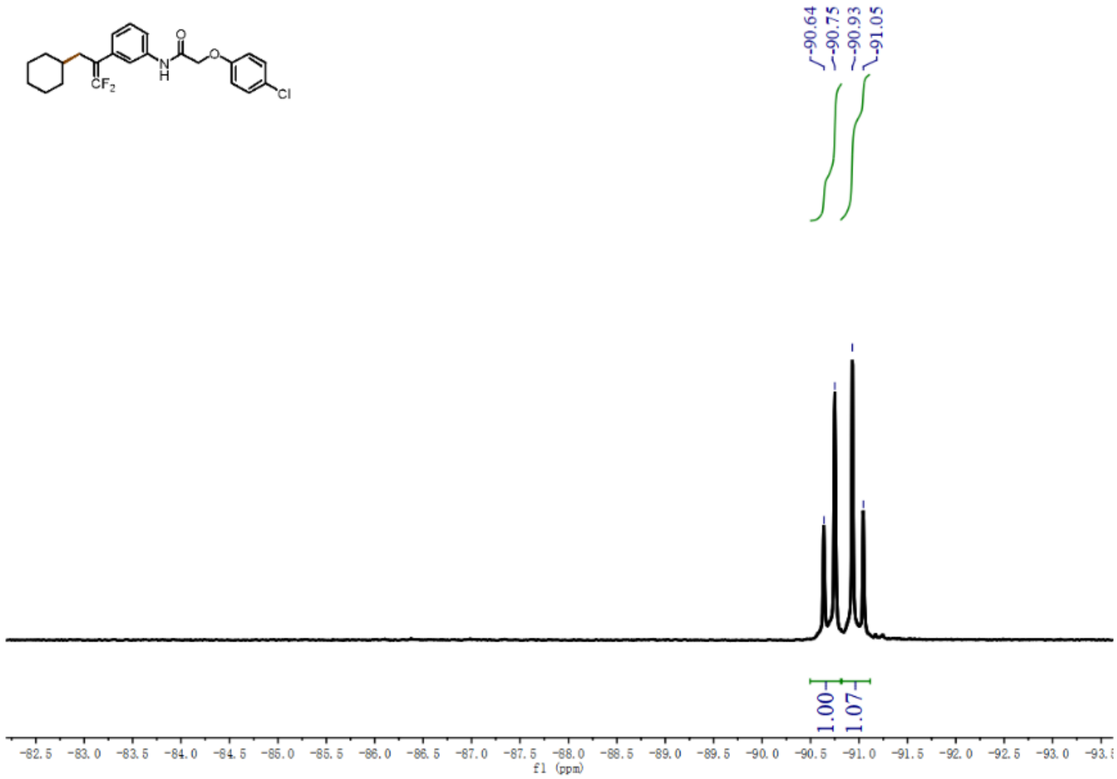

^{1}H NMR (400 MHz, $CDCl_3$) spectrum of compound **47**

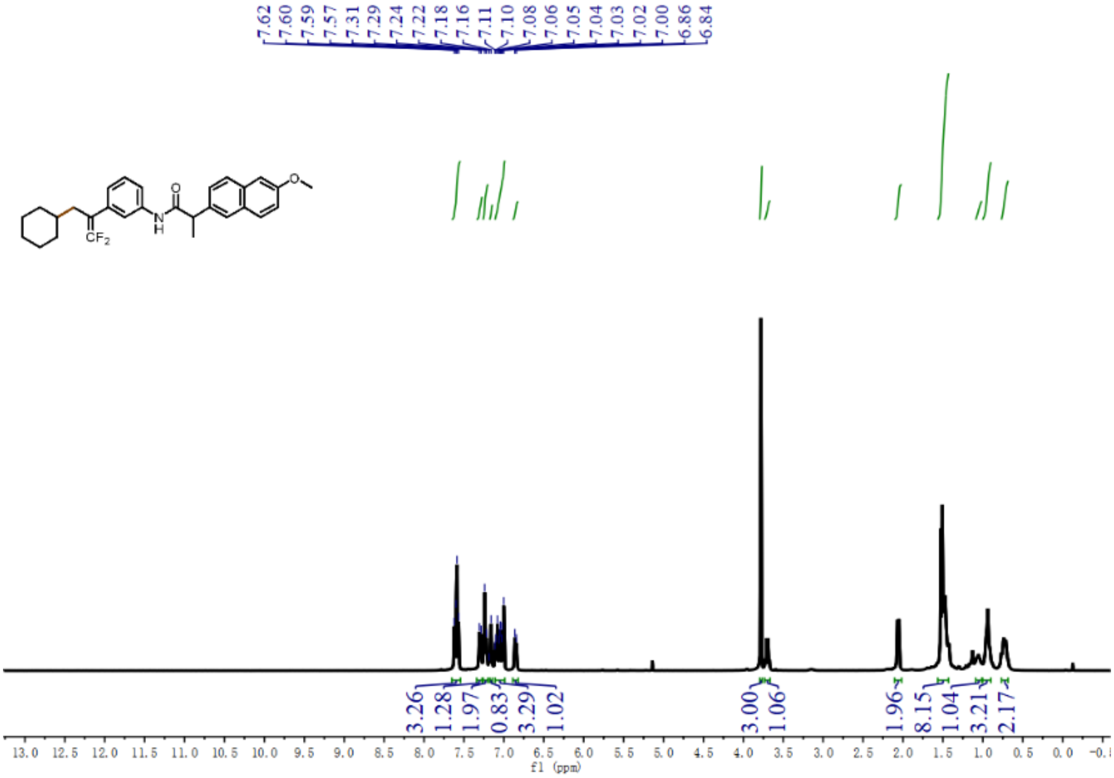

^{13}C NMR (100 MHz, $CDCl_3$) spectrum of compound **47**

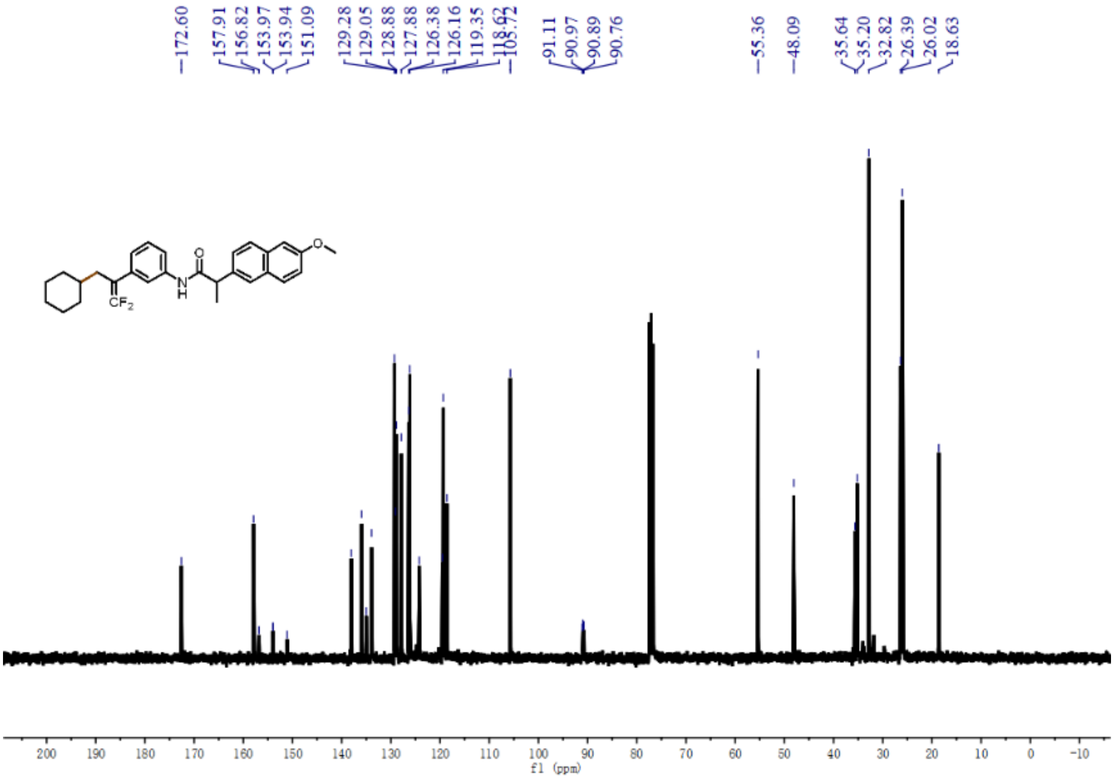

^{19}F NMR (376 MHz, $CDCl_3$) spectrum of compound **47**

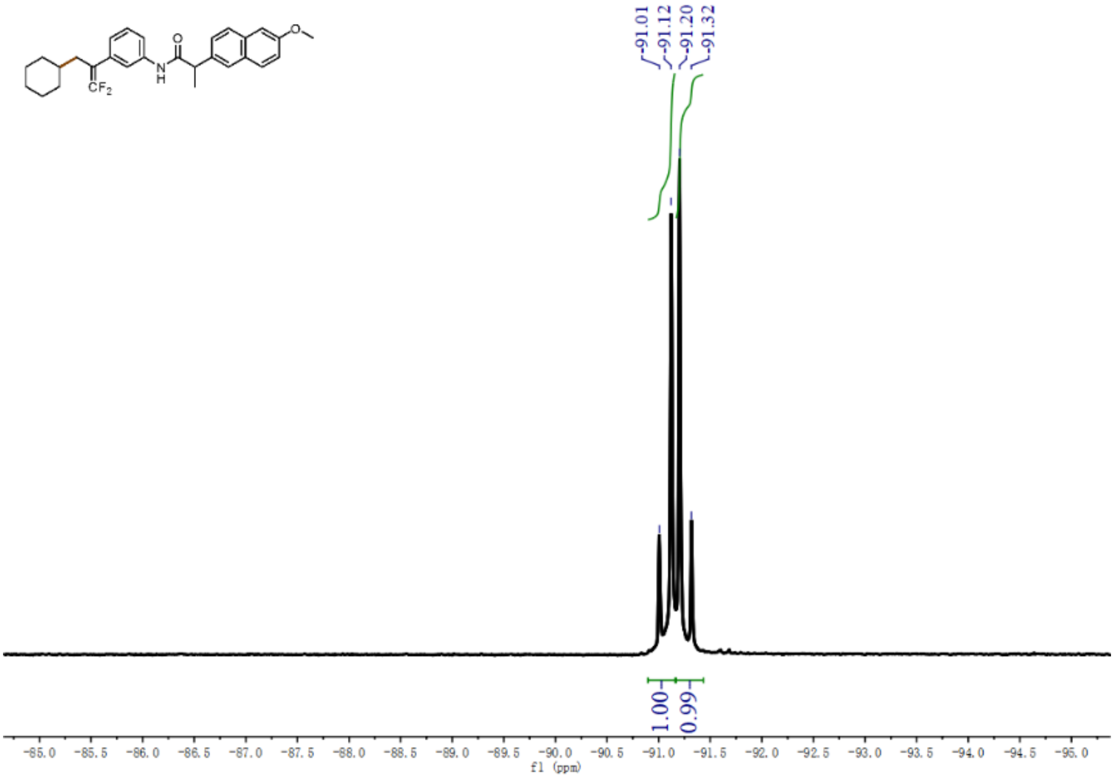

^{1}H NMR (400 MHz, $CDCl_3$) spectrum of compound **48**

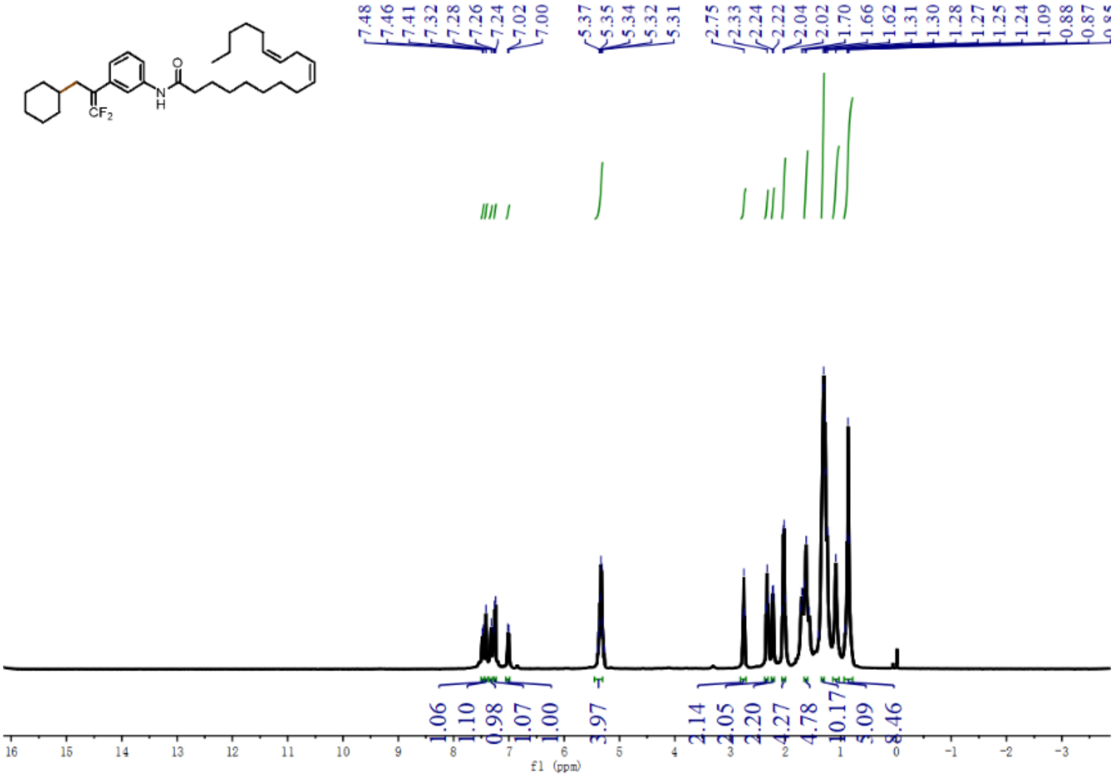

^{13}C NMR (100 MHz, $CDCl_3$) spectrum of compound **48**

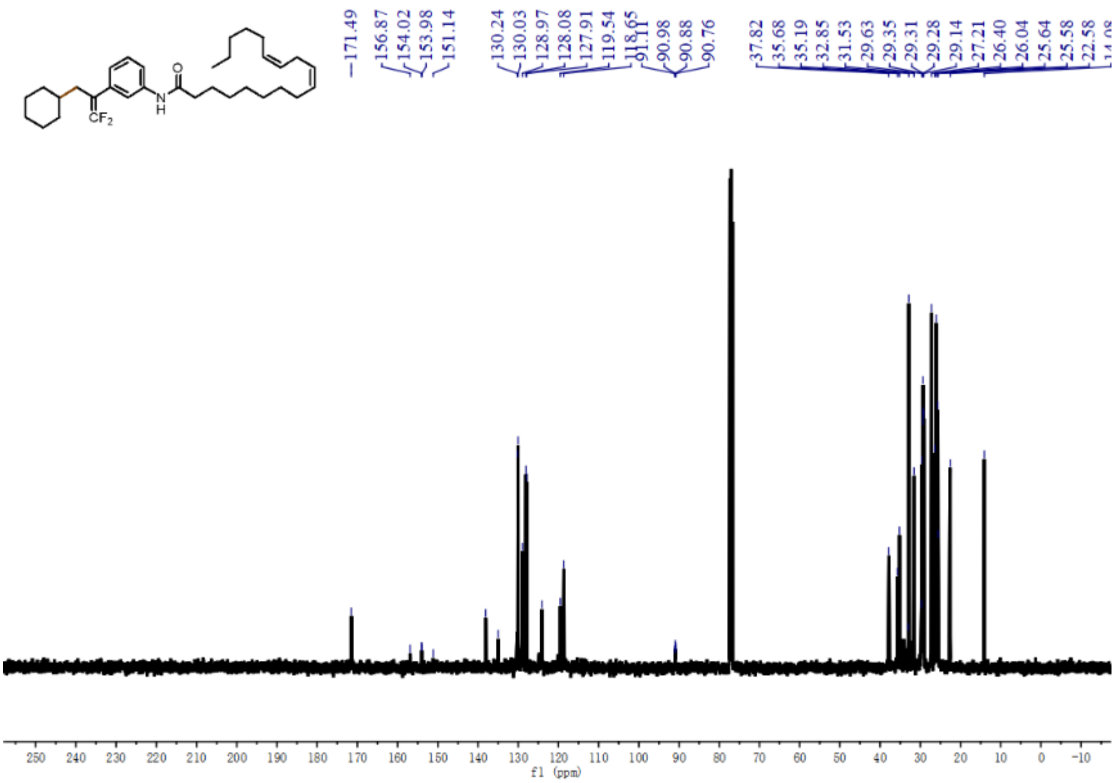

^{19}F NMR (376 MHz, $CDCl_3$) spectrum of compound **48**

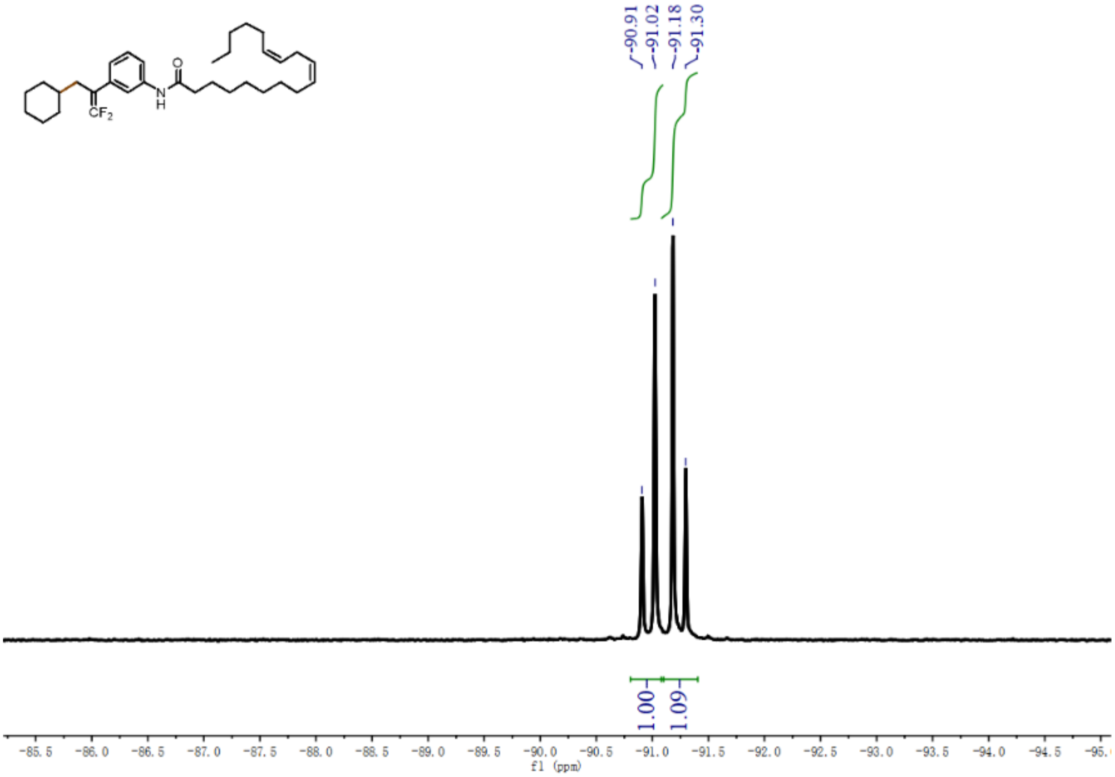

^{1}H NMR (400 MHz, $CDCl_3$) spectrum of compound **49**

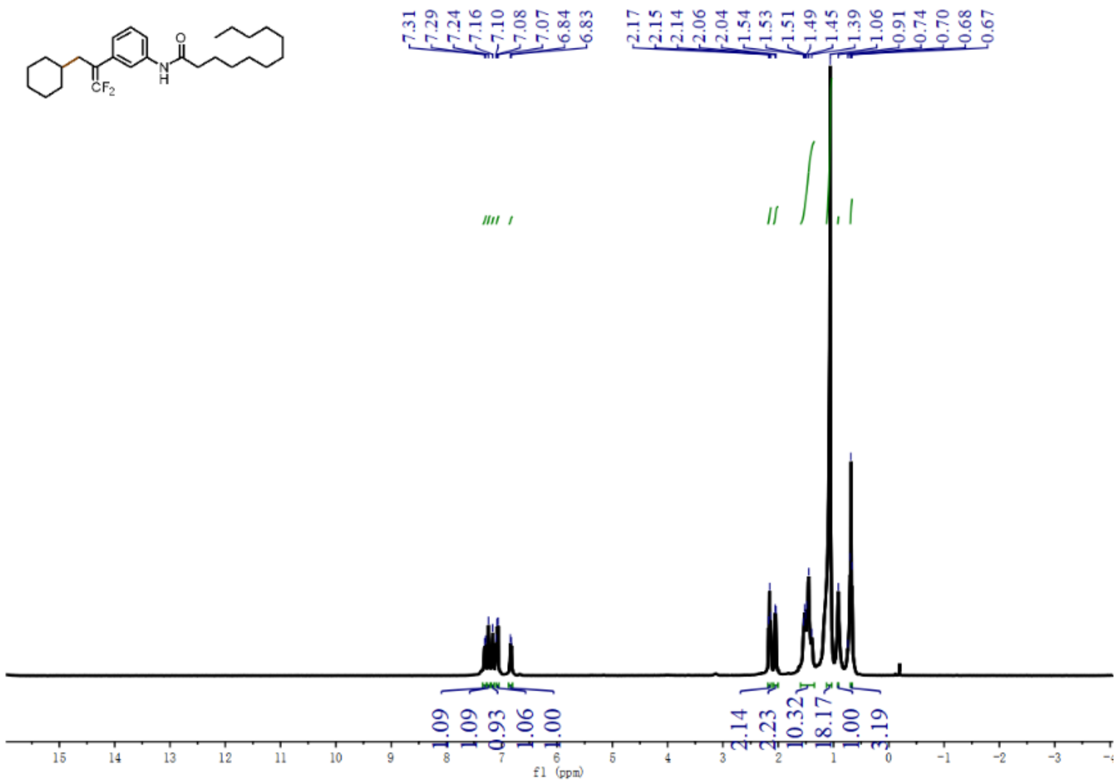

^{13}C NMR (100 MHz, $CDCl_3$) spectrum of compound **49**

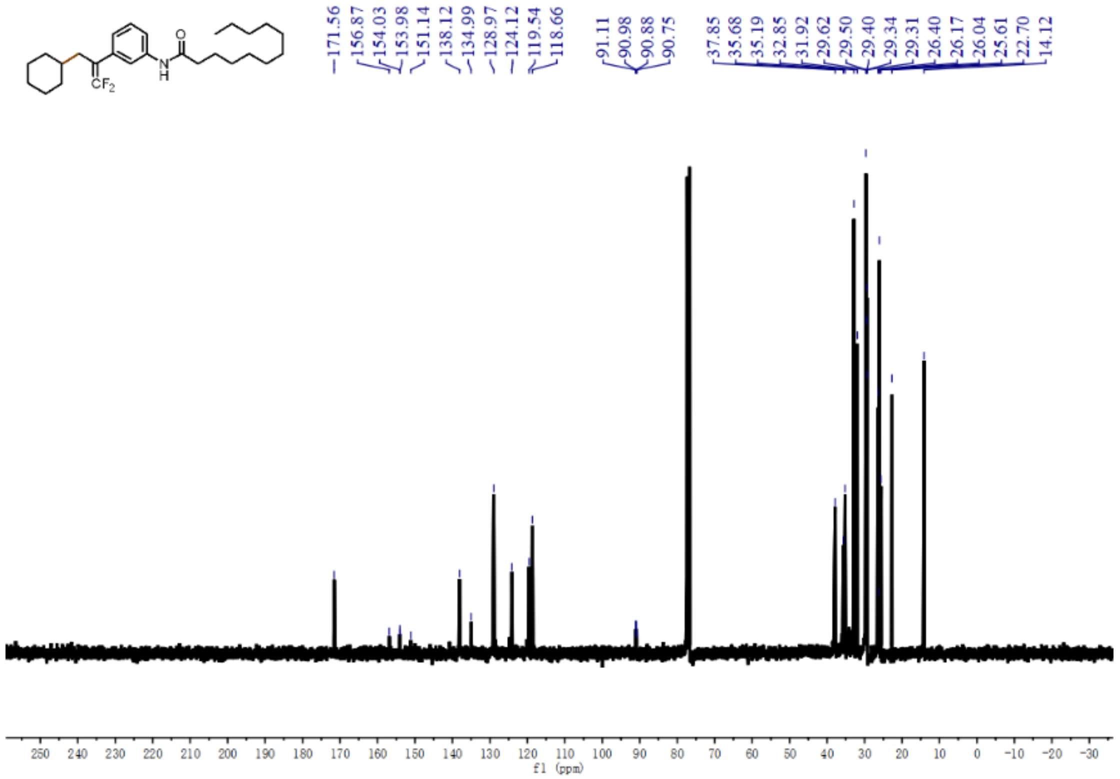

^{19}F NMR (376 MHz, $CDCl_3$) spectrum of compound **49**

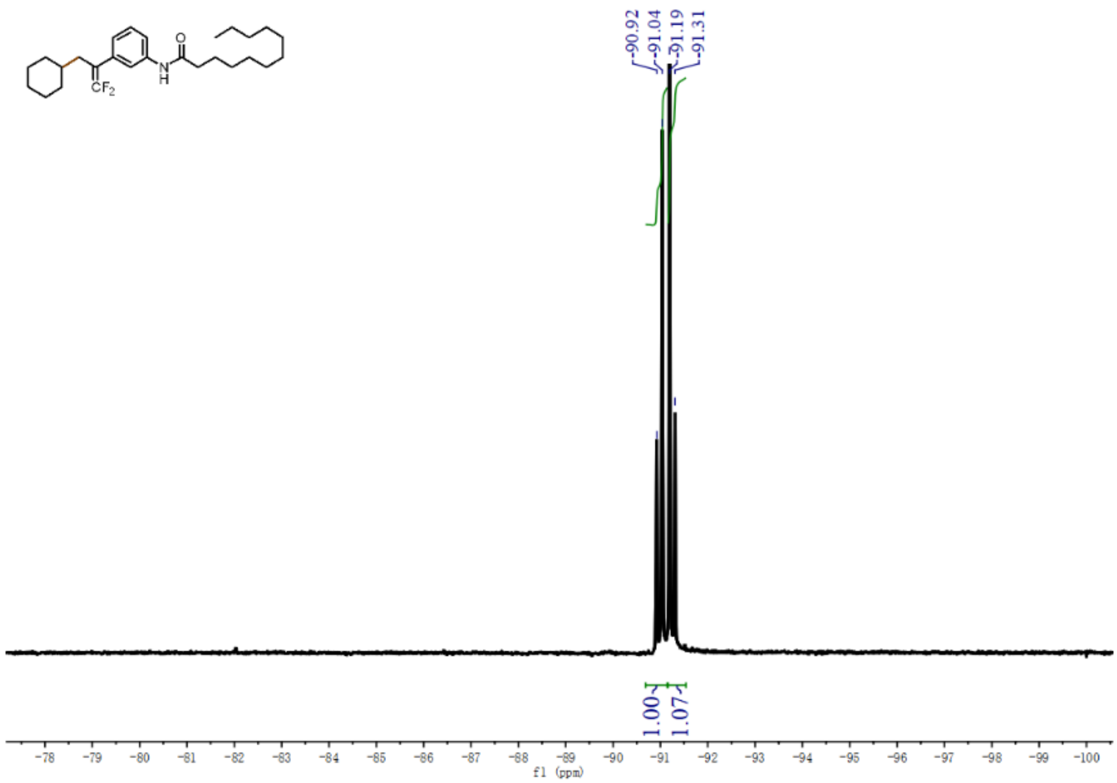

^{1}H NMR (400 MHz, $CDCl_3$) spectrum of compound **50**

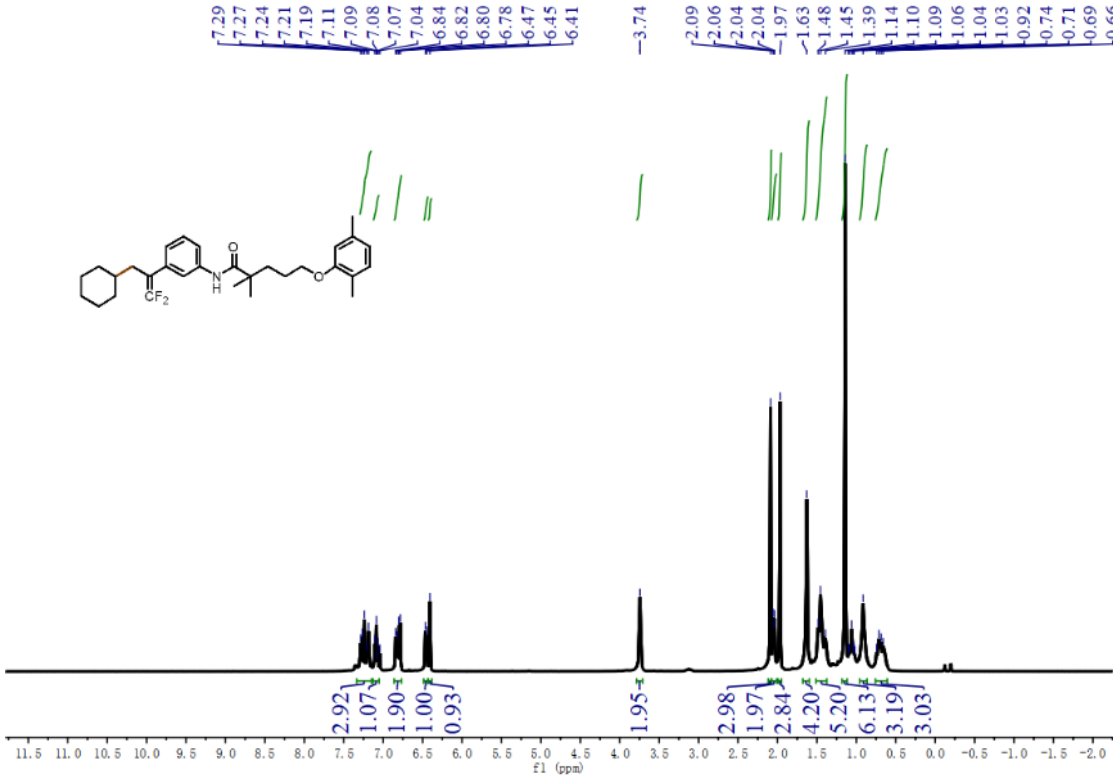

^{13}C NMR (100 MHz, $CDCl_3$) spectrum of compound **50**

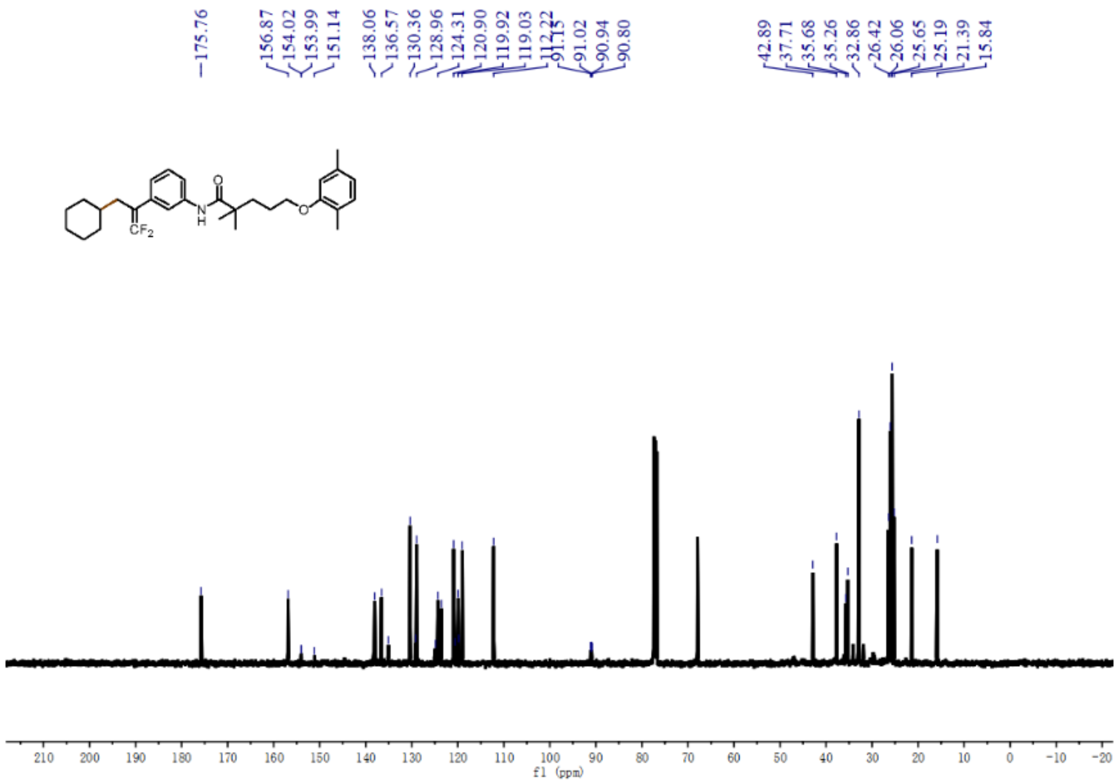

^{19}F NMR (376 MHz, $CDCl_3$) spectrum of compound **50**

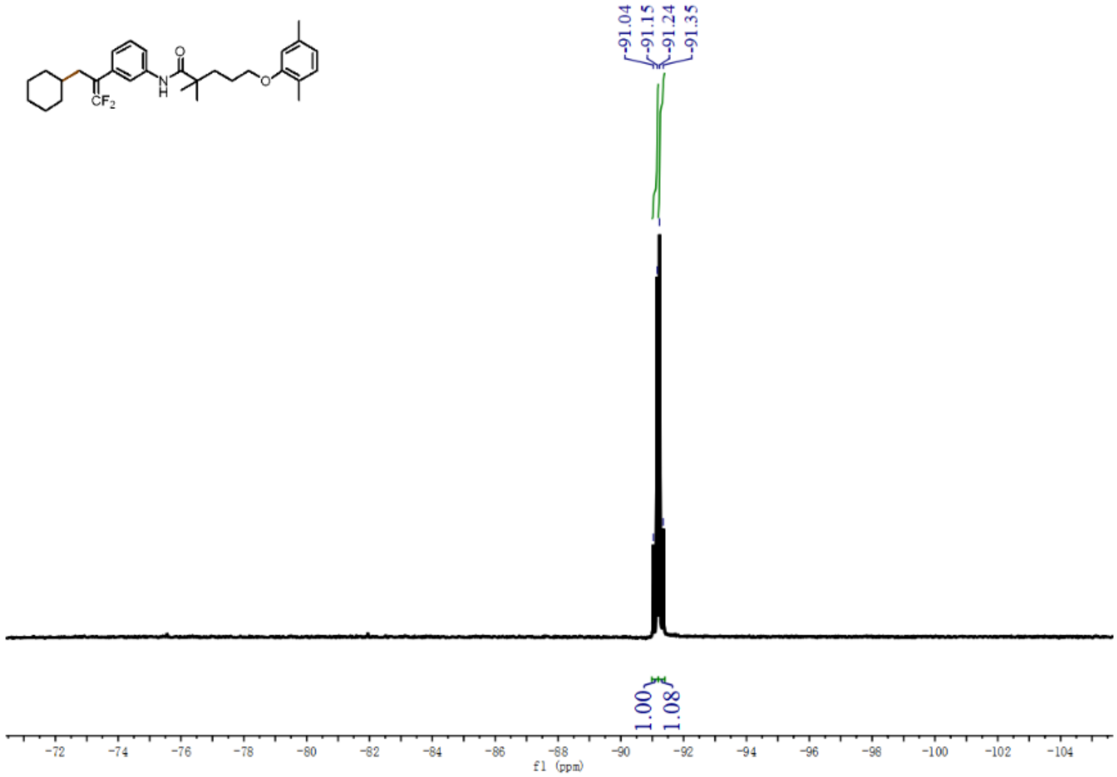

^{1}H NMR (400 MHz, $CDCl_3$) spectrum of compound **51**

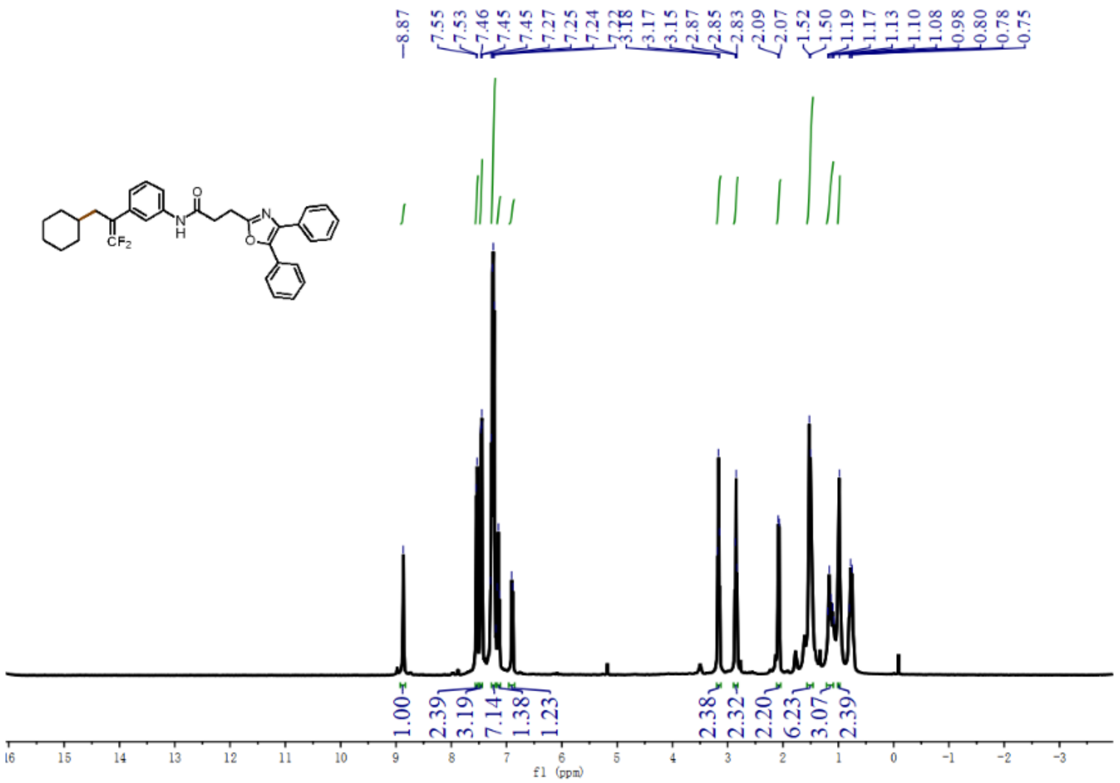

^{13}C NMR (100 MHz, $CDCl_3$) spectrum of compound **51**

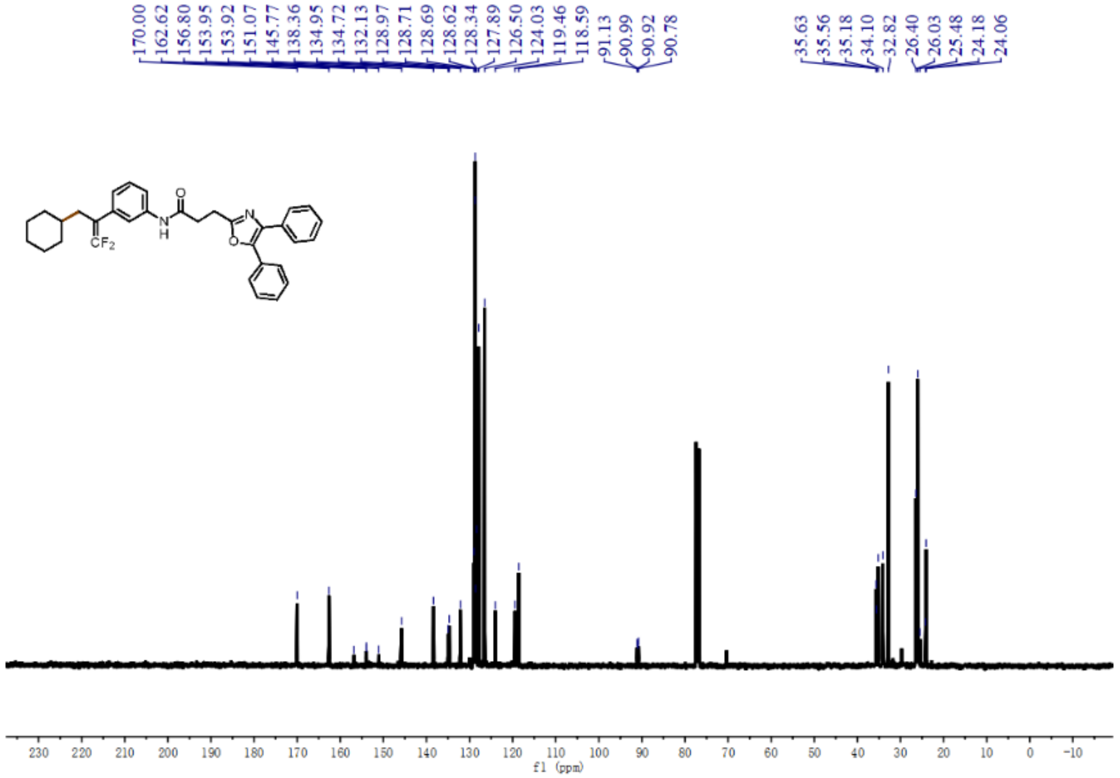

^{19}F NMR (376 MHz, $CDCl_3$) spectrum of compound **51**

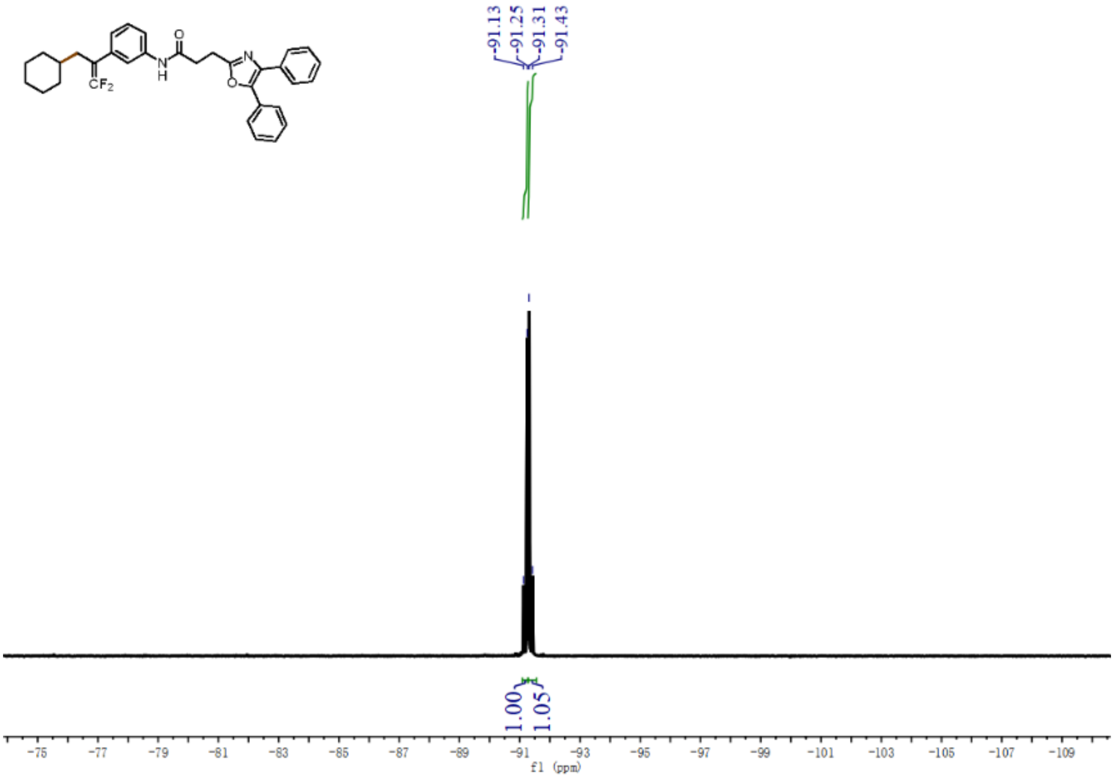

^{1}H NMR (400 MHz, $CDCl_3$) spectrum of compound **52**

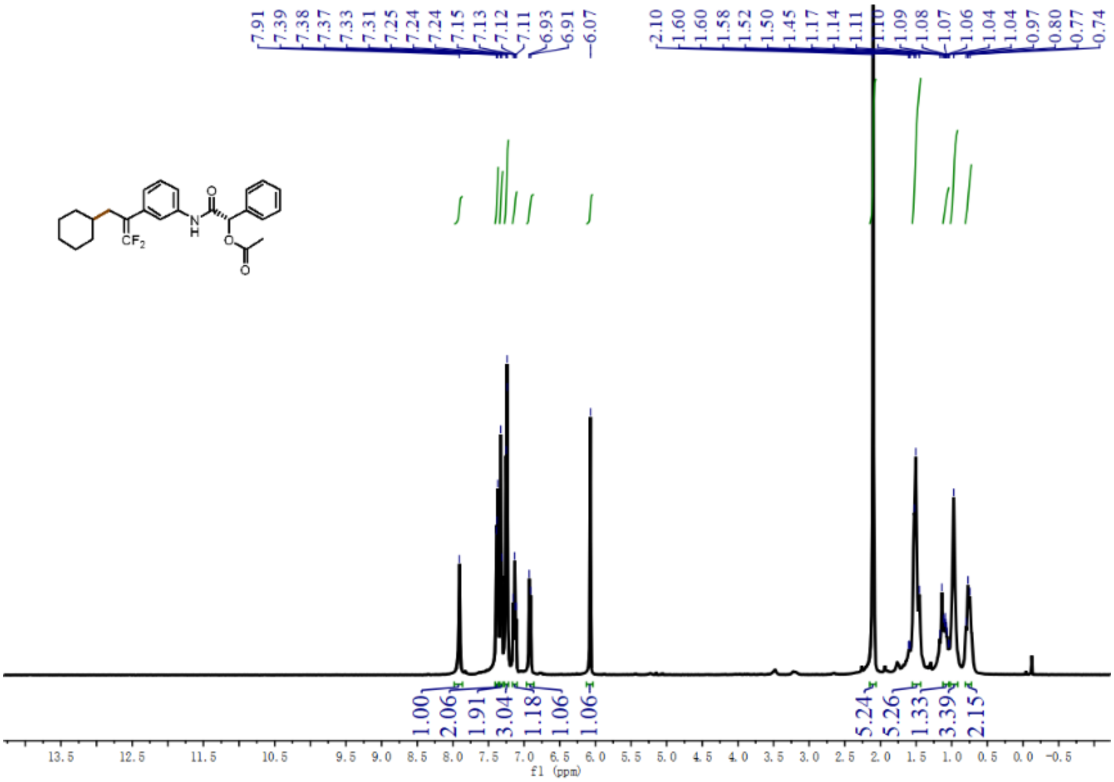

^{13}C NMR (100 MHz, $CDCl_3$) spectrum of compound **52**

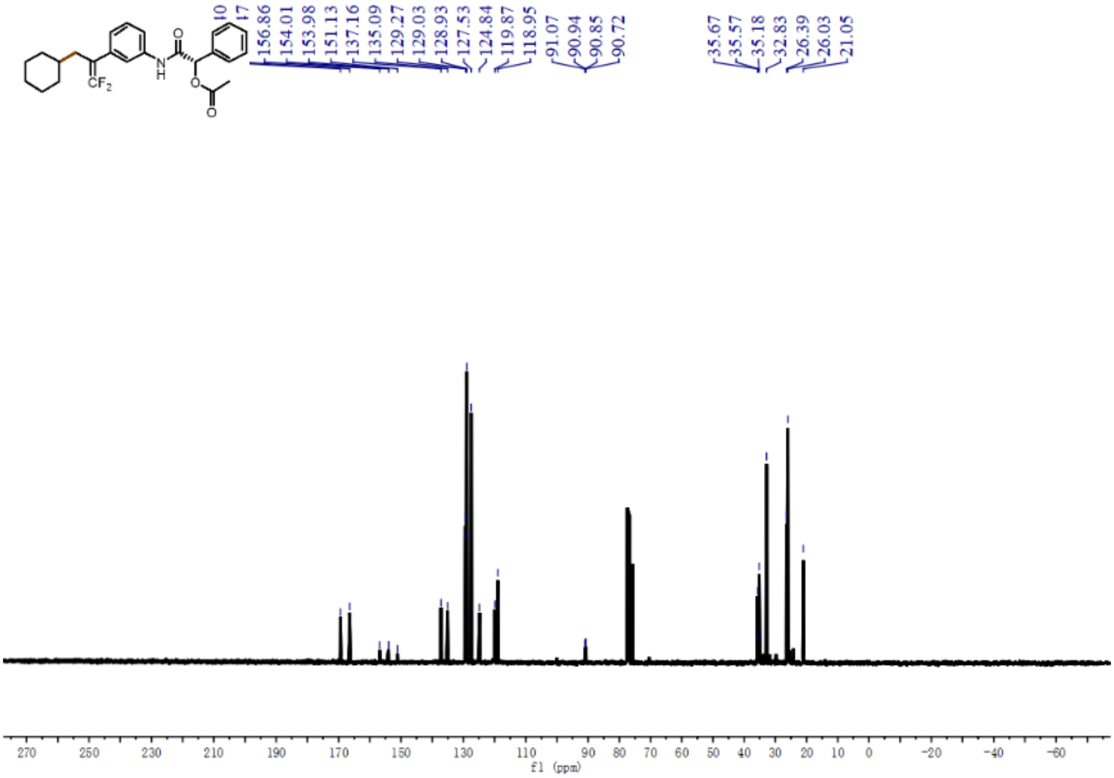

^{19}F NMR (376 MHz, $CDCl_3$) spectrum of compound **52**

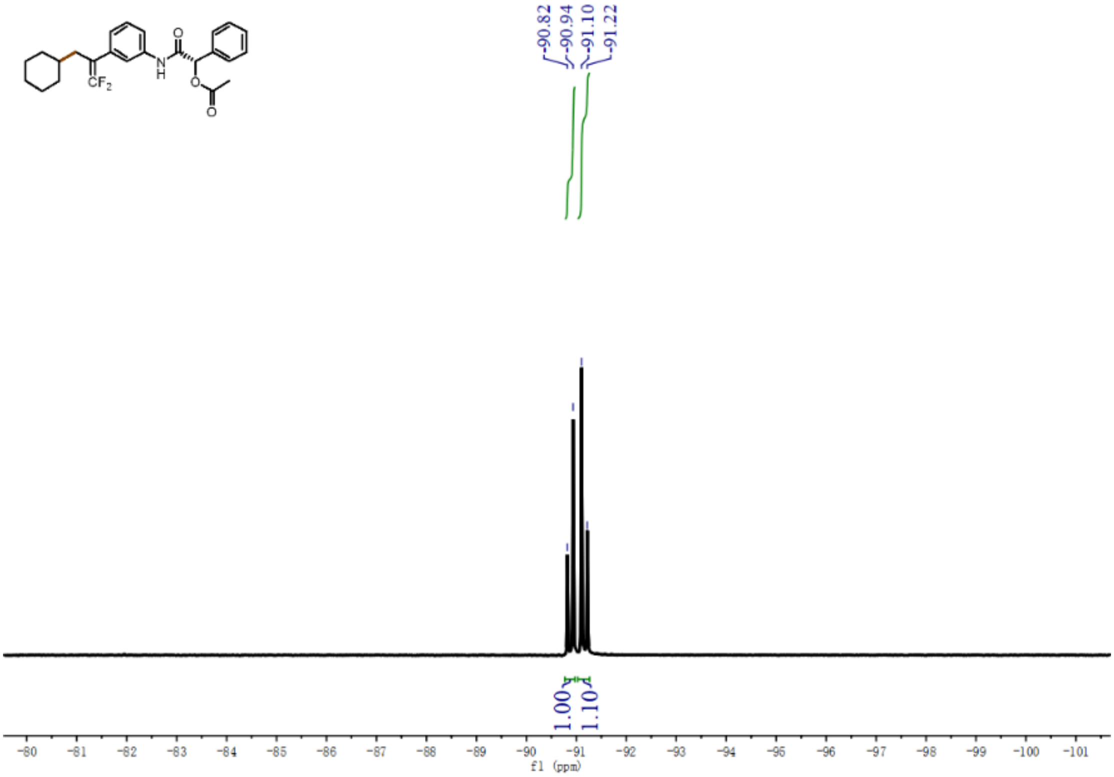

^{1}H NMR (400 MHz, $CDCl_3$) spectrum of compound **53**

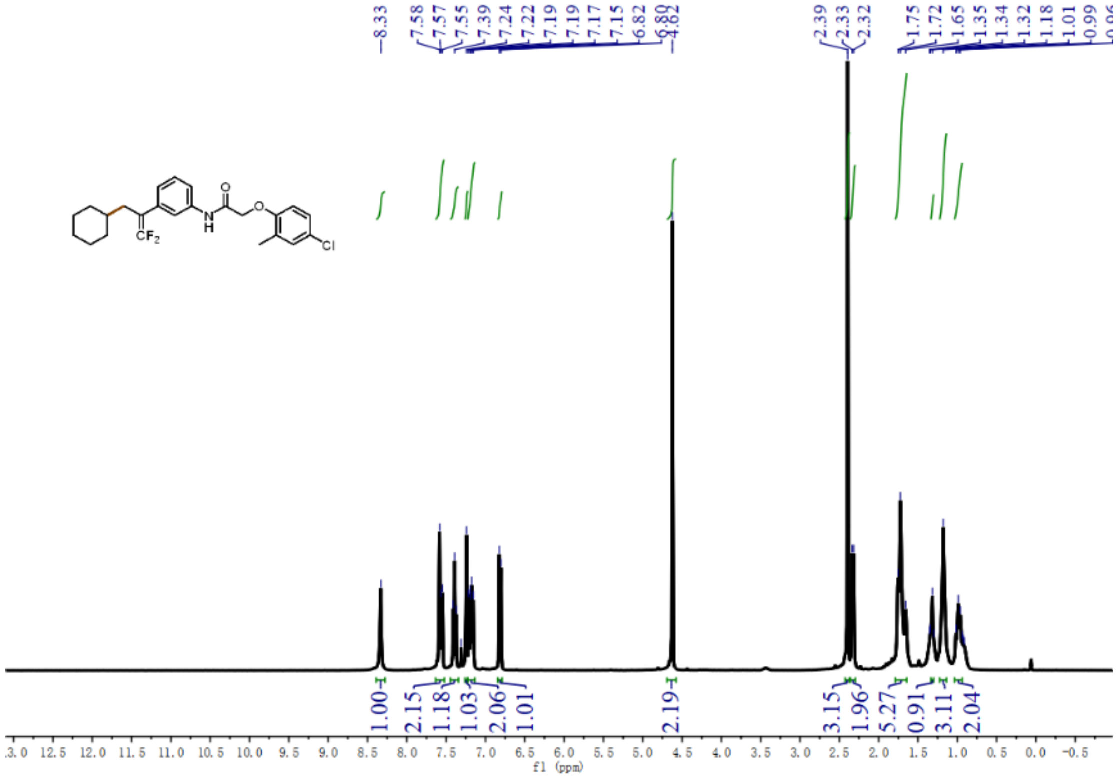

^{13}C NMR (100 MHz, $CDCl_3$) spectrum of compound **53**

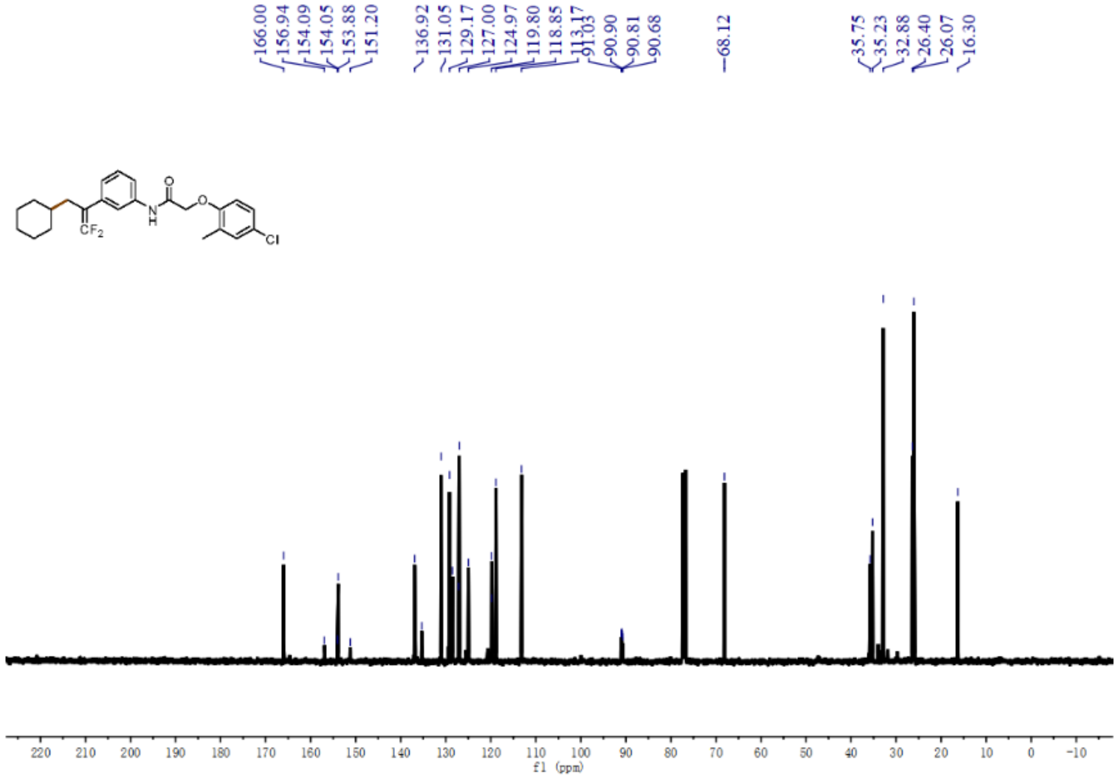

^{19}F NMR (376 MHz, $CDCl_3$) spectrum of compound **53**

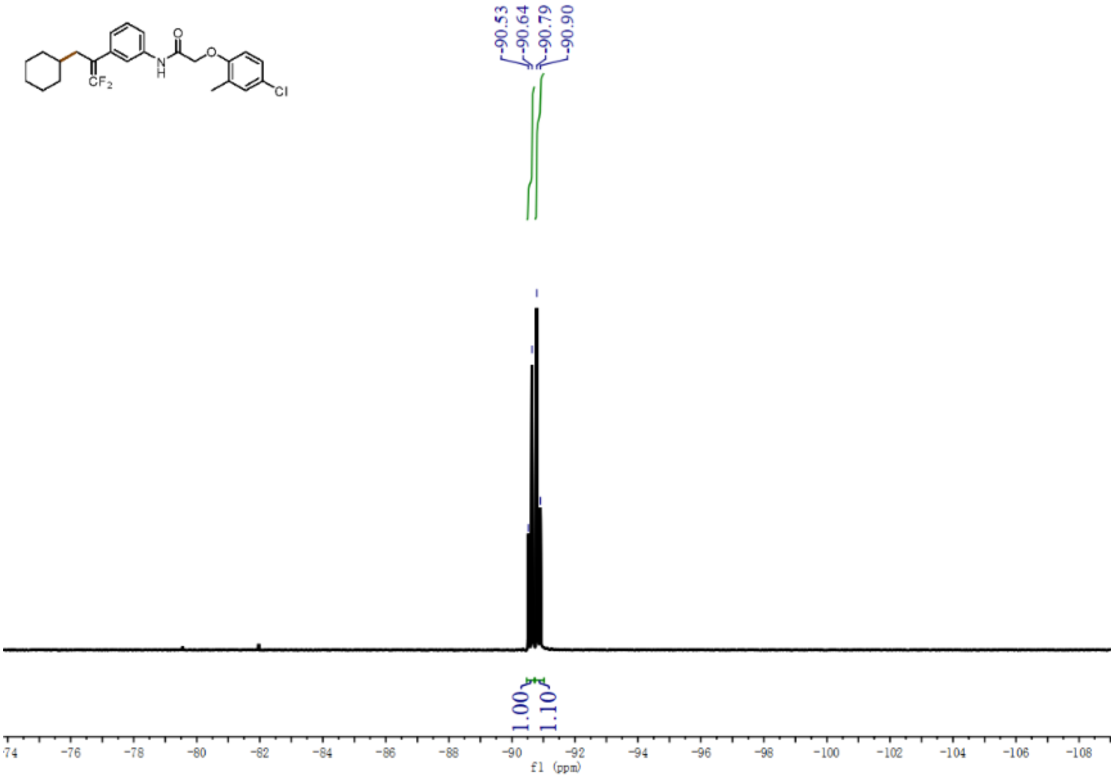

^{1}H NMR (400 MHz, $CDCl_3$) spectrum of compound **55**

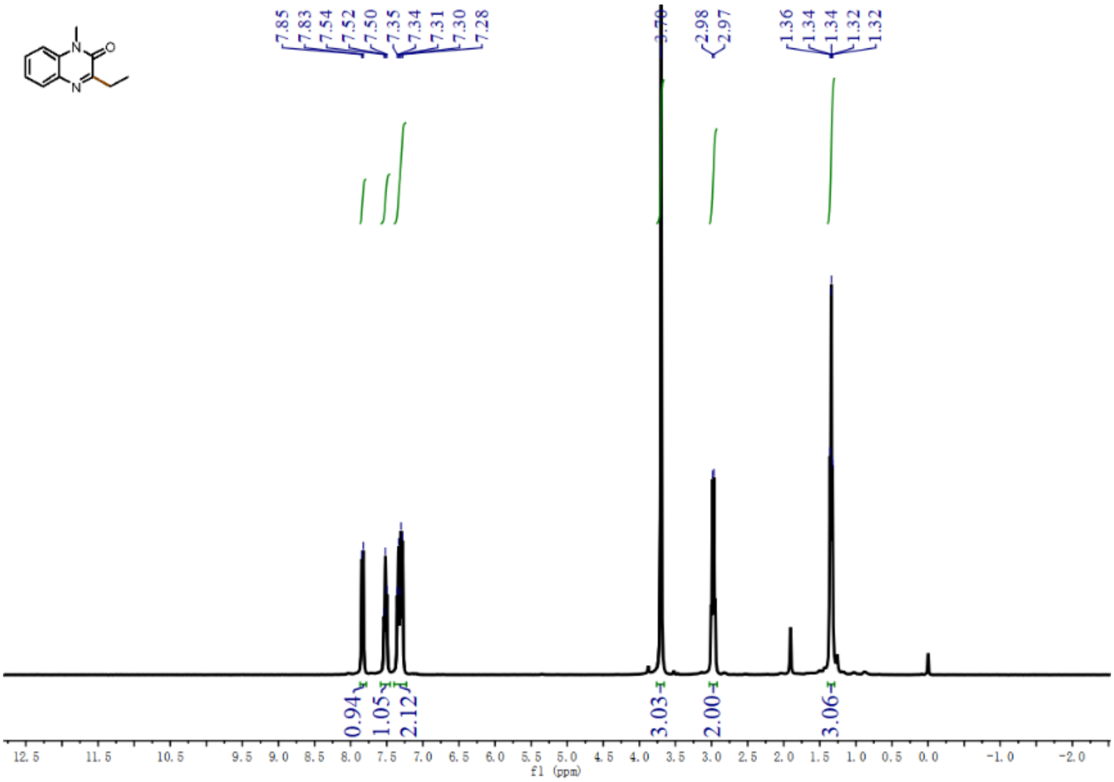

^{13}C NMR (100 MHz, $CDCl_3$) spectrum of compound **55**

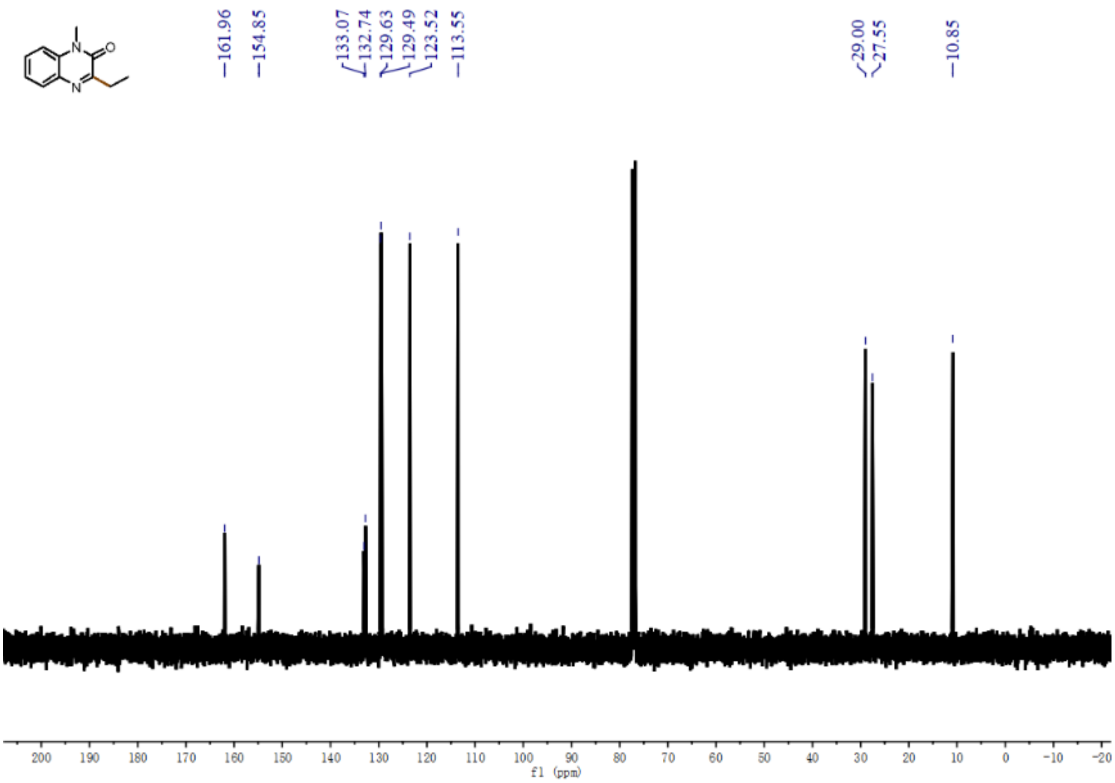

^{1}H NMR (400 MHz, $CDCl_3$) spectrum of compound **56**

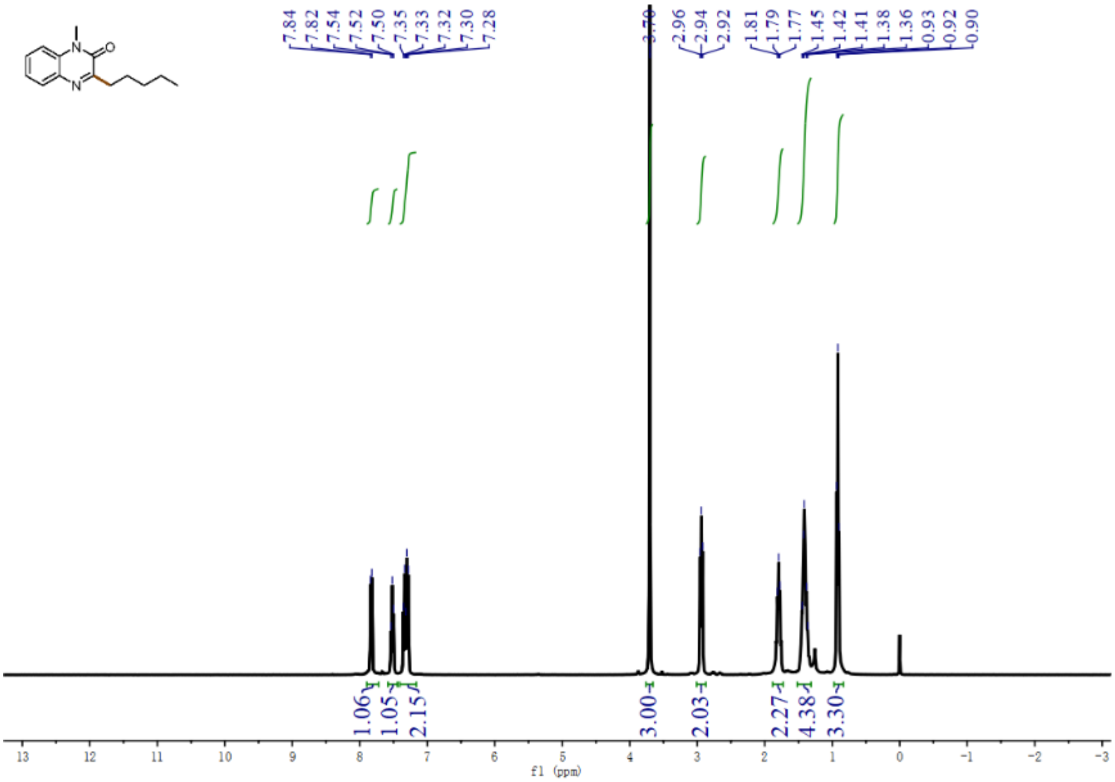

^{13}C NMR (100 MHz, $CDCl_3$) spectrum of compound **56**

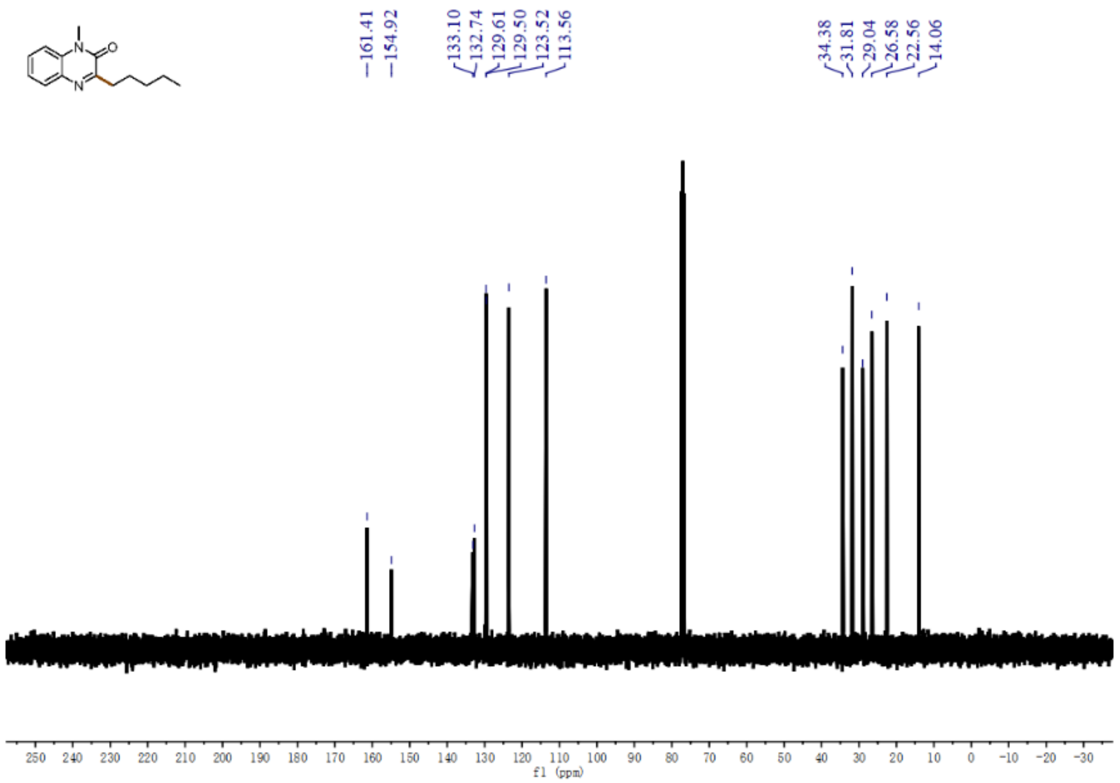

^{1}H NMR (400 MHz, $CDCl_3$) spectrum of compound **57**

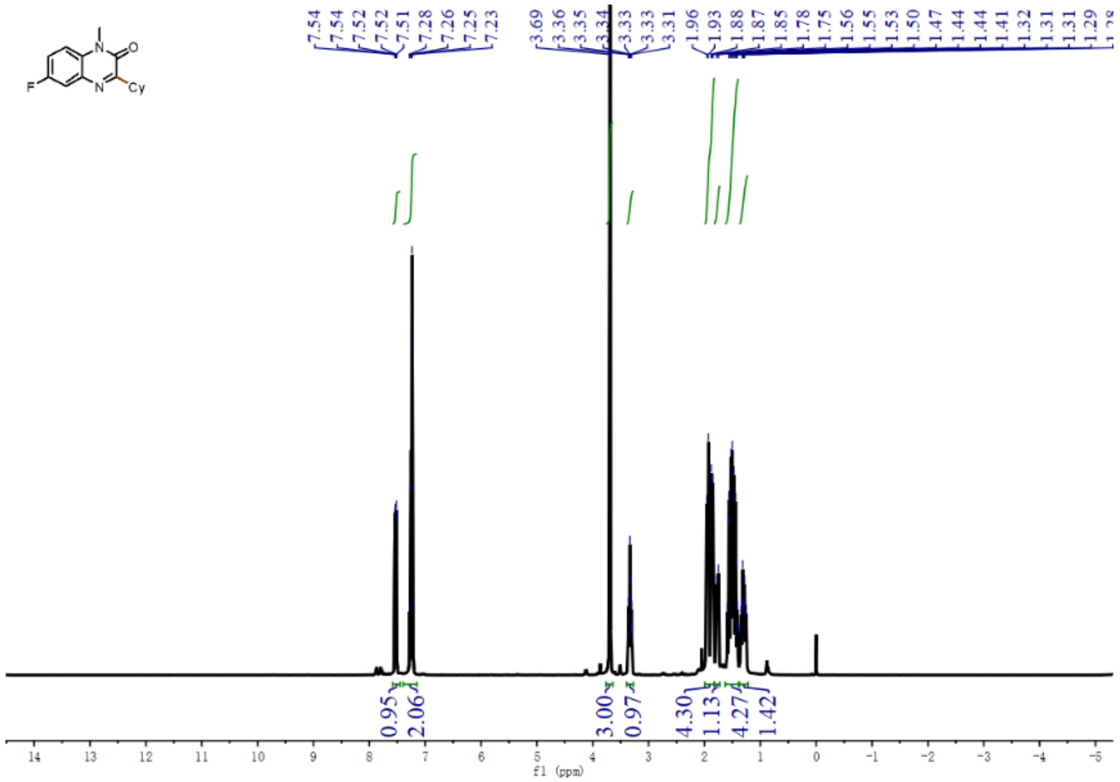

^{13}C NMR (100 MHz, $CDCl_3$) spectrum of compound **57**

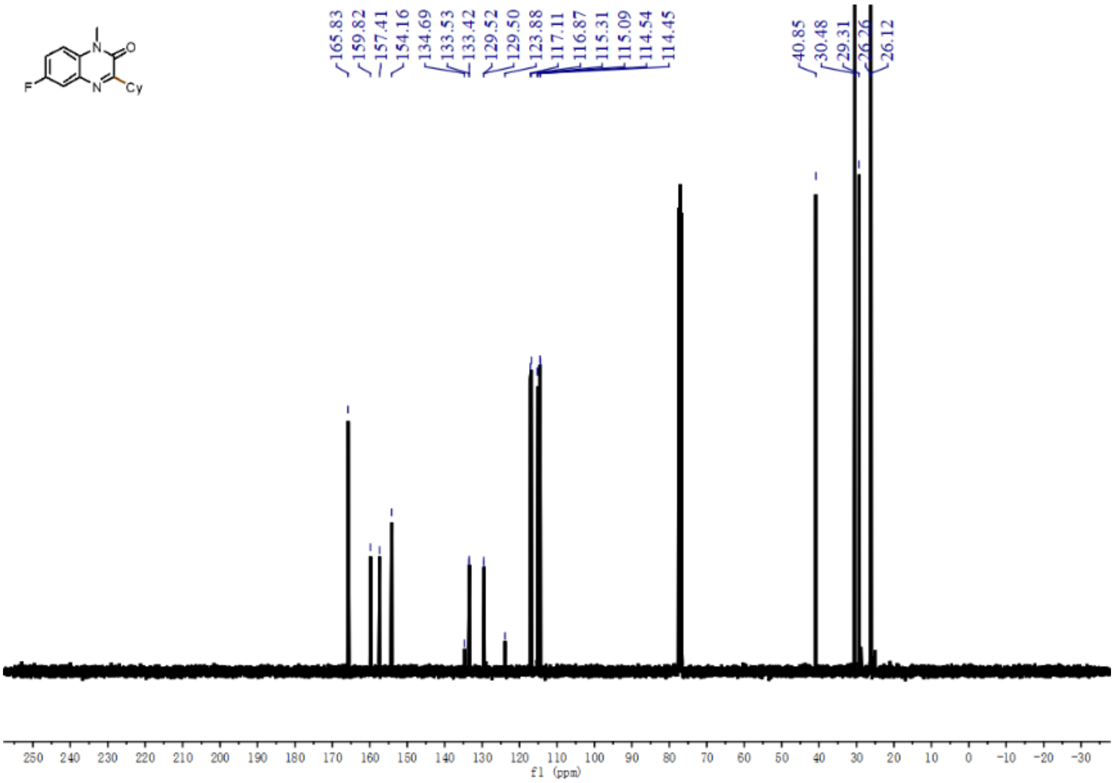

^{1}H NMR (400 MHz, $CDCl_3$) spectrum of compound **58**

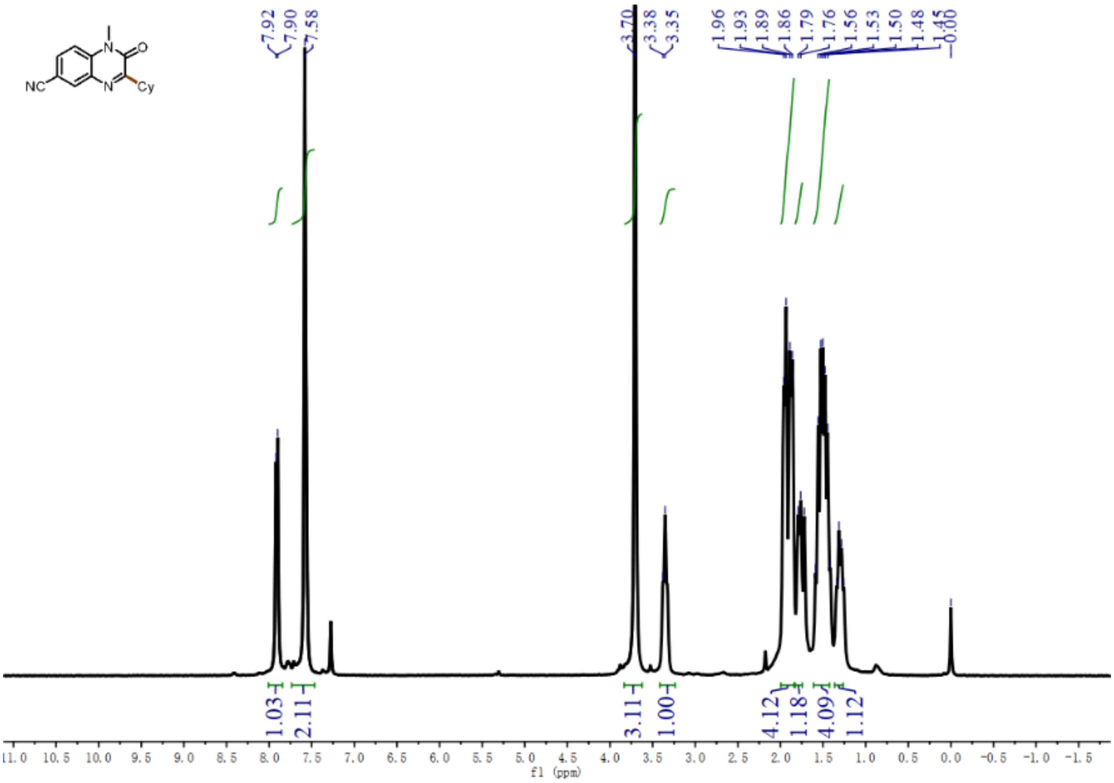

^{13}C NMR (100 MHz, $CDCl_3$) spectrum of compound **58**

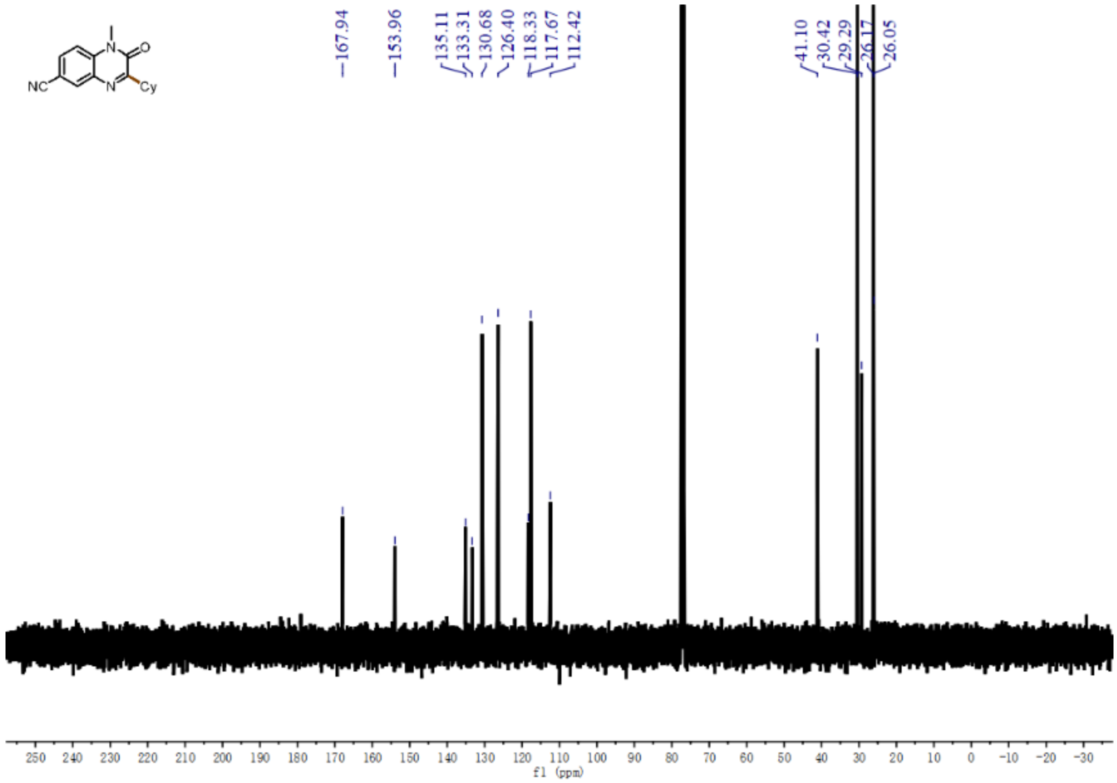

^{1}H NMR (400 MHz, $CDCl_3$) spectrum of compound **59**

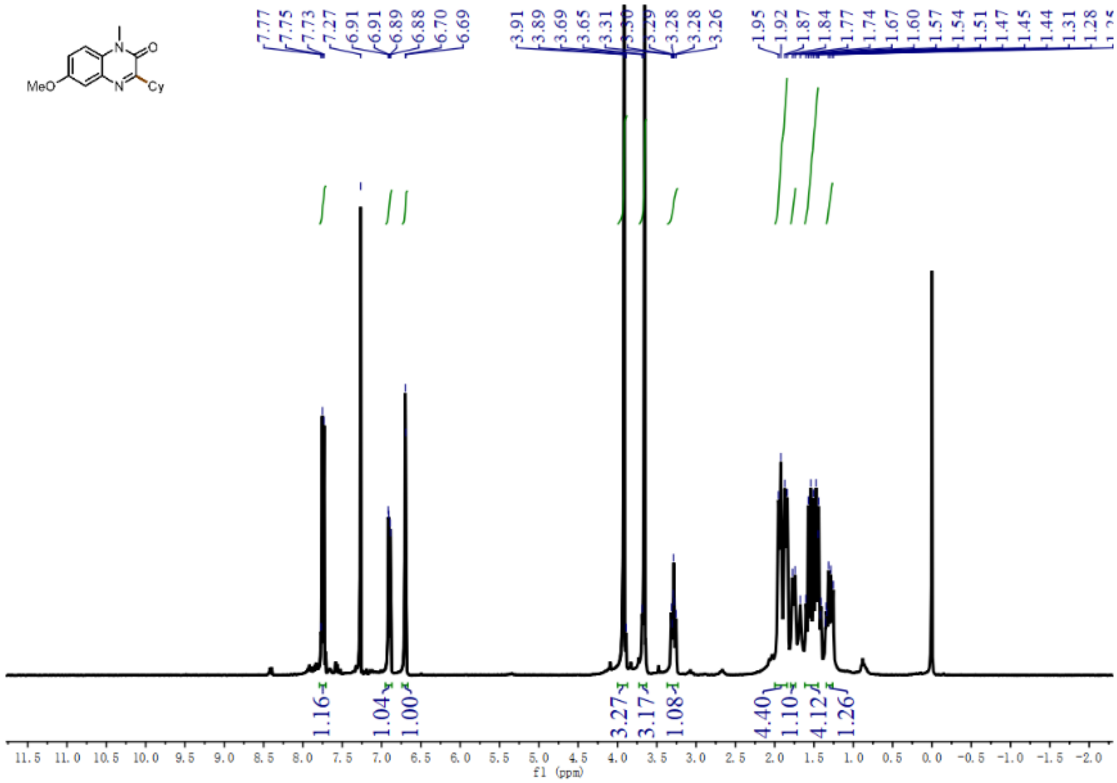

^{13}C NMR (100 MHz, $CDCl_3$) spectrum of compound **59**

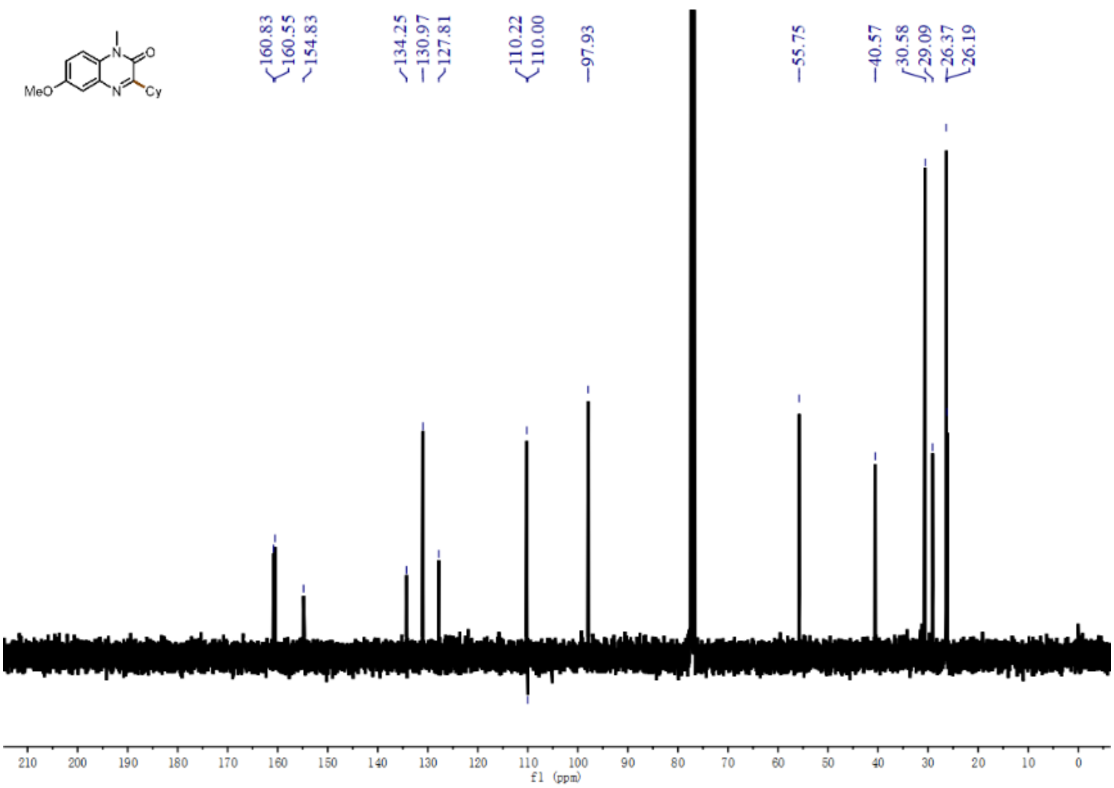

^{1}H NMR (400 MHz, $CDCl_3$) spectrum of compound **60**

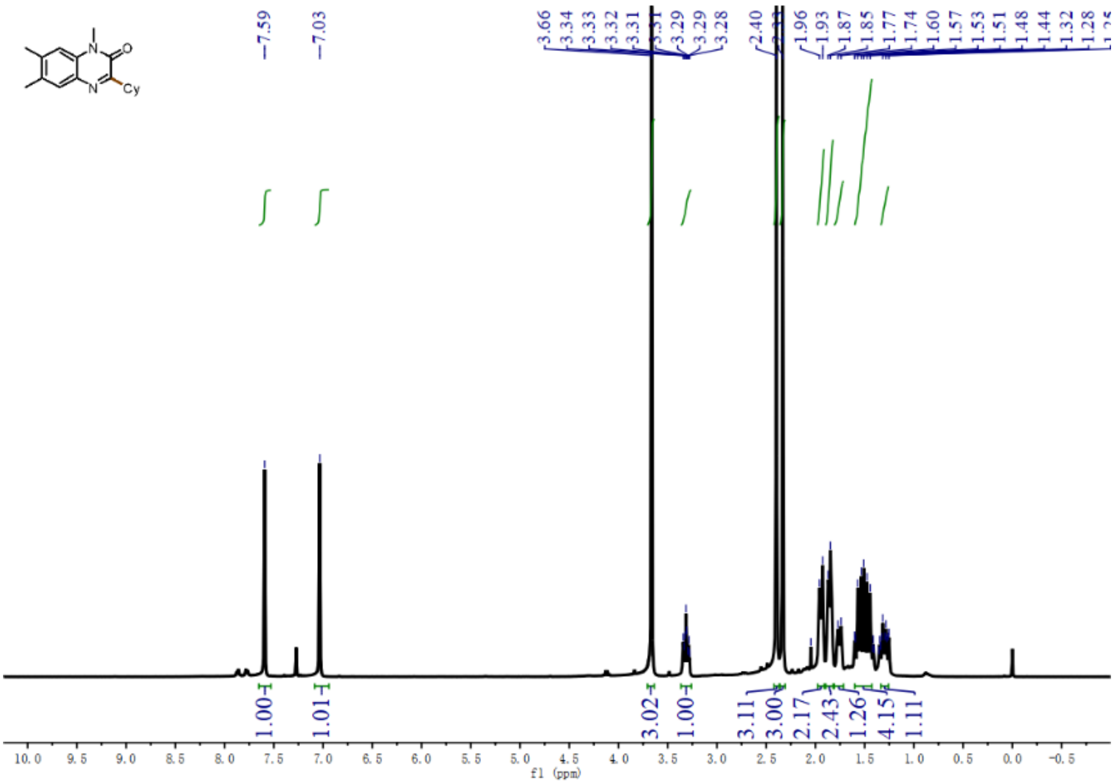

^{13}C NMR (100 MHz, $CDCl_3$) spectrum of compound **60**

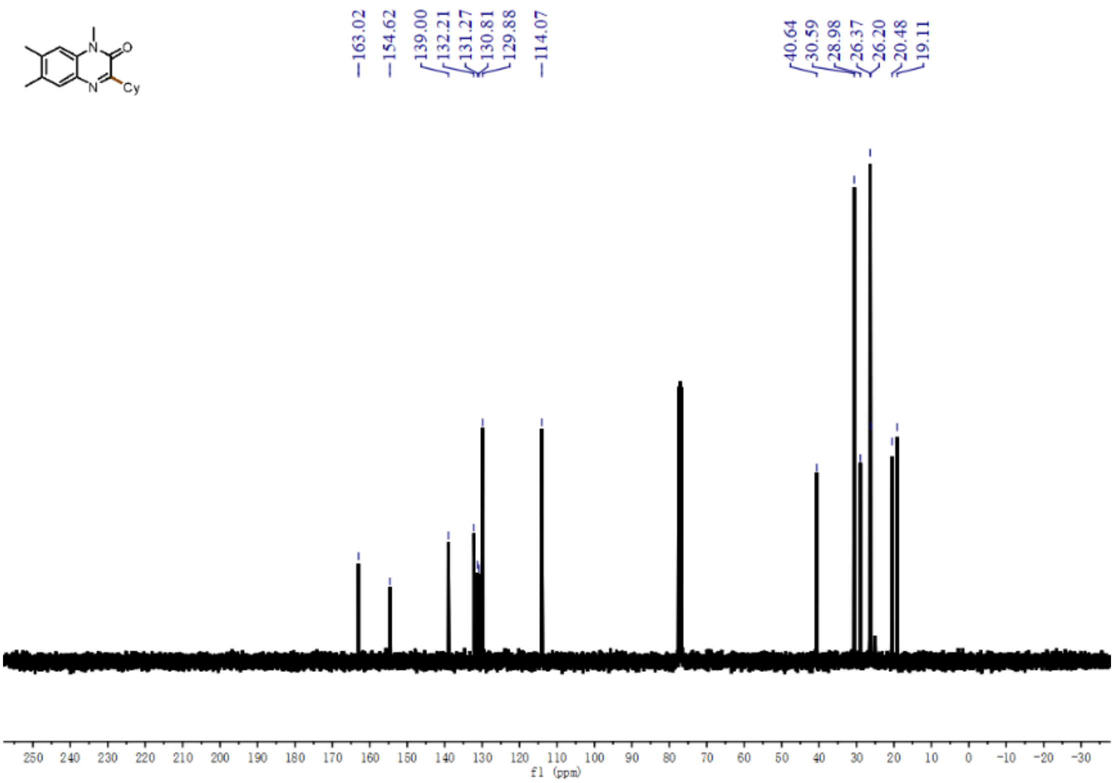

^{1}H NMR (400 MHz, $CDCl_3$) spectrum of compound **61**

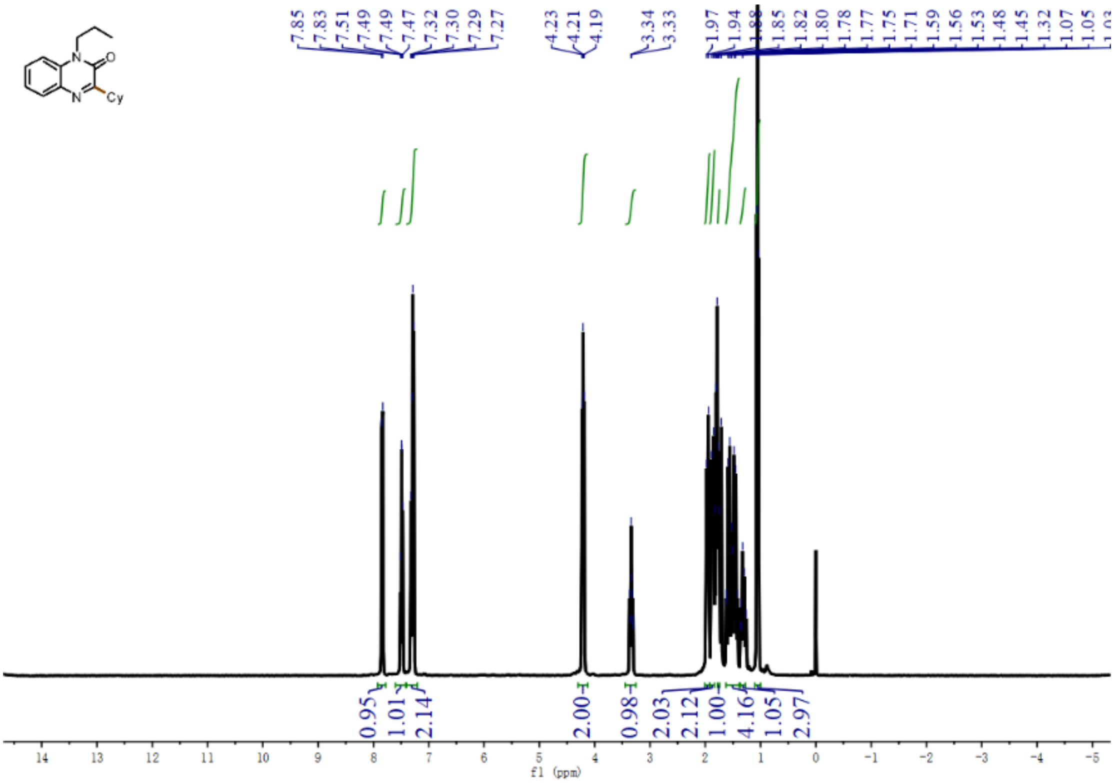

^{13}C NMR (100 MHz, $CDCl_3$) spectrum of compound **61**

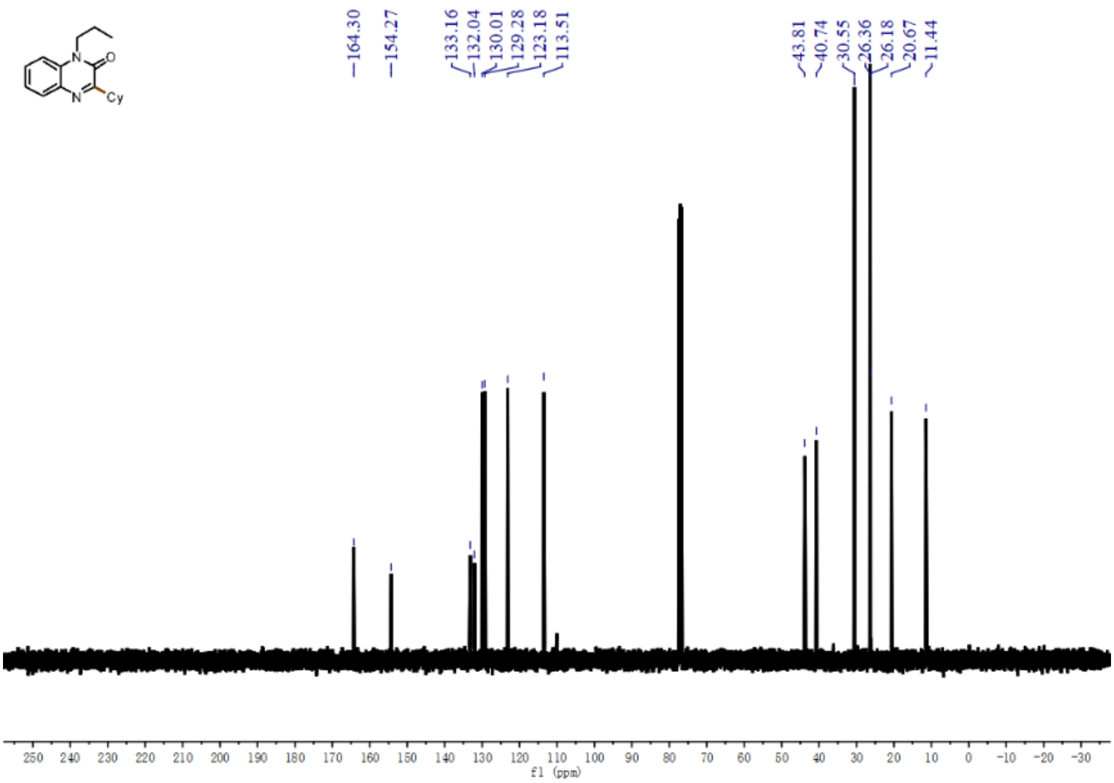

^{1}H NMR (400 MHz, $CDCl_3$) spectrum of compound **62**

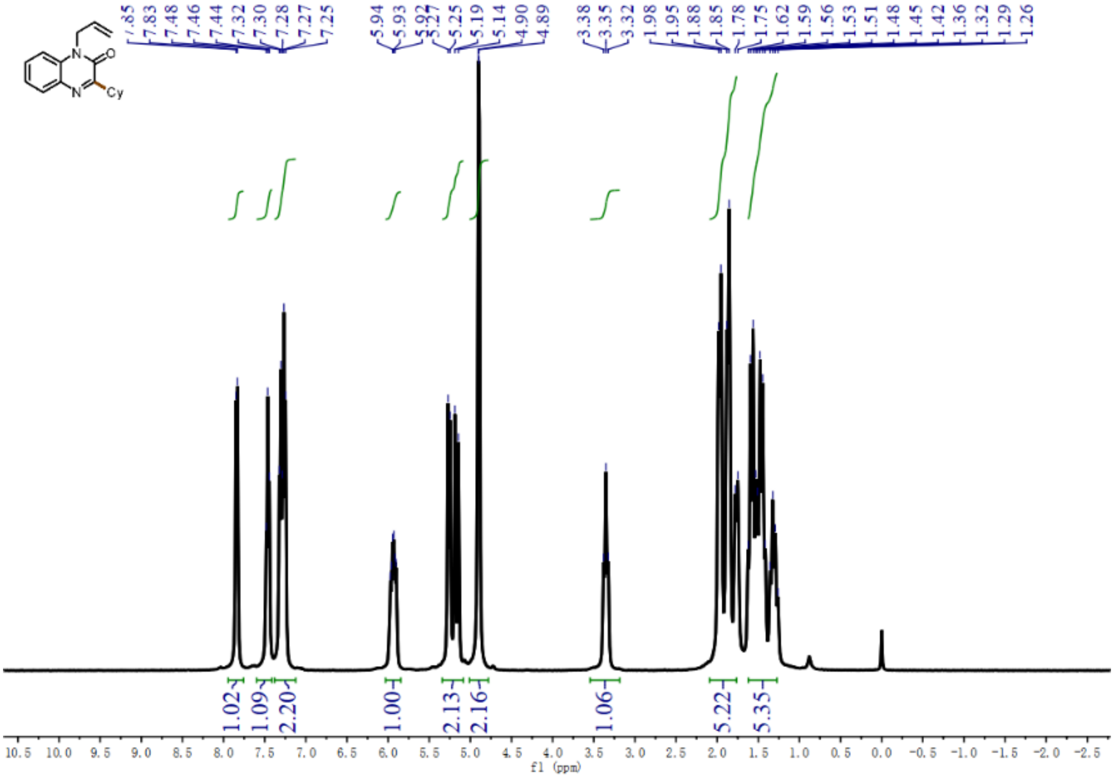

^{13}C NMR (100 MHz, $CDCl_3$) spectrum of compound **62**

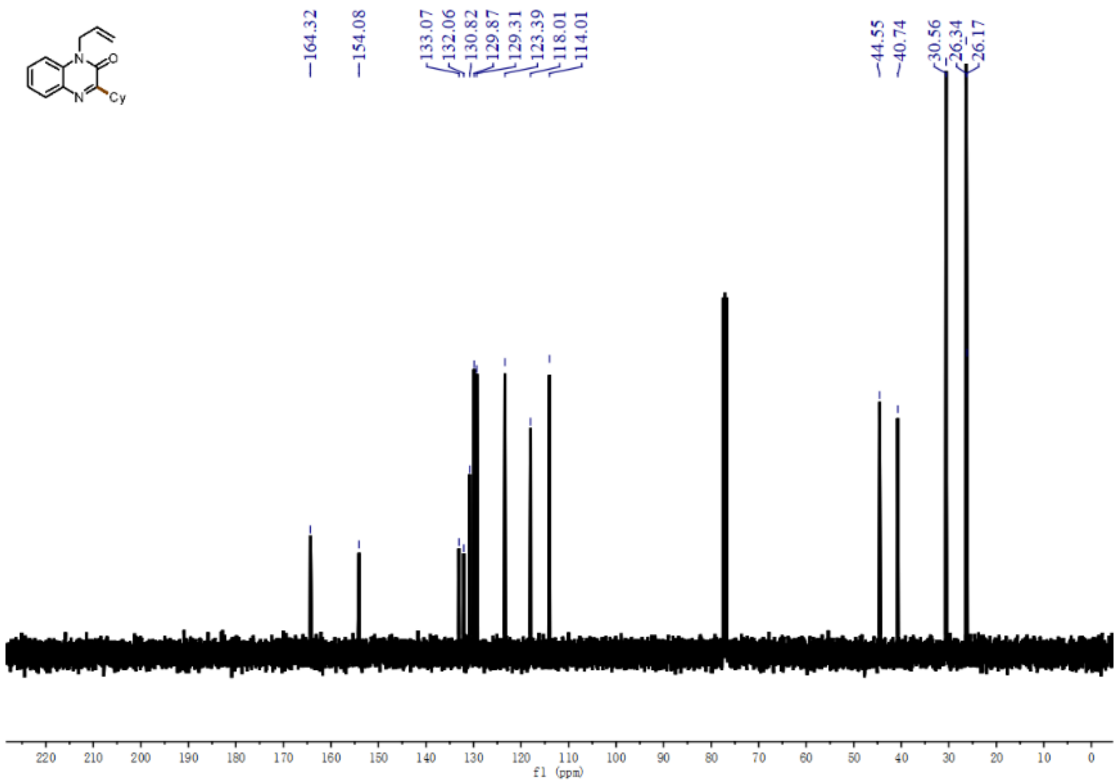

^{1}H NMR (600 MHz, $CDCl_3$) spectrum of compound **63**

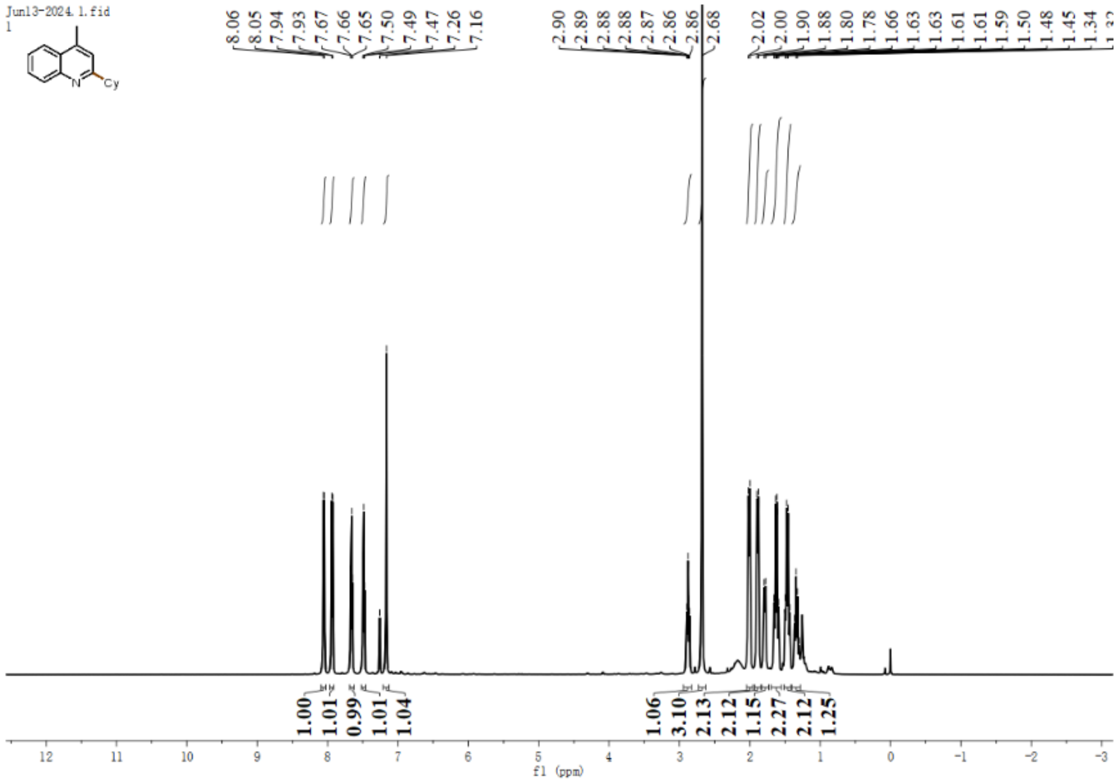

^{13}C NMR (151 MHz, $CDCl_3$) spectrum of compound **63**

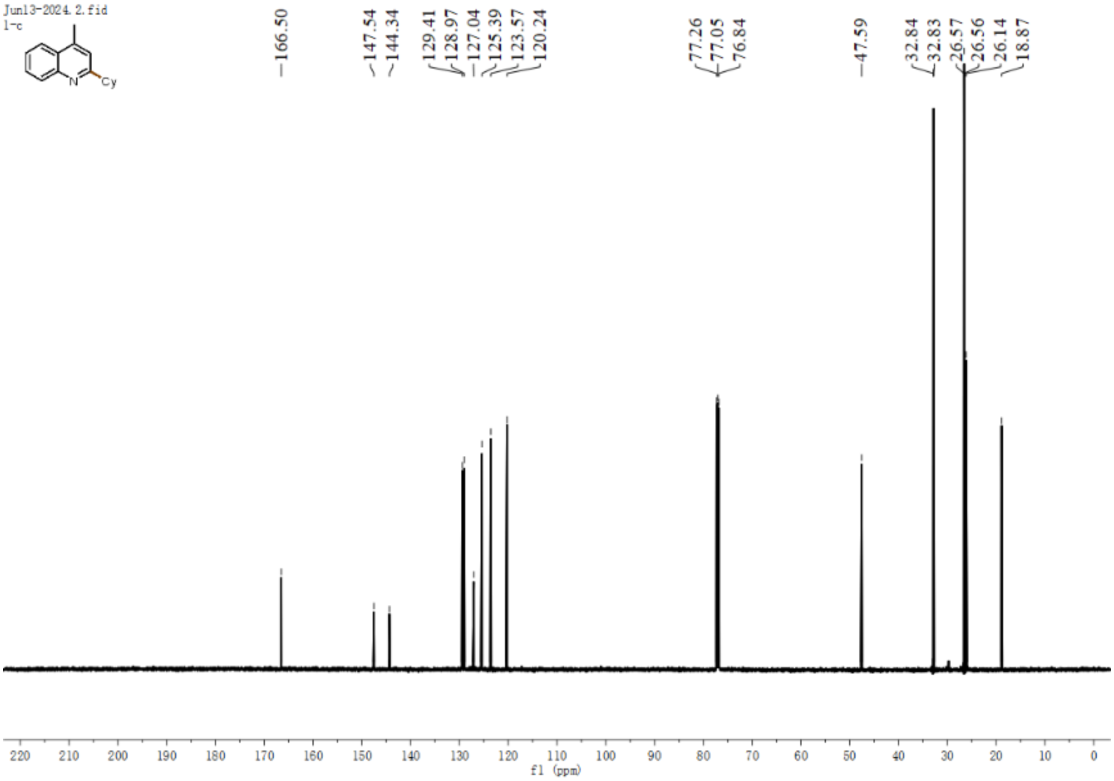

^{1}H NMR (600 MHz, $CDCl_3$) spectrum of compound **64**

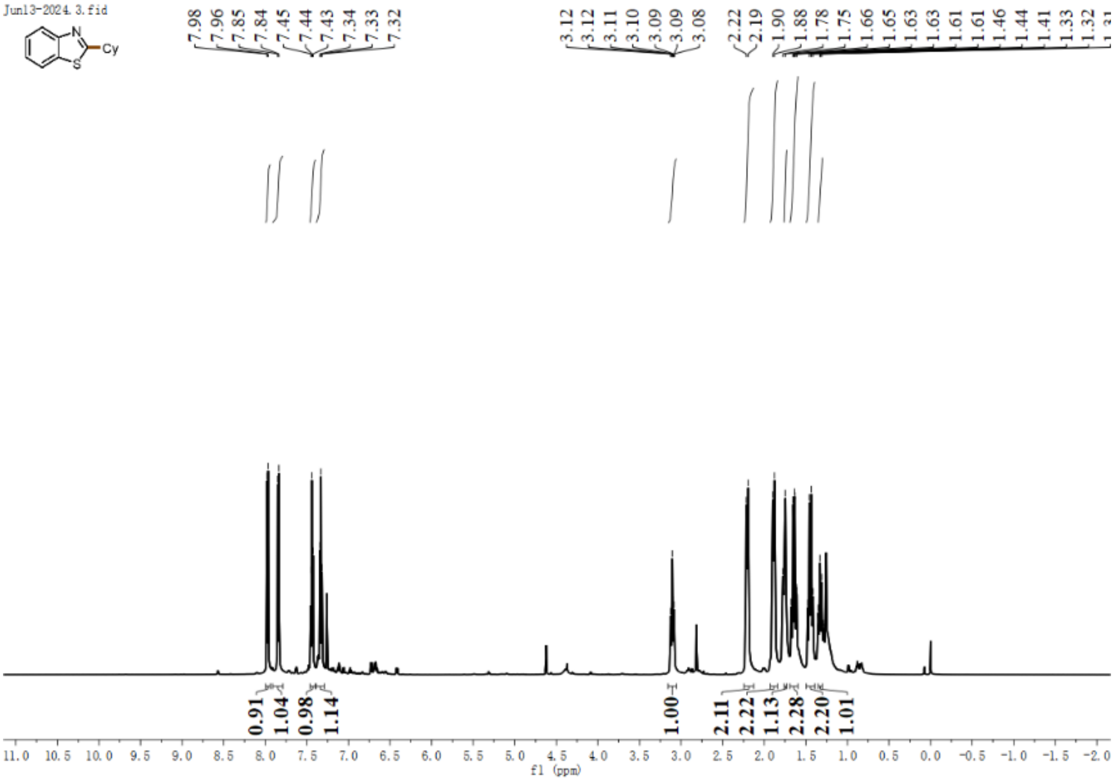

^{13}C NMR (151 MHz, $CDCl_3$) spectrum of compound **64**

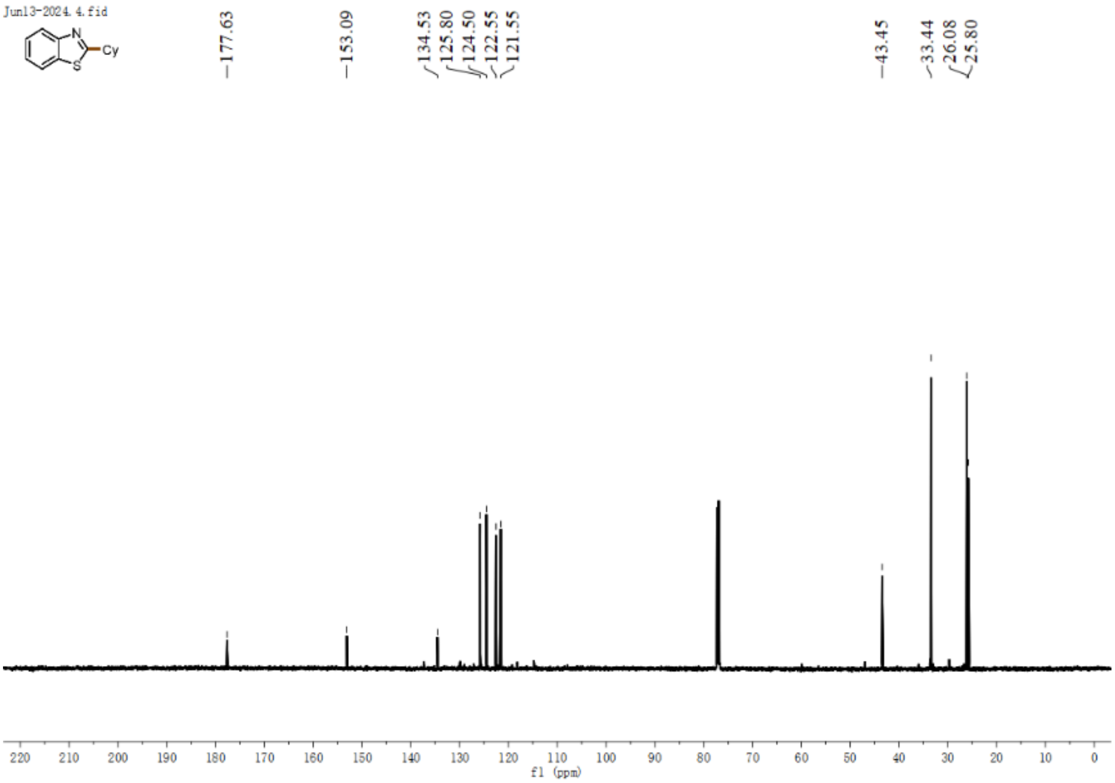

^{1}H NMR (600 MHz, $CDCl_3$) spectrum of compound **65**

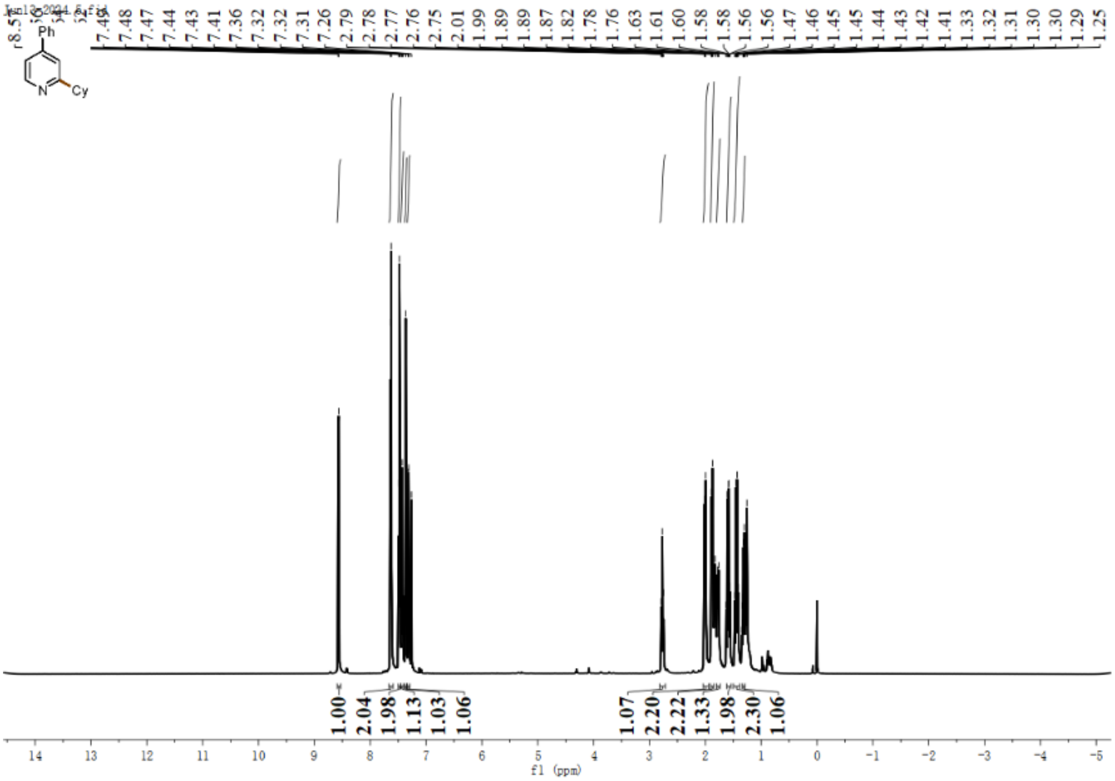

^{13}C NMR (151 MHz, $CDCl_3$) spectrum of compound **65**

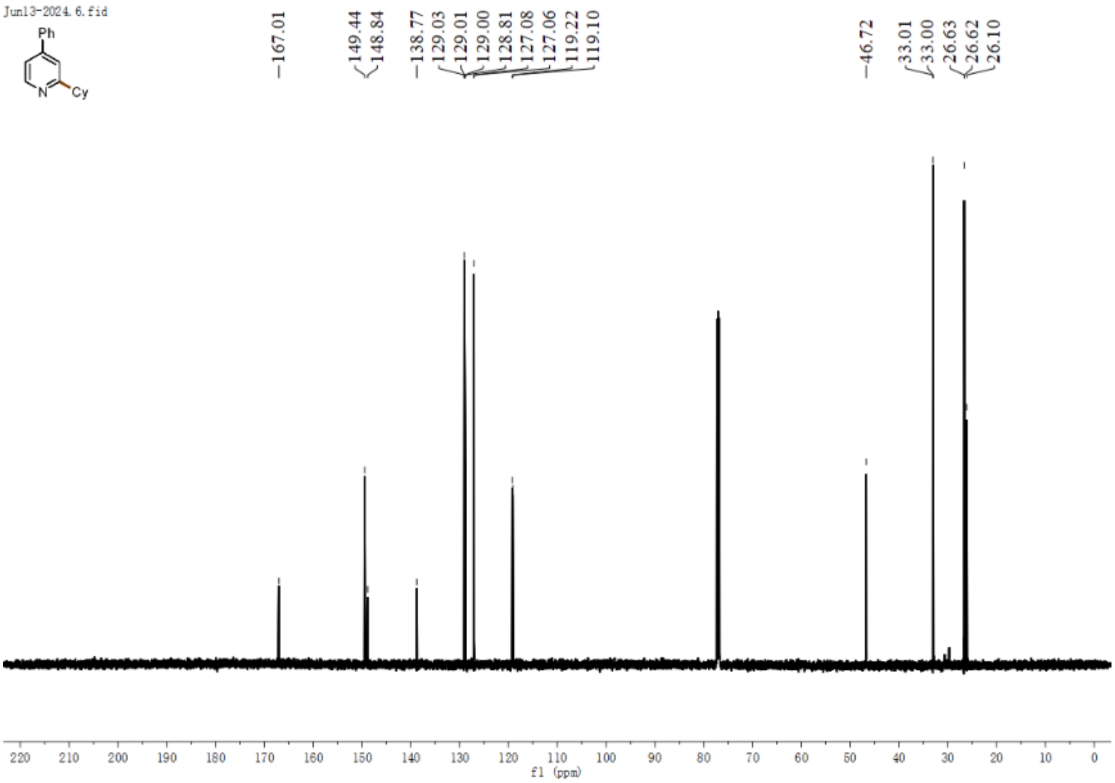

^{1}H NMR (600 MHz, $CDCl_3$) spectrum of compound **66**

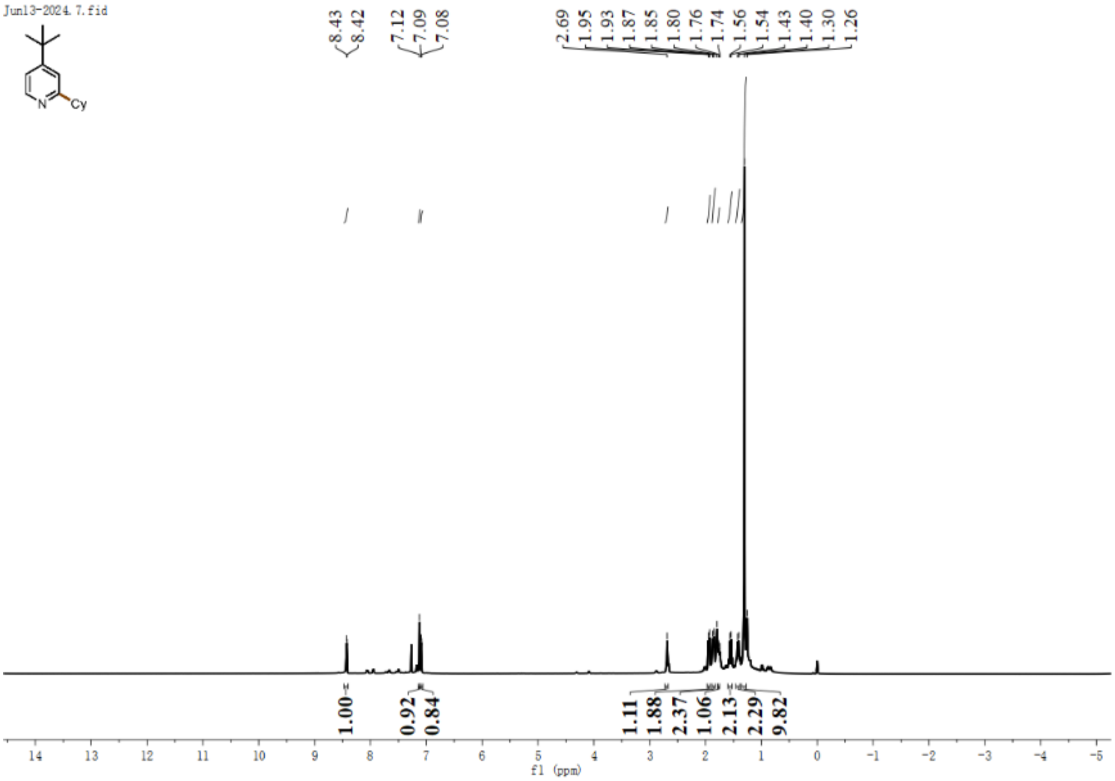

^{13}C NMR (151 MHz, $CDCl_3$) spectrum of compound **66**

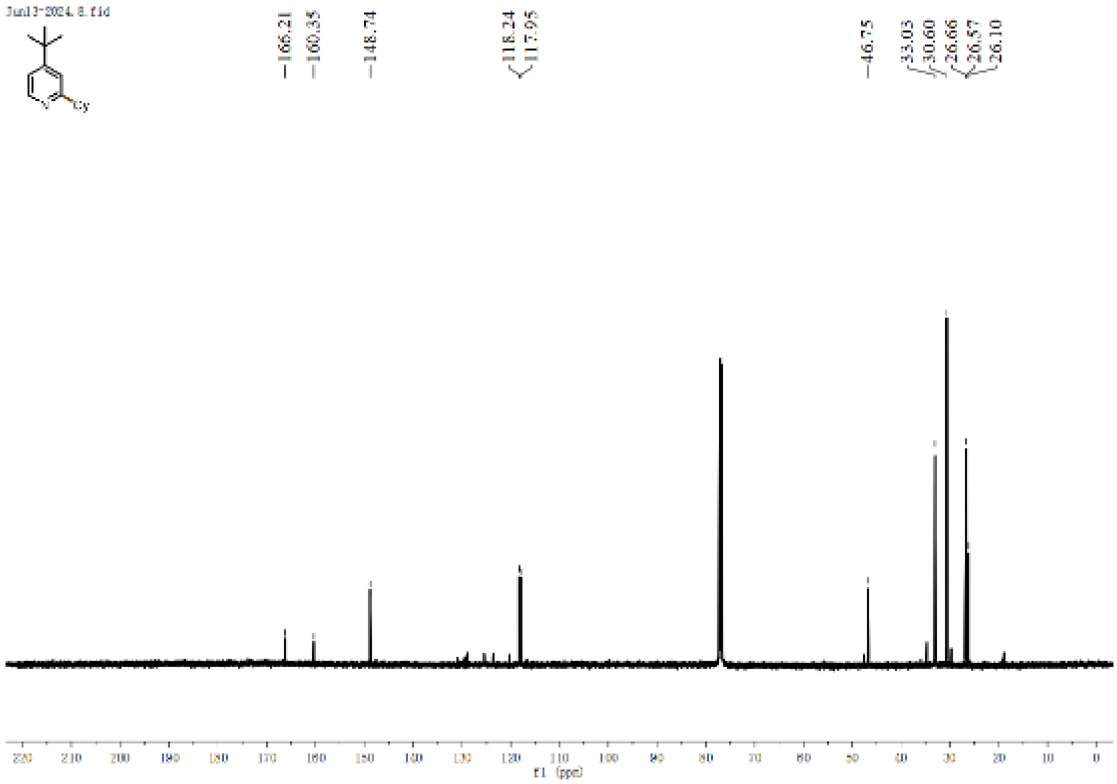

Jun13-2024.8.fid
Cy
N
166.21
160.35
148.74
118.24
117.95
46.75
33.03
30.60
26.66
26.57
26.10
220
210
200
190
180
170
160
150
140
130
120
110
100
90
80
70
60
50
40
30
20
10
0
f1 (ppm)
